# Detection of fusion gene transcripts in the blood samples of prostate cancer patients

**DOI:** 10.1038/s41598-021-96528-9

**Published:** 2021-08-20

**Authors:** Yan-Ping Yu, Silvia Liu, Joel Nelson, Jian-Hua Luo

**Affiliations:** Department of Pathology and Urology, School of Medicine, University of Pittsburgh, Scaife S-728, Pittsburgh, PA 15261 USA

**Keywords:** Cancer, Biomarkers, Urology

## Abstract

Prostate cancer remains one of the most lethal cancers for men in the United States. The study aims to detect fusion transcripts in the blood samples of prostate cancer patients. We analyzed nine fusion transcripts including MAN2A1-FER, SLC45A2-AMACR, TRMT11-GRIK2, CCNH-C5orf30, mTOR-TP53BP1, KDM4-AC011523.2, TMEM135-CCDC67, LRRC59-FLJ60017 and Pten-NOLC1147 in the blood samples from 147 prostate cancer patients and 14 healthy individuals, using Taqman RT-PCR and Sanger’s sequencing. Similar analyses were also performed on 25 matched prostate cancer samples for matched-sample evaluation. Eighty-two percent blood samples from the prostate cancer patients were positive for MAN2A1-FER transcript, while 41.5% and 38.8% blood samples from the prostate cancer patients were positive for SLC45A2-AMACR and Pten-NOLC1, respectively. CCNH-c5orf30 and mTOR-TP53BP1 had low detection rates, positive in only 5.4% and 4% of the blood samples from the prostate cancer patients. Only 2 blood samples were positive for KDM4B-AC011523.2 transcript. Overall, 89.8% patients were positive for at least one fusion transcript in their blood samples. The statistical analysis showed varied sensitivity of fusion transcript detection in the blood based on the types of fusions. In contrast, the blood samples from all healthy individuals were negative for the fusion transcripts. Detection of fusion transcripts in the blood samples of the prostate cancer patients may be a fast and cost-effective way to detect prostate cancer.

## Introduction

Prostate cancer is one of the most frequent cancers for men. In 2020, there were 248,530 new cases of prostate cancer in the United States^1^. Prostate cancer accounted for 34,130 deaths in the same year. Early diagnosis of prostate cancer generally produces excellent clinical outcome through surgical resection or radiation of the organ-confined prostate cancer, while late-stage diagnosis is associated with high mortality rate^2^. Currently, the primary screening tool for prostate cancer detection is quantifying the serum prostate-specific antigen (PSA) level^3^. When PSA level is high, prostate cancer is suspected. Other cancer detection approaches such as rectal digital examination, ultrasound or MRI are required. Ultimately, a biopsy of the prostate gland is necessary to confirm the diagnosis. A variety of other conditions may cause an increase of the serum PSA level. Even with the serum PSA level between 4 to 10 ng/ml, there are only 25% chances that prostate cancer is present^4^. The ambiguity of the interpretation leads to the delay of the diagnosis. The late diagnosis may negatively impact the clinical outcomes of the cancer treatment.

Recent studies showed that many fusion genes are present in the prostate cancer samples, ranging from as low as 2.5% to as high as 80% rate^5–7^. One study showed that up to 80% of prostate cancer samples were positive for Pten-NOLC1 gene fusion in the prostate cancer samples^6^, while the other study showed that a panel of eight fusion genes including MAN2A1-FER, SLC45A2-AMACR, TRMT11-GRIK2, TMEM135-CCDC67, CCNH-C5orf30, LRRC59-FLJ60017, KDM4B-AC011523.2 and mTOR-TP53BP1 occurred in prostate cancer samples with various frequencies^7^. The presence of these fusion genes was associated with aggressive prostate cancers^7^. The underlying mechanism for the generation of these fusion genes is the chromosome rearrangement in the cancer genome. Thus, unlike PSA, these fusion genes only occur in cancerous conditions. Pten-NOLC1, MAN2A1-FER and SLC45A2-AMACR fusion genes have been shown to be the drivers of cancers^6,8,9^. The detection of these fusion genes implicates the presence of cancer cells. The shedding of fusion transcripts from the cancer cells into the serum or other body fluids of cancer patients was previously reported^10,11^. To investigate whether the fusion RNA is present in the blood samples of prostate cancer patients to detect prostate cancer, we analyzed nine fusion transcripts in the blood samples of 147 prostate cancer patients, and found that the fusion transcripts were frequently detectable in the blood samples of the prostate cancer patients.

## Materials and methods

### Tissue samples

The 187 samples analyzed in this study consisted of 25 prostate cancer samples, 147 whole blood samples from prostate cancer patients (supplemental table 1) and 14 blood samples from healthy individuals (no known malignancy) with ages from 51 to 86. The blood samples from the prostate cancer patients were collected after the diagnosis of prostate cancer on prostate biopsy but before any therapeutic intervention. We obtained these samples from the University of Pittsburgh Tissue Bank. The procedure complied with institutional regulatory guidelines. The informed consent exemptions and tissue processing protocol were reviewed and approved by the Institution Review Board of the University of Pittsburgh. All blood samples and prostate cancer samples were fresh-frozen and stored at − 80 °C.

### RNA extraction, cDNA synthesis, and detection of fusion genes

The procedures for RNA extraction, cDNA synthesis, and fusion gene detection are similar to those described previously^6,8,12–19^. Briefly, total RNA from the cells was extracted using Trizol (Invitrogen, CA). The quality of the extracted RNA was assessed through 260/280 and 260/230 ratio analyses by Nanodrop™ spectrophotometer. The samples passing the quality control were accepted for further analysis. The first stranded cDNA was synthesized from ~ 2 µg of the total RNA template from each sample. Random hexamers and Superscript II™ (Invitrogen, Inc, CA) were incubated with the RNA at 42 °C for 2 h. One microliter of each cDNA sample was used for the TaqMan PCR reactions with 50 heat cycles, as follows: 94 °C for 30 s, 61 °C for 30 s, and 72 °C for 30 s, using the primers and probes^6,9,10,19^ listed in supplemental table 2. The PCR reactions were performed in a thermocycler (QuantStudio 3 real time PCR system, Thermofisher, Inc). The 50 cycle is a standard clinical procedure detecting fusion transcripts in highly fragmented RNA and suboptimal tissue samples. A negative control and a synthetic positive control (Supplemental table 3, IDT Inc, Coralville, Iowa) were included in each batch of the reactions. The PCR products from 20 to 100% of the positive samples were sequenced using Sanger sequencing method.

### Statistical analysis

Patients were categorized into fusion-positive and fusion-negative groups. To check the association between fusion status in blood samples and clinical features, Wilcox test was applied to continuous clinical variables (5-year and 10-year NOMOGRAM) and Fisher’s exact test was applied to categorical clinical variables (Gleason’s grading). *p* value ≤ 0.05 was used to define significance. All the statistical analysis was performed by R programming.

## Results

In this analysis, we analyzed nine fusion transcripts in 147 blood samples from prostate cancer patients and 14 blood samples from healthy individuals. These blood samples were collected after the diagnosis of prostate cancer and before any therapeutic intervention. The fusion transcripts in the blood samples were detected through Taqman real-time RT-PCR as described in the methods. As shown in Table 1, supplemental table 1 and supplemental figures 1 through 11, up to 89.8% (132/147) blood samples from the prostate cancer samples are positive for at least one type of the fusion transcripts, while all 14 samples from healthy individuals were negative for any type of the fusion transcripts. MAN2A1-FER, a fusion gene encoding for a tyrosine protein kinase, was present in 82.3% (121/147) blood samples from the prostate cancer patients, representing the most frequent fusion transcript detected in the blood of prostate cancer patients. The next most frequent fusion transcripts present in the blood samples from the prostate cancer patients were SLC45A2-AMACR and Pten-NOLC1, accounting for 41.5% (61/147) and 38.8% (57/147), respectively. Only small percentages of blood samples from the prostate cancer patients were positive for CCNH-C5orf30 (5.4% or 8/147), mTOR-TP53BP1 (4.1% or 6/147) and KDM4B-AC011523.2 (1.4% or 2/147). TRMT11-GRIK, LRRC59-FLJ60017 and TMEM135-CCDC67 were negative for all the tested blood samples.

**Table 1:** Fusion transcripts detected in the blood samples of prostate cancer patients.

Fusion	MAN2A1-FER	TRMT11-GRIK2	mTOR-TP53BP1	CCNH-c5orf30	KDM4B-AC011523.2	SLC45A2-AMACR	TMEM135-CCDC67	LRRC59-FLJ60017	Pten-NOLC1	Any fusion
	82.3% (121/147)	0% (0/147)	4% (6/147)	5.4% (8/147)	1.4% (2/147)	41.5% (61/147)	0% (0/147)	0% (0/147)	38.8% (57/147)	89.8% (132/147)
Pre-operation PSA	7.36	N/A	3.83	9.64	8.43	7.67	N/A	N/A	7.60	7.89
Gleason 6	81.3% (13/16)	0% (0/16)	6.3% (1/16)	0% (0/16)	6.3% (1/16)	37.5% (6/16)	0% (0/16)	0% (0/16)	62.5% (10/16)	87.5% (14/16)
Gleason 7	81% (81/100)	0% (0/100)	5% (5/100)	5% (5/100)	1% (1/100)	42% (42/100)	0% (0/100)	0% (0/100)	38% (38/100)	90% (90/100)
Gleason 8	91.7% (11/12)	0% (0/12)	0% (0/12)	8.3% (1/12)	0% (0/12)	50% (6/12)	0% (0/12)	0% (0/12)	41.7% (5/12)	91.7% (11/12)
Gleason 9	83.3% (15/18)	0% (0/18)	0% (0/18)	11.1% (2/18)	0% (0/18)	33.3% (6/18)	0% (0/18)	0% (0/18)	16.7% (3/18)	88.9% (16/18)
Gleason 10	100% (1/1)	0% (0/1)	0% (0/1)	0% (0/1)	0% (0/1)	100% (1/1)	0% (0/1)	0% (0/1)	100% (1/1)	100% (1/1)
T2a	78.6% (11/14)	0% (0/14)	0% (0/14)	7.1% (1/14)	0% (0/14)	35.7% (5/14)	0% (0/14)	0% (0/14)	42.9% (6/14)	92.9% (13/14)
T2c	77.3% (51/66)	0% (0/66)	6.1% (4/66)	4.5% (3/66)	1.5% (1/66)	45.5% (30/66)	0% (0/66)	0% (0/66)	40.9% (27/66)	87.9% (58/66)
T3a	92.3% (48/52)	0% (0/52)	3.8% (2/52)	5.8% (3/52)	1.9% (1/52)	42.3% (22/52)	0% (0/52)	0% (0/52)	40.4% (21/52)	94.2% (49/52)
T3c	73.3% (11/15)	0% (0/15)	0% (0/15)	6.7% (1/15)	0% (0/15)	26.7% (4/15)	0% (0/15)	0% (0/15)	20% (3/15)	80% (12/15)
N0	82.4% (108/131)	0% (0/131)	4.6% (6/131)	4.6% (6/131)	1.5% (2/131)	42.7% (56/131)	0% (0/131)	0% (0/131)	41.22 (54/131)	90.8% (118/131)
N1	81.3% (13/16)	0% (0/16)	0% (0/16)	12.5% (2/16)	0% (0/16)	31.25% (5/16)	0% (0/16)	0% (0/16)	18.8% (3/16)	87.5% (14/16)
5 year NOMOGRAM										
95–99	25% (3/12)	0% (0/12)	8.3% (1/12)	8.3% (1/12)	0% (0/12)	41.7% (5/12)	0% (0/12)	0% (0/12)	33.3% (4/12)	58.3% (7/12)
90–94	50% (2/4)	0% (0/4)	0% (0/4)	0% (0/4)	0% (0/4)	50% (2/4)	0% (0/4)	0% (0/4)	50% (2/4)	100% (4/4)
80–89	88.9% (8/9)	0% (0/9)	11.1% (1/9)	22.2% (2/9)	11.1% (1/9)	44.4% (4/9)	0% (0/9)	0% (0/9)	33.3% (3/9)	88.9% (8/9)
60–79	72.7% (8/11)	0% (0/11)	0% (0/11)	18.2% (2/11)	0% (0/11)	45.5% (5/11)	0% (0/11)	0% (0/11)	18.2% (2/11)	81.8% (9/11)
10 year NOMOGRAM										
90–99	25% (3/12)	0% (0/12)	8.3% (1/12)	8.3% (1/12)	0% (0/12)	41.7% (5/12)	0% (0/12)	0% (0/12)	33.3% (4/12)	58.3% (7/12)
80–89	60% (3/5)	0% (0/5)	0% (0/5)	20% (1/5)	0% (0/5)	40% (2/5)	0% (0/5)	0% (0/5)	40% (2/5)	100% (5/5)
60–79	81.8% (9/11)	0% (0/11)	9.1% (1/11)	9.1% (1/11)	9.1% (1/11)	45.5% (5/11)	0% (0/11)	0% (0/11)	27.3% (3/11)	90.9% (10/11)
40–59	75% (6/8)	0% (0/8)	0% (0/8)	25% (2/8)	0% (0/8)	50% (4/8)	0% (0/8)	0% (0/8)	25% (2/8)	75% (6/8)
Healthy individuals	0% (0/14)	0% (0/14)	0% (0/14)	0% (0/14)	0% (0/14)	0% (0/14)	0% (0/14)	0% (0/14)	0% (0/14)	0% (0/14)

**Figure 1:**
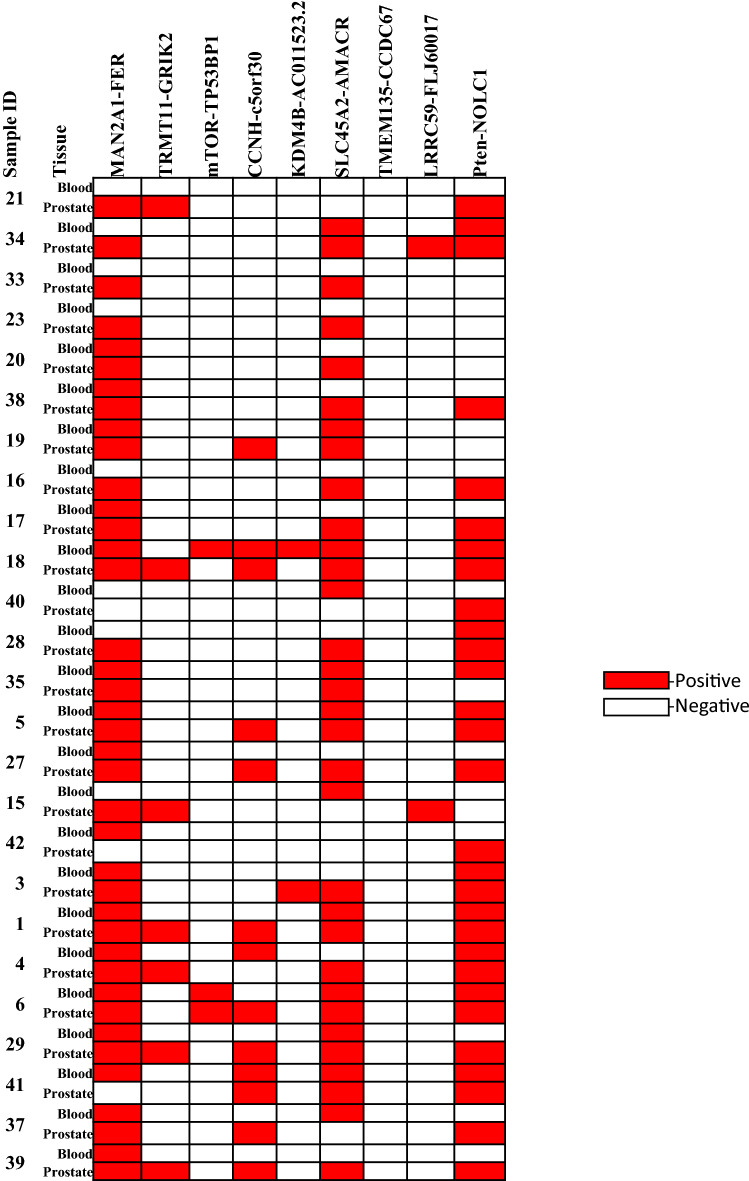
Heatmap fusion genes detected in prostate cancer and blood samples from the same patients. Case number is indicated. Red color indicates a positive detection of the indicated fusion transcript. Blank indicates a negative detection.

To examine whether the fusion transcripts in the blood samples reflect the transcripts in the prostate cancer samples in the same individuals, 25 prostate cancers were examined for the presence of the nine fusion genes. As shown in Fig. 1 and Table 2, all 25 prostate cancer samples were positive for at least one fusion gene. For MAN2A1-FER, 88% (22/25) prostate cancer samples were positive, while only 68% (17/25) matched blood samples were detected to contain the same transcript. The sensitivity of MAN2A1-FER detected in the blood is 68.2% (15/22). The positive predictive value for MAN2A1-FER in the blood samples is 88.2% (15/17). Similar conditions were also found in SLC45A2-AMACR and Pten-NOLC1 fusion: 80% (20/25) SLC45A2-AMACR and 76% (19/25) Pten-NOLC1 were detected in the prostate cancer samples, while the detection rate dropped to 48% (12/25) in the matched blood samples for SLC45A2-AMACR and 40% (10/25) for Pten-NOLC1. The sensitivity of SLC45A2-AMACR in the blood is 45% (9/20), while the sensitivity of Pten-NOLC1 is 47.4% (9/19). The positive prediction rate for SLC45A2-AMACR in blood versus prostate cancer reached 75% (9/12), while the positive prediction rate for Pten-NOLC1 was 90% (9/10). Nine prostate cancer samples were positive for CCNH-c5orf30. However, only 2 matched blood samples were found to contain CCNH-c5orf30 fusion transcript (22.2% sensitivity or 2/9, and a positive prediction rate 66.7% or 2/3). Twenty-eight percent (7/25) of the prostate cancer samples were positive for TRMT11-GRIK2 fusion, but no blood samples were found to have the fusion transcript. Two prostate cancer samples were also found to contain LRRC59-FLJ60017 fusion but not in the blood. The small numbers of blood sample tests from prostate cancer patients who were positive for the fusion transcript but negative for the matched prostate sample may reflect significant heterogeneity of prostate cancers in terms of expression of the fusion genes. Sampling errors of the micro-dissected prostate cancer may account for the discrepancy.

**Table 2:** Concordance statistics of the matched prostate cancer and blood samples.

Statistics	MAN2A1-FER	TRMT11-GRIK2	mTOR-TP53BP1	CCNH-c5orf30	KDM4B-AC011523.2	SLC45A2-AMACR	TMEM135-CCDC67	LRRC59-FLJ60017	Pten-NOLC1
Prostate fusion sensitivity	88% (22/25)	28% (7/25)	4% (1/25)	36% (9/25)	4% (1/25)	80% (20/25)	0% (0/25)	8% (2/25)	76% (19/25)
Blood fusion sensitivity	68% (15/25)	0% (0/25)	100% (1/1)	22.2% (2/9)	0% (0/1)	45% (9/20)	N/A	0% (0/2)	47.4% (9/19)
Blood fusion positive prediction value	88.2% (15/17)	N/A	50% (1/2)	66.7% (2/3)	0% (0/1)	75% (9/12)	N/A	N/A	90% (9/10)

Association analysis showed that positive Pten-NOLC1 fusion transcript detection in the blood sample is associated with better cancer differentiation (lower Gleason’s grading, *p* = 0.042), with 62.5% Gleason 6 is positive for Pten-NOLC1, while only 38.4% for Gleason 7-8 and 16.7% for Gleason 9-10 (Table 1). Nomogram is a clinical algorithm that has been used as a tool in gauging the likelihood of clinical outcomes of radical prostatectomy^20^. The pre-surgery nomogram incorporates the information of patient’s age, pre-operation PSA level, Gleason’s grade, clinical stage, the percentage of the positive biopsy cores^21^. It has been shown that low Nomogram scores are closely associated with the likelihood of prostate cancer recurrence^22–24^ . Interestingly, the presence of MAN2A1-FER in blood was associated with lower NOMOGRAM score (p-values equal to 0.010 and 0.019 for 5-year and 10-year NOMOGRAM respectively): Only 25% patients with 5-year NOMOGRAM scores between 95 and 99 (low risk of recurrence) were positive for MAN2A1-FER in the blood samples, while the positive rate reached 72.7% for patients with scores between 60 and 79 (high risk of recurrence). Similar associations were also identified for 10-year NOMOGRAM: only 25% patients with 10-year scores between 90 and 99 were positive for MAN2A1-FER in the blood, while 75% patients with scores between 40 and 59 were positive for the fusion genes.

## Discussion

Gene fusion is one of the most frequent events in prostate cancer^25^. The underlying cause of gene fusion is abnormal chromosome recombination. Previous studies showed that gene fusion MAN2A1-FER, Pten-NOLC1 and SLC45A2-AMACR are the cancer drivers^6,8,9^. Thus, detection of these fusion transcripts in the blood samples implicates the presence of cancer cells in the body. The high positive prediction rates for MAN2A1-FER (88.2%), SLC45A2-AMACR (75%) and Pten-NOLC1 (90.9%) between blood samples and the primary prostate cancer samples suggests that the source of the fusion transcripts in the blood samples is likely prostate cancer. Our results suggest that cancer cells were constantly dislodged from the tumor, and traveled in the bloodstream. Alternatively, the presence of fusion transcript fragments in the blood may reflect the shedding of fusion RNA pieces from the lysed cancer cells. Either way, the detection of cancer fusion transcripts in the blood opened a new window to examine whether prostate cancer cells are present. Interestingly, the detection of MAN2A1-FER is highly correlated with the low nomogram score, suggesting that blood MAN2A1-FER may have a prognostic value. Future clinical outcome analysis may reveal whether blood MAN2A1-FER is associated with poor clinical outcomes of the prostate cancer.

Blood screening of prostate cancer is probably the most cost-effective way for early diagnosis of the disease. Currently, serum PSA has been the surrogate marker for prostate cancer^26^. However, PSA is a serine protease and a physiological product expressed in prostate acinar cells^27^. It is present in normal prostate tissues and several other organs. As a result, interpretation of the results from serum PSA level can be ambiguous and requires significant elevation of the PSA level to suspect the presence of prostate cancer. Furthermore, other benign conditions such as prostatitis, hyperplasia, or aging may also lead to increased PSA level^28–30^. The fusion transcript blood test described in this study offers an alternative blood screening test to detect prostate cancer. Despite these fusion genes can be associated with other human malignancies^6,8–10,19^, the combination radiology imaging and the fusion gene test may help to identify the location of the cancer lesion. There are two distinctions for the fusion gene blood test for the prostate cancer: First, these transcripts are the products of the cancer cells. They are not present in the normal prostate. The detection of these fusion transcripts signals the presence of cancer. Thus, the interpretation is qualitative. Second, several fusion genes described in this study are the cancer drivers. They are targetable by small molecules or genome editing^31^. The detection of these fusion transcripts allows the proper selection of targeting drugs to treat cancer.

Despite all the samples in the study had been stored for at least 5 years, the detection of the fusion transcripts in the blood remains robust, probably due to our primer and probe design adapting to the fragmented RNA. The sensitivity of the fusion transcripts detected in the blood samples from the prostate cancer patients appeared to vary among different types of fusion genes. The most sensitive fusion transcript is MAN2A1-FER, reaching 82.3%, while several fusion transcripts such as TRMT11-GRIK2 were mostly not detectable in the blood even they were present in the prostate cancer samples. The causes of such variability could be due to the varied vulnerability of different fusion RNA sequences to RNAse in the blood. Despite the varied sensitivities of the fusion transcript detection, when multiple fusion transcripts were put into the test, close to 90% blood samples from prostate cancer patients were positive for at least one fusion gene, making the fusion gene blood test a potential clinical screening method for the diagnosis of prostate cancer. In comparison with the test of circulating tumor cell RNA (ctRNA), whole blood fusion gene test using Taqman RT-PCR bypasses the laborious procedure of tumor cell isolation that is essential for ctRNA, and requires much less volume of blood than that of ctRNA test. Taqman RT-PCR described in this study can be adopted into droplet digital RT-PCR easily. Overall, fusion transcript detection in the blood may be a promising method to screen prostate cancer.

## Supplementary Information

Supplementary Information 1.

Supplementary Information 2.

Supplementary Information 3.

Supplementary Information 4.

## References

[1] Siegel RL, Miller KD, Fuchs HE, Jemal A (2021). Cancer statistics, 2021. CA Cancer J. Clin..

[2] Siegel DA, O'Neil ME, Richards TB, Dowling NF, Weir HK (2020). Prostate cancer incidence and survival, by stage and race/ethnicity—United States, 2001–2017. MMWR Morb. Mortal Wkly. Rep..

[3] Ilic D (2018). Prostate cancer screening with prostate-specific antigen (PSA) test: a systematic review and meta-analysis. BMJ.

[4] Liu J (2020). Establishment of two new predictive models for prostate cancer to determine whether to require prostate biopsy when the PSA level is in the diagnostic gray zone (4–10 ng ml^−^^1^). Asian J. Androl..

[5] Tomlins SA (2005). Recurrent fusion of TMPRSS2 and ETS transcription factor genes in prostate cancer. Science (New York, N.Y.).

[6] Luo JH (2021). Pten-NOLC1 fusion promotes cancers involving MET and EGFR signalings. Oncogene.

[7] Yu YP (2014). Novel fusion transcripts associate with progressive prostate cancer. Am. J. Pathol..

[8] Chen ZH (2017). MAN2A1-FER fusion gene is expressed by human liver and other tumor types and has oncogenic activity in mice. Gastroenterology.

[9] Zuo, Z.-H. *et al.* Oncogenic activity of SLC45A2-AMACR gene fusion is mediated by mitogen-activated protein kinase. *Hepatol. Commun.*, in press (2021). 10.1002/hep4.1724 PMC8710797 34505419

[10] Yu YP (2019). Detection of fusion transcripts in the serum samples of patients with hepatocellular carcinoma. Oncotarget.

[11] Laxman B (2006). Noninvasive detection of TMPRSS2:ERG fusion transcripts in the urine of men with prostate cancer. Neoplasia (New York, N.Y.).

[12] Lin F (2001). Myopodin, a synaptopodin homologue, is frequently deleted in invasive prostate cancers. Am. J. Pathol..

[13] Yu YP (2004). Gene expression alterations in prostate cancer predicting tumor aggression and preceding development of malignancy. J Clin Oncol.

[14] Luo JH (2006). Transcriptomic and genomic analysis of human hepatocellular carcinomas and hepatoblastomas. Hepatology (Baltimore, MD).

[15] Luo JH (2013). Genome-wide methylation analysis of prostate tissues reveals global methylation patterns of prostate cancer. Am. J. Pathol..

[16] Yu YP (2013). Whole-genome methylation sequencing reveals distinct impact of differential methylations on gene transcription in prostate cancer. Am. J. Pathol..

[17] Yu YP (2015). Genomic copy number variations in the genomes of leukocytes predict prostate cancer clinical outcomes. PLoS ONE.

[18] He DM (2017). Oncogenic activity of amplified miniature chromosome maintenance 8 in human malignancies. Oncogene.

[19] Yu YP (2019). Identification of recurrent fusion genes across multiple cancer types. Sci. Rep..

[20] Shariat SF, Kattan MW, Vickers AJ, Karakiewicz PI, Scardino PT (2009). Critical review of prostate cancer predictive tools. Future Oncol..

[21] Kattan MW, Eastham JA, Stapleton AM, Wheeler TM, Scardino PT (1998). A preoperative nomogram for disease recurrence following radical prostatectomy for prostate cancer. J. Natl. Cancer Inst..

[22] Kattan MW (2001). Pretreatment nomogram for predicting freedom from recurrence after permanent prostate brachytherapy in prostate cancer. Urology.

[23] Lughezzani G (2012). Development and internal validation of a Prostate Health Index based nomogram for predicting prostate cancer at extended biopsy. J. Urol..

[24] Kattan MW (2000). Pretreatment nomogram for predicting the outcome of three-dimensional conformal radiotherapy in prostate cancer. J. Clin. Oncol..

[25] Luo JH (2015). Discovery and classification of fusion transcripts in prostate cancer and normal prostate tissue. Am. J. Pathol..

[26] Kuriyama M (1980). Quantitation of prostate-specific antigen in serum by a sensitive enzyme immunoassay. Can. Res..

[27] Rao AR, Motiwala HG, Karim OM (2008). The discovery of prostate-specific antigen. BJU Int..

[28] Neal DE, Clejan S, Sarma D, Moon TD (1992). Prostate specific antigen and prostatitis. I. Effect of prostatitis on serum PSA in the human and nonhuman primate. Prostate.

[29] Stephan C, Lein M, Jung K, Schnorr D, Loening SA (1997). The influence of prostate volume on the ratio of free to total prostate specific antigen in serum of patients with prostate carcinoma and benign prostate hyperplasia. Cancer.

[30] Oesterling JE, Cooner WH, Jacobsen SJ, Guess HA, Lieber MM (1993). Influence of patient age on the serum PSA concentration. An important clinical observation. Urol. Clin. North. Am..

[31] Chen ZH (2017). Targeting genomic rearrangements in tumor cells through Cas9-mediated insertion of a suicide gene. Nat. Biotechnol..

