# Supplementary material for: Detection of fusion gene transcripts in the blood samples of prostate cancer patients
Source: Sci Rep. 2021 Aug 20;11:16995. doi: 10.1038/s41598-021-96528-9 (PMC8379170; doi:10.1038/s41598-021-96528-9)
Supplement: Supplementary file 1 — Supplementary Information 1. [file 41598_2021_96528_MOESM1_ESM.pdf]

Supplemental figure 1. Taqman RT-PCR detection of MAN2A1-FER in the blood samples of prostate cancer patients.

Supplemental figure 2. Taqman RT-PCR detection of TRMT11-GRIK2 in the blood samples of prostate cancer patients.

Supplemental figure 3. Taqman RT-PCR detection of mTOR-TP53BP1 in the blood samples of prostate cancer patients.

Supplemental figure 4. Taqman RT-PCR detection of CCNH-c5orf30 in the blood samples of prostate cancer patients.

Supplemental figure 5. Taqman RT-PCR detection of KDM4B-AC011523.2 in the blood samples of prostate cancer patients.

Supplemental figure 6. Taqman RT-PCR detection of SLC45A2-AMACR in the blood samples of prostate cancer patients.

Supplemental figure 7. Taqman RT-PCR detection of TMEM135-CCDC67 in the blood samples of prostate cancer patients.

Supplemental figure 8. Taqman RT-PCR detection of LRRC59-FLJ60017 in the blood samples of prostate cancer patients.

Supplemental figure 9. Taqman RT-PCR detection of Pten-NOLC1 in the blood samples of prostate cancer patients.

Supplemental figure 10. Taqman RT-PCR detection of β-actin in the blood samples of prostate cancer patients.

Supplemental figure 11. Images of representative Sanger's sequencings of fusion transcripts from the blood samples of the prostate cancer patients. Twenty to 100% of blood samples positive for fusion genes KDM4B-AC011523.2, CCNH-c5orf30, mTOR-TP53BP1, MAN2A1-FER, SLC45A2-AMACR and Pten-NOLC1 were sequenced using Sanger's method. The fusion juncture regions were shown and indicated.

Supplemental figure 1

MAN2A1-FER fusion detection

MAN2A1-FER-PROSTATE-BLOOD SAMPLE 1-20

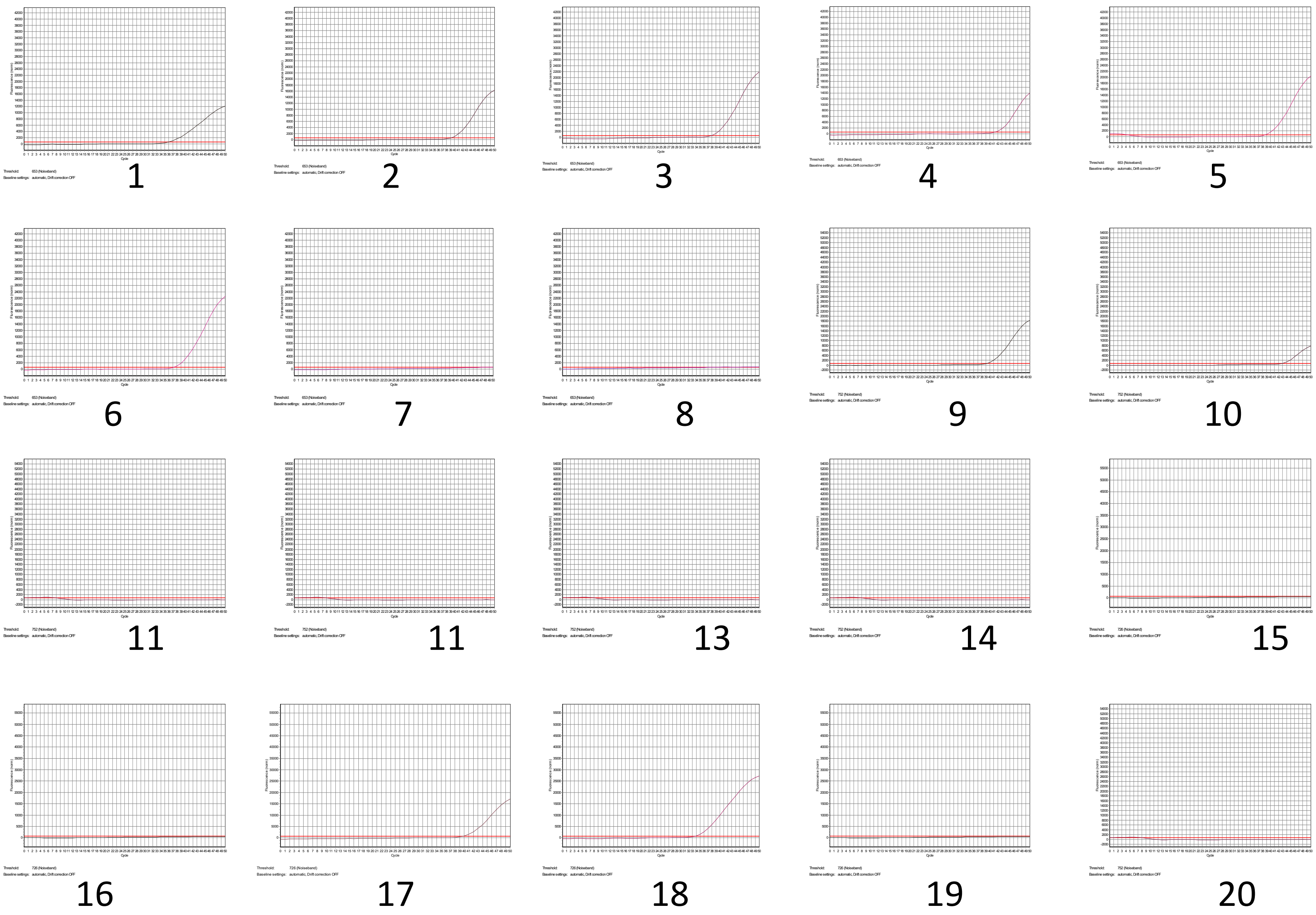

MAN2A1-FER-PROSTATE-BLOOD SAMPLE 21-40

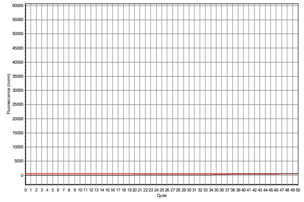

Threshold: 507 (Noiseband)
Baseline settings: automatic, Drift correction OFF

21

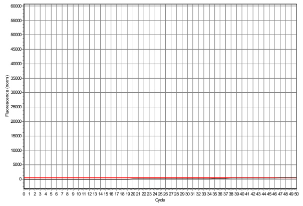

Threshold: 507 (Noiseband)
Baseline settings: automatic, Drift correction OFF

22

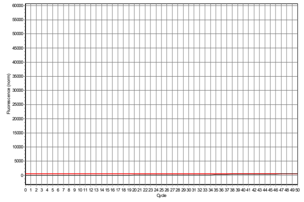

Threshold: 507 (Noiseband)
Baseline settings: automatic, Drift correction OFF

23

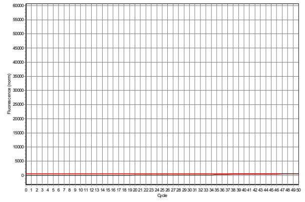

Threshold: 507 (Noiseband)
Baseline settings: automatic, Drift correction OFF

24

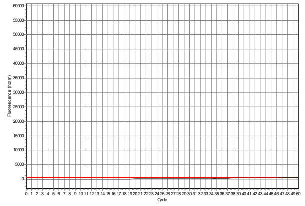

Threshold: 507 (Noiseband)
Baseline settings: automatic, Drift correction OFF

25

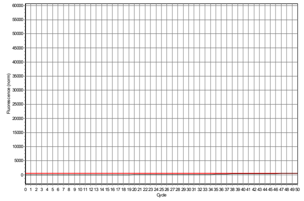

Threshold: 507 (Noiseband)
Baseline settings: automatic, Drift correction OFF

26

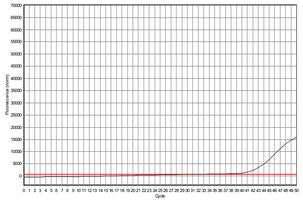

Threshold: 615 (Noiseband)
Baseline settings: automatic, Drift correction OFF

27

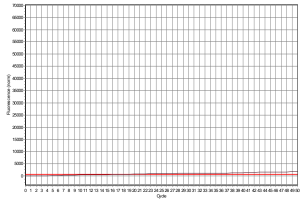

Threshold: 615 (Noiseband)
Baseline settings: automatic, Drift correction OFF

28

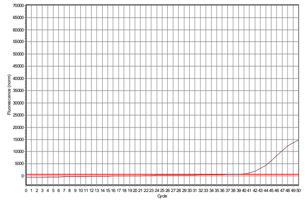

Threshold: 615 (Noiseband)
Baseline settings: automatic, Drift correction OFF

29

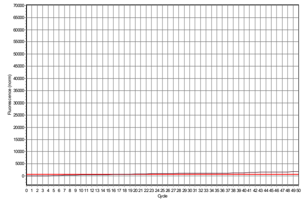

Threshold: 615 (Noiseband)
Baseline settings: automatic, Drift correction OFF

30

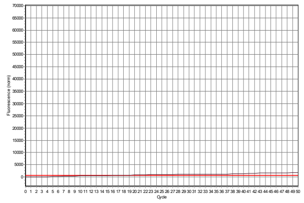

Threshold: 615 (Noiseband)
Baseline settings: automatic, Drift correction OFF

31

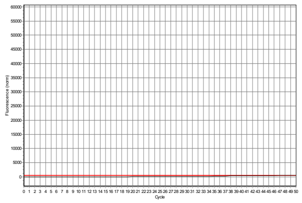

Threshold: 507 (Noiseband)
Baseline settings: automatic, Drift correction OFF

32

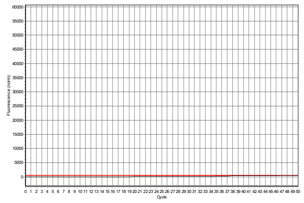

Threshold: 507 (Noiseband)
Baseline settings: automatic, Drift correction OFF

33

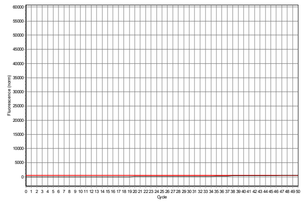

Threshold: 507 (Noiseband)
Baseline settings: automatic, Drift correction OFF

34

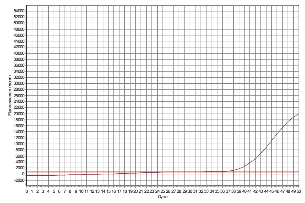

Threshold: 706 (Noiseband)
Baseline settings: automatic, Drift correction OFF

35

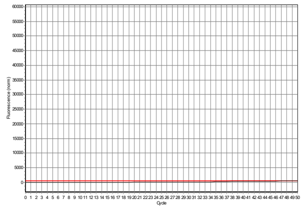

Threshold: 507 (Noiseband)
Baseline settings: automatic, Drift correction OFF

36

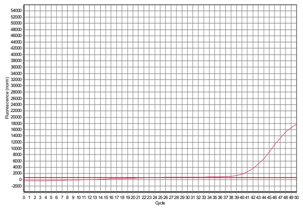

Threshold: 706 (Noiseband)
Baseline settings: automatic, Drift correction OFF

37

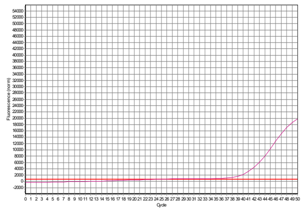

Threshold: 706 (Noiseband)
Baseline settings: automatic, Drift correction OFF

38

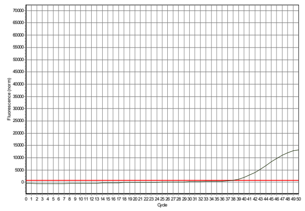

Threshold: 781 (Noiseband)
Baseline settings: automatic, Drift correction OFF

39

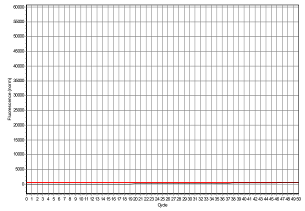

Threshold: 507 (Noiseband)
Baseline settings: automatic, Drift correction OFF

40

MAN2A1-FER-PROSTATE-BLOOD SAMPLE 41-60

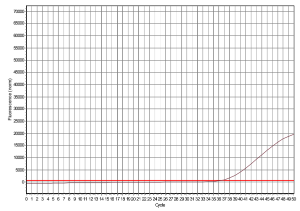
Threshold: 781 (Noiseband)
Baseline settings: automatic, Drift correction OFF

41

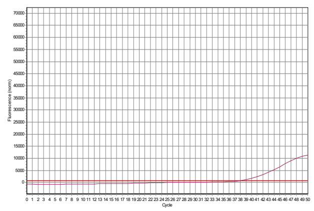
Threshold: 781 (Noiseband)
Baseline settings: automatic, Drift correction OFF

42

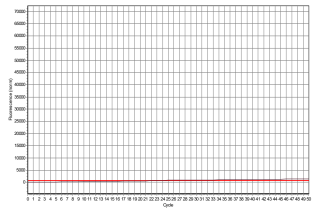
Threshold: 781 (Noiseband)
Baseline settings: automatic, Drift correction OFF

43

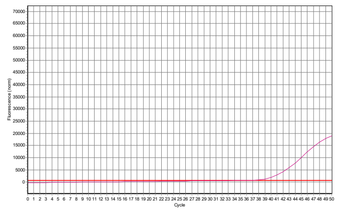
Threshold: 781 (Noiseband)
Baseline settings: automatic, Drift correction OFF

44

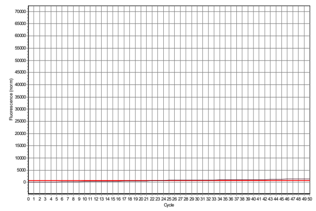
Threshold: 781 (Noiseband)
Baseline settings: automatic, Drift correction OFF

45

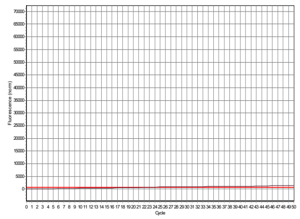
Threshold: 781 (Noiseband)
Baseline settings: automatic, Drift correction OFF

46

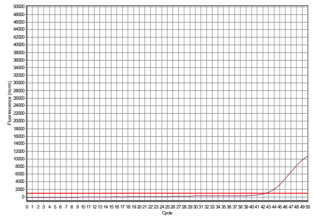
Threshold: 1016 (Noiseband)
Baseline settings: automatic, Drift correction OFF

47

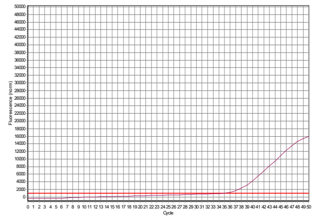
Threshold: 1016 (Noiseband)
Baseline settings: automatic, Drift correction OFF

47

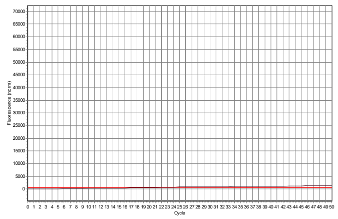
Threshold: 781 (Noiseband)
Baseline settings: automatic, Drift correction OFF

49

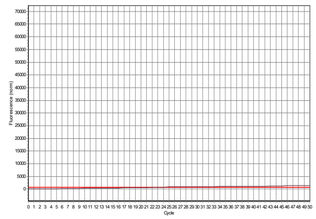
Threshold: 781 (Noiseband)
Baseline settings: automatic, Drift correction OFF

50

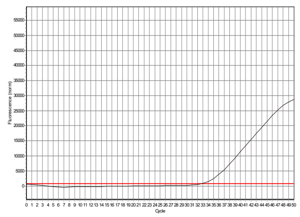
Threshold: 763 (Noiseband)
Baseline settings: automatic, Drift correction OFF

51

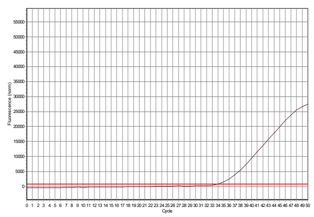
Threshold: 763 (Noiseband)
Baseline settings: automatic, Drift correction OFF

52

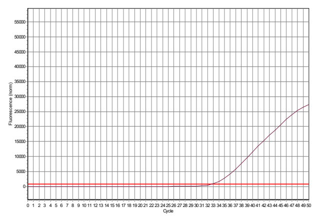
Threshold: 763 (Noiseband)
Baseline settings: automatic, Drift correction OFF

53

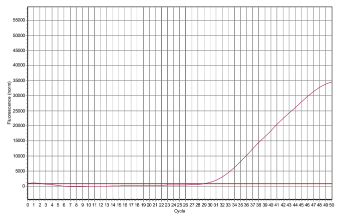
Threshold: 763 (Noiseband)
Baseline settings: automatic, Drift correction OFF

54

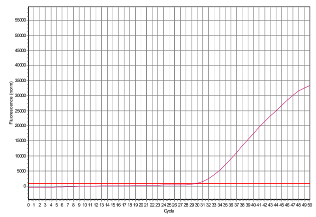
Threshold: 763 (Noiseband)
Baseline settings: automatic, Drift correction OFF

55

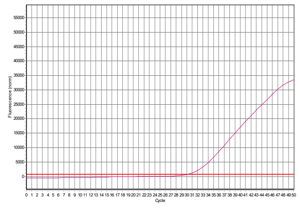
Threshold: 763 (Noiseband)
Baseline settings: automatic, Drift correction OFF

56

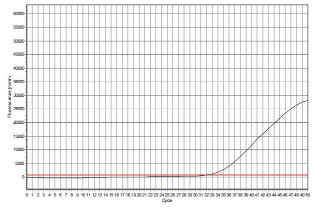
Threshold: 759 (Noiseband)
Baseline settings: automatic, Drift correction OFF

57

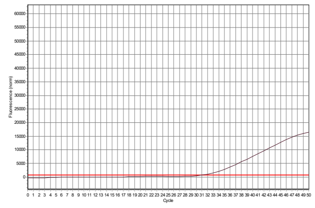
Threshold: 759 (Noiseband)
Baseline settings: automatic, Drift correction OFF

58

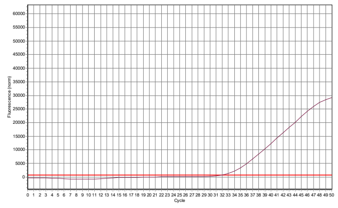
Threshold: 759 (Noiseband)
Baseline settings: automatic, Drift correction OFF

59

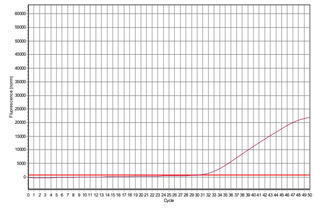
Threshold: 759 (Noiseband)
Baseline settings: automatic, Drift correction OFF

60

MAN2A1-FER-PROSTATE-BLOOD SAMPLE 61-80

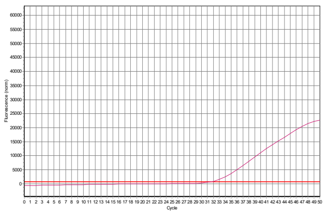
Threshold: 759 (Noiseband)
Baseline settings: automatic, Drift correction OFF
61

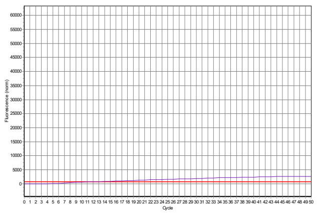
Threshold: 759 (Noiseband)
Baseline settings: automatic, Drift correction OFF
62

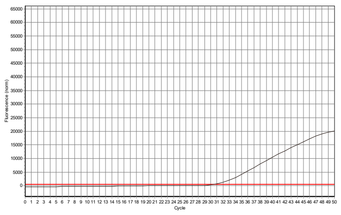
Threshold: 516 (Noiseband)
Baseline settings: automatic, Drift correction OFF
63

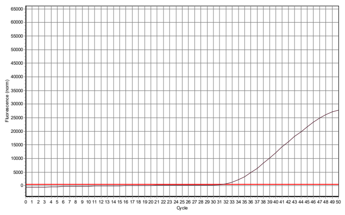
Threshold: 516 (Noiseband)
Baseline settings: automatic, Drift correction OFF
64

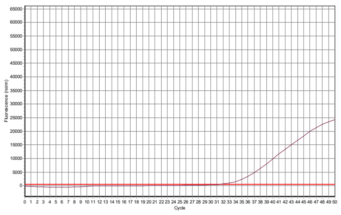
Threshold: 516 (Noiseband)
Baseline settings: automatic, Drift correction OFF
65

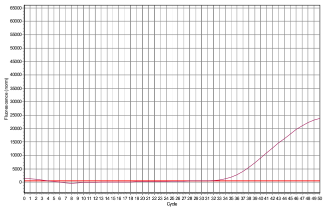
Threshold: 516 (Noiseband)
Baseline settings: automatic, Drift correction OFF
66

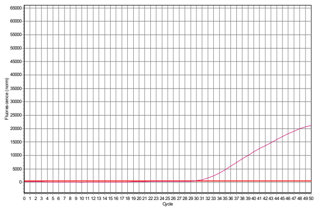
Threshold: 516 (Noiseband)
Baseline settings: automatic, Drift correction OFF
67

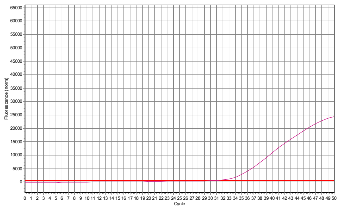
Threshold: 516 (Noiseband)
Baseline settings: automatic, Drift correction OFF
68

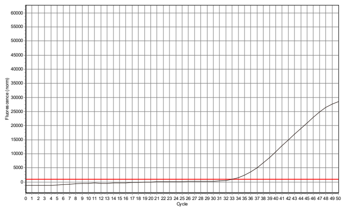
Threshold: 1024 (Noiseband)
Baseline settings: automatic, Drift correction OFF
69

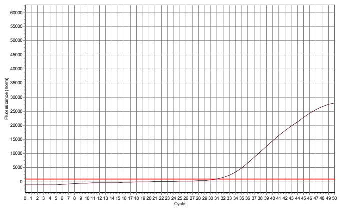
Threshold: 1024 (Noiseband)
Baseline settings: automatic, Drift correction OFF
70

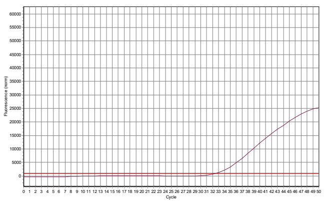
Threshold: 1024 (Noiseband)
Baseline settings: automatic, Drift correction OFF
71

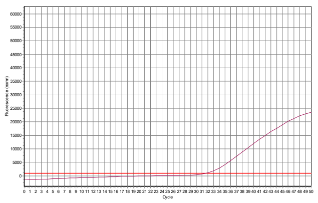
Threshold: 1024 (Noiseband)
Baseline settings: automatic, Drift correction OFF
72

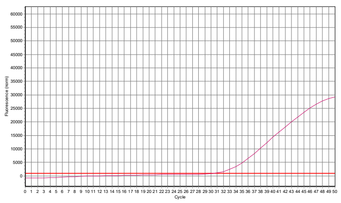
Threshold: 1024 (Noiseband)
Baseline settings: automatic, Drift correction OFF
73

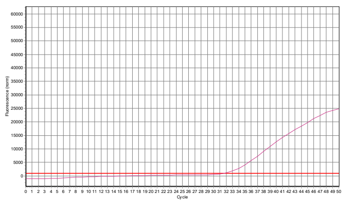
Threshold: 1024 (Noiseband)
Baseline settings: automatic, Drift correction OFF
74

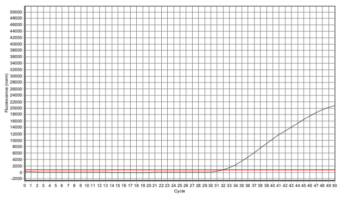
Threshold: 741 (Noiseband)
Baseline settings: automatic, Drift correction OFF
75

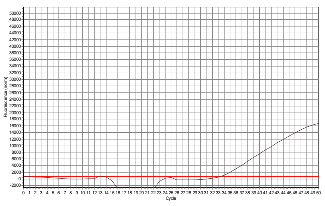
Threshold: 741 (Noiseband)
Baseline settings: automatic, Drift correction OFF
76

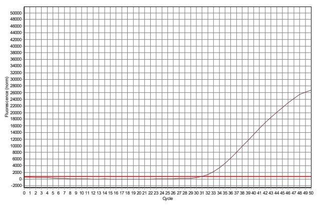
Threshold: 741 (Noiseband)
Baseline settings: automatic, Drift correction OFF
77

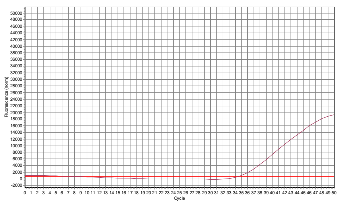
Threshold: 741 (Noiseband)
Baseline settings: automatic, Drift correction OFF
78

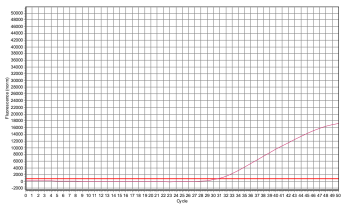
Threshold: 741 (Noiseband)
Baseline settings: automatic, Drift correction OFF
79

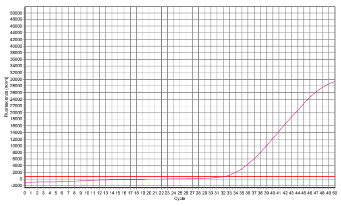
Threshold: 741 (Noiseband)
Baseline settings: automatic, Drift correction OFF
80

MAN2A1-FER-PROSTATE-BLOOD SAMPLE 81-100

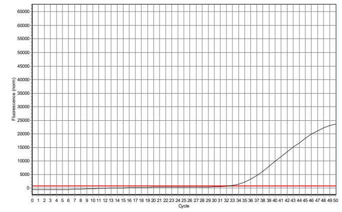
81

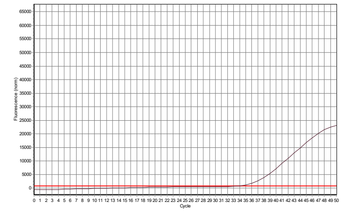
82

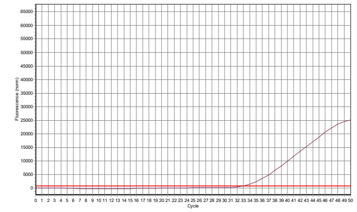
83

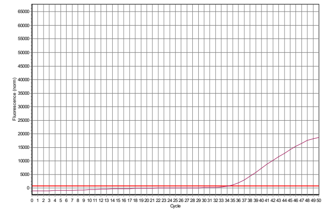
84

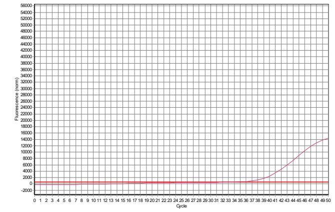
85

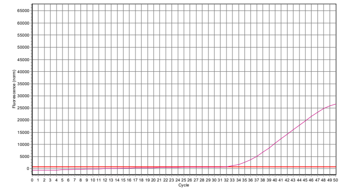
86

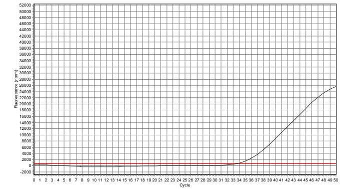
87

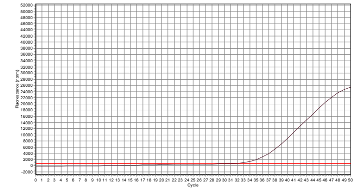
88

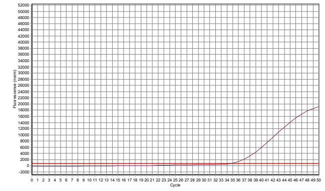
89

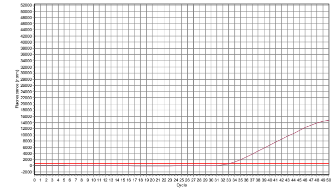
90

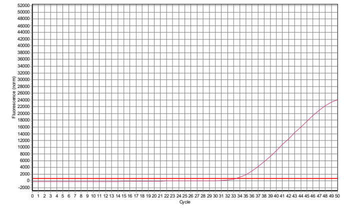
91

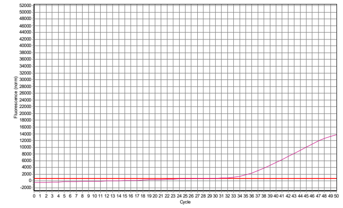
92

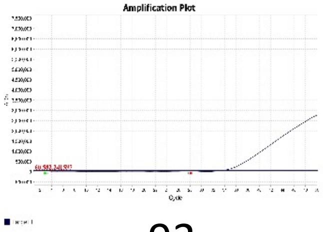

93

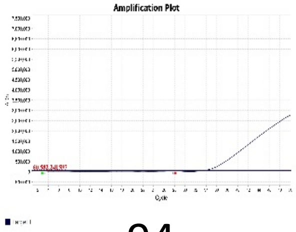

94

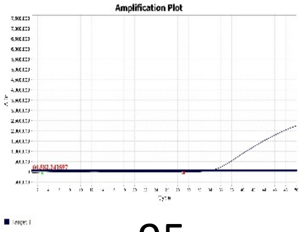

95

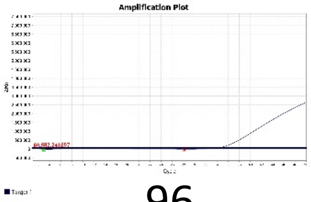

96

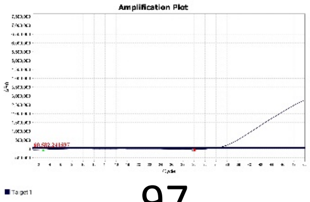

97

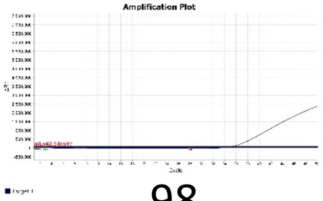

98

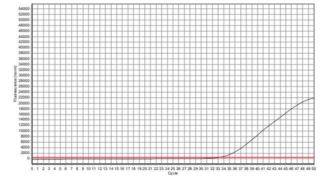
99

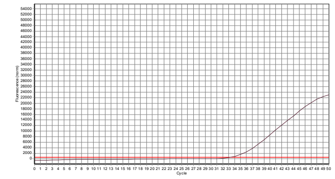
100

MAN2A1-FER-PROSTATE-BLOOD SAMPLE 101-120

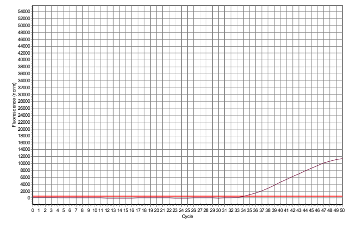
101

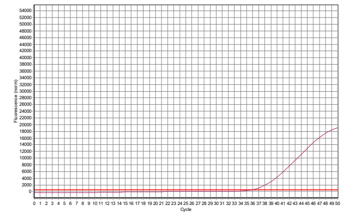
102

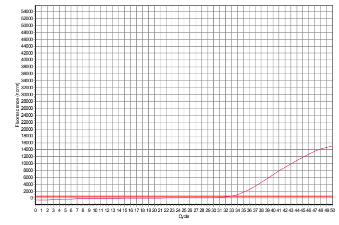
103

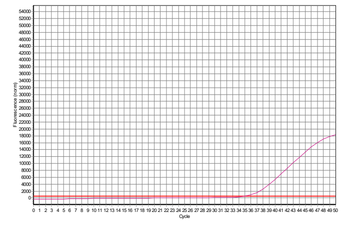
104

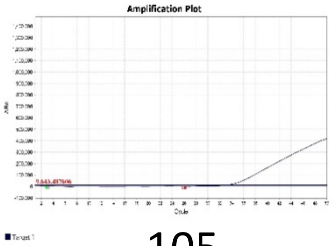
105

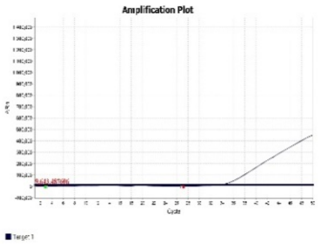
106

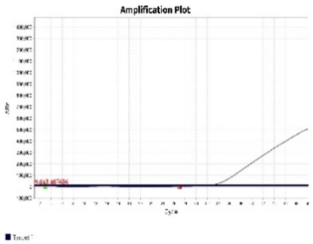
107

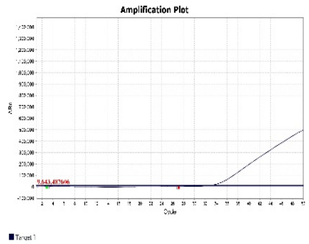
108

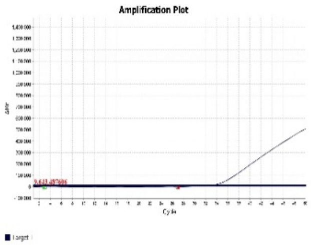
109

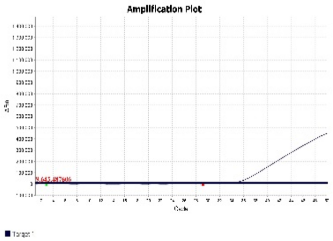
110

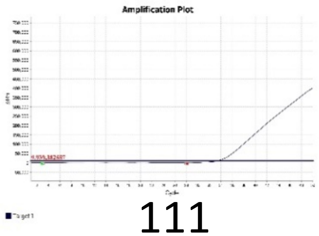
111

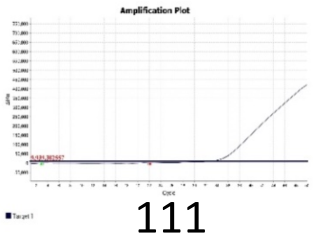
111

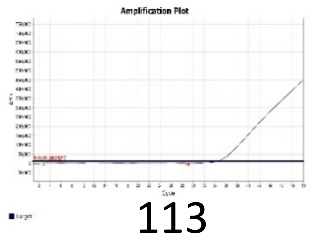
113

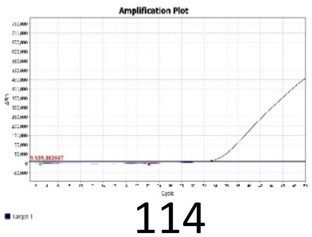
114

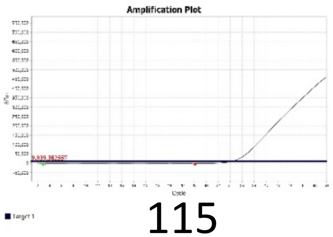
115

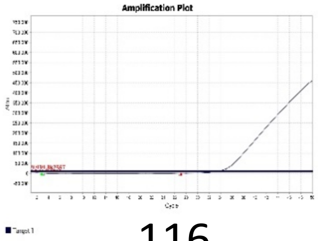
116

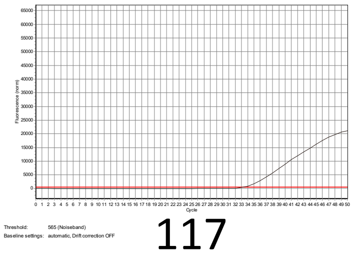
117

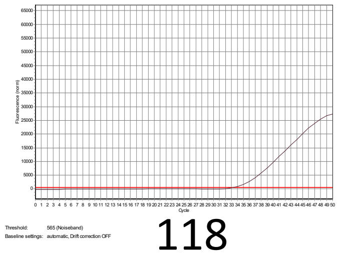
118

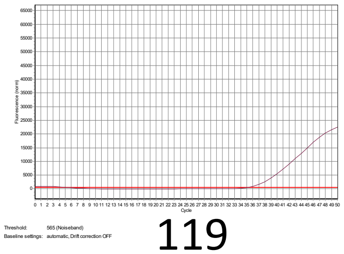
119

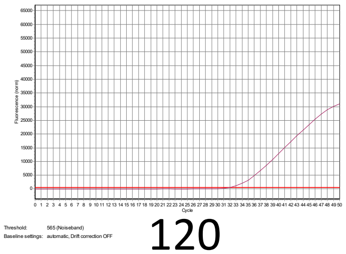
120

MAN2A1-FER-PROSTATE-BLOOD SAMPLE 121-140

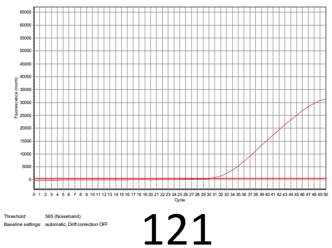
121

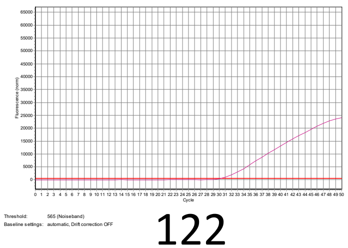
122

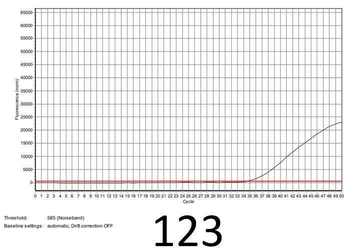
123

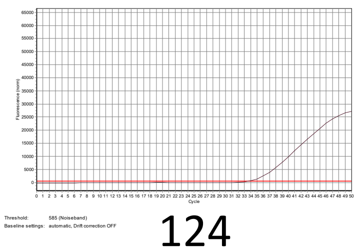
124

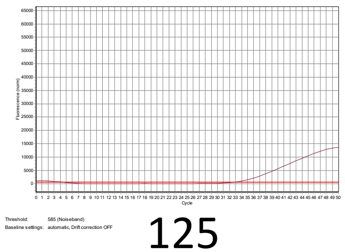
125

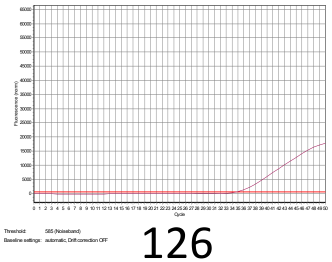
126

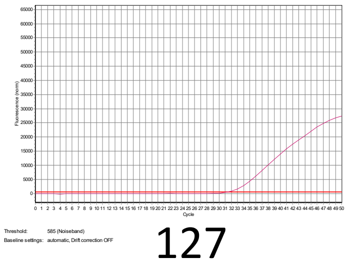
127

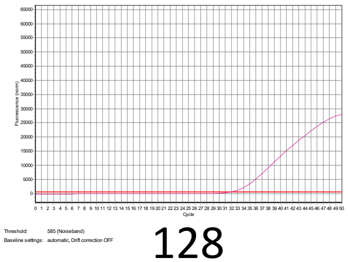
128

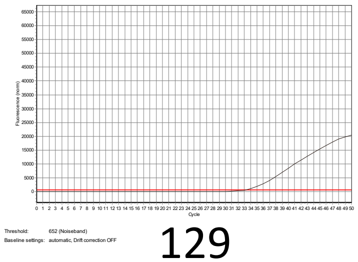
129

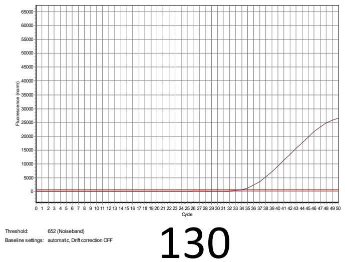
130

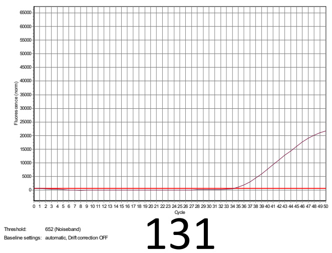
131

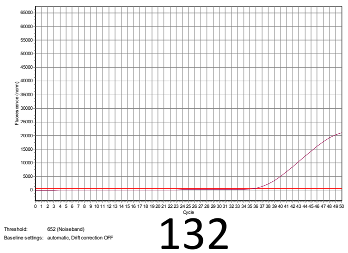
132

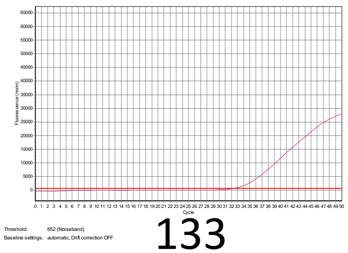
133

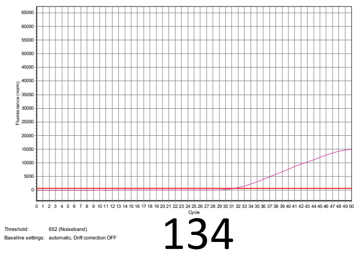
134

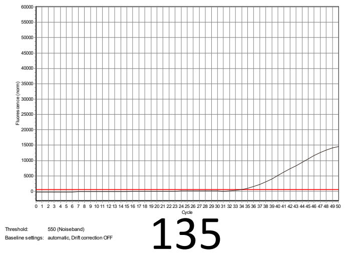
135

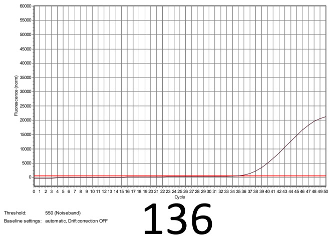
136

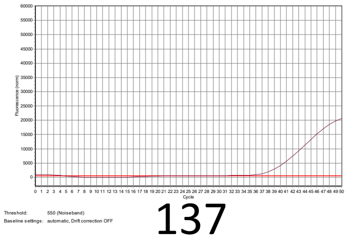
137

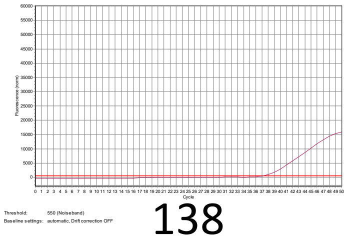
138

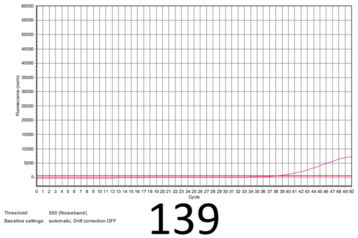
139

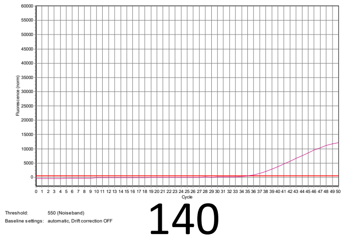
140

MAN2A1-FER-PROSTATE-BLOOD SAMPLE 141-150

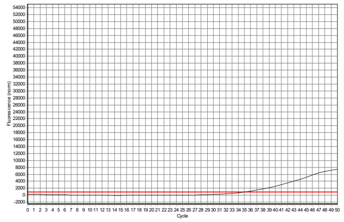

Threshold: 896 (Adjusted manually)
Baseline settings: automatic, Drift correction OFF

141

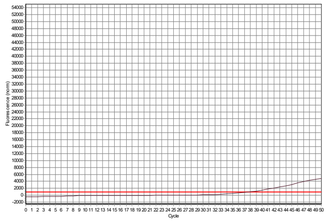

Threshold: 896 (Adjusted manually)
Baseline settings: automatic, Drift correction OFF

142

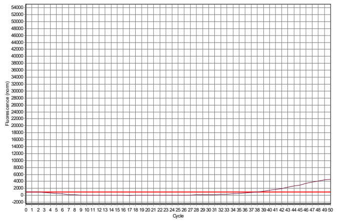

Threshold: 896 (Adjusted manually)
Baseline settings: automatic, Drift correction OFF

143

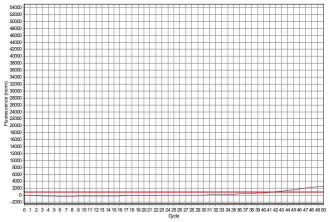

Threshold: 896 (Adjusted manually)
Baseline settings: automatic, Drift correction OFF

144

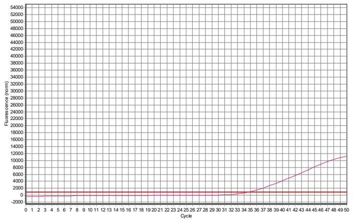

Threshold: 896 (Adjusted manually)
Baseline settings: automatic, Drift correction OFF

145

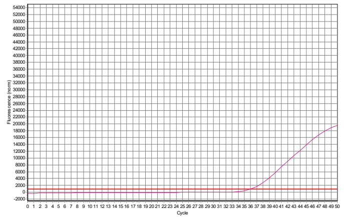

Threshold: 896 (Adjusted manually)
Baseline settings: automatic, Drift correction OFF

146

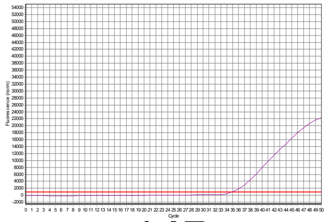

Threshold: 896 (Adjusted manually)
Baseline settings: automatic, Drift correction OFF

147

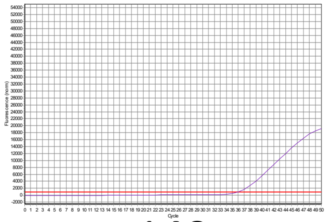

Threshold: 896 (Adjusted manually)
Baseline settings: automatic, Drift correction OFF

148

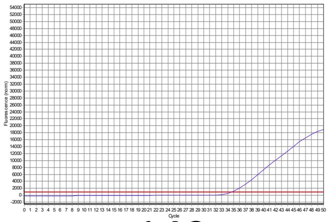

Threshold: 896 (Adjusted manually)
Baseline settings: automatic, Drift correction OFF

149

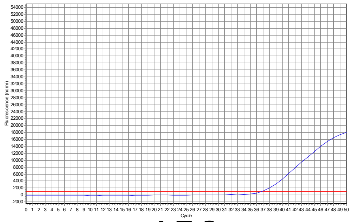

Threshold: 896 (Adjusted manually)
Baseline settings: automatic, Drift correction OFF

150

Supplemental figure 2 TRMT11-TRIK2 fusion detection

TRMT11-GRIK2-PROSTATE-BLOOD SAMPLE 1-20

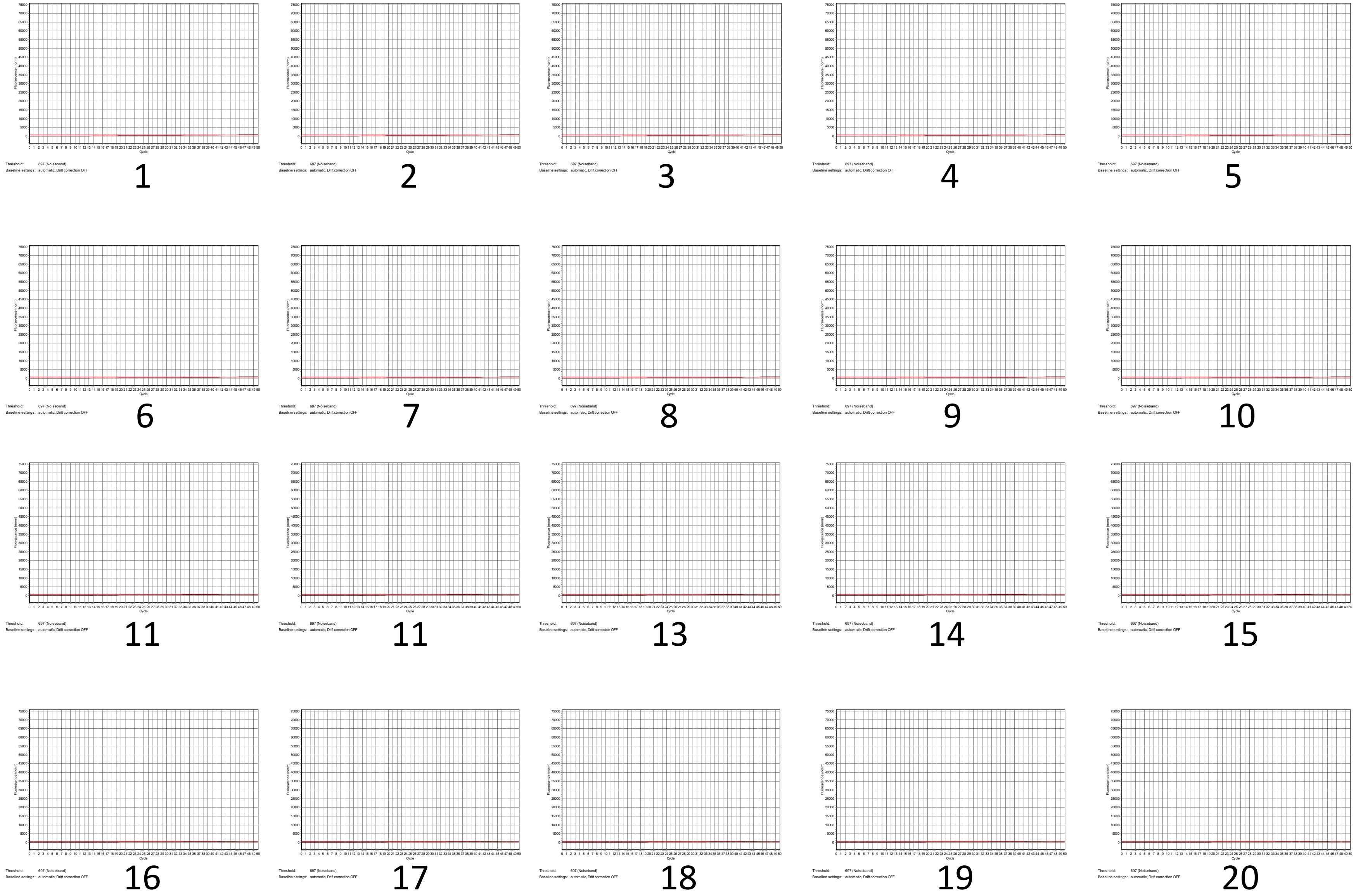

TRMT11-GRIK2-PROSTATE-BLOOD SAMPLE 21-40

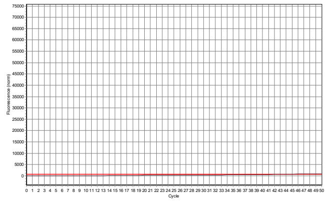

Threshold: 697 (Noiseband)
Baseline settings: automatic, Drift correction OFF

21

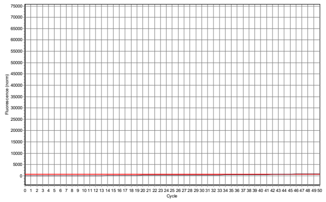

Threshold: 697 (Noiseband)
Baseline settings: automatic, Drift correction OFF

22

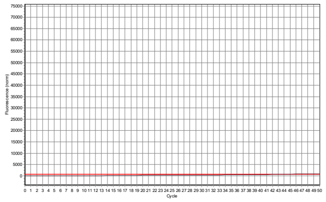

Threshold: 697 (Noiseband)
Baseline settings: automatic, Drift correction OFF

23

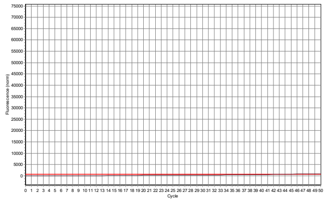

Threshold: 697 (Noiseband)
Baseline settings: automatic, Drift correction OFF

24

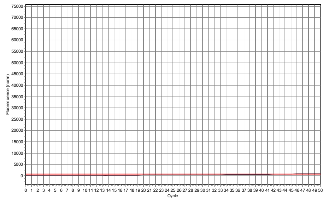

Threshold: 697 (Noiseband)
Baseline settings: automatic, Drift correction OFF

25

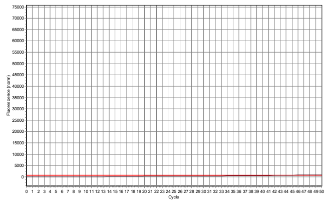

Threshold: 697 (Noiseband)
Baseline settings: automatic, Drift correction OFF

26

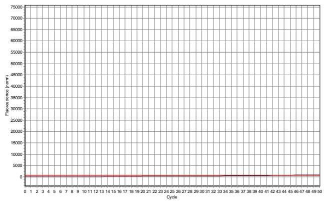

Threshold: 697 (Noiseband)
Baseline settings: automatic, Drift correction OFF

27

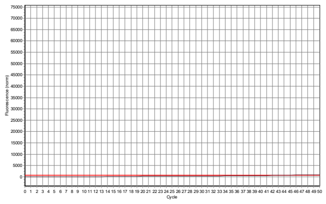

Threshold: 697 (Noiseband)
Baseline settings: automatic, Drift correction OFF

28

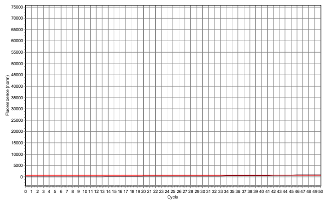

Threshold: 697 (Noiseband)
Baseline settings: automatic, Drift correction OFF

29

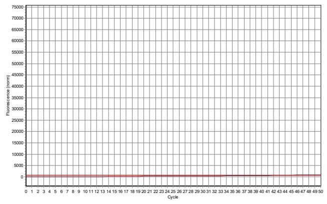

Threshold: 697 (Noiseband)
Baseline settings: automatic, Drift correction OFF

30

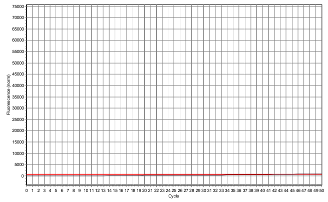

Threshold: 697 (Noiseband)
Baseline settings: automatic, Drift correction OFF

31

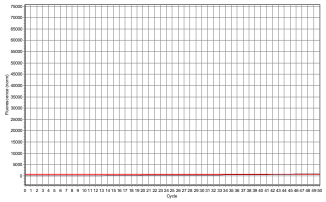

Threshold: 697 (Noiseband)
Baseline settings: automatic, Drift correction OFF

32

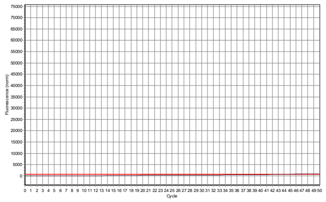

Threshold: 697 (Noiseband)
Baseline settings: automatic, Drift correction OFF

33

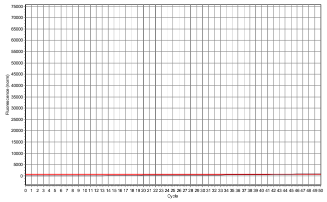

Threshold: 697 (Noiseband)
Baseline settings: automatic, Drift correction OFF

34

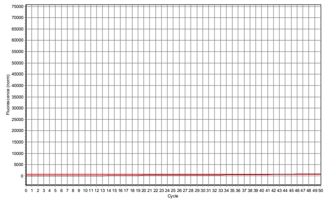

Threshold: 697 (Noiseband)
Baseline settings: automatic, Drift correction OFF

35

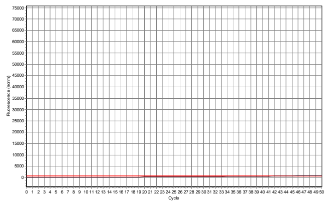

Threshold: 697 (Noiseband)
Baseline settings: automatic, Drift correction OFF

36

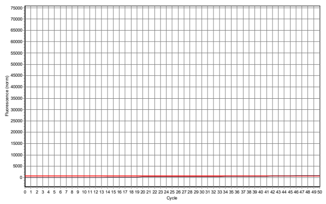

Threshold: 697 (Noiseband)
Baseline settings: automatic, Drift correction OFF

37

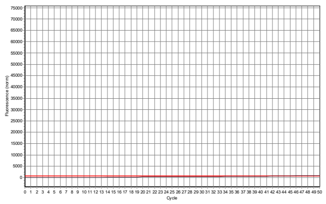

Threshold: 697 (Noiseband)
Baseline settings: automatic, Drift correction OFF

38

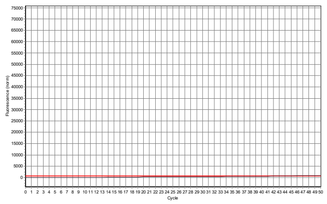

Threshold: 697 (Noiseband)
Baseline settings: automatic, Drift correction OFF

39

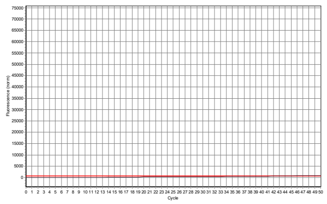

Threshold: 697 (Noiseband)
Baseline settings: automatic, Drift correction OFF

40

TRMT11-GRIK2-PROSTATE-BLOOD SAMPLE 41-60

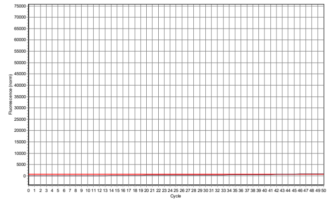

Threshold: 697 (Noiseband)
Baseline settings: automatic, Drift correction OFF

41

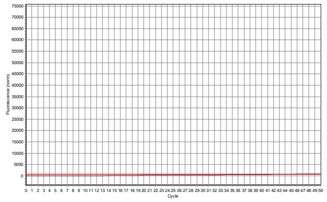

Threshold: 697 (Noiseband)
Baseline settings: automatic, Drift correction OFF

42

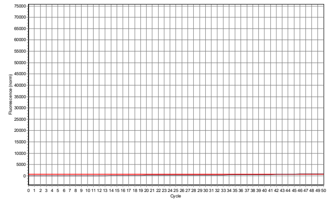

Threshold: 697 (Noiseband)
Baseline settings: automatic, Drift correction OFF

43

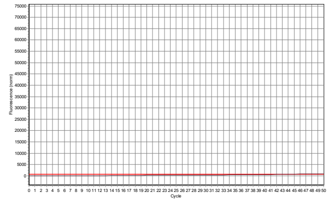

Threshold: 697 (Noiseband)
Baseline settings: automatic, Drift correction OFF

44

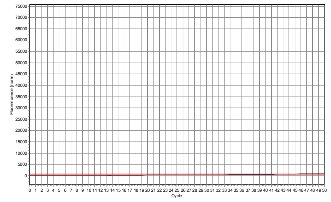

Threshold: 697 (Noiseband)
Baseline settings: automatic, Drift correction OFF

45

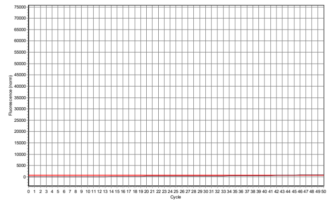

Threshold: 697 (Noiseband)
Baseline settings: automatic, Drift correction OFF

46

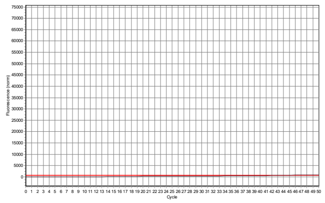

Threshold: 697 (Noiseband)
Baseline settings: automatic, Drift correction OFF

47

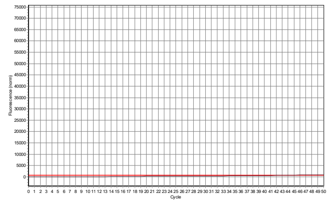

Threshold: 697 (Noiseband)
Baseline settings: automatic, Drift correction OFF

47

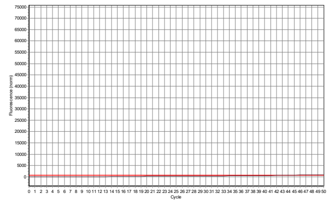

Threshold: 697 (Noiseband)
Baseline settings: automatic, Drift correction OFF

49

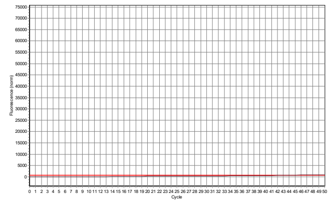

Threshold: 697 (Noiseband)
Baseline settings: automatic, Drift correction OFF

50

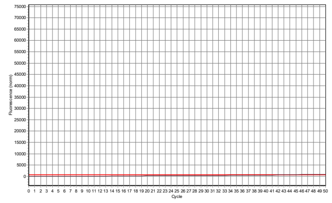

Threshold: 697 (Noiseband)
Baseline settings: automatic, Drift correction OFF

51

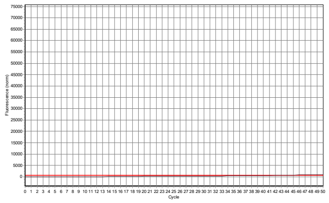

Threshold: 697 (Noiseband)
Baseline settings: automatic, Drift correction OFF

52

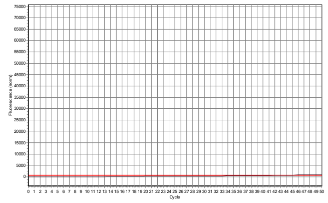

Threshold: 697 (Noiseband)
Baseline settings: automatic, Drift correction OFF

53

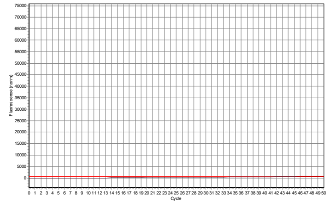

Threshold: 697 (Noiseband)
Baseline settings: automatic, Drift correction OFF

54

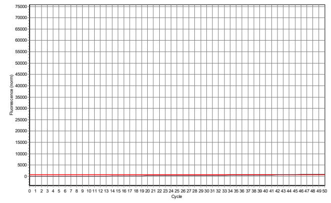

Threshold: 697 (Noiseband)
Baseline settings: automatic, Drift correction OFF

55

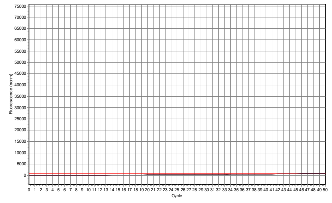

Threshold: 697 (Noiseband)
Baseline settings: automatic, Drift correction OFF

56

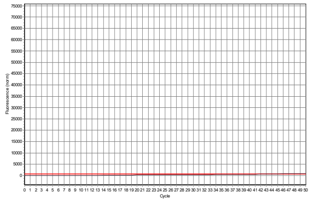

Threshold: 697 (Noiseband)
Baseline settings: automatic, Drift correction OFF

57

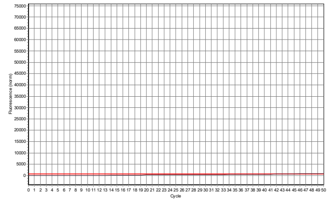

Threshold: 697 (Noiseband)
Baseline settings: automatic, Drift correction OFF

58

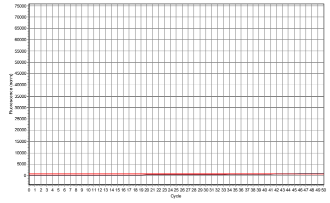

Threshold: 697 (Noiseband)
Baseline settings: automatic, Drift correction OFF

59

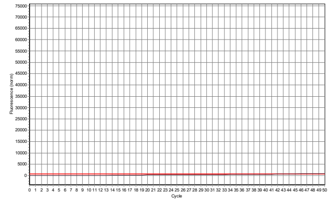

Threshold: 697 (Noiseband)
Baseline settings: automatic, Drift correction OFF

60

TRMT11-GRIK2-PROSTATE-BLOOD SAMPLE 61-80

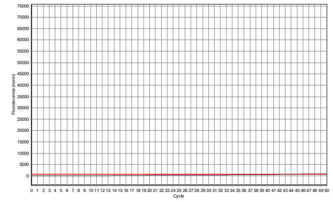
Threshold: 697 (Noiseband)
Baseline settings: automatic, Drift correction OFF

61

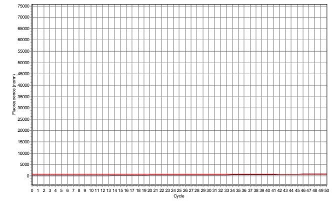
Threshold: 697 (Noiseband)
Baseline settings: automatic, Drift correction OFF

62

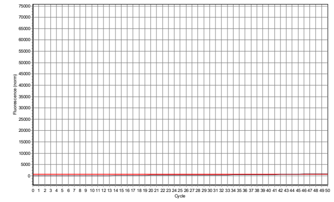
Threshold: 697 (Noiseband)
Baseline settings: automatic, Drift correction OFF

63

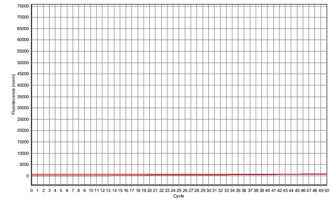
Threshold: 697 (Noiseband)
Baseline settings: automatic, Drift correction OFF

64

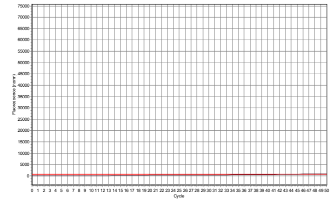
Threshold: 697 (Noiseband)
Baseline settings: automatic, Drift correction OFF

65

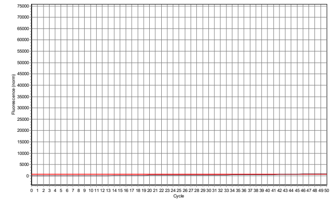
Threshold: 697 (Noiseband)
Baseline settings: automatic, Drift correction OFF

66

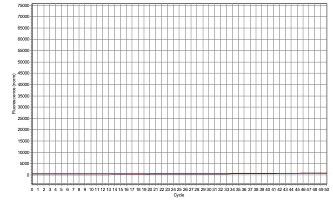
Threshold: 697 (Noiseband)
Baseline settings: automatic, Drift correction OFF

67

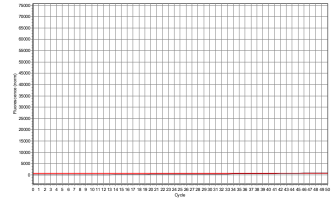
Threshold: 697 (Noiseband)
Baseline settings: automatic, Drift correction OFF

68

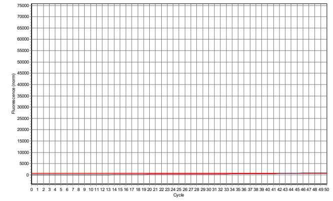
Threshold: 697 (Noiseband)
Baseline settings: automatic, Drift correction OFF

69

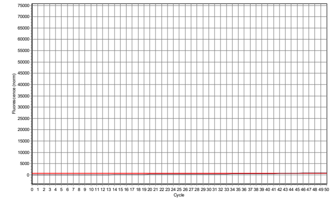
Threshold: 697 (Noiseband)
Baseline settings: automatic, Drift correction OFF

70

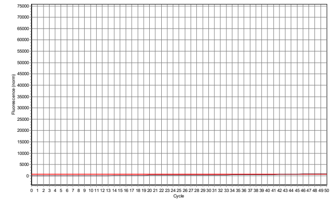
Threshold: 697 (Noiseband)
Baseline settings: automatic, Drift correction OFF

71

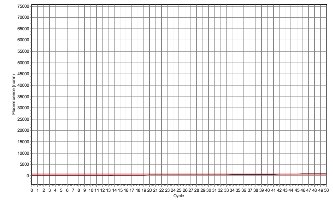
Threshold: 697 (Noiseband)
Baseline settings: automatic, Drift correction OFF

72

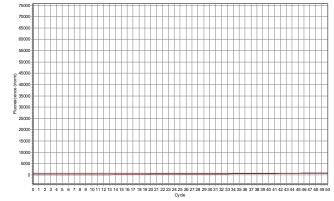
Threshold: 697 (Noiseband)
Baseline settings: automatic, Drift correction OFF

73

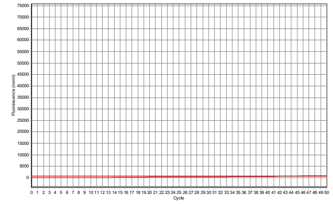
Threshold: 697 (Noiseband)
Baseline settings: automatic, Drift correction OFF

74

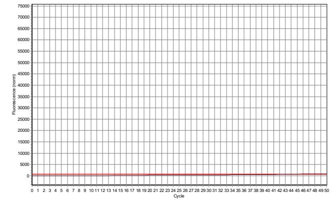
Threshold: 697 (Noiseband)
Baseline settings: automatic, Drift correction OFF

75

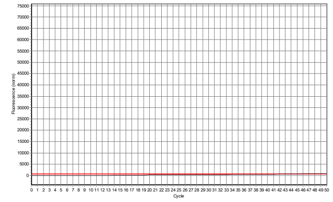
Threshold: 697 (Noiseband)
Baseline settings: automatic, Drift correction OFF

76

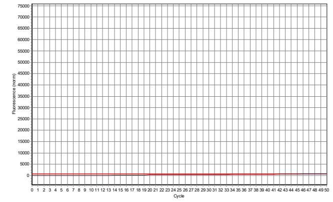
Threshold: 697 (Noiseband)
Baseline settings: automatic, Drift correction OFF

77

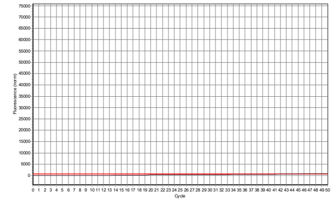
Threshold: 697 (Noiseband)
Baseline settings: automatic, Drift correction OFF

78

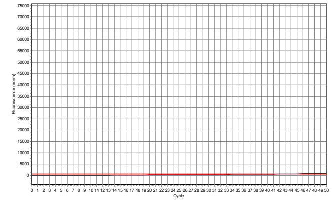
Threshold: 697 (Noiseband)
Baseline settings: automatic, Drift correction OFF

79

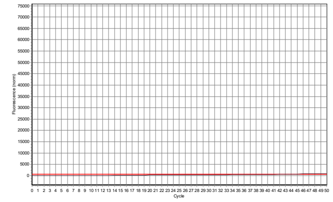
Threshold: 697 (Noiseband)
Baseline settings: automatic, Drift correction OFF

80

TRMT11-GRIK2-PROSTATE-BLOOD SAMPLE 81-100

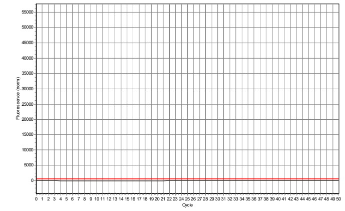
Threshold: 487 (Noiseband)
Baseline settings: automatic, Drift correction OFF

81

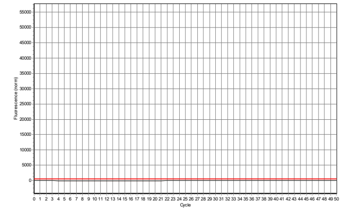
Threshold: 487 (Noiseband)
Baseline settings: automatic, Drift correction OFF

82

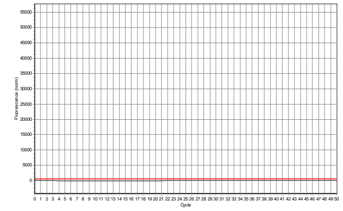
Threshold: 487 (Noiseband)
Baseline settings: automatic, Drift correction OFF

83

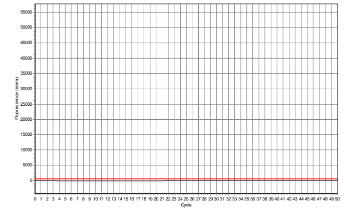
Threshold: 487 (Noiseband)
Baseline settings: automatic, Drift correction OFF

84

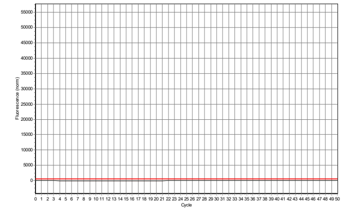
Threshold: 487 (Noiseband)
Baseline settings: automatic, Drift correction OFF

85

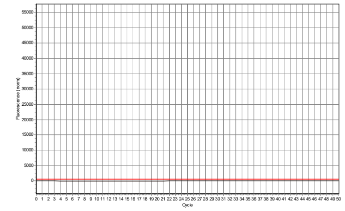
Threshold: 487 (Noiseband)
Baseline settings: automatic, Drift correction OFF

86

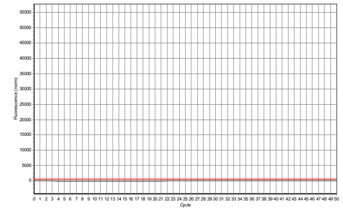
Threshold: 487 (Noiseband)
Baseline settings: automatic, Drift correction OFF

87

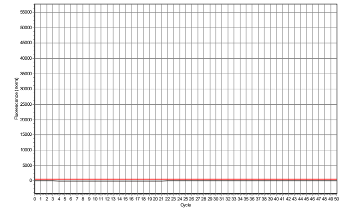
Threshold: 487 (Noiseband)
Baseline settings: automatic, Drift correction OFF

88

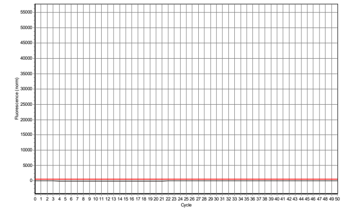
Threshold: 487 (Noiseband)
Baseline settings: automatic, Drift correction OFF

89

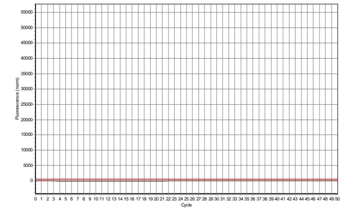
Threshold: 487 (Noiseband)
Baseline settings: automatic, Drift correction OFF

90

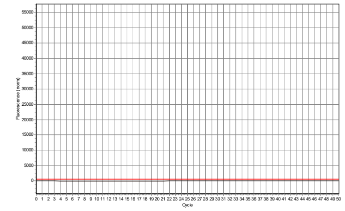
Threshold: 487 (Noiseband)
Baseline settings: automatic, Drift correction OFF

91

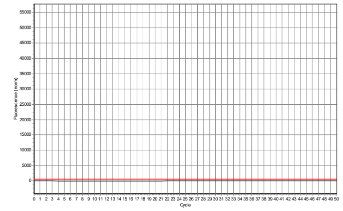
Threshold: 487 (Noiseband)
Baseline settings: automatic, Drift correction OFF

92

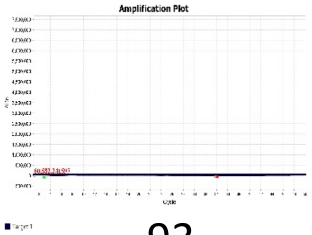

93

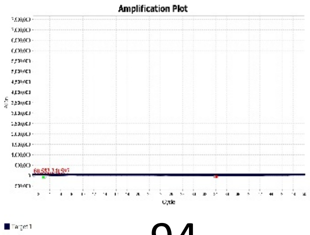

94

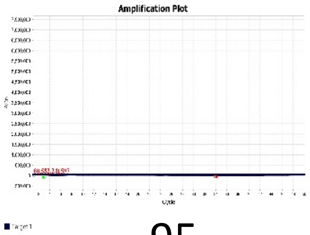

95

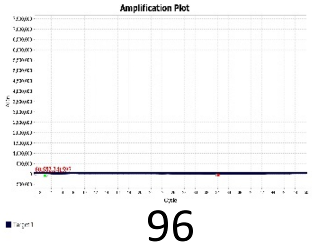

96

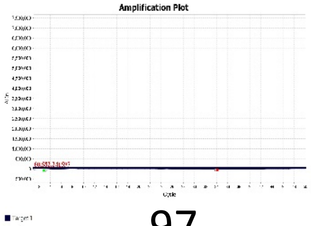

97

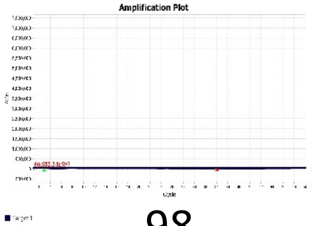

98

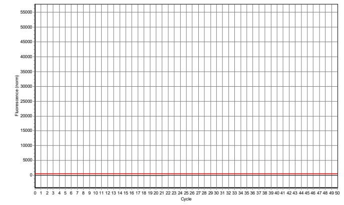
Threshold: 487 (Noiseband)
Baseline settings: automatic, Drift correction OFF

99

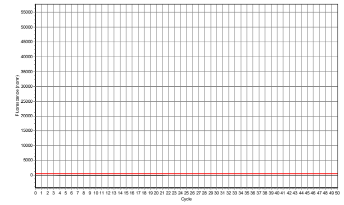
Threshold: 487 (Noiseband)
Baseline settings: automatic, Drift correction OFF

100

TRMT11-GRIK2-PROSTATE-BLOOD SAMPLE 101-120

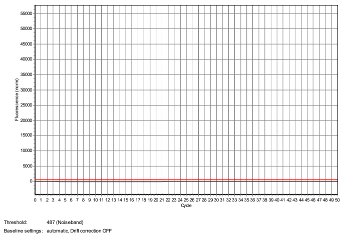

101

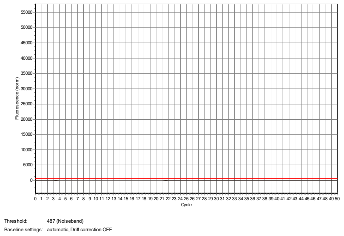

102

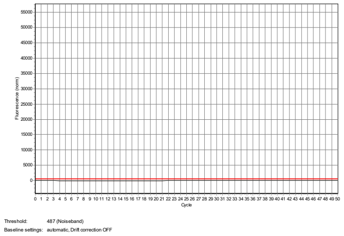

103

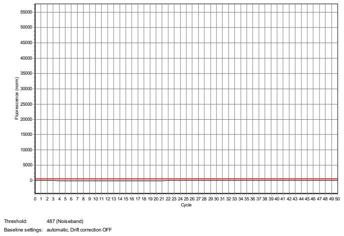

104

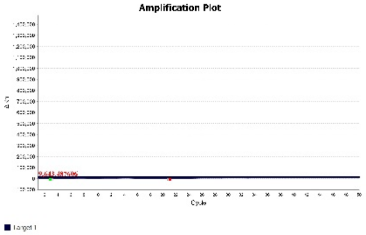

105

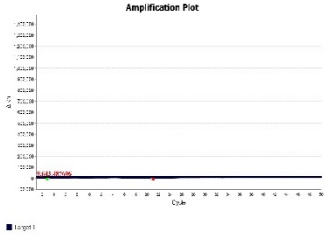

106

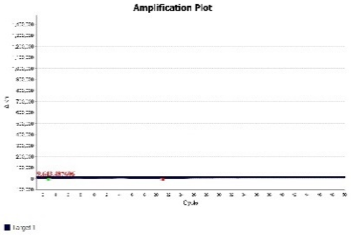

107

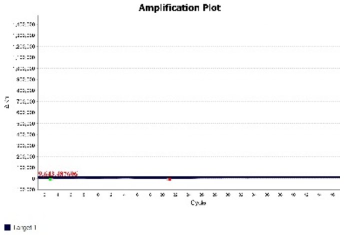

108

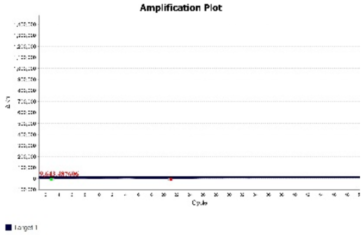

109

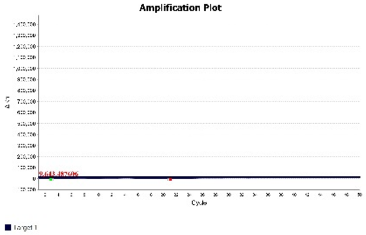

110

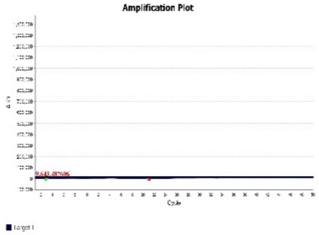

111

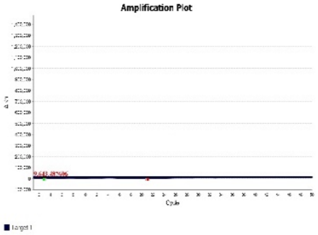

111

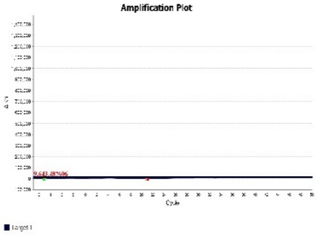

113

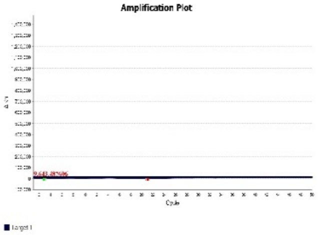

114

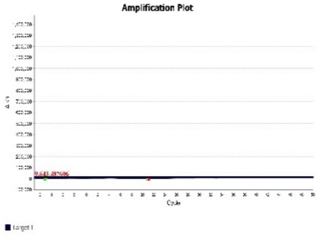

115

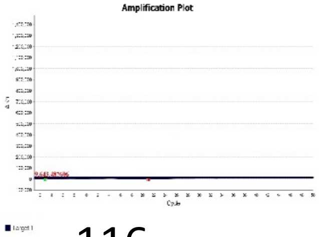

116

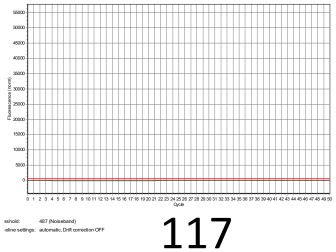

117

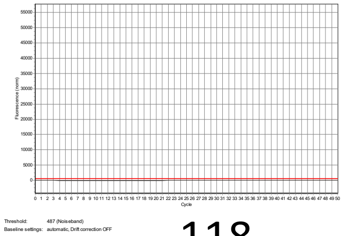

118

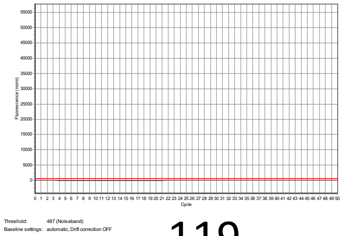

119

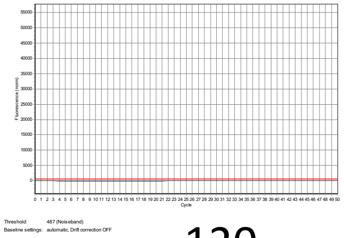

120

TRMT11-GRIK2-PROSTATE-BLOOD SAMPLE 121-140

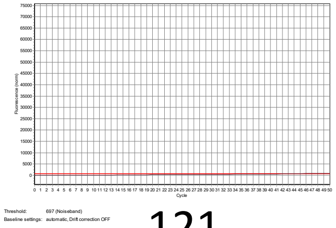

121

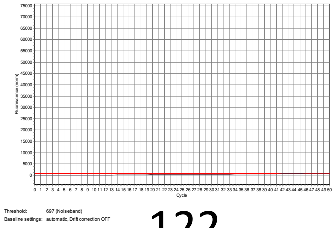

122

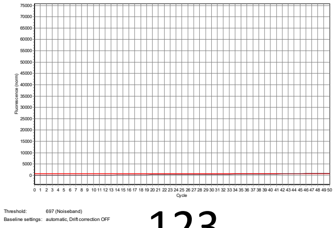

123

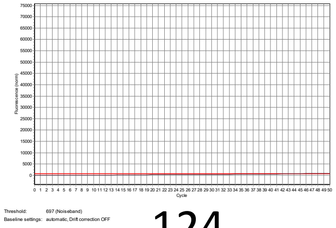

124

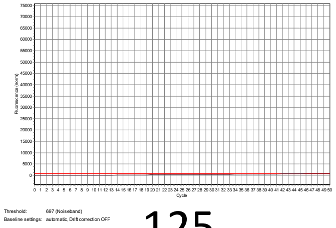

125

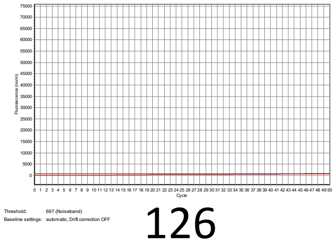

126

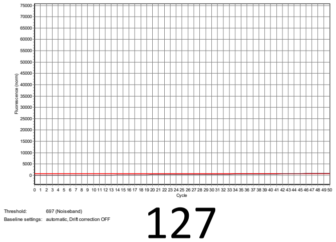

127

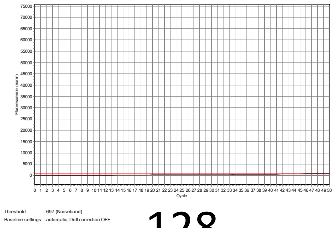

128

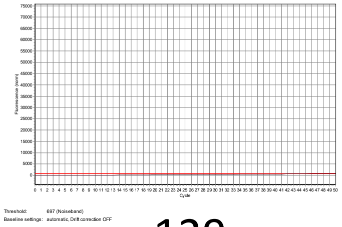

129

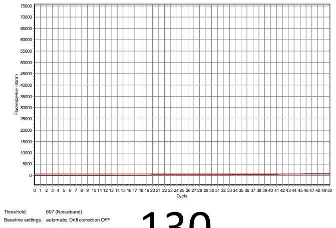

130

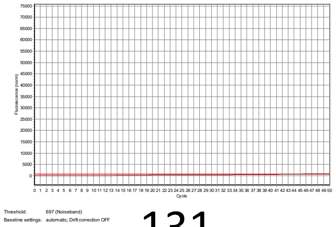

131

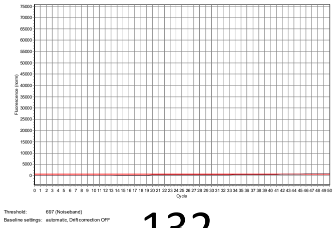

132

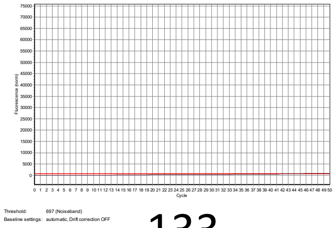

133

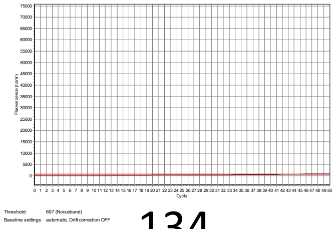

134

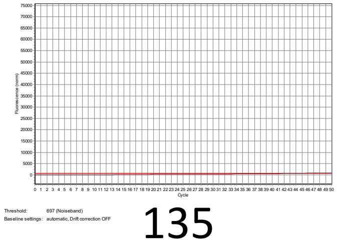

135

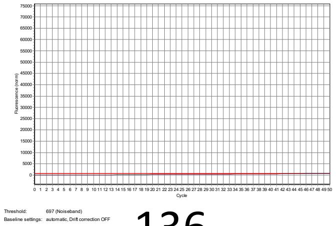

136

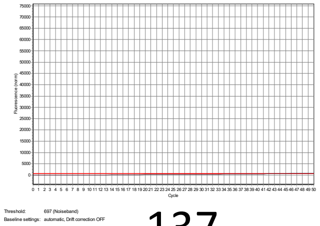

137

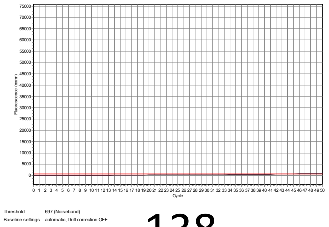

138

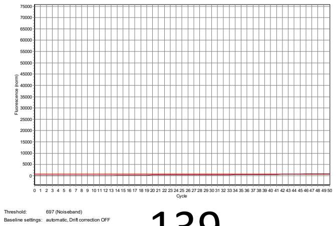

139

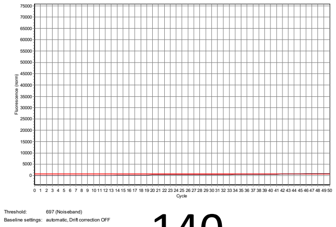

140

TRMT11-GRIK2-PROSTATE-BLOOD SAMPLE 141-150

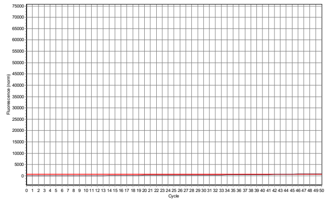

Threshold: 697 (Noiseband)
Baseline settings: automatic, Drift correction OFF

141

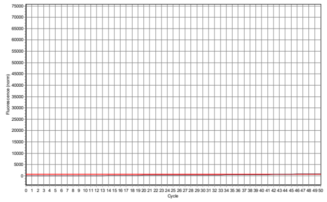

Threshold: 697 (Noiseband)
Baseline settings: automatic, Drift correction OFF

142

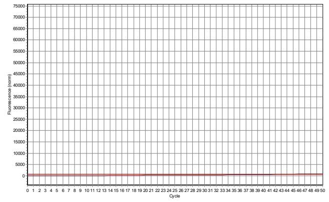

Threshold: 697 (Noiseband)
Baseline settings: automatic, Drift correction OFF

143

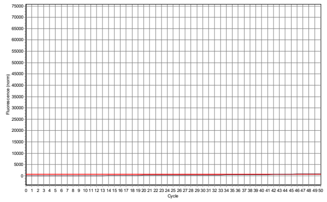

Threshold: 697 (Noiseband)
Baseline settings: automatic, Drift correction OFF

144

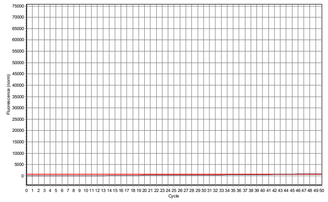

Threshold: 697 (Noiseband)
Baseline settings: automatic, Drift correction OFF

145

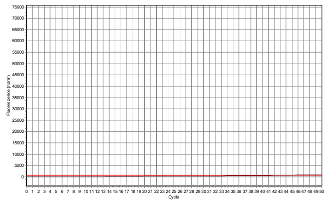

Threshold: 697 (Noiseband)
Baseline settings: automatic, Drift correction OFF

146

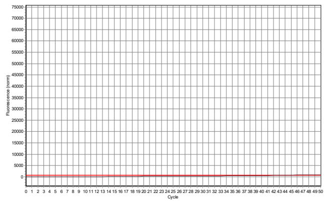

Threshold: 697 (Noiseband)
Baseline settings: automatic, Drift correction OFF

147

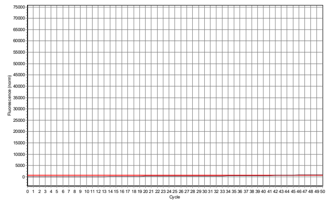

Threshold: 697 (Noiseband)
Baseline settings: automatic, Drift correction OFF

148

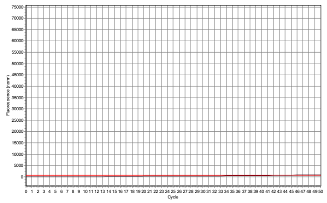

Threshold: 697 (Noiseband)
Baseline settings: automatic, Drift correction OFF

149

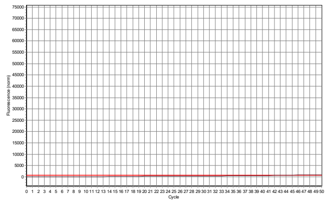

Threshold: 697 (Noiseband)
Baseline settings: automatic, Drift correction OFF

150

Supplemental figure 3

mTOR-TP53BP1 fusion detection

mTOR-TP53BP1-PROSTATE-BLOOD SAMPLE 1-20

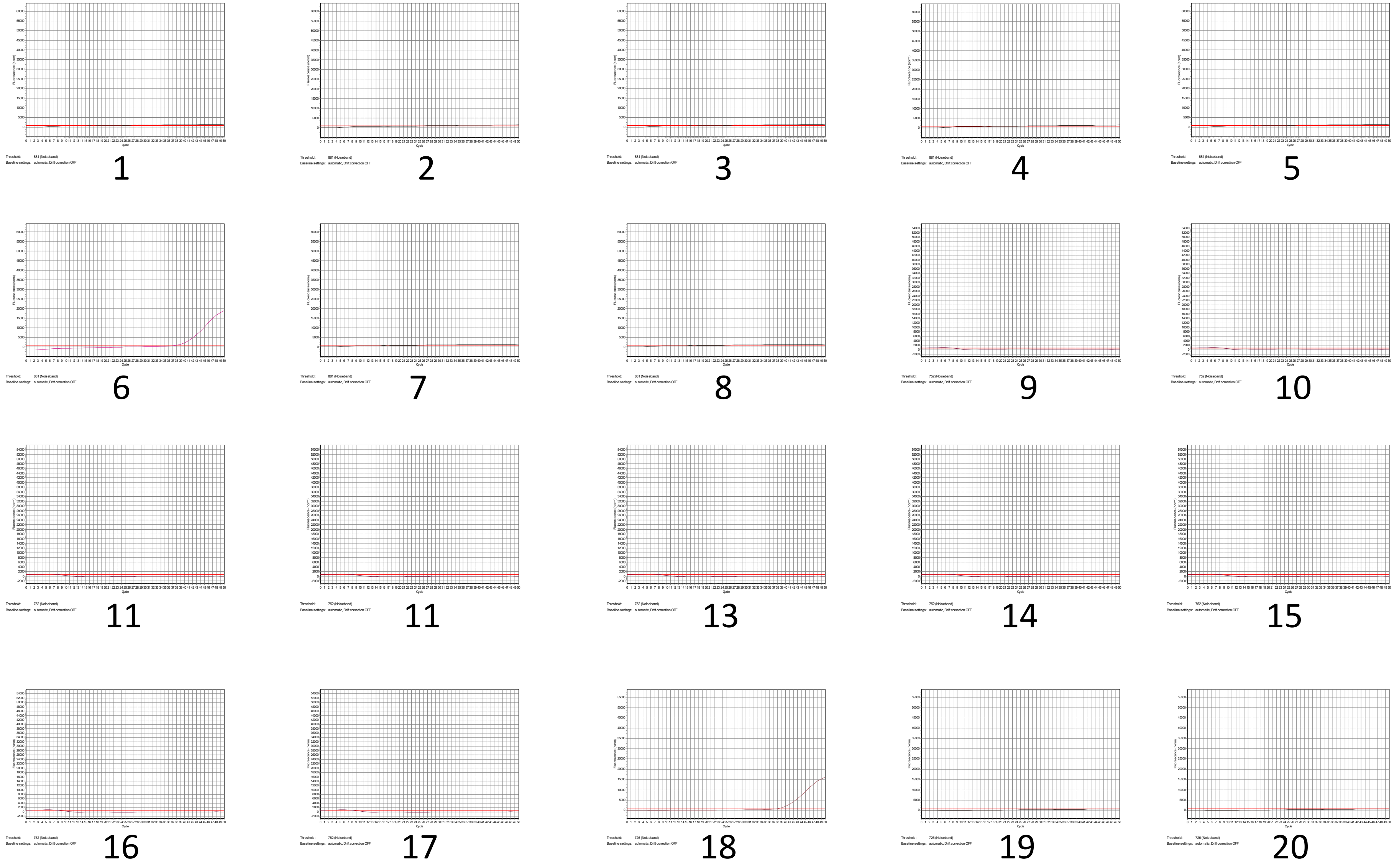

mTOR-TP53BP1-PROSTATE-BLOOD SAMPLE 21-40

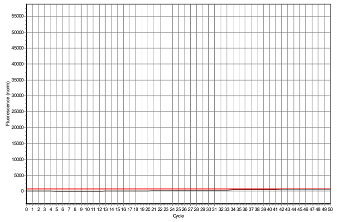

Threshold: 726 (Noiseband)
Baseline settings: automatic, Drift correction OFF

21

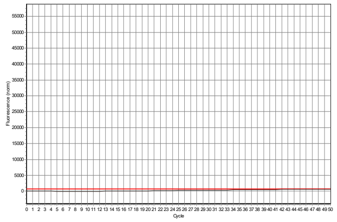

Threshold: 726 (Noiseband)
Baseline settings: automatic, Drift correction OFF

22

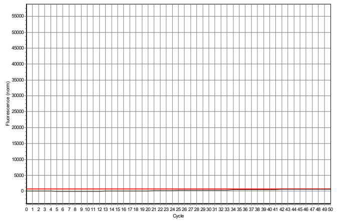

Threshold: 726 (Noiseband)
Baseline settings: automatic, Drift correction OFF

23

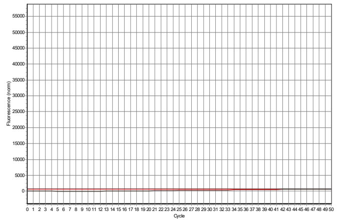

Threshold: 726 (Noiseband)
Baseline settings: automatic, Drift correction OFF

24

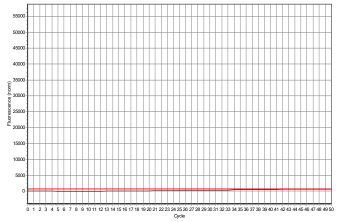

Threshold: 726 (Noiseband)
Baseline settings: automatic, Drift correction OFF

25

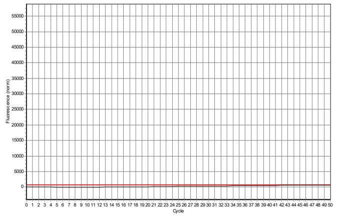

Threshold: 726 (Noiseband)
Baseline settings: automatic, Drift correction OFF

26

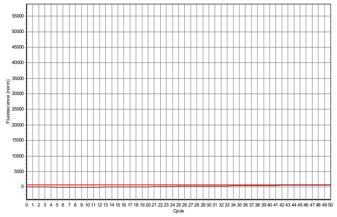

Threshold: 726 (Noiseband)
Baseline settings: automatic, Drift correction OFF

27

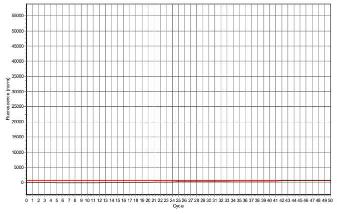

Threshold: 726 (Noiseband)
Baseline settings: automatic, Drift correction OFF

28

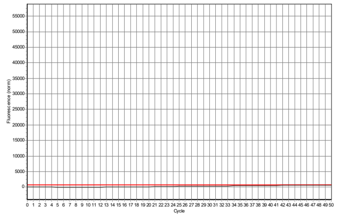

Threshold: 726 (Noiseband)
Baseline settings: automatic, Drift correction OFF

29

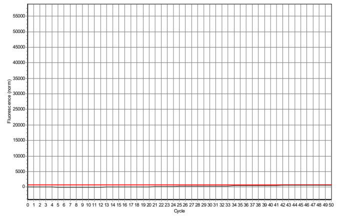

Threshold: 726 (Noiseband)
Baseline settings: automatic, Drift correction OFF

30

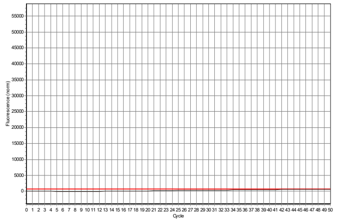

Threshold: 726 (Noiseband)
Baseline settings: automatic, Drift correction OFF

31

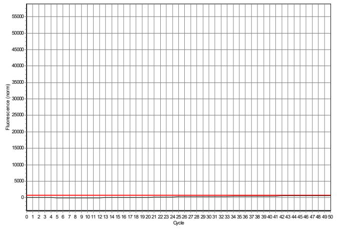

Threshold: 726 (Noiseband)
Baseline settings: automatic, Drift correction OFF

32

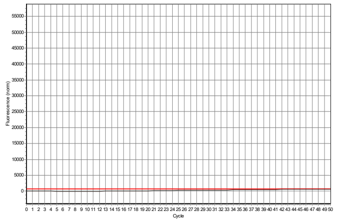

Threshold: 726 (Noiseband)
Baseline settings: automatic, Drift correction OFF

33

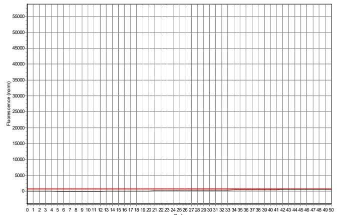

Threshold: 726 (Noiseband)
Baseline settings: automatic, Drift correction OFF

34

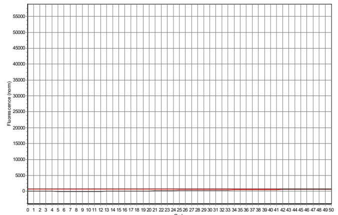

Threshold: 726 (Noiseband)
Baseline settings: automatic, Drift correction OFF

35

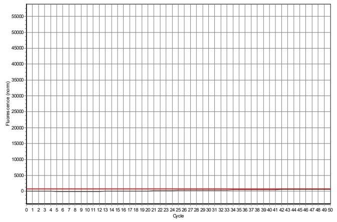

Threshold: 726 (Noiseband)
Baseline settings: automatic, Drift correction OFF

36

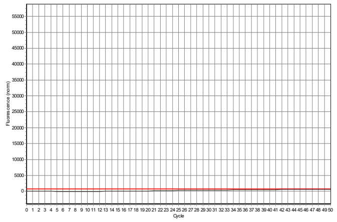

Threshold: 726 (Noiseband)
Baseline settings: automatic, Drift correction OFF

37

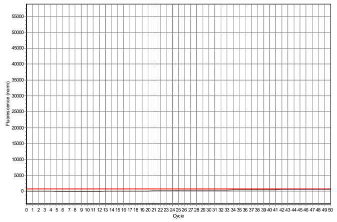

Threshold: 726 (Noiseband)
Baseline settings: automatic, Drift correction OFF

38

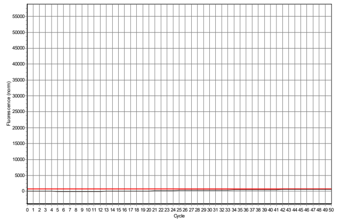

Threshold: 726 (Noiseband)
Baseline settings: automatic, Drift correction OFF

39

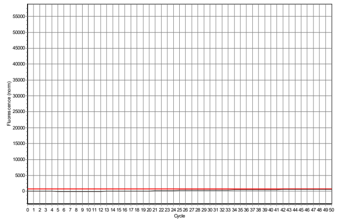

Threshold: 726 (Noiseband)
Baseline settings: automatic, Drift correction OFF

40

mTOR-TP53BP1-PROSTATE-BLOOD SAMPLE 41-60

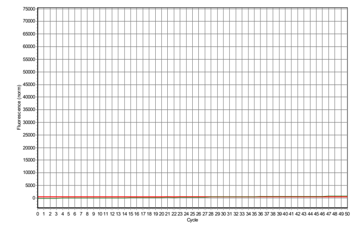

Threshold: 493 (Noiseband)
Baseline settings: automatic, Drift correction OFF

41

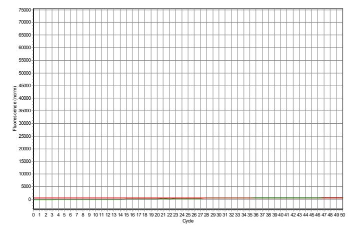

Threshold: 493 (Noiseband)
Baseline settings: automatic, Drift correction OFF

42

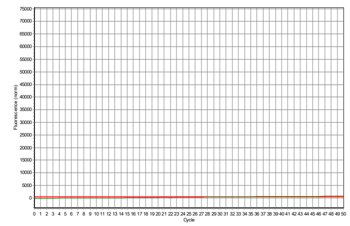

Threshold: 493 (Noiseband)
Baseline settings: automatic, Drift correction OFF

43

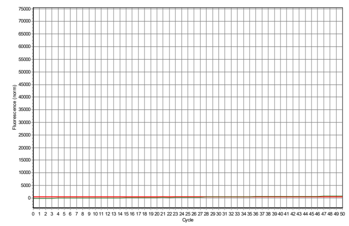

Threshold: 493 (Noiseband)
Baseline settings: automatic, Drift correction OFF

44

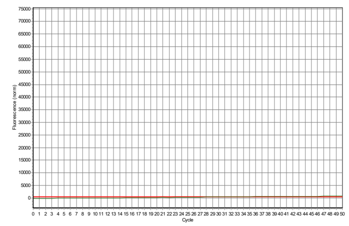

Threshold: 493 (Noiseband)
Baseline settings: automatic, Drift correction OFF

45

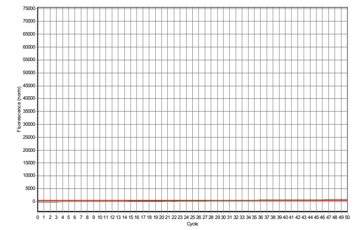

Threshold: 493 (Noiseband)
Baseline settings: automatic, Drift correction OFF

46

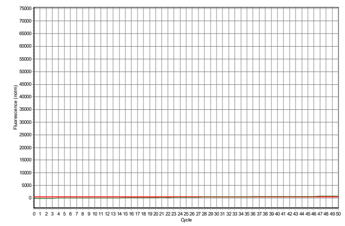

Threshold: 493 (Noiseband)
Baseline settings: automatic, Drift correction OFF

47

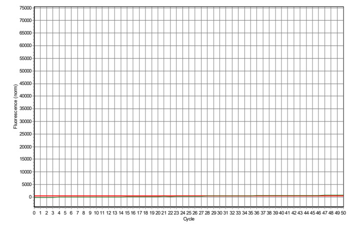

Threshold: 493 (Noiseband)
Baseline settings: automatic, Drift correction OFF

47

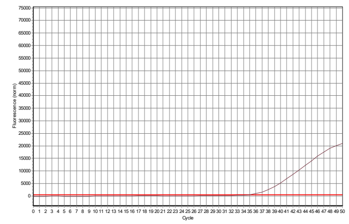

Threshold: 493 (Noiseband)
Baseline settings: automatic, Drift correction OFF

49

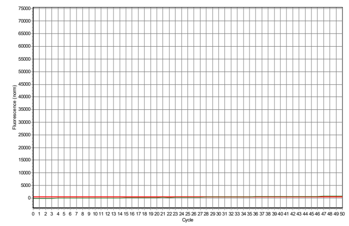

Threshold: 493 (Noiseband)
Baseline settings: automatic, Drift correction OFF

50

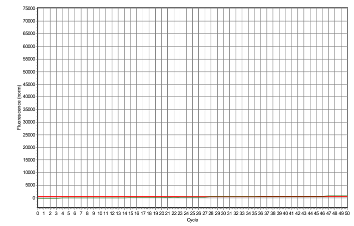

Threshold: 493 (Noiseband)
Baseline settings: automatic, Drift correction OFF

51

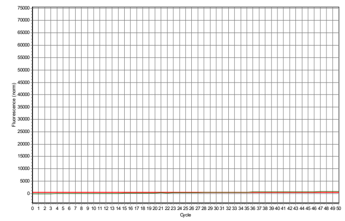

Threshold: 493 (Noiseband)
Baseline settings: automatic, Drift correction OFF

52

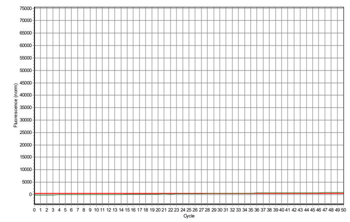

Threshold: 493 (Noiseband)
Baseline settings: automatic, Drift correction OFF

53

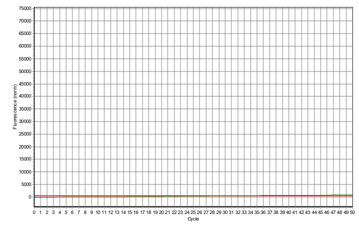

Threshold: 493 (Noiseband)
Baseline settings: automatic, Drift correction OFF

54

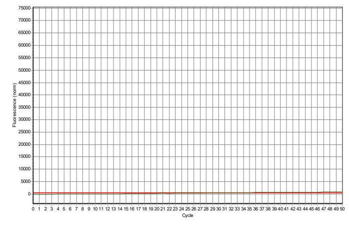

Threshold: 493 (Noiseband)
Baseline settings: automatic, Drift correction OFF

55

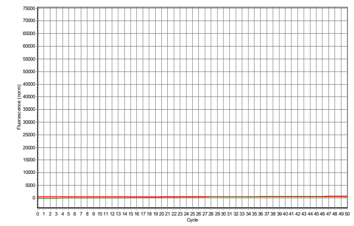

Threshold: 493 (Noiseband)
Baseline settings: automatic, Drift correction OFF

56

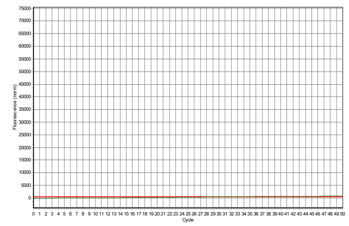

Threshold: 493 (Noiseband)
Baseline settings: automatic, Drift correction OFF

57

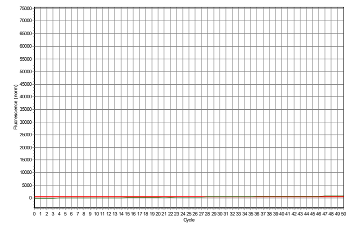

Threshold: 493 (Noiseband)
Baseline settings: automatic, Drift correction OFF

58

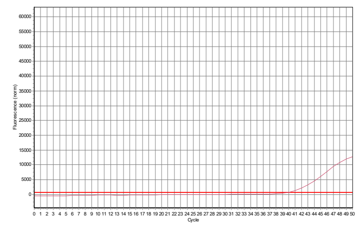

Threshold: 759 (Noiseband)
Baseline settings: automatic, Drift correction OFF

59

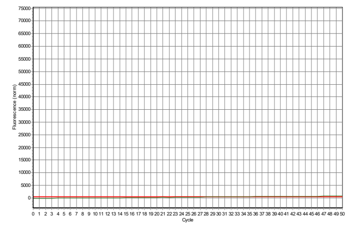

Threshold: 493 (Noiseband)
Baseline settings: automatic, Drift correction OFF

60

mTOR-TP53BP1-PROSTATE-BLOOD SAMPLE 61-80

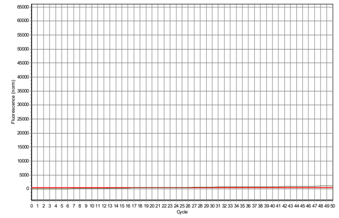

Threshold: 516 (Noiseband)
Baseline settings: automatic, Drift correction OFF

61

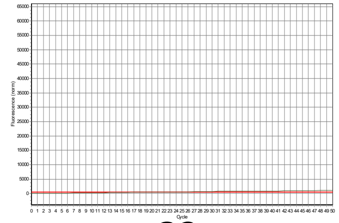

Threshold: 516 (Noiseband)
Baseline settings: automatic, Drift correction OFF

62

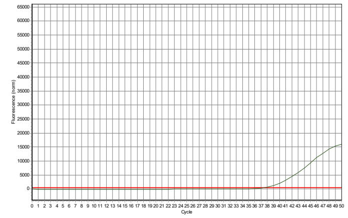

Threshold: 516 (Noiseband)
Baseline settings: automatic, Drift correction OFF

63

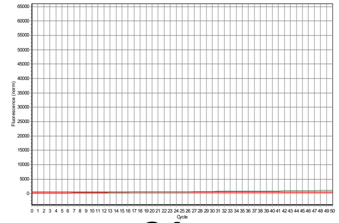

Threshold: 516 (Noiseband)
Baseline settings: automatic, Drift correction OFF

64

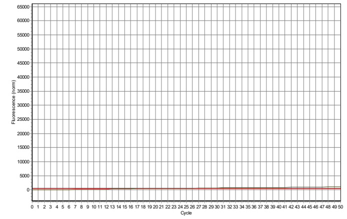

Threshold: 516 (Noiseband)
Baseline settings: automatic, Drift correction OFF

65

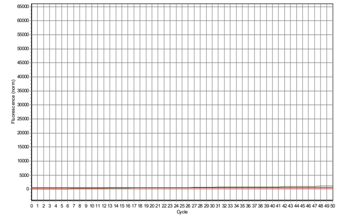

Threshold: 516 (Noiseband)
Baseline settings: automatic, Drift correction OFF

66

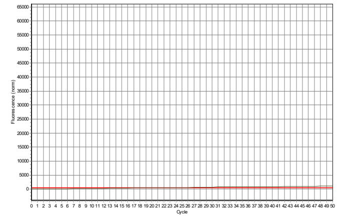

Threshold: 516 (Noiseband)
Baseline settings: automatic, Drift correction OFF

67

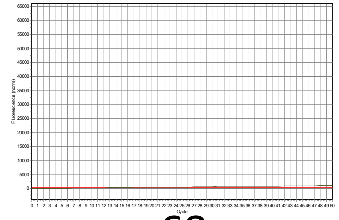

Threshold: 516 (Noiseband)
Baseline settings: automatic, Drift correction OFF

68

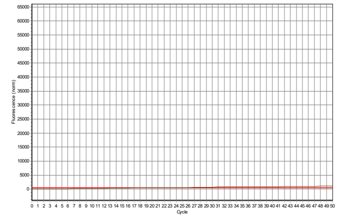

Threshold: 516 (Noiseband)
Baseline settings: automatic, Drift correction OFF

69

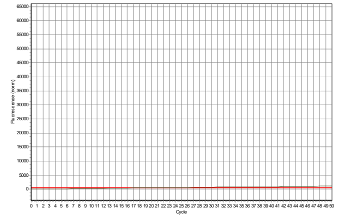

Threshold: 516 (Noiseband)
Baseline settings: automatic, Drift correction OFF

70

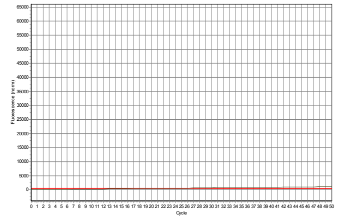

Threshold: 516 (Noiseband)
Baseline settings: automatic, Drift correction OFF

71

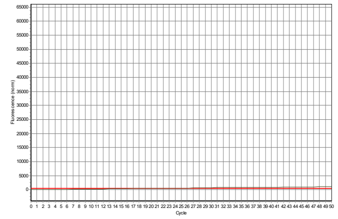

Threshold: 516 (Noiseband)
Baseline settings: automatic, Drift correction OFF

72

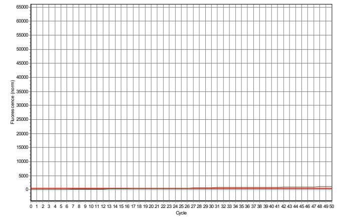

Threshold: 516 (Noiseband)
Baseline settings: automatic, Drift correction OFF

73

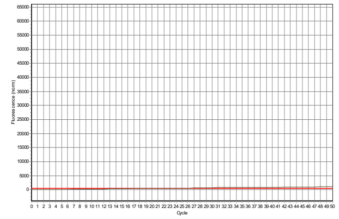

Threshold: 516 (Noiseband)
Baseline settings: automatic, Drift correction OFF

74

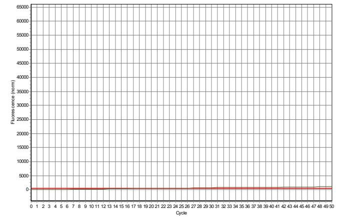

Threshold: 516 (Noiseband)
Baseline settings: automatic, Drift correction OFF

75

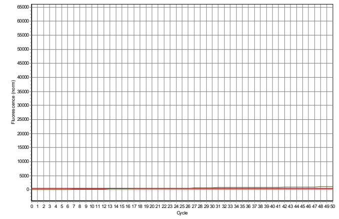

Threshold: 516 (Noiseband)
Baseline settings: automatic, Drift correction OFF

76

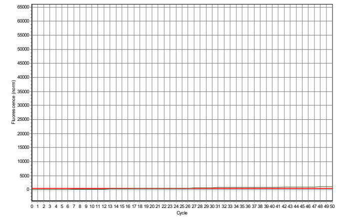

Threshold: 516 (Noiseband)
Baseline settings: automatic, Drift correction OFF

77

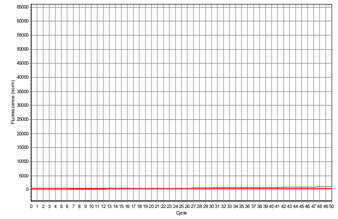

Threshold: 516 (Noiseband)
Baseline settings: automatic, Drift correction OFF

78

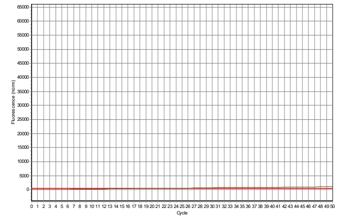

Threshold: 516 (Noiseband)
Baseline settings: automatic, Drift correction OFF

79

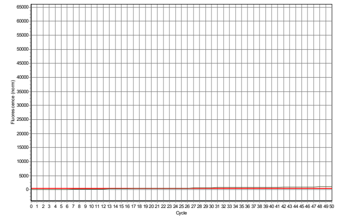

Threshold: 516 (Noiseband)
Baseline settings: automatic, Drift correction OFF

80

mTOR-TP53BP1-PROSTATE-BLOOD SAMPLE 81-100

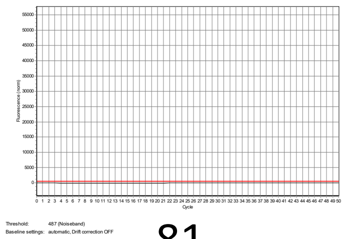
81

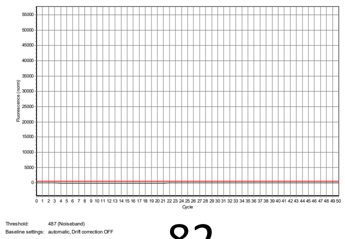
82

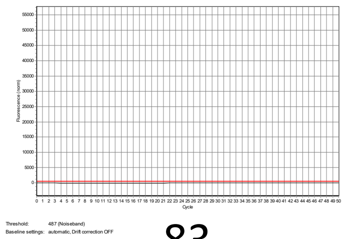
83

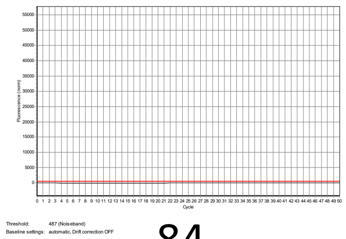
84

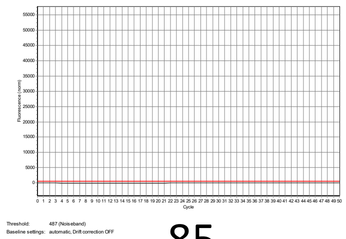
85

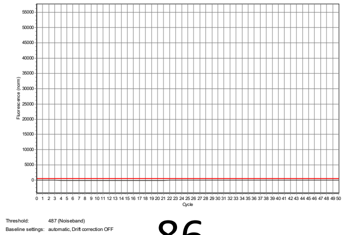
86

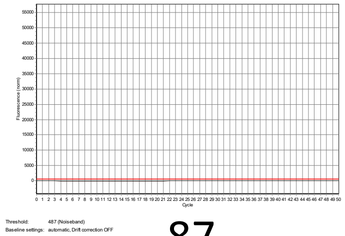
87

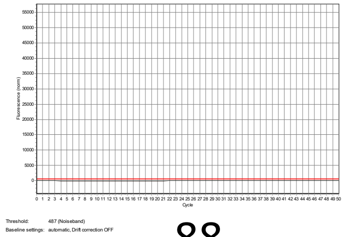
88

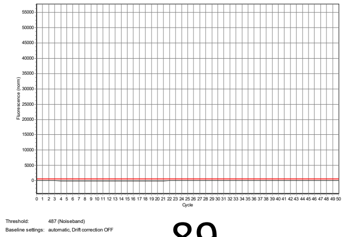
89

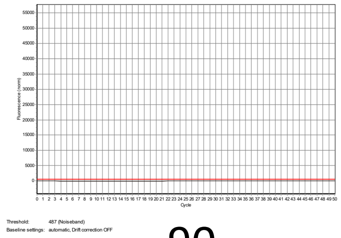
90

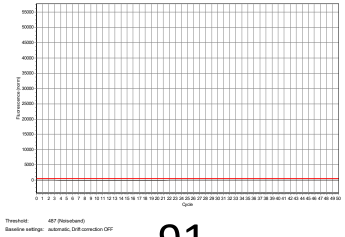
91

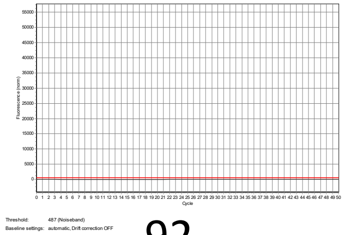
92

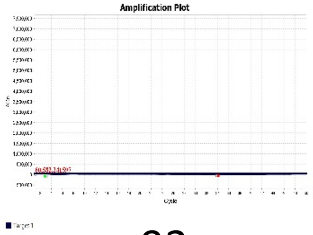

93

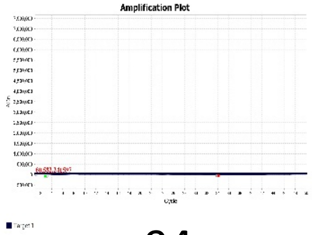

94

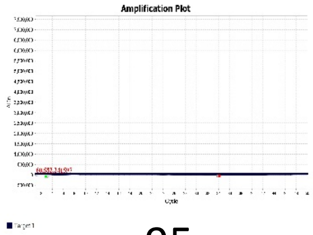

95

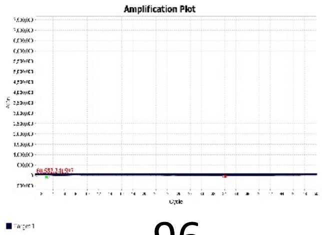

96

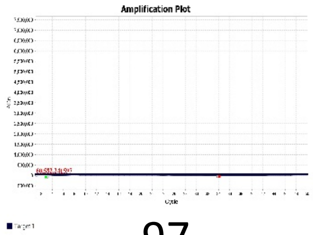

97

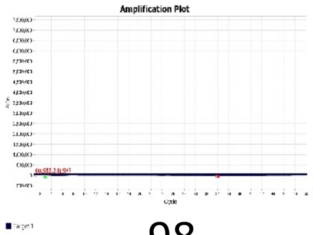

98

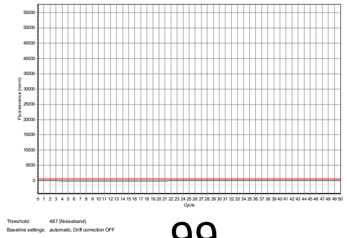
99

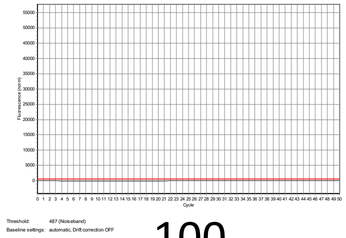
100

mTOR-TP53BP1-PROSTATE-BLOOD SAMPLE 101-120

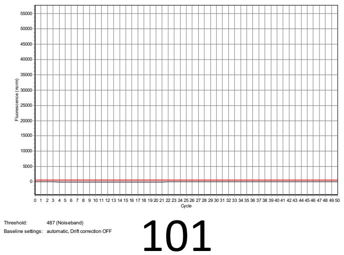

101

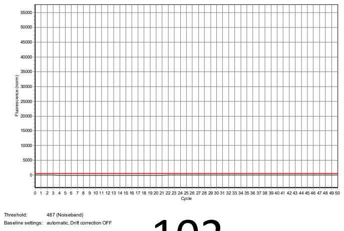

102

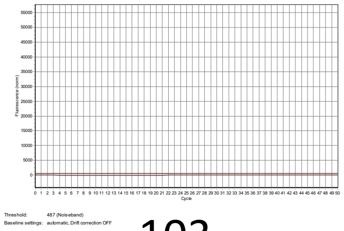

103

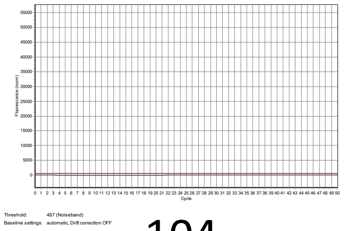

104

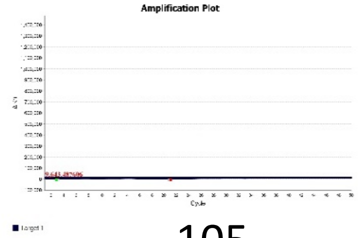

105

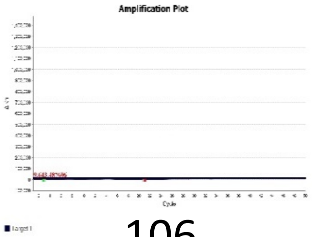

106

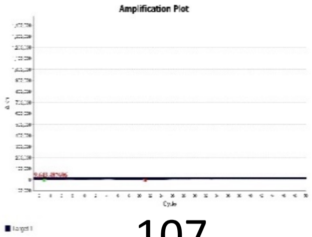

107

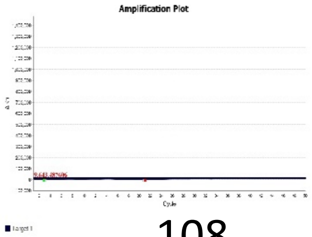

108

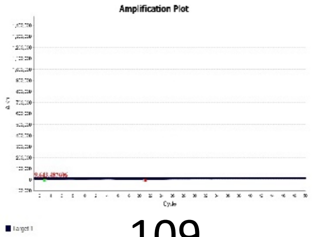

109

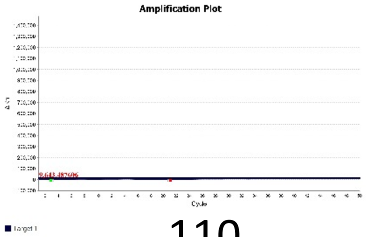

110

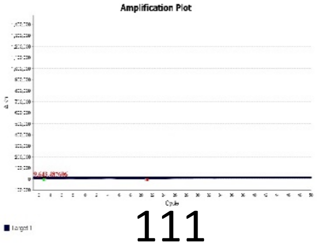

111

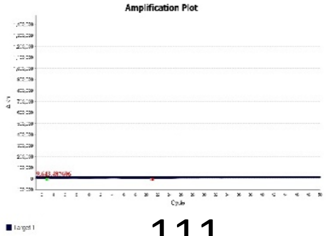

111

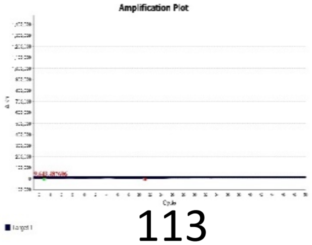

113

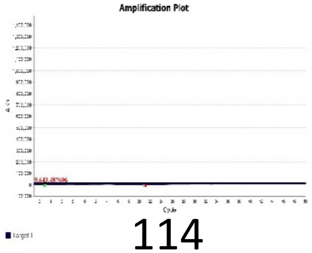

114

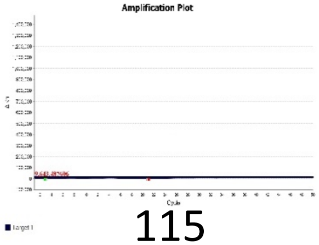

115

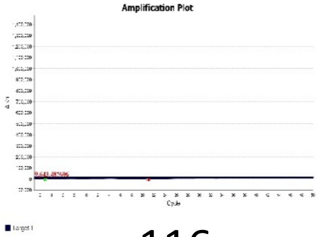

116

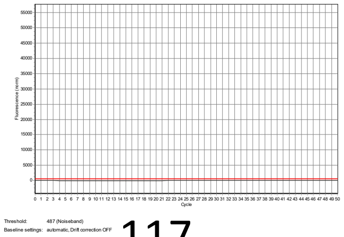

117

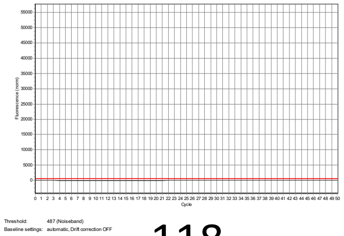

118

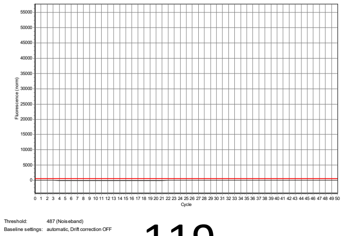

119

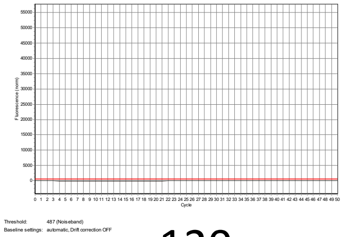

120

mTOR-TP53BP1-PROSTATE-BLOOD SAMPLE 121-140

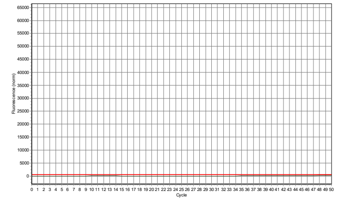

Threshold: 585 (Noiseband)
Baseline settings: automatic, Drift correction OFF

121

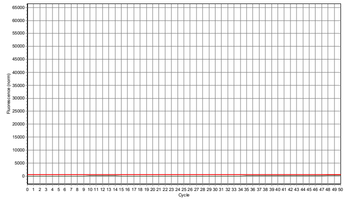

Threshold: 585 (Noiseband)
Baseline settings: automatic, Drift correction OFF

122

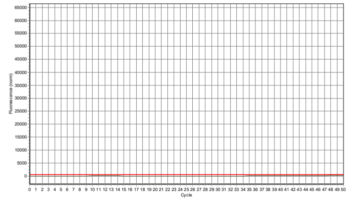

Threshold: 585 (Noiseband)
Baseline settings: automatic, Drift correction OFF

123

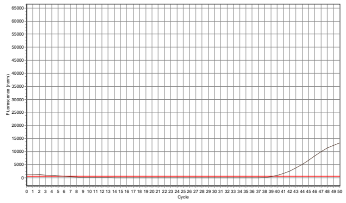

Threshold: 585 (Noiseband)
Baseline settings: automatic, Drift correction OFF

124

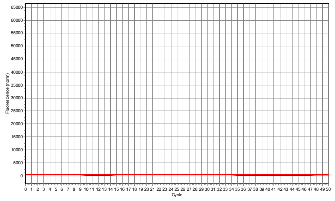

Threshold: 585 (Noiseband)
Baseline settings: automatic, Drift correction OFF

125

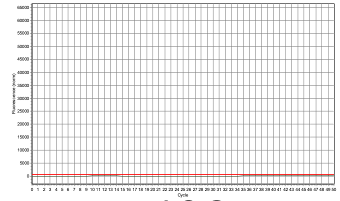

Threshold: 585 (Noiseband)
Baseline settings: automatic, Drift correction OFF

126

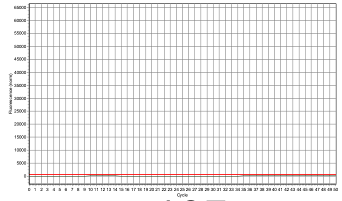

Threshold: 585 (Noiseband)
Baseline settings: automatic, Drift correction OFF

127

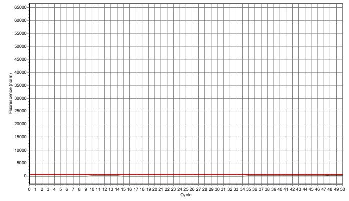

Threshold: 585 (Noiseband)
Baseline settings: automatic, Drift correction OFF

128

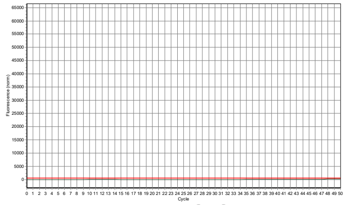

Threshold: 585 (Noiseband)
Baseline settings: automatic, Drift correction OFF

129

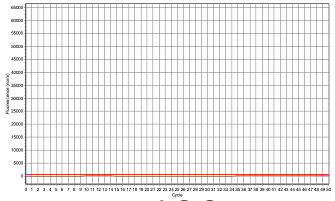

Threshold: 585 (Noiseband)
Baseline settings: automatic, Drift correction OFF

130

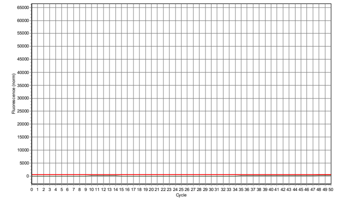

Threshold: 585 (Noiseband)
Baseline settings: automatic, Drift correction OFF

131

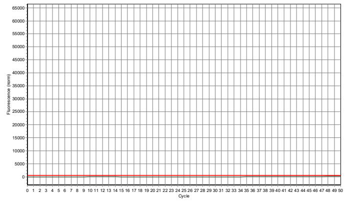

Threshold: 585 (Noiseband)
Baseline settings: automatic, Drift correction OFF

132

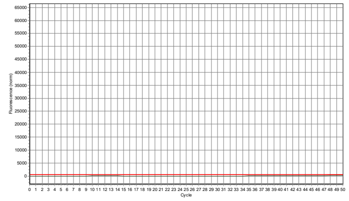

Threshold: 585 (Noiseband)
Baseline settings: automatic, Drift correction OFF

133

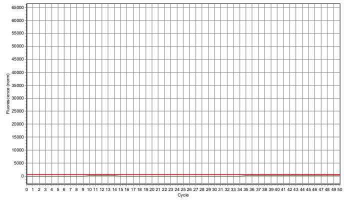

Threshold: 585 (Noiseband)
Baseline settings: automatic, Drift correction OFF

134

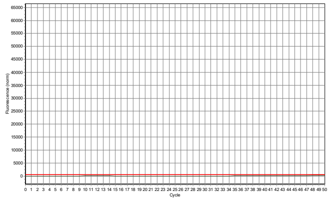

Threshold: 585 (Noiseband)
Baseline settings: automatic, Drift correction OFF

135

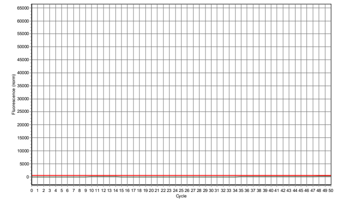

Threshold: 585 (Noiseband)
Baseline settings: automatic, Drift correction OFF

136

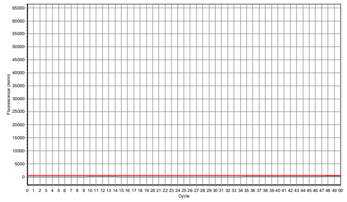

Threshold: 585 (Noiseband)
Baseline settings: automatic, Drift correction OFF

137

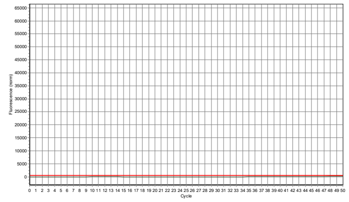

Threshold: 585 (Noiseband)
Baseline settings: automatic, Drift correction OFF

138

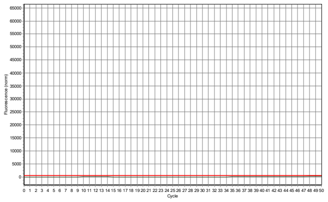

Threshold: 585 (Noiseband)
Baseline settings: automatic, Drift correction OFF

139

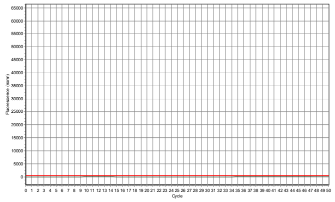

Threshold: 585 (Noiseband)
Baseline settings: automatic, Drift correction OFF

140

mTOR-TP53BP1-PROSTATE-BLOOD SAMPLE 141-150

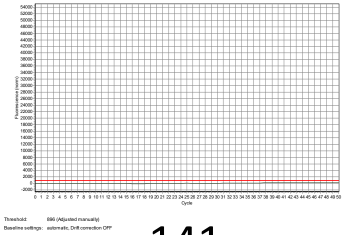

141

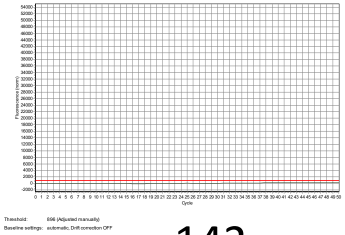

142

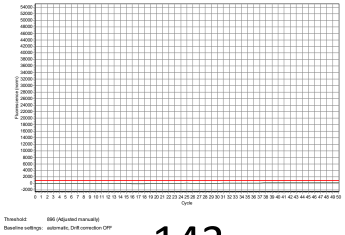

143

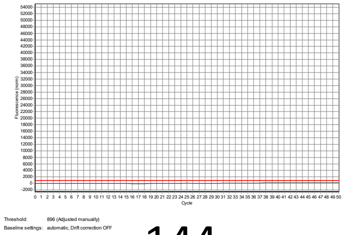

144

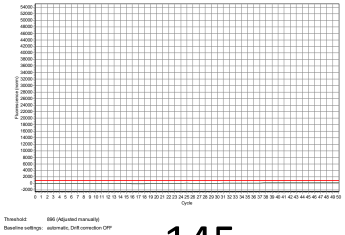

145

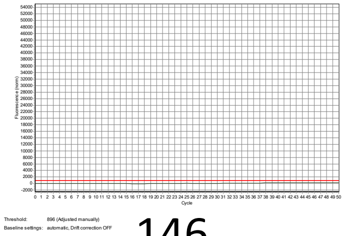

146

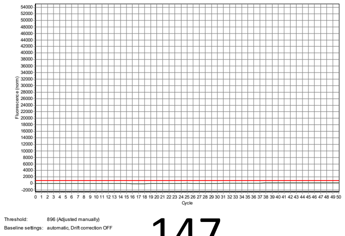

147

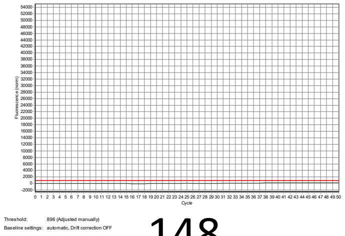

148

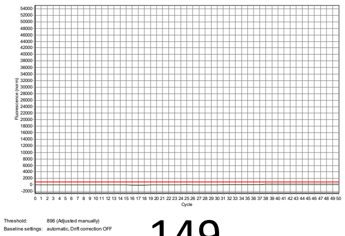

149

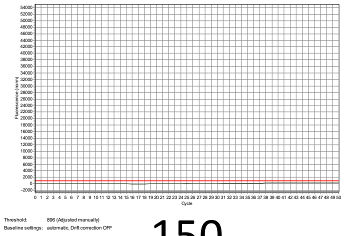

150

Supplemental figure 4

CCNH-c5orf30 fusion detection
CCNH-c5orf30-PROSTATE-BLOOD SAMPLE 1-20

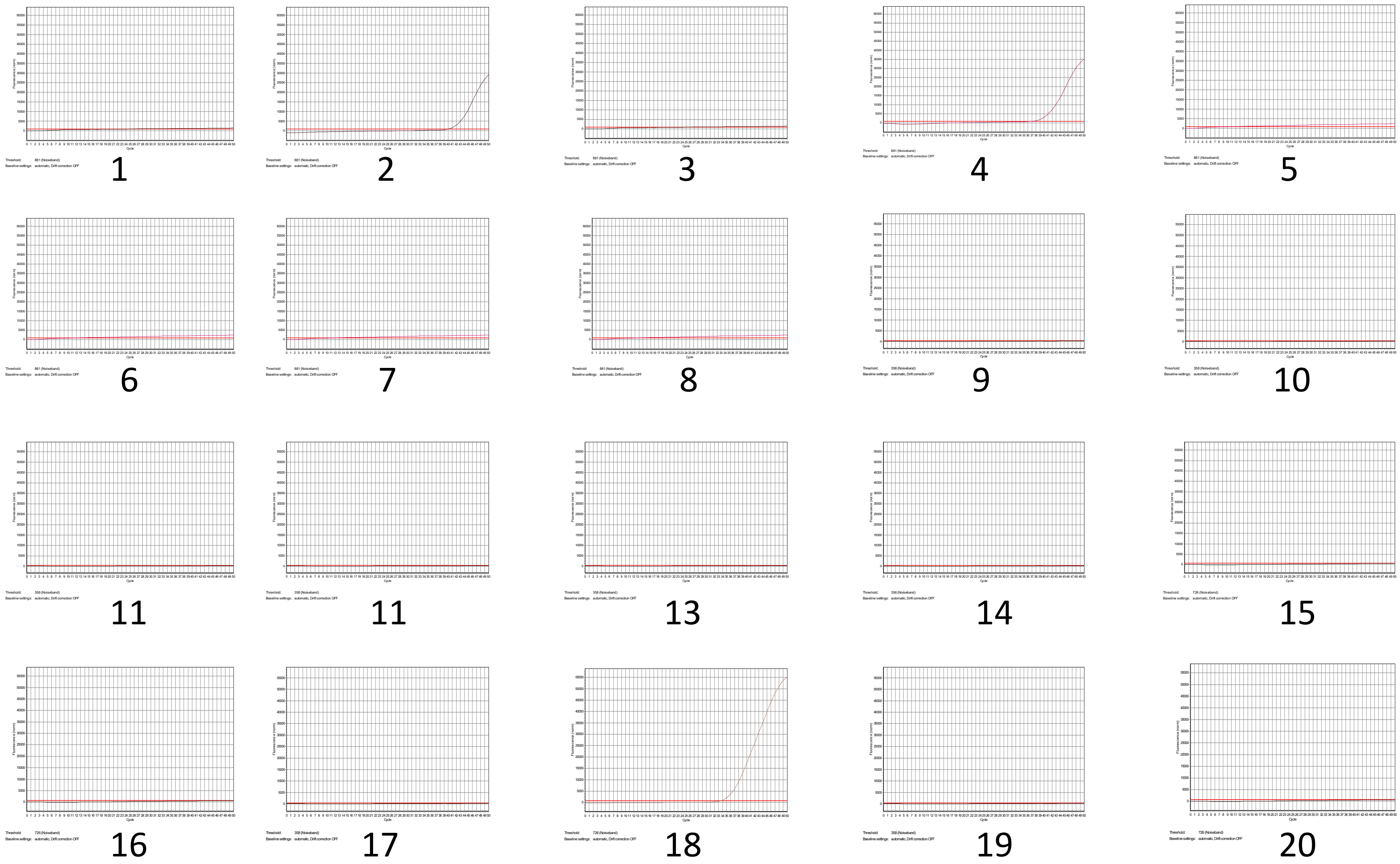

CCNH-c5orf30-PROSTATE-BLOOD SAMPLE 21-40

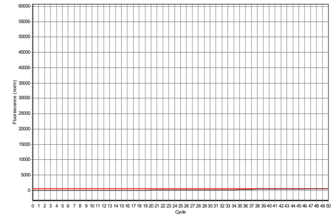

Threshold: 507 (Noiseband)
Baseline settings: automatic, Drift correction OFF

21

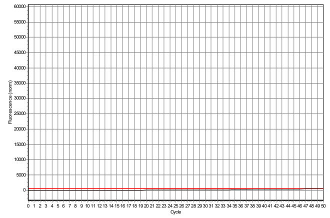

Threshold: 507 (Noiseband)
Baseline settings: automatic, Drift correction OFF

22

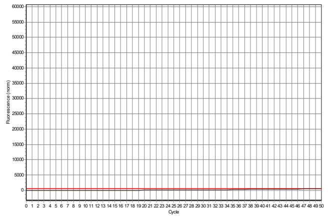

Threshold: 507 (Noiseband)
Baseline settings: automatic, Drift correction OFF

23

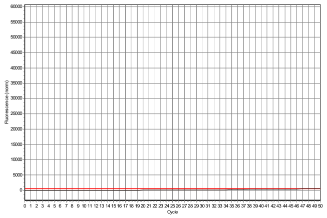

Threshold: 507 (Noiseband)
Baseline settings: automatic, Drift correction OFF

24

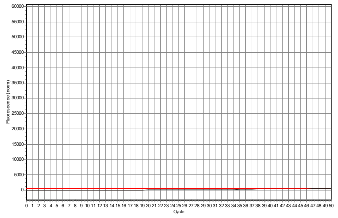

Threshold: 507 (Noiseband)
Baseline settings: automatic, Drift correction OFF

25

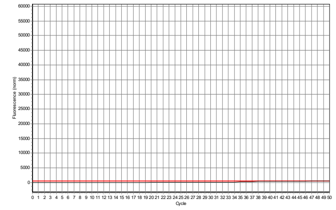

Threshold: 507 (Noiseband)
Baseline settings: automatic, Drift correction OFF

26

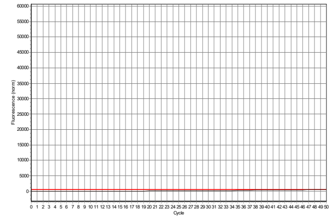

Threshold: 507 (Noiseband)
Baseline settings: automatic, Drift correction OFF

27

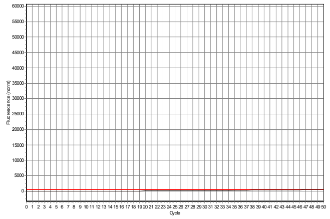

Threshold: 507 (Noiseband)
Baseline settings: automatic, Drift correction OFF

28

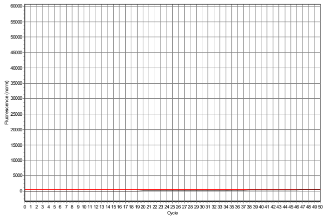

Threshold: 507 (Noiseband)
Baseline settings: automatic, Drift correction OFF

29

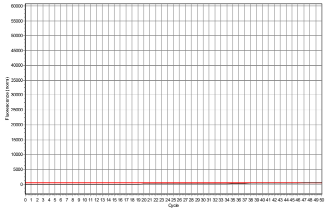

Threshold: 507 (Noiseband)
Baseline settings: automatic, Drift correction OFF

30

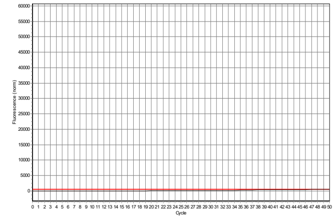

Threshold: 507 (Noiseband)
Baseline settings: automatic, Drift correction OFF

31

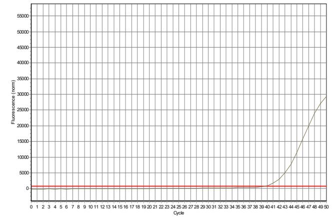

Threshold: 726 (Noiseband)
Baseline settings: automatic, Drift correction OFF

32

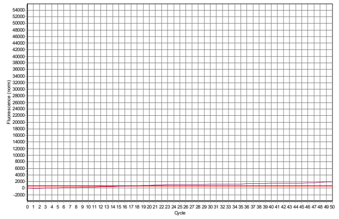

Threshold: 706 (Noiseband)
Baseline settings: automatic, Drift correction OFF

33

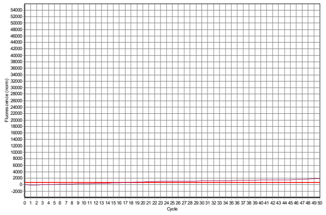

Threshold: 706 (Noiseband)
Baseline settings: automatic, Drift correction OFF

34

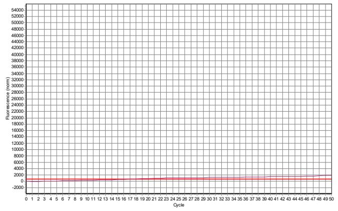

Threshold: 706 (Noiseband)
Baseline settings: automatic, Drift correction OFF

35

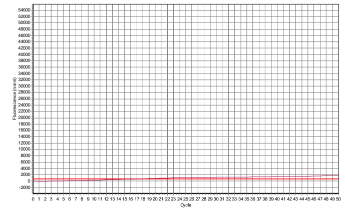

Threshold: 706 (Noiseband)
Baseline settings: automatic, Drift correction OFF

36

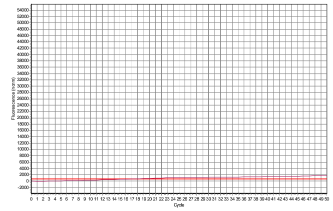

Threshold: 706 (Noiseband)
Baseline settings: automatic, Drift correction OFF

37

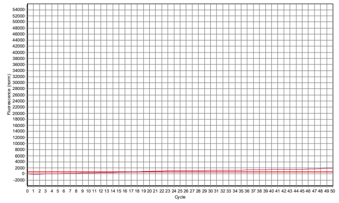

Threshold: 706 (Noiseband)
Baseline settings: automatic, Drift correction OFF

38

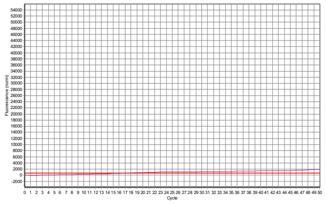

Threshold: 706 (Noiseband)
Baseline settings: automatic, Drift correction OFF

39

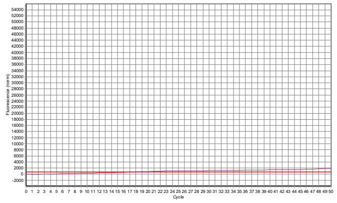

Threshold: 706 (Noiseband)
Baseline settings: automatic, Drift correction OFF

40

CCNH-c5orf30-PROSTATE-BLOOD SAMPLE 41-60

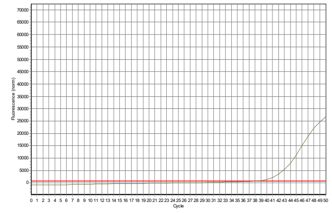

Threshold: 781 (Noiseband)
Baseline settings: automatic, Drift correction OFF

41

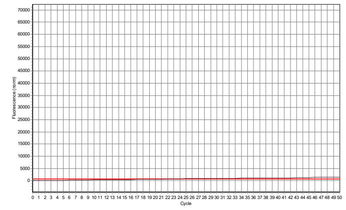

Threshold: 781 (Noiseband)
Baseline settings: automatic, Drift correction OFF

42

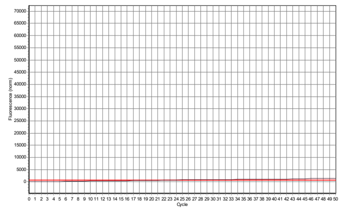

Threshold: 781 (Noiseband)
Baseline settings: automatic, Drift correction OFF

43

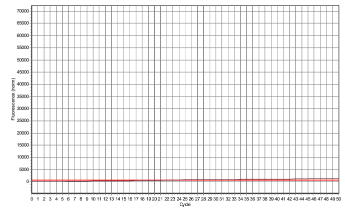

Threshold: 781 (Noiseband)
Baseline settings: automatic, Drift correction OFF

44

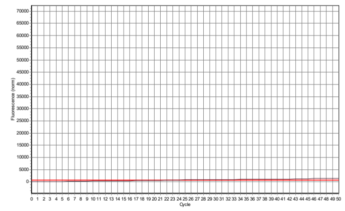

Threshold: 781 (Noiseband)
Baseline settings: automatic, Drift correction OFF

45

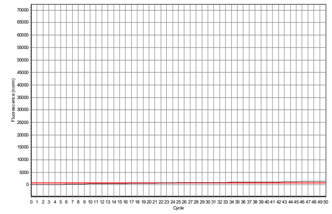

Threshold: 781 (Noiseband)
Baseline settings: automatic, Drift correction OFF

46

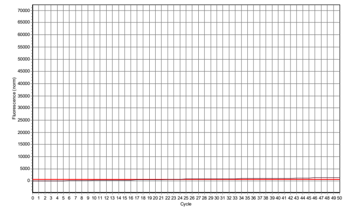

Threshold: 781 (Noiseband)
Baseline settings: automatic, Drift correction OFF

47

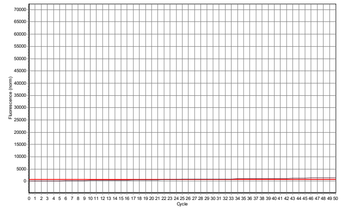

Threshold: 781 (Noiseband)
Baseline settings: automatic, Drift correction OFF

47

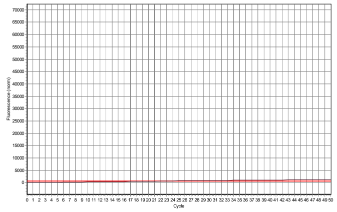

Threshold: 781 (Noiseband)
Baseline settings: automatic, Drift correction OFF

49

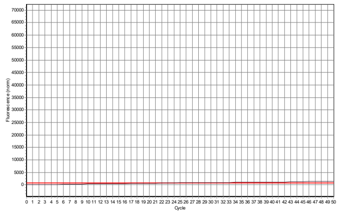

Threshold: 781 (Noiseband)
Baseline settings: automatic, Drift correction OFF

50

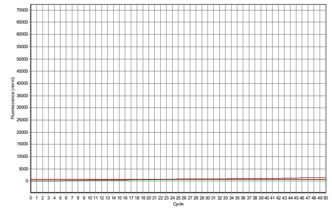

Threshold: 781 (Noiseband)
Baseline settings: automatic, Drift correction OFF

51

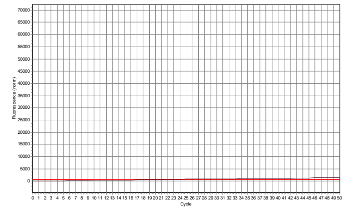

Threshold: 781 (Noiseband)
Baseline settings: automatic, Drift correction OFF

52

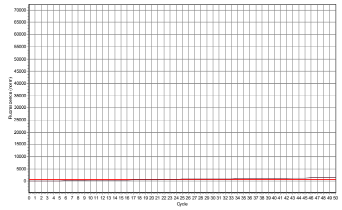

Threshold: 781 (Noiseband)
Baseline settings: automatic, Drift correction OFF

53

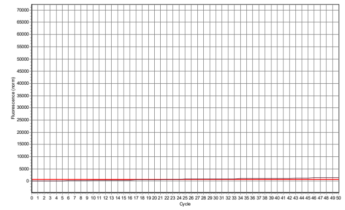

Threshold: 781 (Noiseband)
Baseline settings: automatic, Drift correction OFF

54

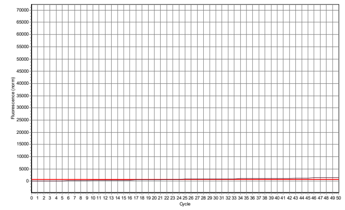

Threshold: 781 (Noiseband)
Baseline settings: automatic, Drift correction OFF

55

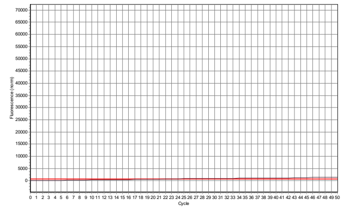

Threshold: 781 (Noiseband)
Baseline settings: automatic, Drift correction OFF

56

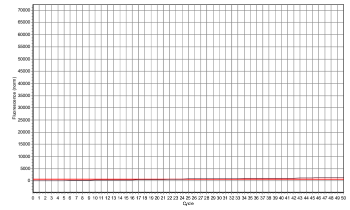

Threshold: 781 (Noiseband)
Baseline settings: automatic, Drift correction OFF

57

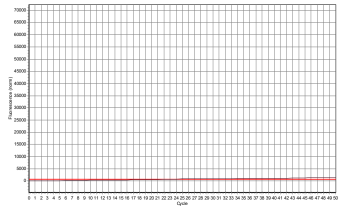

Threshold: 781 (Noiseband)
Baseline settings: automatic, Drift correction OFF

58

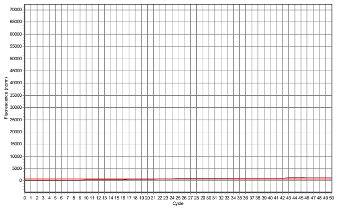

Threshold: 781 (Noiseband)
Baseline settings: automatic, Drift correction OFF

59

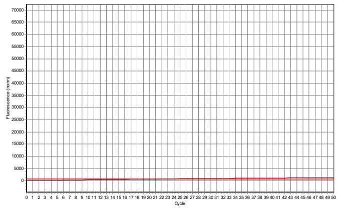

Threshold: 781 (Noiseband)
Baseline settings: automatic, Drift correction OFF

60

CCNH-c5orf30-PROSTATE-BLOOD SAMPLE 61-80

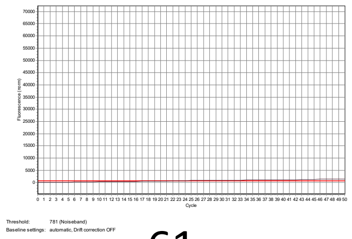

61

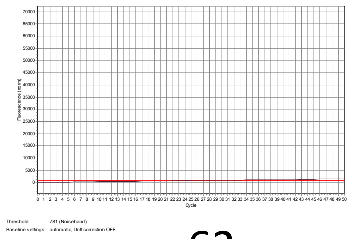

62

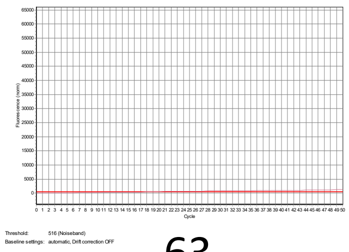

63

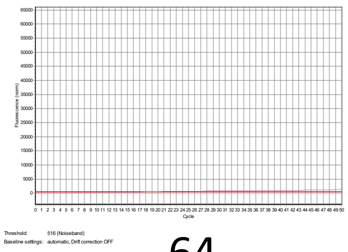

64

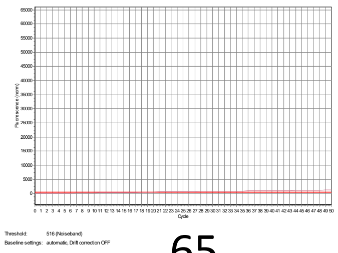

65

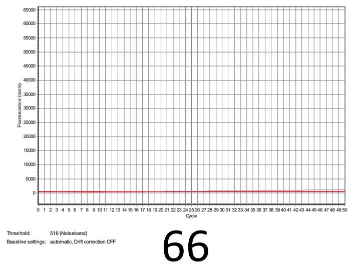

66

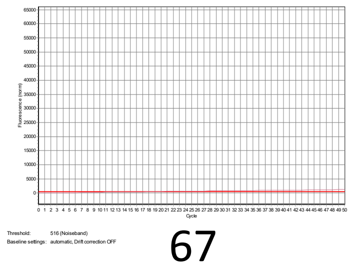

67

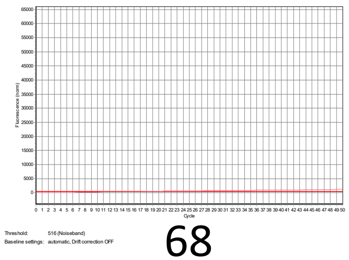

68

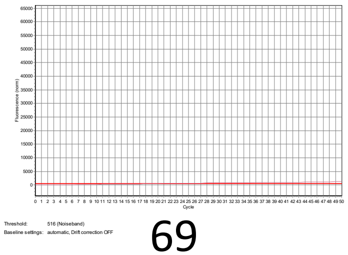

69

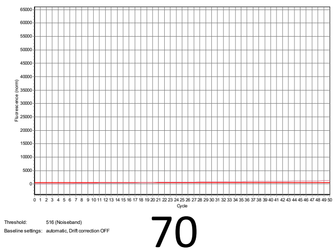

70

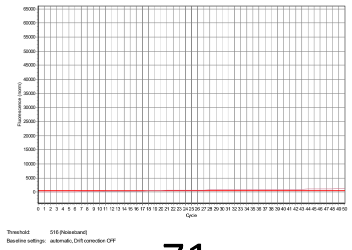

71

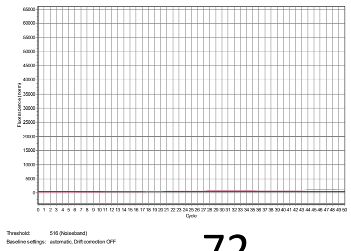

72

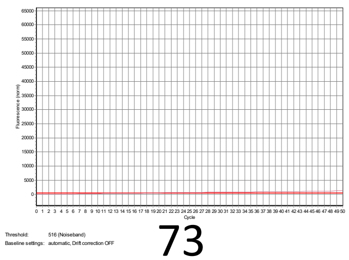

73

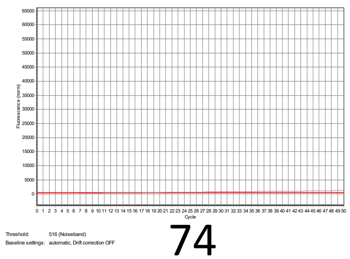

74

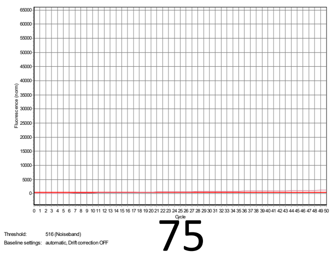

75

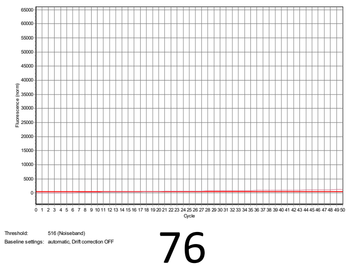

76

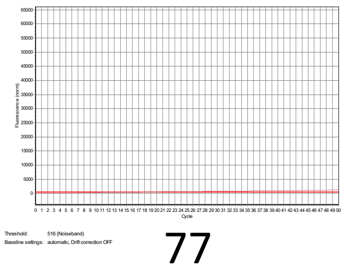

77

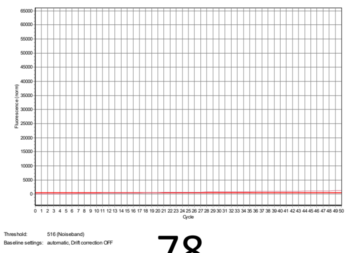

78

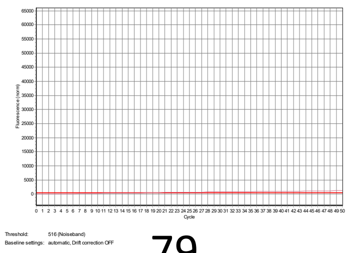

79

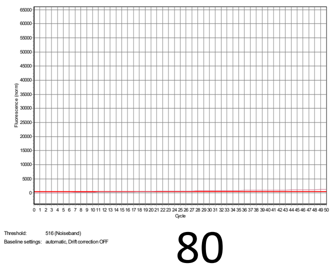

80

CCNH-c5orf30-PROSTATE-BLOOD SAMPLE 81-100

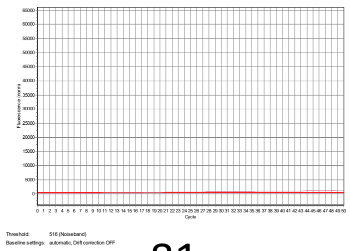

81

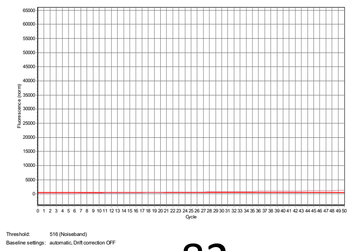

82

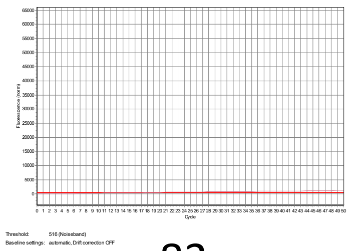

83

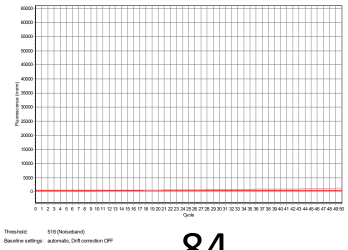

84

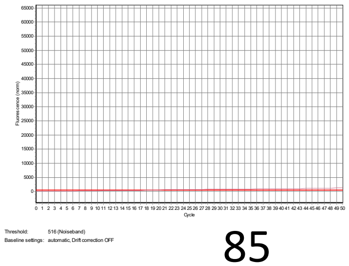

85

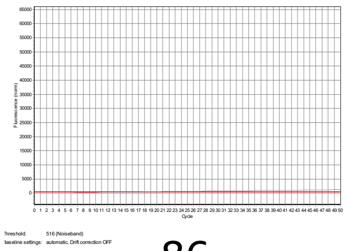

86

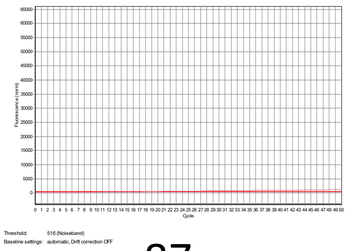

87

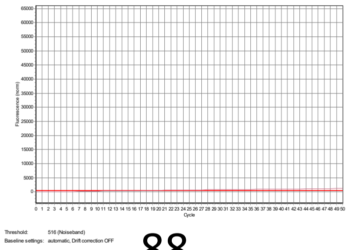

88

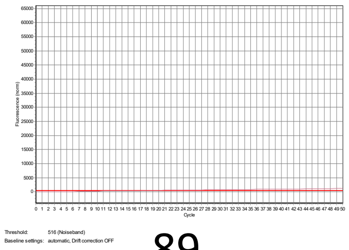

89

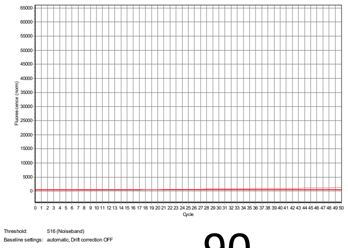

90

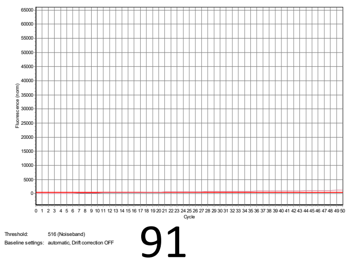

91

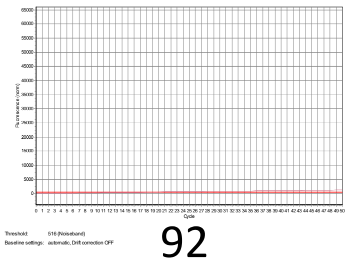

92

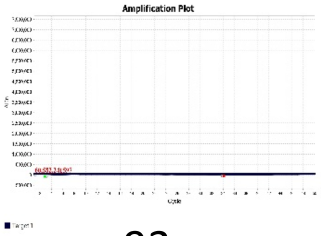

93

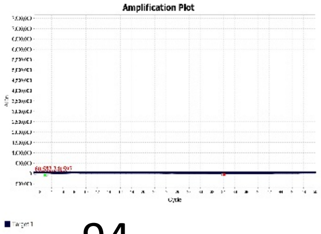

94

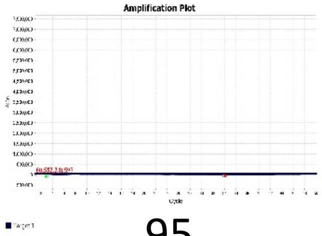

95

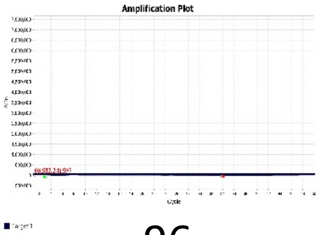

96

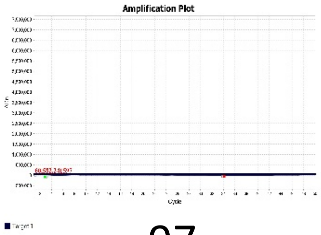

97

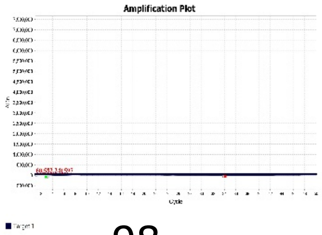

98

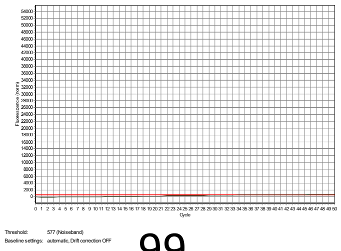

99

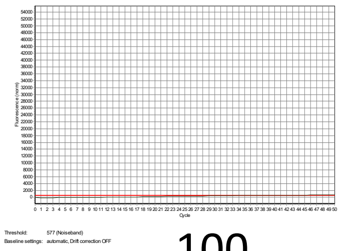

100

CCNH-c5orf30-PROSTATE-BLOOD SAMPLE 101-120

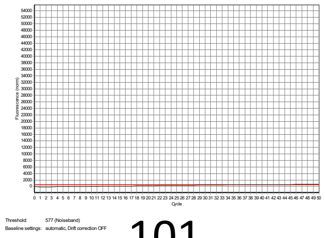
101

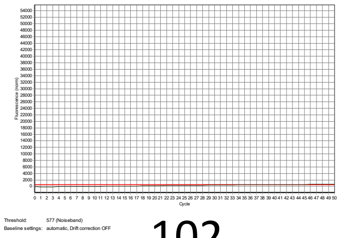
102

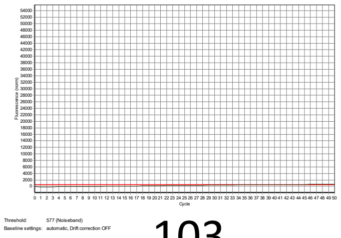
103

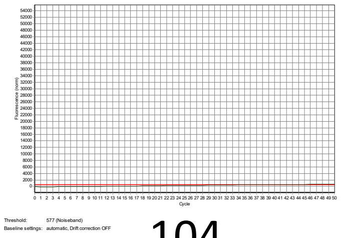
104

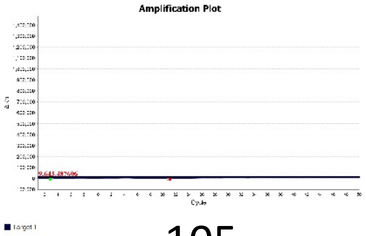

105

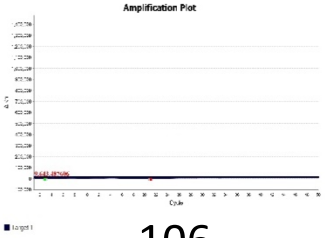

106

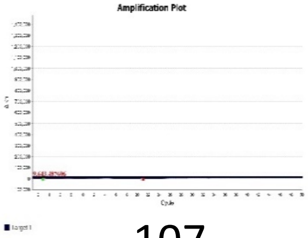

107

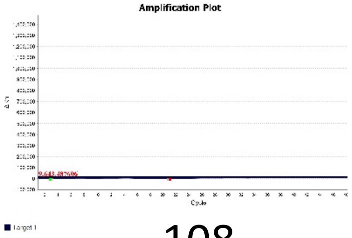

108

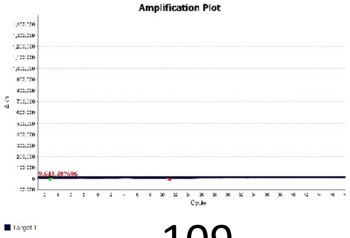

109

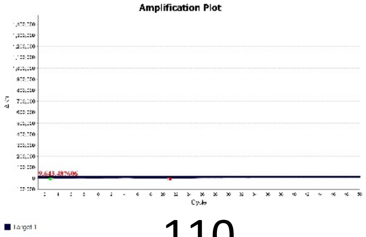

110

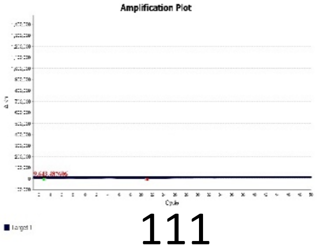

111

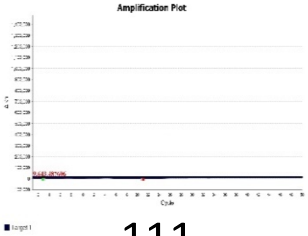

111

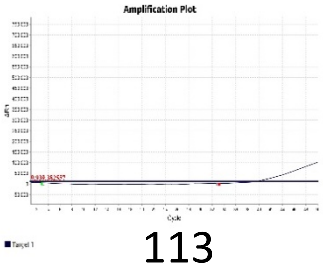

113

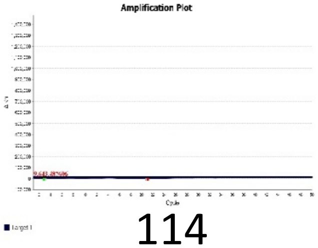

114

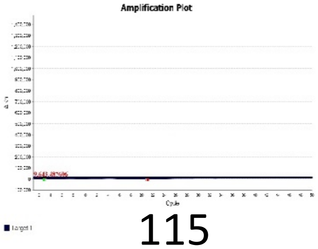

115

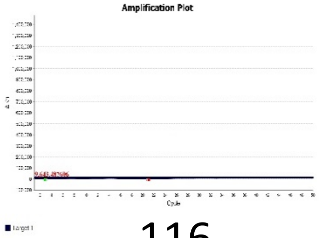

116

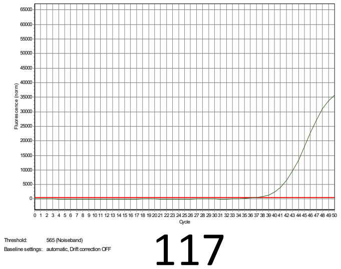
117

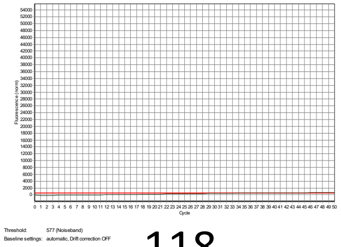
118

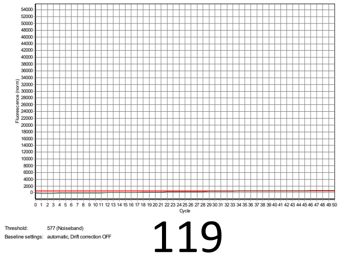
119

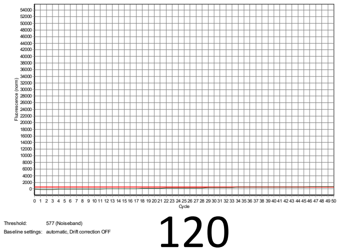
120

CCNH-c5orf30-PROSTATE-BLOOD SAMPLE 121-140

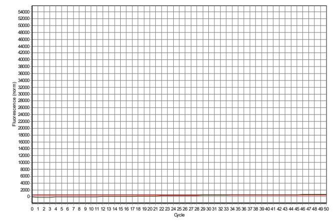
121

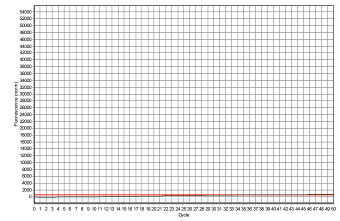
122

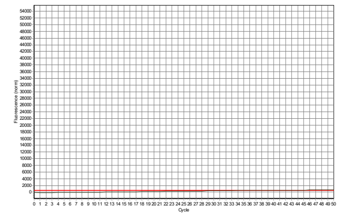
123

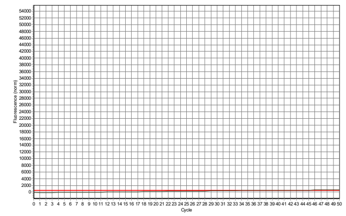
124

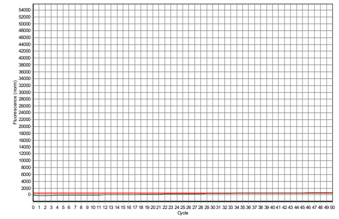
125

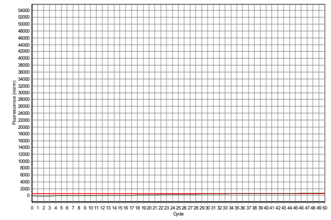
126

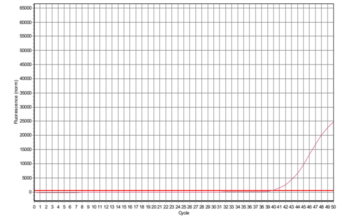
127

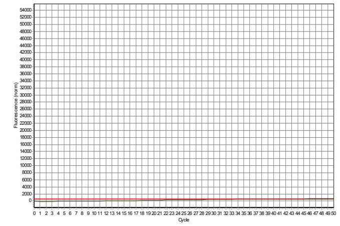
128

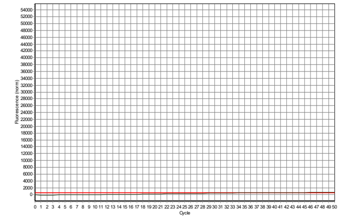
129

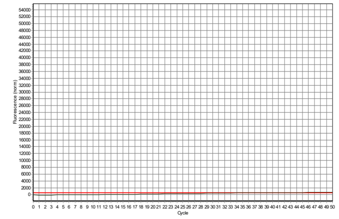
130

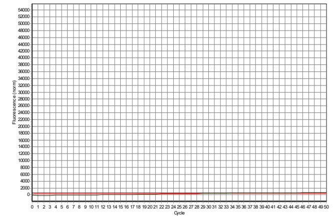
131

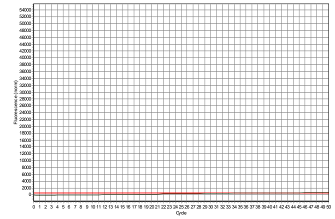
132

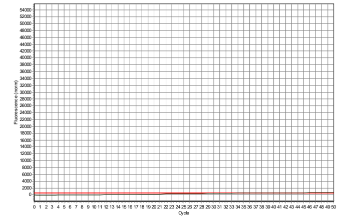
133

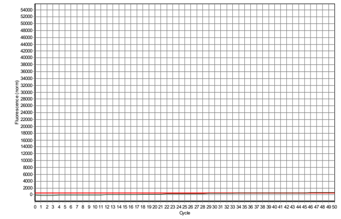
134

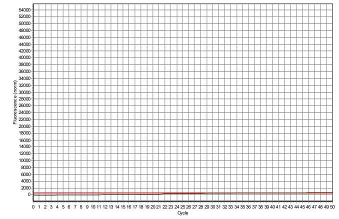
135

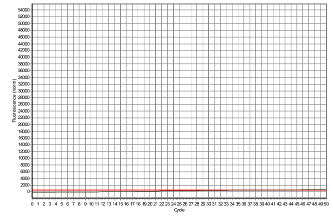
136

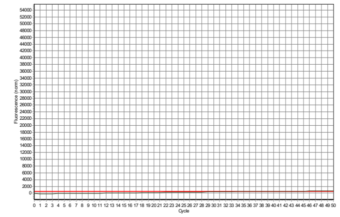
137

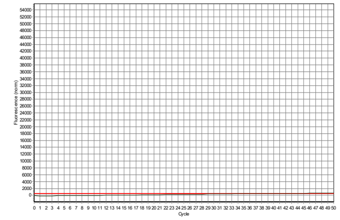
138

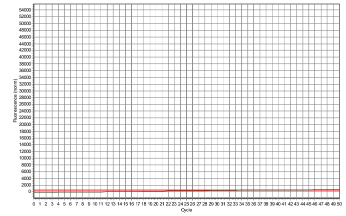
139

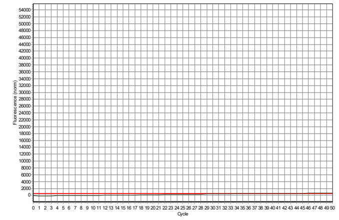
140

CCNH-c5orf30-PROSTATE-BLOOD SAMPLE 141-150

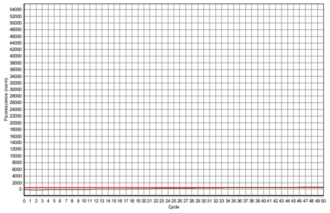

Threshold: 577 (Noiseband)
Baseline settings: automatic, Drift correction OFF

141

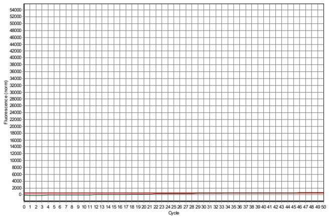

Threshold: 577 (Noiseband)
Baseline settings: automatic, Drift correction OFF

142

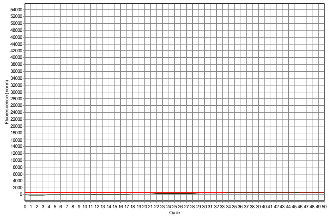

Threshold: 577 (Noiseband)
Baseline settings: automatic, Drift correction OFF

143

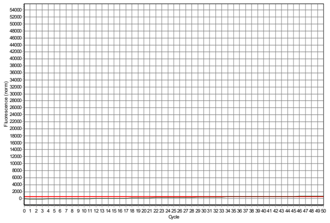

Threshold: 577 (Noiseband)
Baseline settings: automatic, Drift correction OFF

144

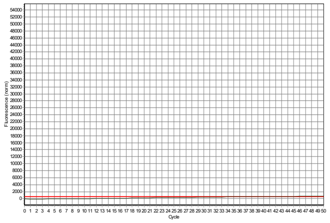

Threshold: 577 (Noiseband)
Baseline settings: automatic, Drift correction OFF

145

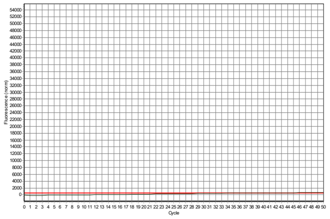

Threshold: 577 (Noiseband)
Baseline settings: automatic, Drift correction OFF

146

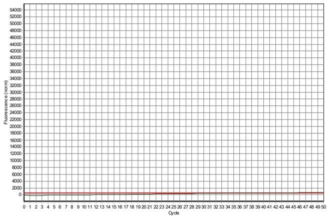

Threshold: 577 (Noiseband)
Baseline settings: automatic, Drift correction OFF

147

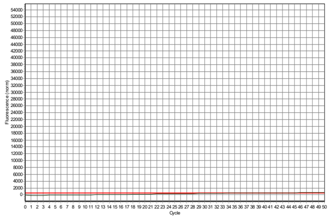

Threshold: 577 (Noiseband)
Baseline settings: automatic, Drift correction OFF

148

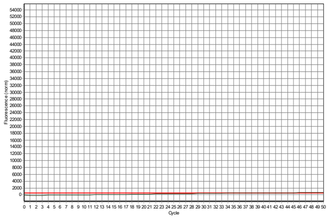

Threshold: 577 (Noiseband)
Baseline settings: automatic, Drift correction OFF

149

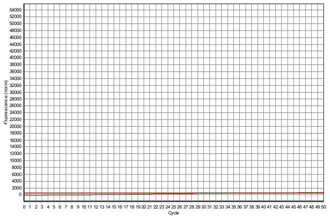

Threshold: 577 (Noiseband)
Baseline settings: automatic, Drift correction OFF

150

Supplemental figure 5

KDM4B-AC011523.2 fusion detection

KDM4B-AC011523.2 -PROSTATE-BLOOD SAMPLE 1-20

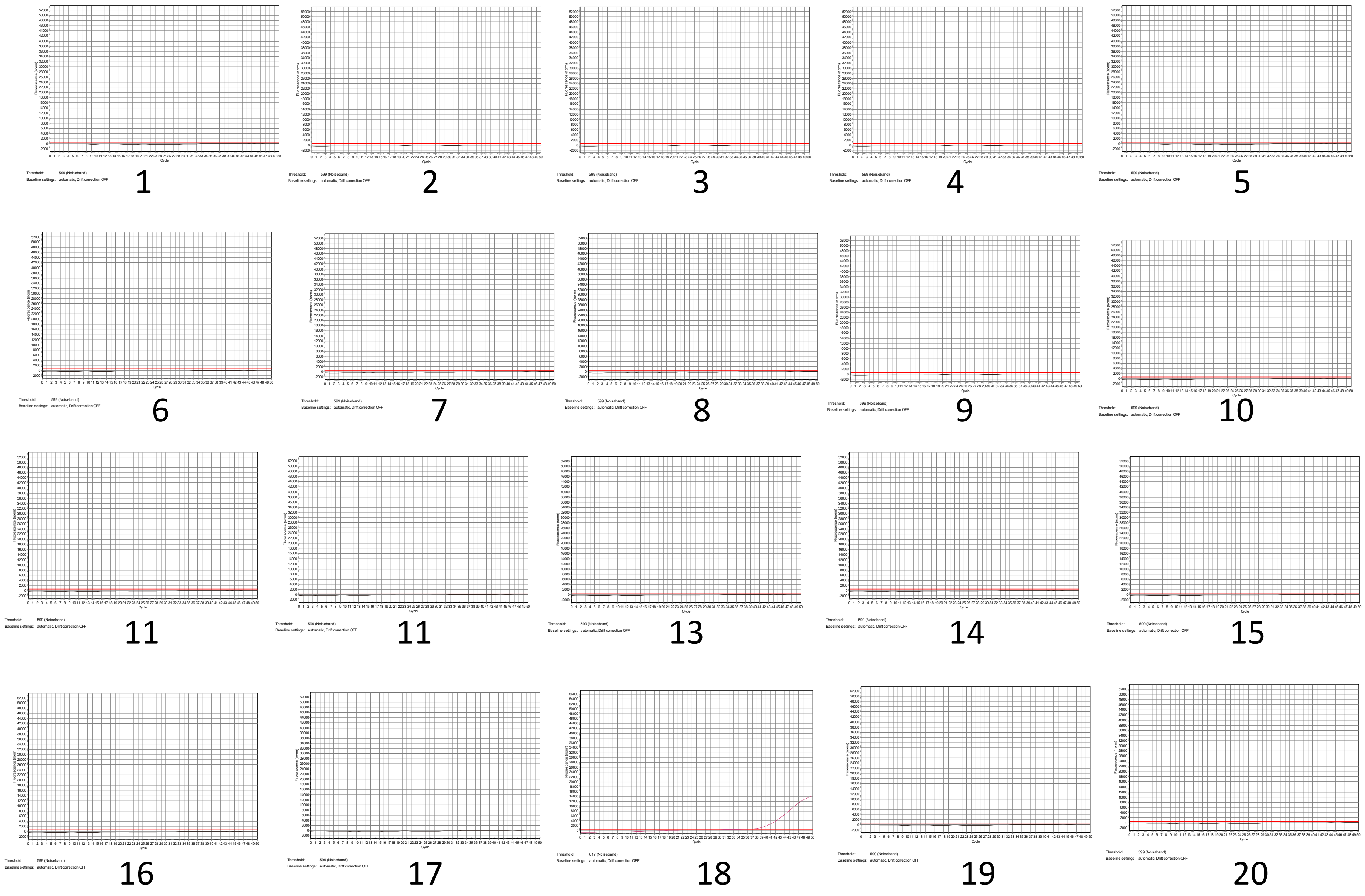

KDM4B-AC011523.2-PROSTATE-BLOOD SAMPLE 21-40

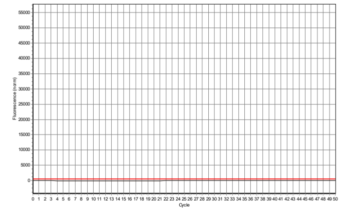
Threshold: 487 (Noiseband)
Baseline settings: automatic, Drift correction OFF

21

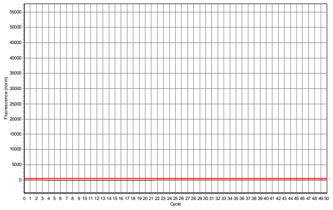
Threshold: 487 (Noiseband)
Baseline settings: automatic, Drift correction OFF

22

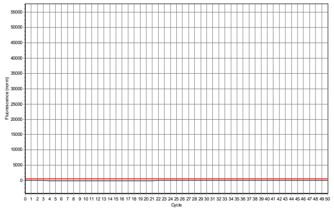
Threshold: 487 (Noiseband)
Baseline settings: automatic, Drift correction OFF

23

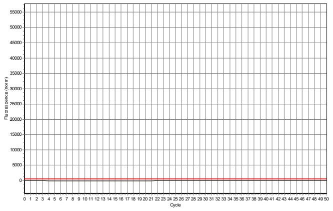
Threshold: 487 (Noiseband)
Baseline settings: automatic, Drift correction OFF

24

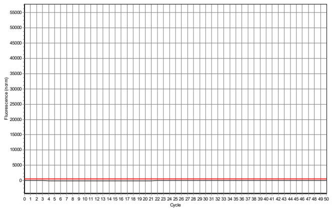
Threshold: 487 (Noiseband)
Baseline settings: automatic, Drift correction OFF

25

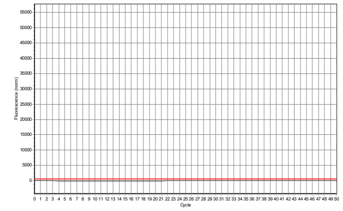
Threshold: 487 (Noiseband)
Baseline settings: automatic, Drift correction OFF

26

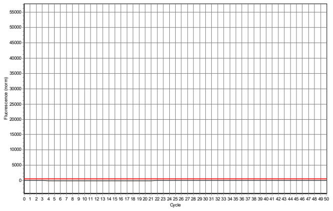
Threshold: 487 (Noiseband)
Baseline settings: automatic, Drift correction OFF

27

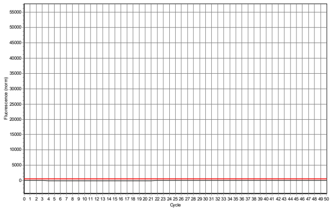
Threshold: 487 (Noiseband)
Baseline settings: automatic, Drift correction OFF

28

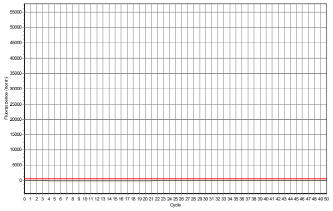
Threshold: 487 (Noiseband)
Baseline settings: automatic, Drift correction OFF

29

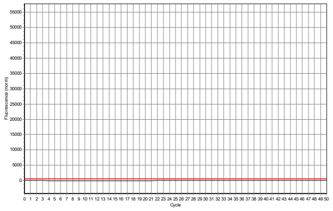
Threshold: 487 (Noiseband)
Baseline settings: automatic, Drift correction OFF

30

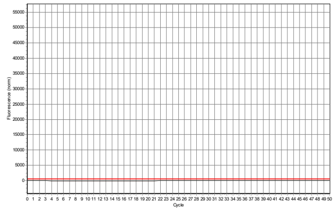
Threshold: 487 (Noiseband)
Baseline settings: automatic, Drift correction OFF

31

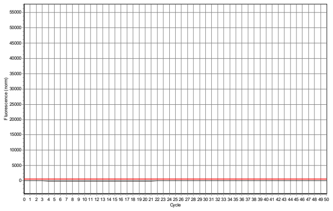
Threshold: 487 (Noiseband)
Baseline settings: automatic, Drift correction OFF

32

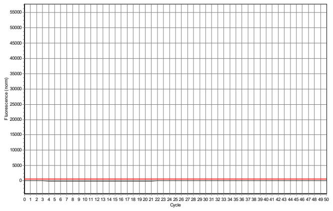
Threshold: 487 (Noiseband)
Baseline settings: automatic, Drift correction OFF

33

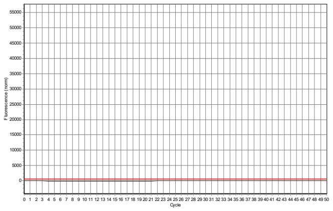
Threshold: 487 (Noiseband)
Baseline settings: automatic, Drift correction OFF

34

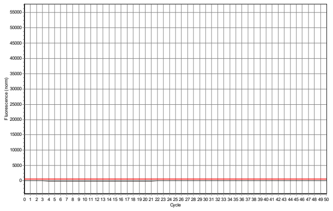
Threshold: 487 (Noiseband)
Baseline settings: automatic, Drift correction OFF

35

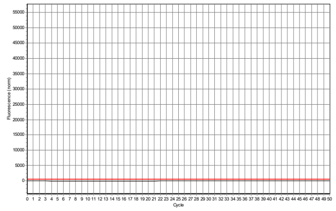
Threshold: 487 (Noiseband)
Baseline settings: automatic, Drift correction OFF

36

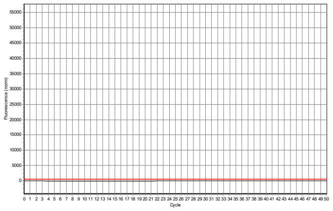
Threshold: 487 (Noiseband)
Baseline settings: automatic, Drift correction OFF

37

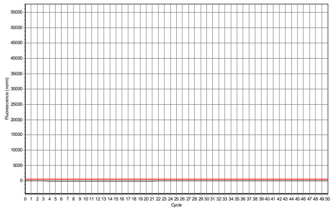
Threshold: 487 (Noiseband)
Baseline settings: automatic, Drift correction OFF

38

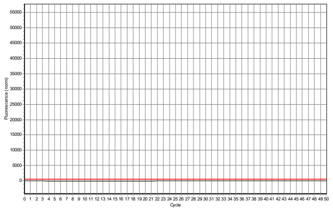
Threshold: 487 (Noiseband)
Baseline settings: automatic, Drift correction OFF

39

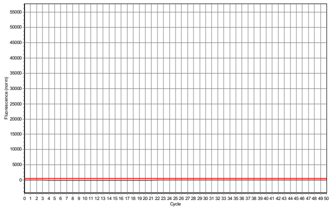
Threshold: 487 (Noiseband)
Baseline settings: automatic, Drift correction OFF

40

KDM4B-AC011523.2-PROSTATE-BLOOD SAMPLE 41-60

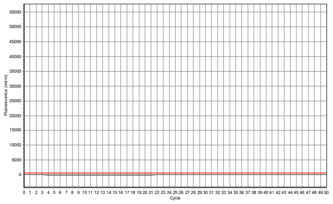

Threshold: 487 (Noiseband)
Baseline settings: automatic, Drift correction OFF

41

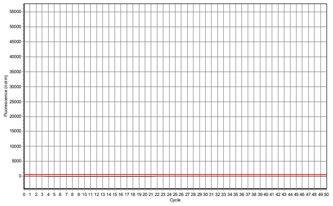

Threshold: 487 (Noiseband)
Baseline settings: automatic, Drift correction OFF

42

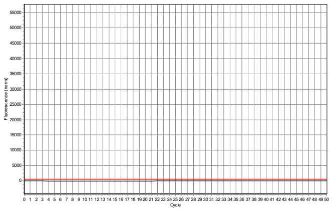

Threshold: 487 (Noiseband)
Baseline settings: automatic, Drift correction OFF

43

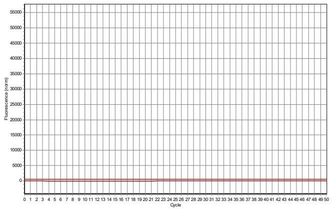

Threshold: 487 (Noiseband)
Baseline settings: automatic, Drift correction OFF

44

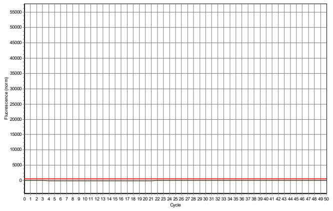

Threshold: 487 (Noiseband)
Baseline settings: automatic, Drift correction OFF

45

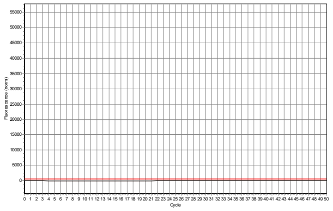

Threshold: 487 (Noiseband)
Baseline settings: automatic, Drift correction OFF

46

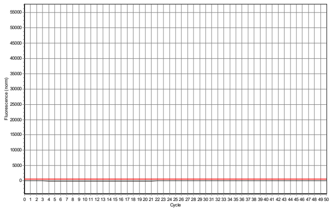

Threshold: 487 (Noiseband)
Baseline settings: automatic, Drift correction OFF

47

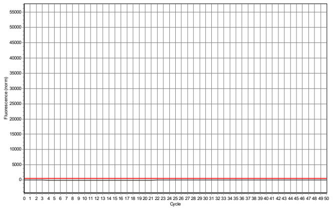

Threshold: 487 (Noiseband)
Baseline settings: automatic, Drift correction OFF

47

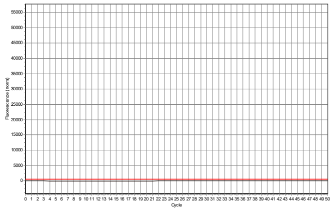

Threshold: 487 (Noiseband)
Baseline settings: automatic, Drift correction OFF

49

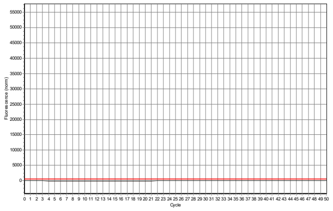

Threshold: 487 (Noiseband)
Baseline settings: automatic, Drift correction OFF

50

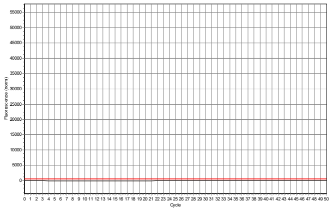

Threshold: 487 (Noiseband)
Baseline settings: automatic, Drift correction OFF

51

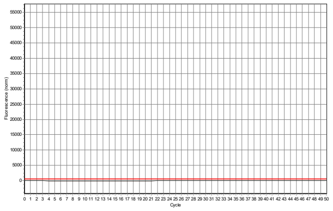

Threshold: 487 (Noiseband)
Baseline settings: automatic, Drift correction OFF

52

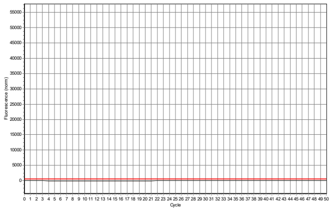

Threshold: 487 (Noiseband)
Baseline settings: automatic, Drift correction OFF

53

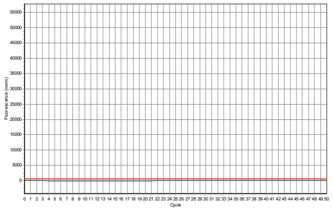

Threshold: 487 (Noiseband)
Baseline settings: automatic, Drift correction OFF

54

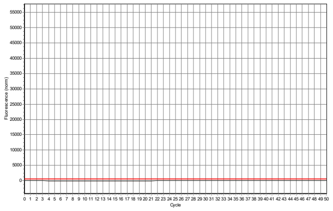

Threshold: 487 (Noiseband)
Baseline settings: automatic, Drift correction OFF

55

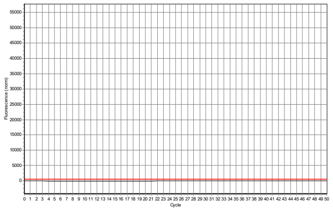

Threshold: 487 (Noiseband)
Baseline settings: automatic, Drift correction OFF

56

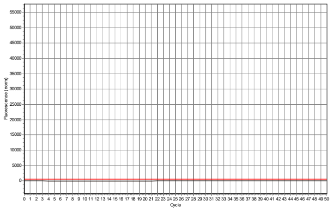

Threshold: 487 (Noiseband)
Baseline settings: automatic, Drift correction OFF

57

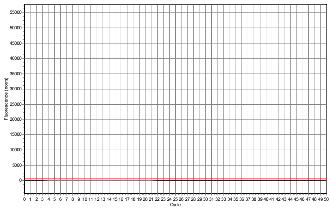

Threshold: 487 (Noiseband)
Baseline settings: automatic, Drift correction OFF

58

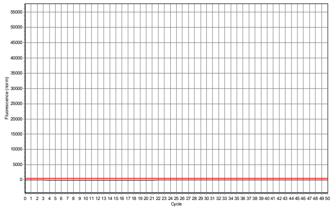

Threshold: 487 (Noiseband)
Baseline settings: automatic, Drift correction OFF

59

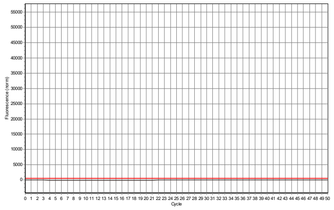

Threshold: 487 (Noiseband)
Baseline settings: automatic, Drift correction OFF

60

KDM4B-AC011523.2-PROSTATE-BLOOD SAMPLE 61-80

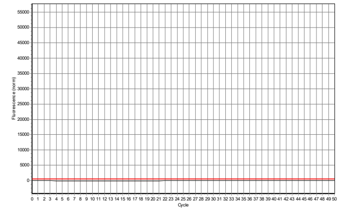

Threshold: 487 (Noiseband)
Baseline settings: automatic, Drift correction OFF

61

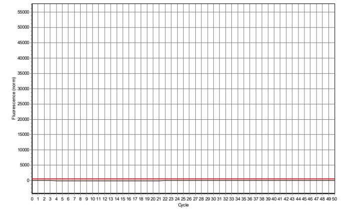

Threshold: 487 (Noiseband)
Baseline settings: automatic, Drift correction OFF

62

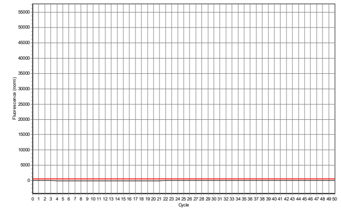

Threshold: 487 (Noiseband)
Baseline settings: automatic, Drift correction OFF

63

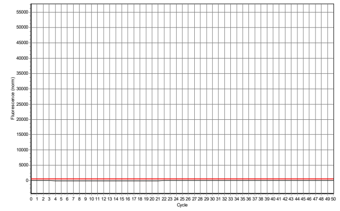

Threshold: 487 (Noiseband)
Baseline settings: automatic, Drift correction OFF

64

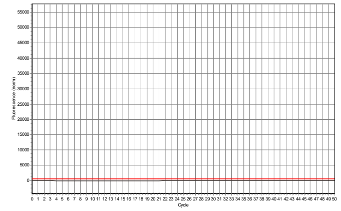

Threshold: 487 (Noiseband)
Baseline settings: automatic, Drift correction OFF

65

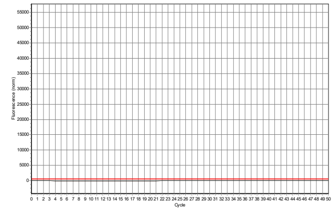

Threshold: 487 (Noiseband)
Baseline settings: automatic, Drift correction OFF

66

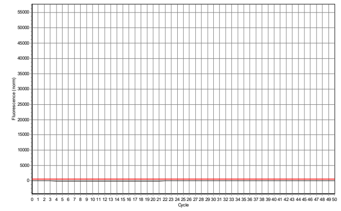

Threshold: 487 (Noiseband)
Baseline settings: automatic, Drift correction OFF

67

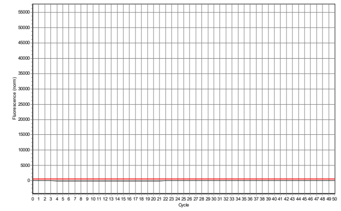

Threshold: 487 (Noiseband)
Baseline settings: automatic, Drift correction OFF

68

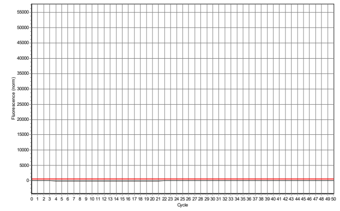

Threshold: 487 (Noiseband)
Baseline settings: automatic, Drift correction OFF

69

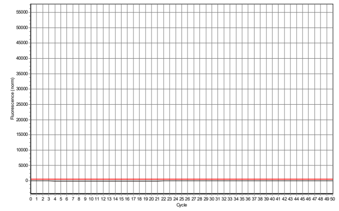

Threshold: 487 (Noiseband)
Baseline settings: automatic, Drift correction OFF

70

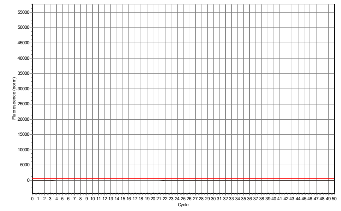

Threshold: 487 (Noiseband)
Baseline settings: automatic, Drift correction OFF

71

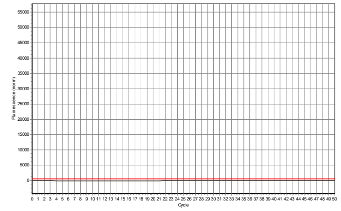

Threshold: 487 (Noiseband)
Baseline settings: automatic, Drift correction OFF

72

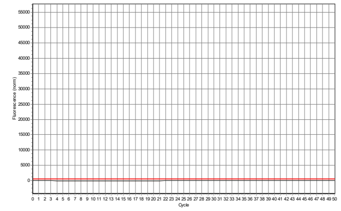

Threshold: 487 (Noiseband)
Baseline settings: automatic, Drift correction OFF

73

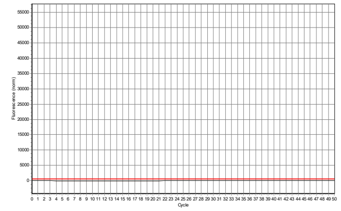

Threshold: 487 (Noiseband)
Baseline settings: automatic, Drift correction OFF

74

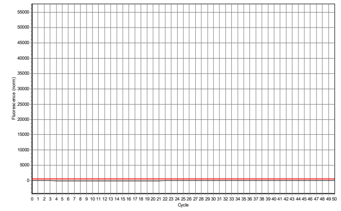

Threshold: 487 (Noiseband)
Baseline settings: automatic, Drift correction OFF

75

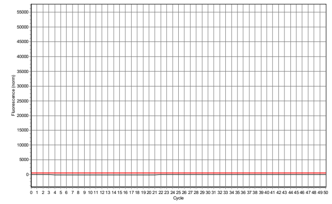

Threshold: 487 (Noiseband)
Baseline settings: automatic, Drift correction OFF

76

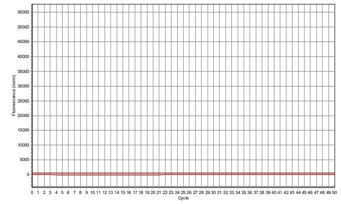

Threshold: 487 (Noiseband)
Baseline settings: automatic, Drift correction OFF

77

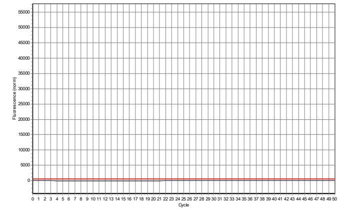

Threshold: 487 (Noiseband)
Baseline settings: automatic, Drift correction OFF

78

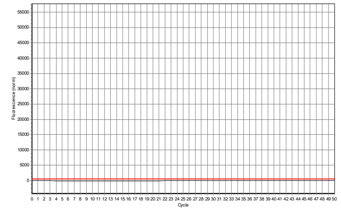

Threshold: 487 (Noiseband)
Baseline settings: automatic, Drift correction OFF

79

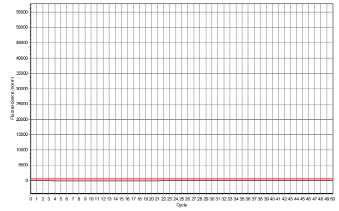

Threshold: 487 (Noiseband)
Baseline settings: automatic, Drift correction OFF

80

KDM4B-AC011523.2-PROSTATE-BLOOD SAMPLE 81-100

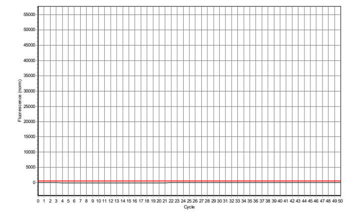

Threshold: 487 (Noiseband)
Baseline settings: automatic, Drift correction OFF

81

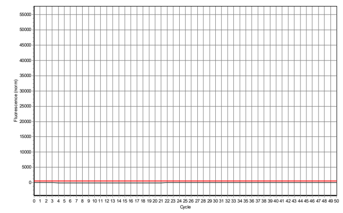

Threshold: 487 (Noiseband)
Baseline settings: automatic, Drift correction OFF

82

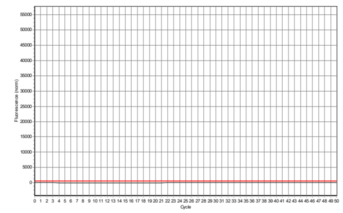

Threshold: 487 (Noiseband)
Baseline settings: automatic, Drift correction OFF

83

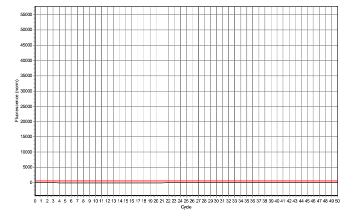

Threshold: 487 (Noiseband)
Baseline settings: automatic, Drift correction OFF

84

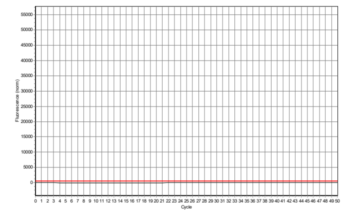

Threshold: 487 (Noiseband)
Baseline settings: automatic, Drift correction OFF

85

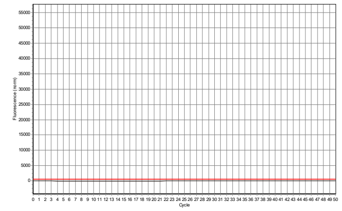

Threshold: 487 (Noiseband)
Baseline settings: automatic, Drift correction OFF

86

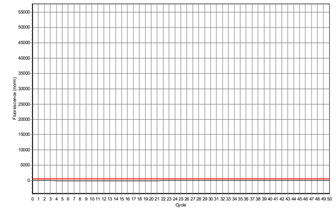

Threshold: 487 (Noiseband)
Baseline settings: automatic, Drift correction OFF

87

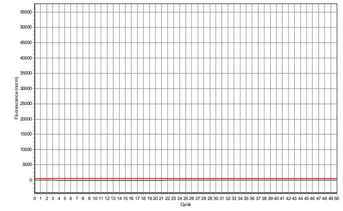

Threshold: 487 (Noiseband)
Baseline settings: automatic, Drift correction OFF

88

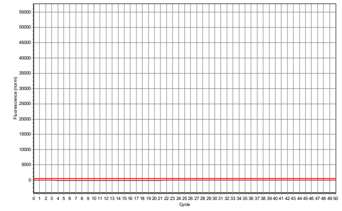

Threshold: 487 (Noiseband)
Baseline settings: automatic, Drift correction OFF

89

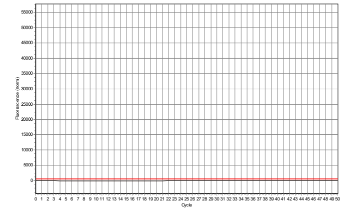

Threshold: 487 (Noiseband)
Baseline settings: automatic, Drift correction OFF

90

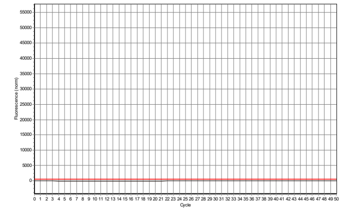

Threshold: 487 (Noiseband)
Baseline settings: automatic, Drift correction OFF

91

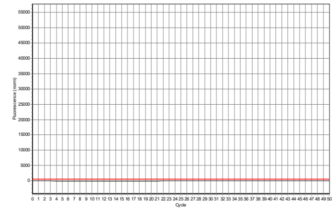

Threshold: 487 (Noiseband)
Baseline settings: automatic, Drift correction OFF

92

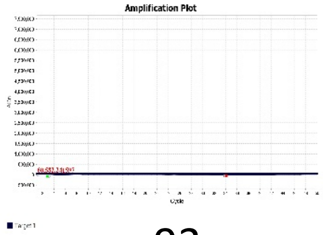

93

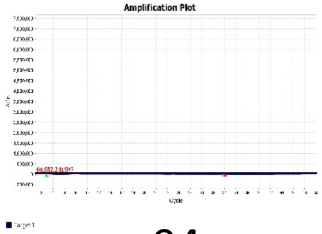

94

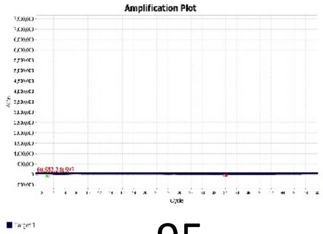

95

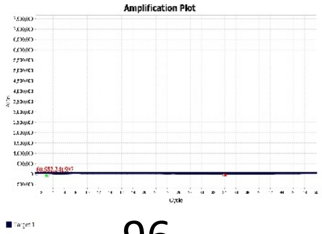

96

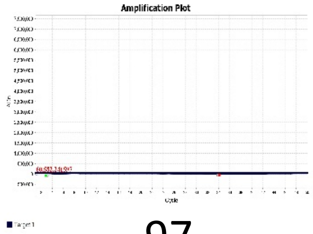

97

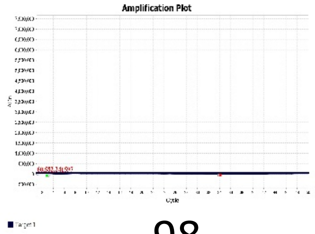

98

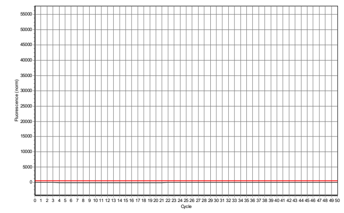

Threshold: 487 (Noiseband)
Baseline settings: automatic, Drift correction OFF

99

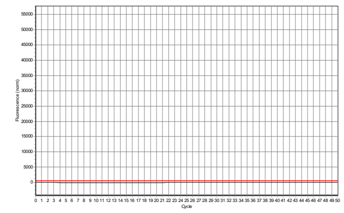

Threshold: 487 (Noiseband)
Baseline settings: automatic, Drift correction OFF

100

KDM4B-AC011523.2-PROSTATE-BLOOD SAMPLE 101-120

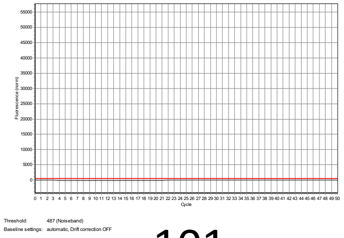
101

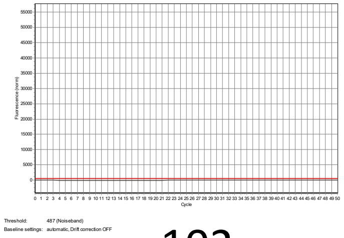
102

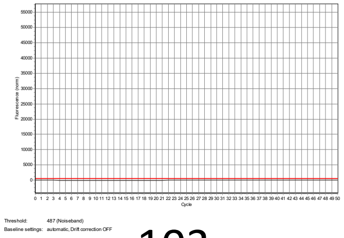
103

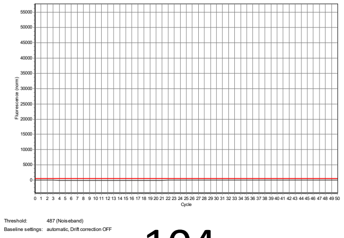
104

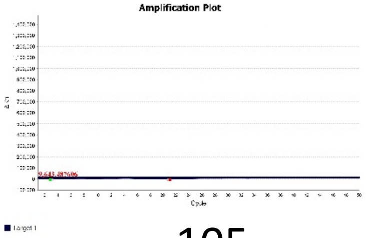

105

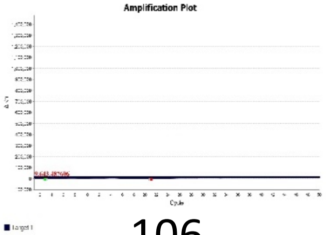

106

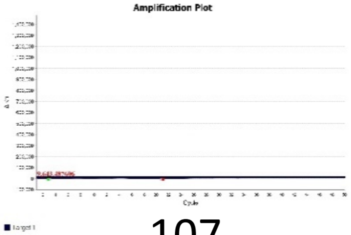

107

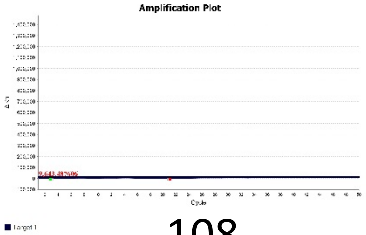

108

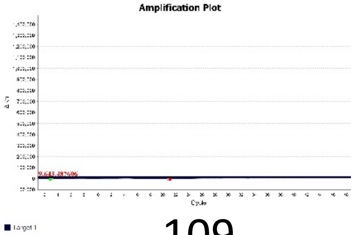

109

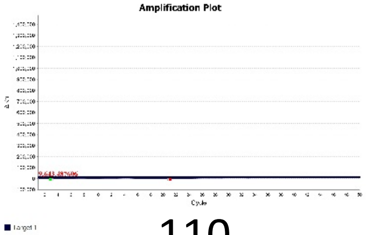

110

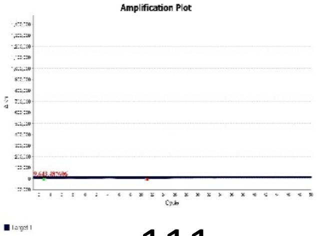

111

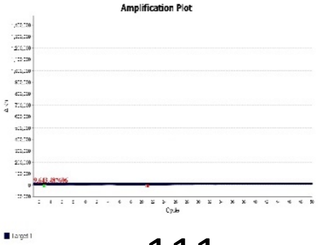

111

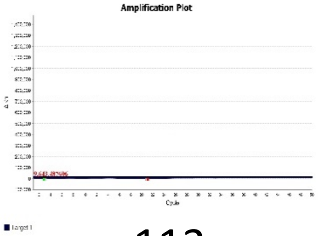

113

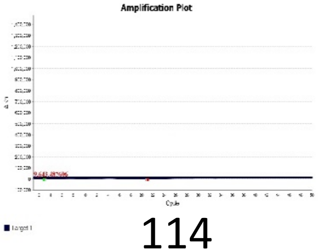

114

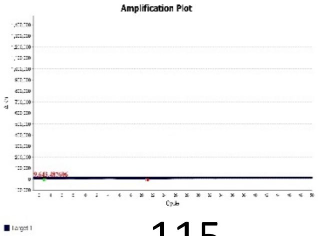

115

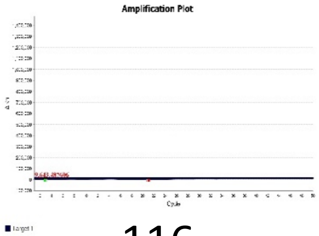

116

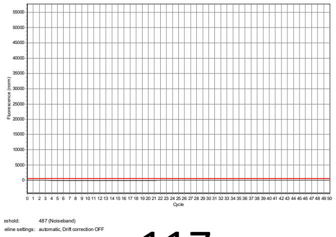
117

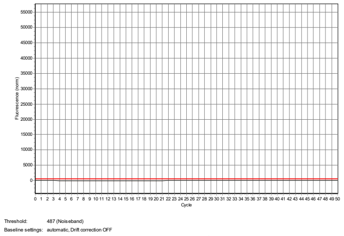
118

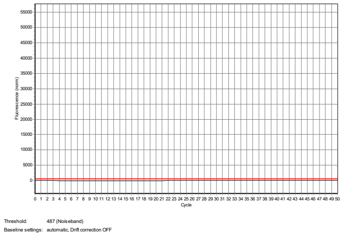
119

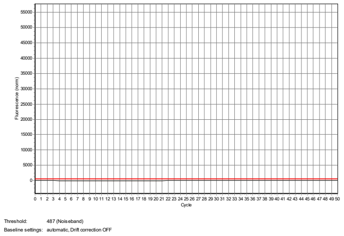
120

KDM4B-AC011523.2-PROSTATE-BLOOD SAMPLE 121-140

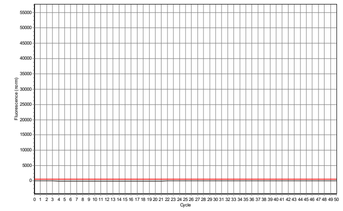

Threshold: 487 (Noiseband)
Baseline settings: automatic, Drift correction OFF

121

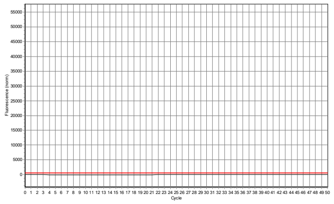

Threshold: 487 (Noiseband)
Baseline settings: automatic, Drift correction OFF

122

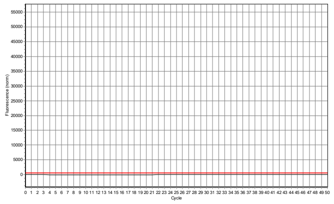

Threshold: 487 (Noiseband)
Baseline settings: automatic, Drift correction OFF

123

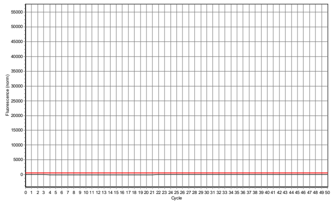

Threshold: 487 (Noiseband)
Baseline settings: automatic, Drift correction OFF

124

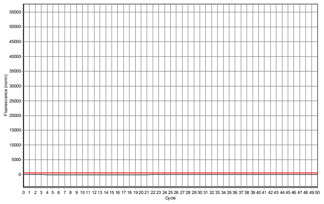

Threshold: 487 (Noiseband)
Baseline settings: automatic, Drift correction OFF

125

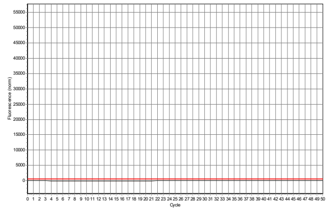

Threshold: 487 (Noiseband)
Baseline settings: automatic, Drift correction OFF

126

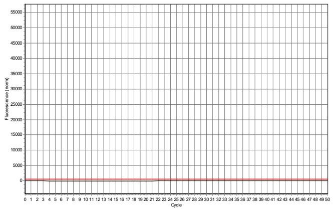

Threshold: 487 (Noiseband)
Baseline settings: automatic, Drift correction OFF

127

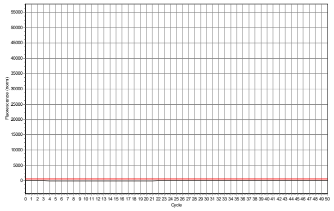

Threshold: 487 (Noiseband)
Baseline settings: automatic, Drift correction OFF

128

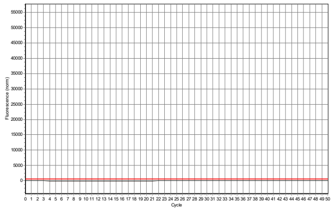

Threshold: 487 (Noiseband)
Baseline settings: automatic, Drift correction OFF

129

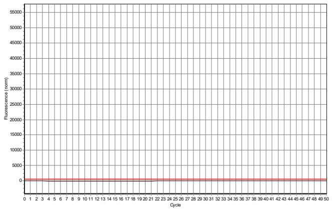

Threshold: 487 (Noiseband)
Baseline settings: automatic, Drift correction OFF

130

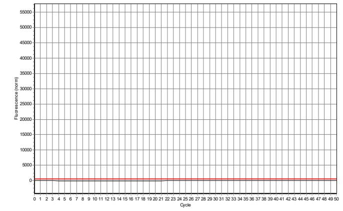

Threshold: 487 (Noiseband)
Baseline settings: automatic, Drift correction OFF

131

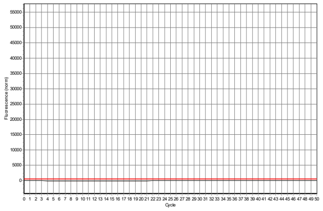

Threshold: 487 (Noiseband)
Baseline settings: automatic, Drift correction OFF

132

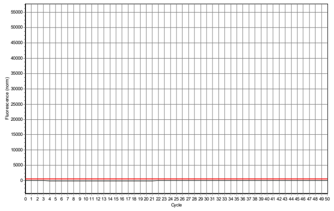

Threshold: 487 (Noiseband)
Baseline settings: automatic, Drift correction OFF

133

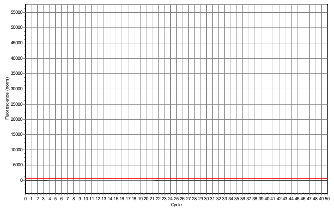

Threshold: 487 (Noiseband)
Baseline settings: automatic, Drift correction OFF

134

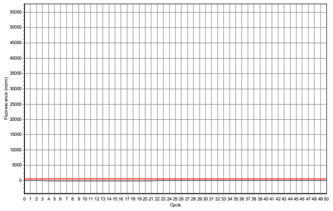

Threshold: 487 (Noiseband)
Baseline settings: automatic, Drift correction OFF

135

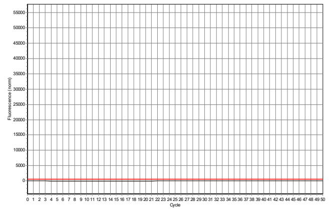

Threshold: 487 (Noiseband)
Baseline settings: automatic, Drift correction OFF

136

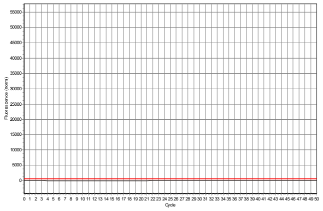

Threshold: 487 (Noiseband)
Baseline settings: automatic, Drift correction OFF

137

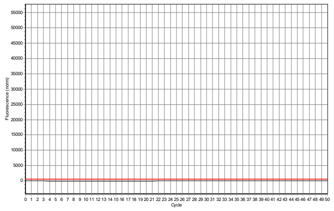

Threshold: 487 (Noiseband)
Baseline settings: automatic, Drift correction OFF

138

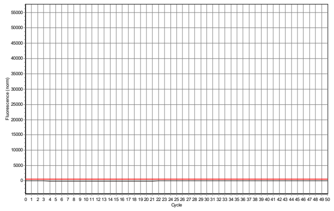

Threshold: 487 (Noiseband)
Baseline settings: automatic, Drift correction OFF

139

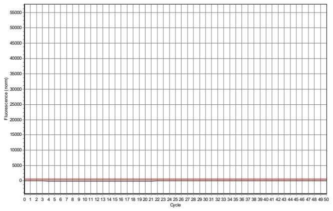

Threshold: 487 (Noiseband)
Baseline settings: automatic, Drift correction OFF

140

KDM4B-AC011523.2-PROSTATE-BLOOD SAMPLE 141-150

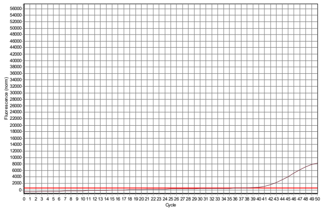

Threshold: 617 (Noiseband)
Baseline settings: automatic, Drift correction OFF

141

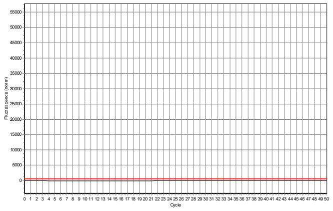

Threshold: 487 (Noiseband)
Baseline settings: automatic, Drift correction OFF

142

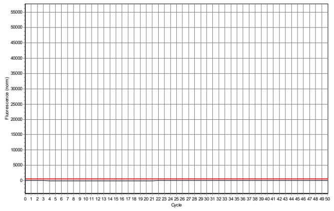

Threshold: 487 (Noiseband)
Baseline settings: automatic, Drift correction OFF

143

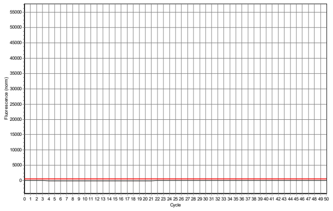

Threshold: 487 (Noiseband)
Baseline settings: automatic, Drift correction OFF

144

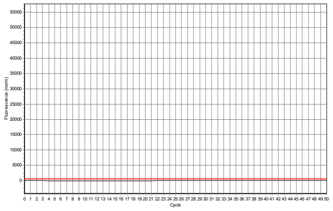

Threshold: 487 (Noiseband)
Baseline settings: automatic, Drift correction OFF

145

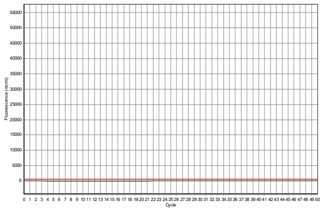

Threshold: 487 (Noiseband)
Baseline settings: automatic, Drift correction OFF

146

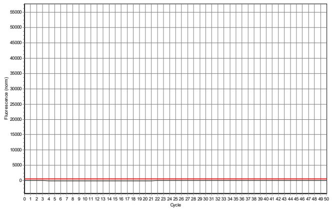

Threshold: 487 (Noiseband)
Baseline settings: automatic, Drift correction OFF

147

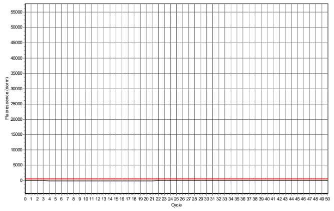

Threshold: 487 (Noiseband)
Baseline settings: automatic, Drift correction OFF

148

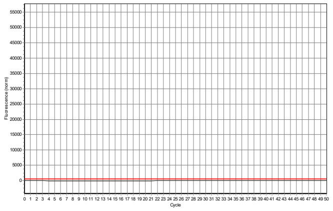

Threshold: 487 (Noiseband)
Baseline settings: automatic, Drift correction OFF

149

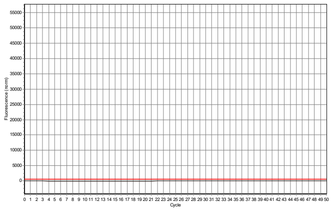

Threshold: 487 (Noiseband)
Baseline settings: automatic, Drift correction OFF

150

Supplemental figure 6

SLC45A2-AMACR fusion detection

SLC45A2-AMACR-PROSTATE-BLOOD SAMPLE 1-20

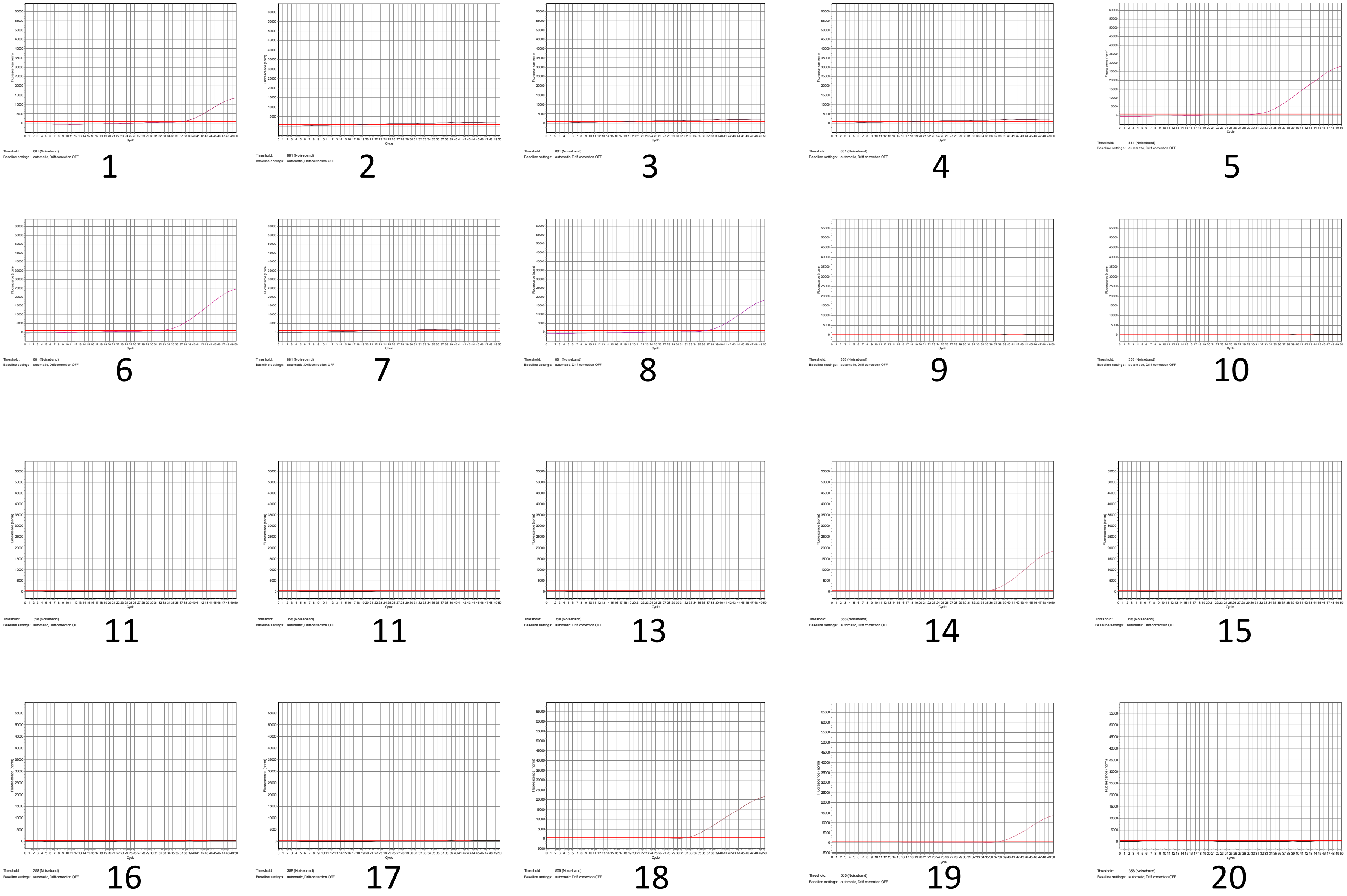

SLC45A2-AMACR-PROSTATE-BLOOD SAMPLE 21-40

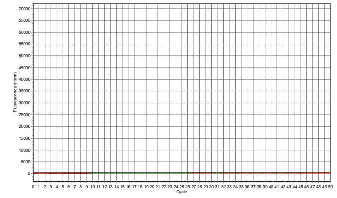

Threshold: 405 (Noiseband)
Baseline settings: automatic, Drift correction OFF

21

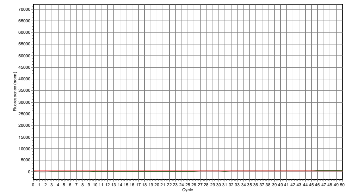

Threshold: 405 (Noiseband)
Baseline settings: automatic, Drift correction OFF

22

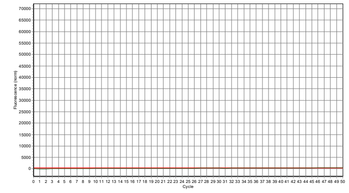

Threshold: 405 (Noiseband)
Baseline settings: automatic, Drift correction OFF

23

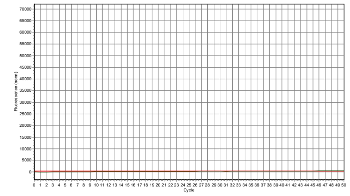

Threshold: 405 (Noiseband)
Baseline settings: automatic, Drift correction OFF

24

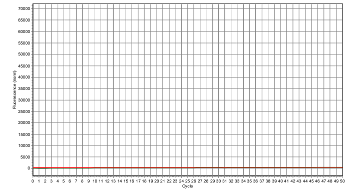

Threshold: 405 (Noiseband)
Baseline settings: automatic, Drift correction OFF

25

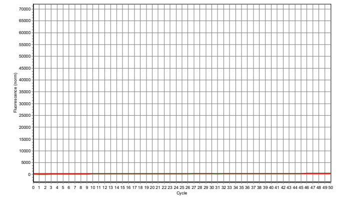

Threshold: 405 (Noiseband)
Baseline settings: automatic, Drift correction OFF

26

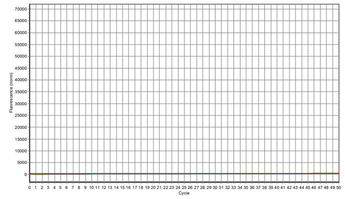

Threshold: 405 (Noiseband)
Baseline settings: automatic, Drift correction OFF

27

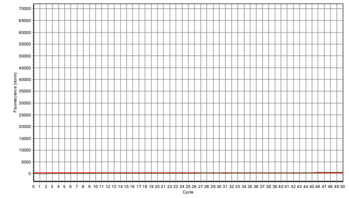

Threshold: 405 (Noiseband)
Baseline settings: automatic, Drift correction OFF

28

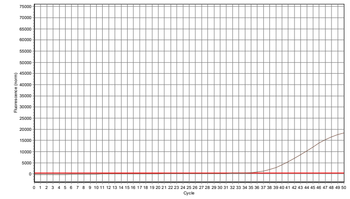

Threshold: 410 (Noiseband)
Baseline settings: automatic, Drift correction OFF

29

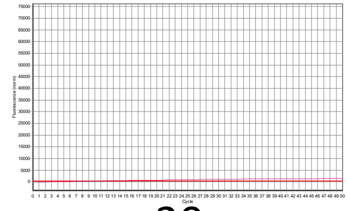

Threshold: 410 (Noiseband)
Baseline settings: automatic, Drift correction OFF

30

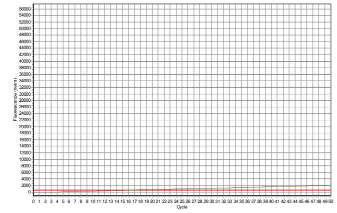

Threshold: 617 (Noiseband)
Baseline settings: automatic, Drift correction OFF

31

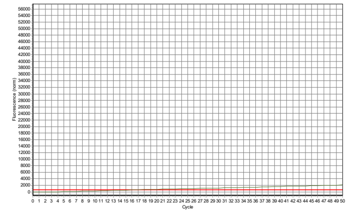

Threshold: 617 (Noiseband)
Baseline settings: automatic, Drift correction OFF

32

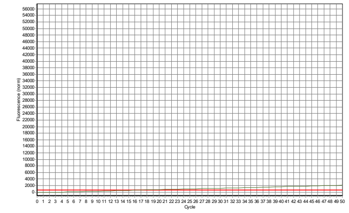

Threshold: 617 (Noiseband)
Baseline settings: automatic, Drift correction OFF

33

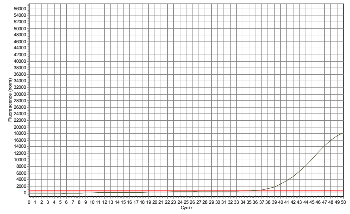

Threshold: 617 (Noiseband)
Baseline settings: automatic, Drift correction OFF

34

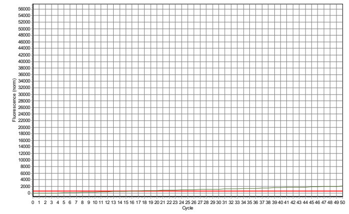

Threshold: 617 (Noiseband)
Baseline settings: automatic, Drift correction OFF

35

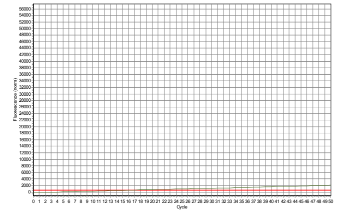

Threshold: 617 (Noiseband)
Baseline settings: automatic, Drift correction OFF

36

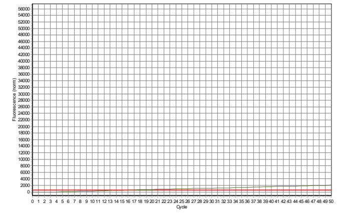

Threshold: 617 (Noiseband)
Baseline settings: automatic, Drift correction OFF

37

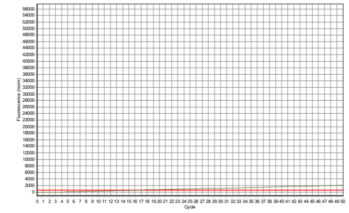

Threshold: 617 (Noiseband)
Baseline settings: automatic, Drift correction OFF

38

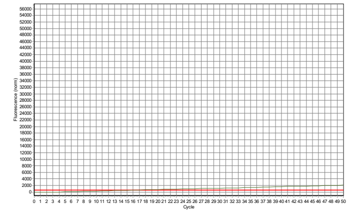

Threshold: 617 (Noiseband)
Baseline settings: automatic, Drift correction OFF

39

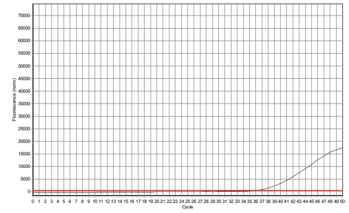

Threshold: 456 (Noiseband)
Baseline settings: automatic, Drift correction OFF

40

SLC45A2-AMACR-PROSTATE-BLOOD SAMPLE 41-60

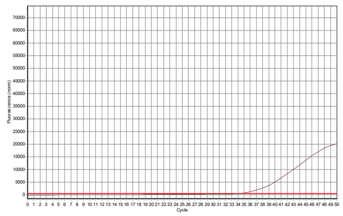

Threshold: 456 (Noiseband)
Baseline settings: automatic, Drift correction OFF

41

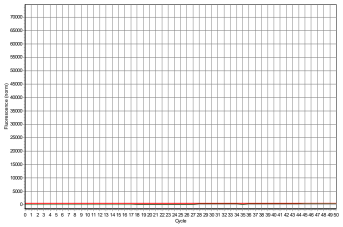

Threshold: 456 (Noiseband)
Baseline settings: automatic, Drift correction OFF

42

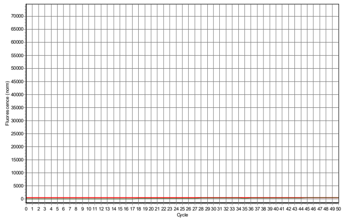

Threshold: 456 (Noiseband)
Baseline settings: automatic, Drift correction OFF

43

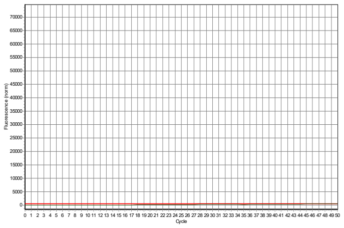

Threshold: 456 (Noiseband)
Baseline settings: automatic, Drift correction OFF

44

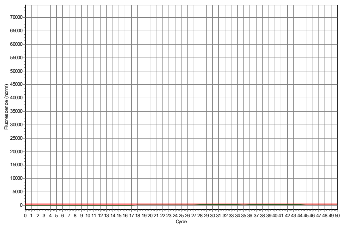

Threshold: 456 (Noiseband)
Baseline settings: automatic, Drift correction OFF

45

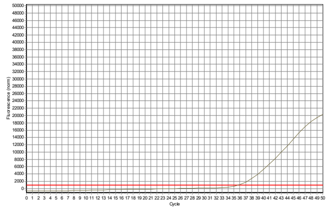

Threshold: 1016 (Noiseband)
Baseline settings: automatic, Drift correction OFF

46

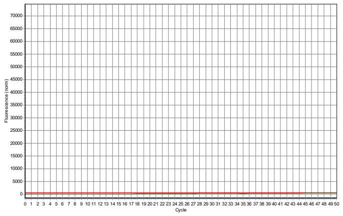

Threshold: 456 (Noiseband)
Baseline settings: automatic, Drift correction OFF

47

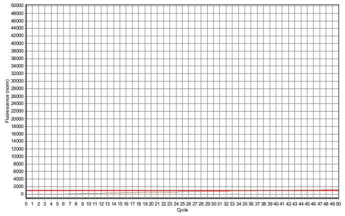

Threshold: 1016 (Noiseband)
Baseline settings: automatic, Drift correction OFF

47

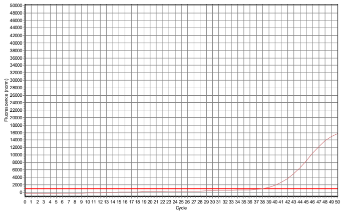

Threshold: 1016 (Noiseband)
Baseline settings: automatic, Drift correction OFF

49

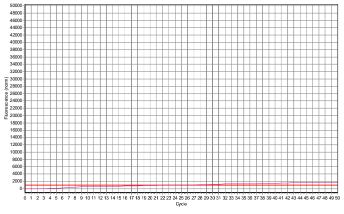

Threshold: 1016 (Noiseband)
Baseline settings: automatic, Drift correction OFF

50

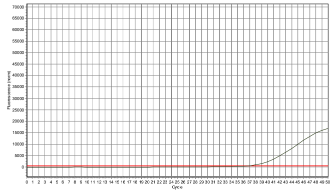

Threshold: 510 (Noiseband)
Baseline settings: automatic, Drift correction OFF

51

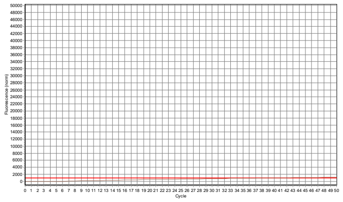

Threshold: 1016 (Noiseband)
Baseline settings: automatic, Drift correction OFF

52

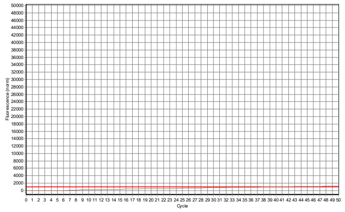

Threshold: 1016 (Noiseband)
Baseline settings: automatic, Drift correction OFF

53

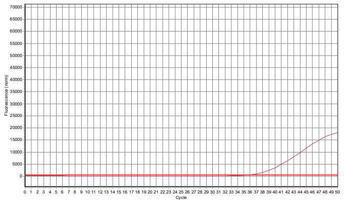

Threshold: 510 (Noiseband)
Baseline settings: automatic, Drift correction OFF

54

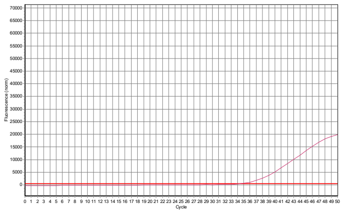

Threshold: 510 (Noiseband)
Baseline settings: automatic, Drift correction OFF

55

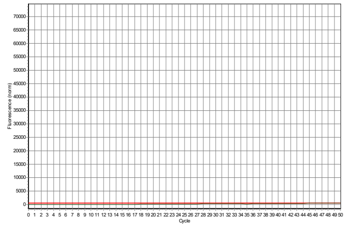

Threshold: 456 (Noiseband)
Baseline settings: automatic, Drift correction OFF

56

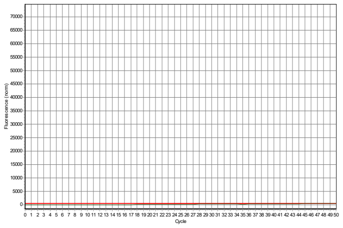

Threshold: 456 (Noiseband)
Baseline settings: automatic, Drift correction OFF

57

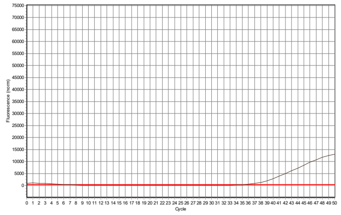

Threshold: 454 (Noiseband)
Baseline settings: automatic, Drift correction OFF

58

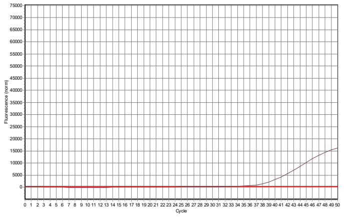

Threshold: 454 (Noiseband)
Baseline settings: automatic, Drift correction OFF

59

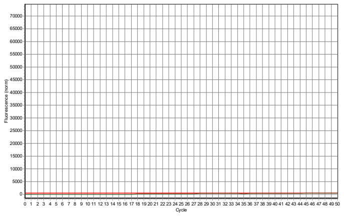

Threshold: 456 (Noiseband)
Baseline settings: automatic, Drift correction OFF

60

SLC45A2-AMACR-PROSTATE-BLOOD SAMPLE 61-80

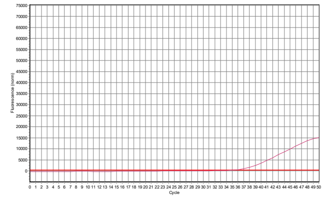
Threshold: 454 (Noiseband)
Baseline settings: automatic, Drift correction OFF

61

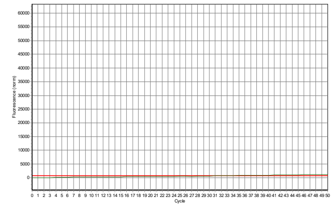
Threshold: 759 (Noiseband)
Baseline settings: automatic, Drift correction OFF

62

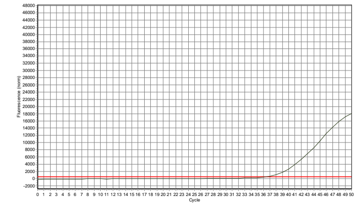
Threshold: 555 (Noiseband)
Baseline settings: automatic, Drift correction OFF

63

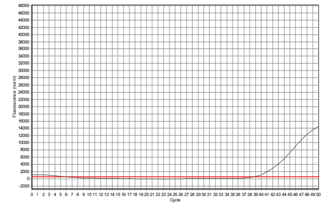
Threshold: 555 (Noiseband)
Baseline settings: automatic, Drift correction OFF

64

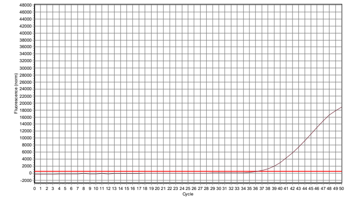
Threshold: 555 (Noiseband)
Baseline settings: automatic, Drift correction OFF

65

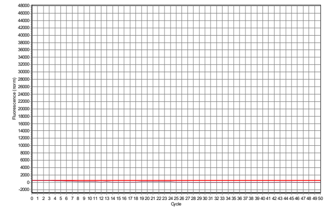
Threshold: 555 (Noiseband)
Baseline settings: automatic, Drift correction OFF

66

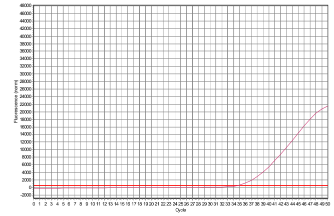
Threshold: 555 (Noiseband)
Baseline settings: automatic, Drift correction OFF

67

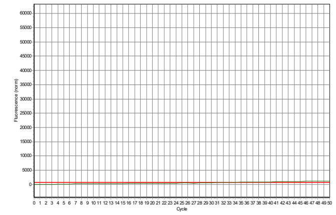
Threshold: 759 (Noiseband)
Baseline settings: automatic, Drift correction OFF

68

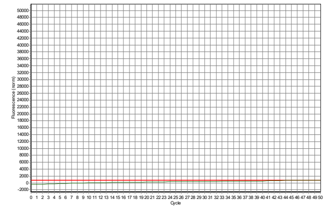
Threshold: 741 (Noiseband)
Baseline settings: automatic, Drift correction OFF

69

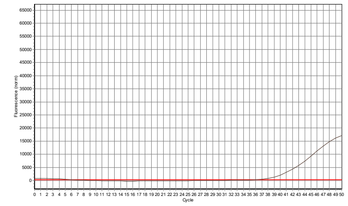
Threshold: 312 (Noiseband)
Baseline settings: automatic, Drift correction OFF

70

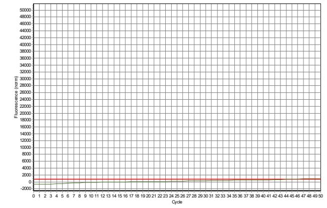
Threshold: 741 (Noiseband)
Baseline settings: automatic, Drift correction OFF

71

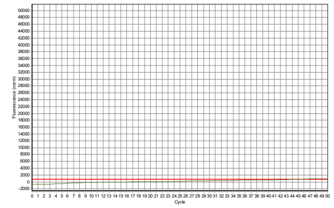
Threshold: 741 (Noiseband)
Baseline settings: automatic, Drift correction OFF

72

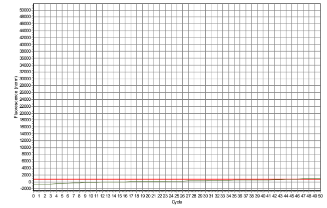
Threshold: 741 (Noiseband)
Baseline settings: automatic, Drift correction OFF

73

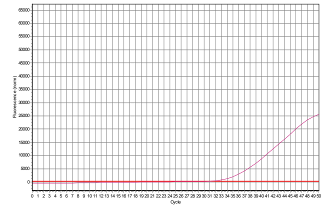
Threshold: 312 (Noiseband)
Baseline settings: automatic, Drift correction OFF

74

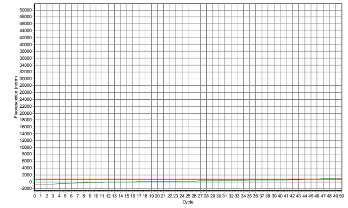
Threshold: 741 (Noiseband)
Baseline settings: automatic, Drift correction OFF

75

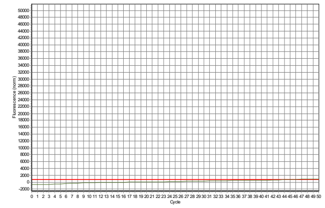
Threshold: 741 (Noiseband)
Baseline settings: automatic, Drift correction OFF

76

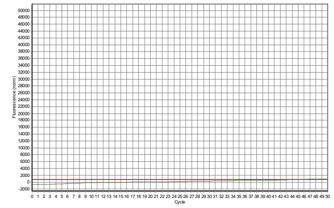
Threshold: 741 (Noiseband)
Baseline settings: automatic, Drift correction OFF

77

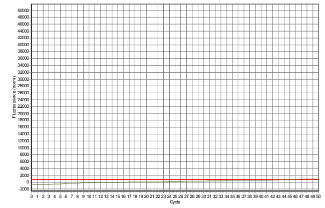
Threshold: 741 (Noiseband)
Baseline settings: automatic, Drift correction OFF

78

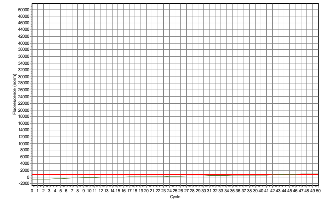
Threshold: 741 (Noiseband)
Baseline settings: automatic, Drift correction OFF

79

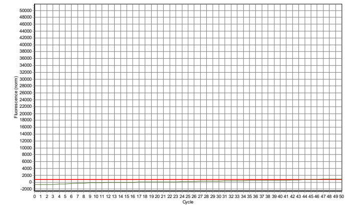
Threshold: 741 (Noiseband)
Baseline settings: automatic, Drift correction OFF

80

SLC45A2-AMACR-PROSTATE-BLOOD SAMPLE 81-100

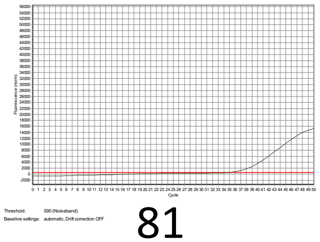
81

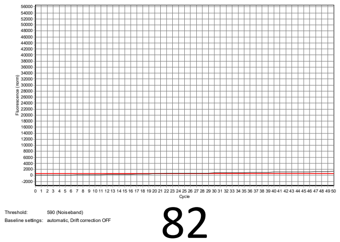
82

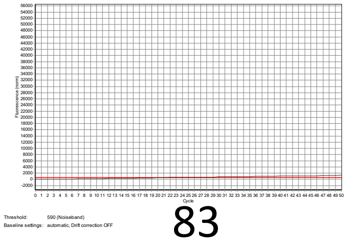
83

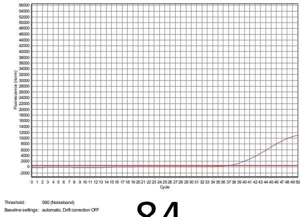
84

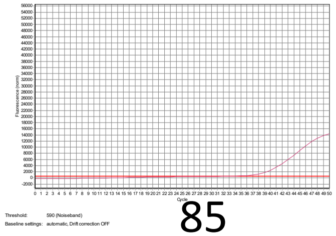
85

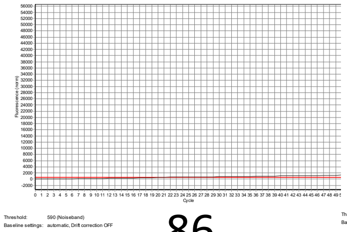
86

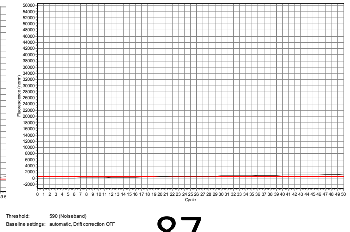
87

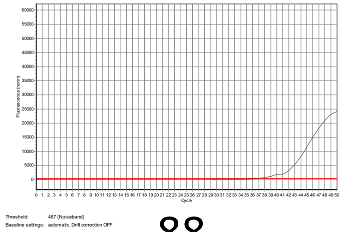
88

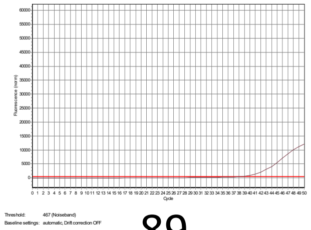
89

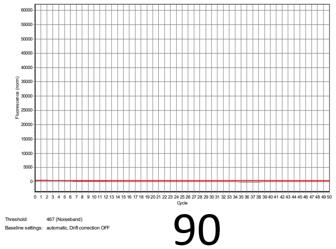
90

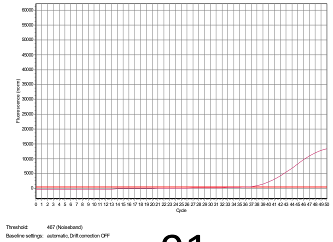
91

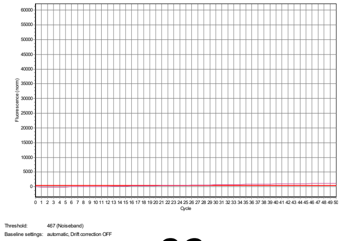
92

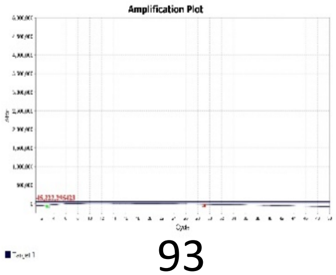

93

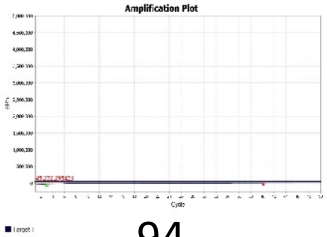

94

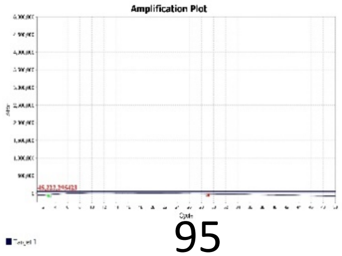

95

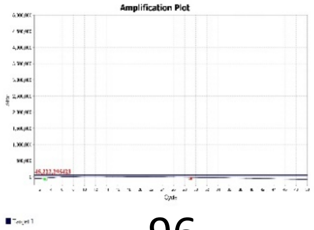

96

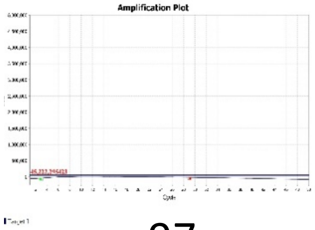

97

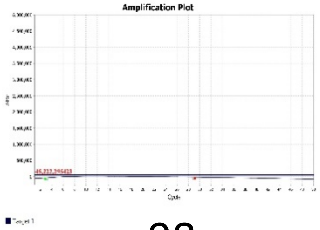

98

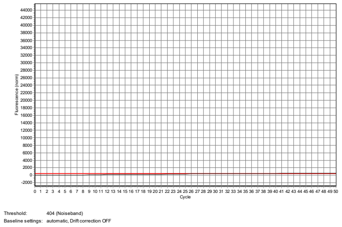
99

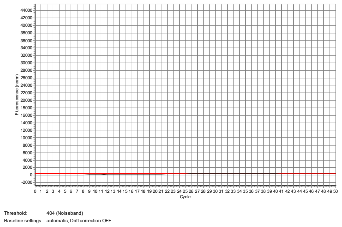
100

SLC45A2-AMACR-PROSTATE-BLOOD SAMPLE 101-120

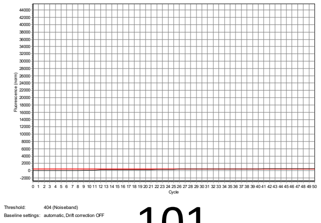
101

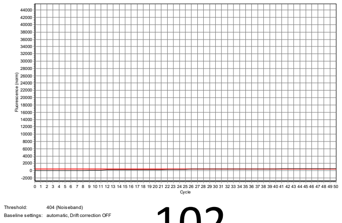
102

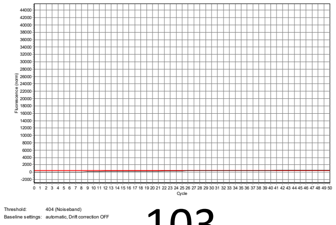
103

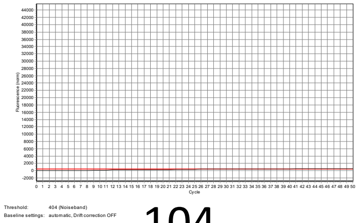
104

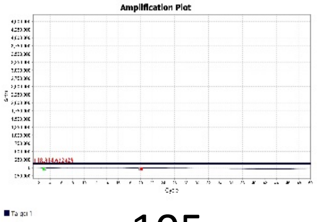

105

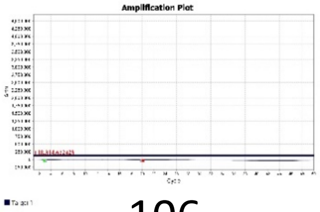

106

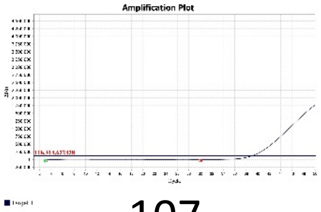

107

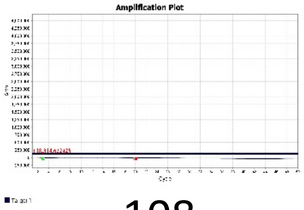

108

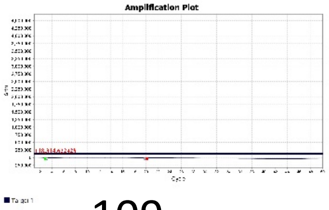

109

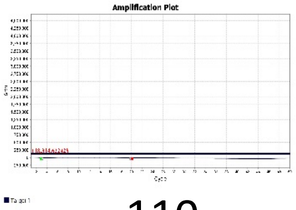

110

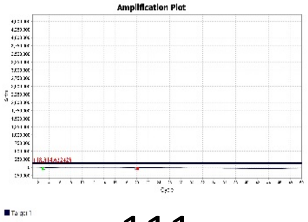

111

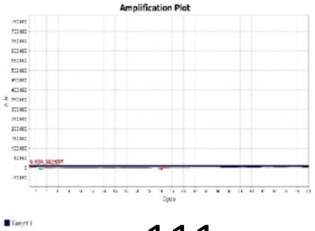

111

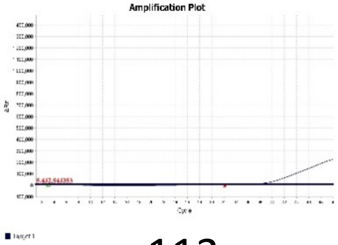

113

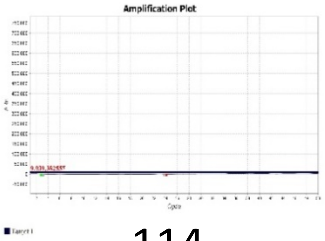

114

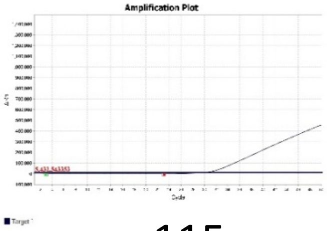

115

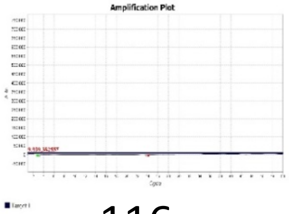

116

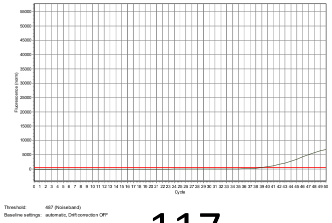
117

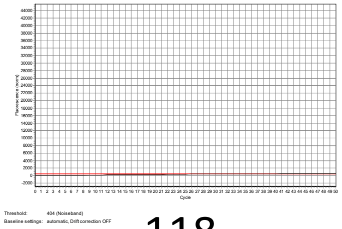
118

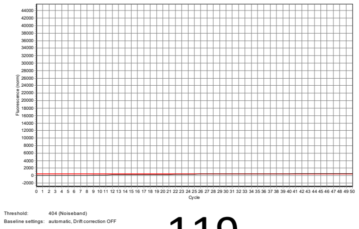
119

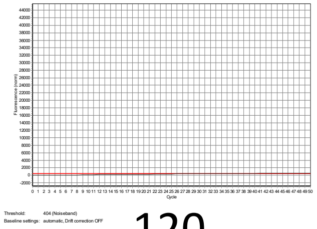
120

SLC45A2-AMACR-PROSTATE-BLOOD SAMPLE 121-140

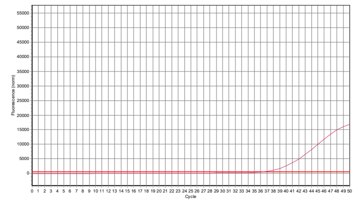
Threshold: 487 (Noiseband)
Baseline settings: automatic, Drift correction OFF

121

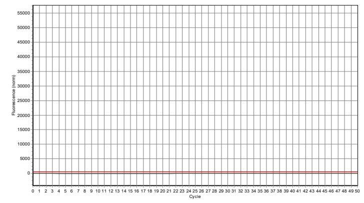
Threshold: 487 (Noiseband)
Baseline settings: automatic, Drift correction OFF

122

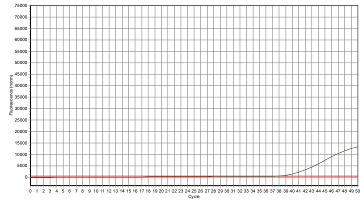
Threshold: 514 (Noiseband)
Baseline settings: automatic, Drift correction OFF

123

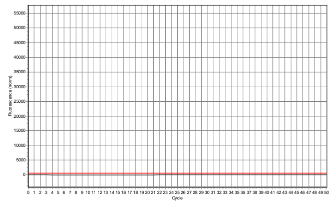
Threshold: 487 (Noiseband)
Baseline settings: automatic, Drift correction OFF

124

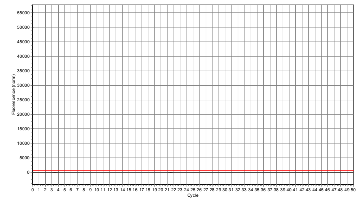
Threshold: 487 (Noiseband)
Baseline settings: automatic, Drift correction OFF

125

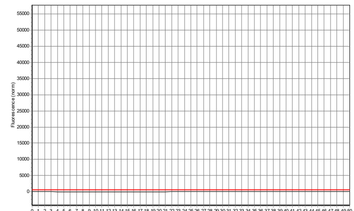
Threshold: 487 (Noiseband)
Baseline settings: automatic, Drift correction OFF

126

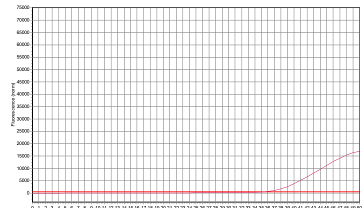
Threshold: 514 (Noiseband)
Baseline settings: automatic, Drift correction OFF

127

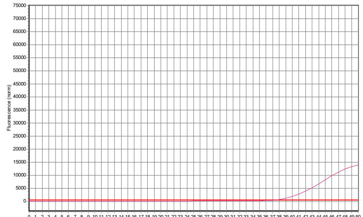
Threshold: 514 (Noiseband)
Baseline settings: automatic, Drift correction OFF

128

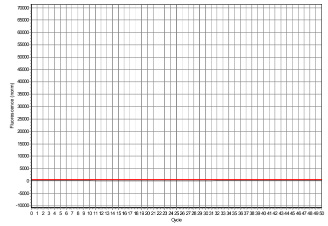
Threshold: 600 (Noiseband)
Baseline settings: automatic, Drift correction OFF

129

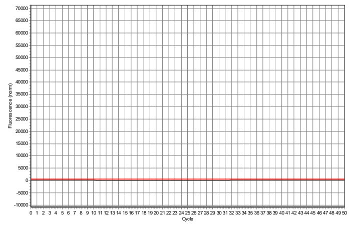
Threshold: 600 (Noiseband)
Baseline settings: automatic, Drift correction OFF

130

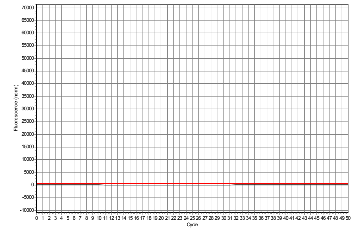
Threshold: 600 (Noiseband)
Baseline settings: automatic, Drift correction OFF

131

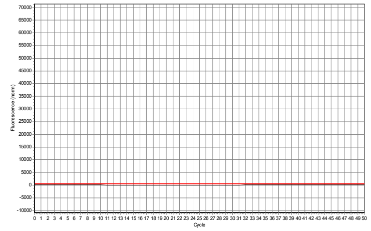
Threshold: 600 (Noiseband)
Baseline settings: automatic, Drift correction OFF

132

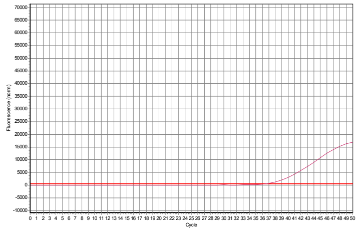
Threshold: 600 (Noiseband)
Baseline settings: automatic, Drift correction OFF

133

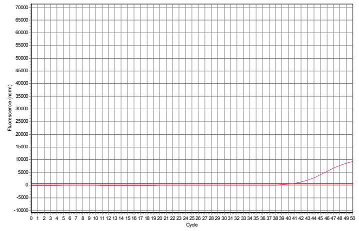
Threshold: 600 (Noiseband)
Baseline settings: automatic, Drift correction OFF

134

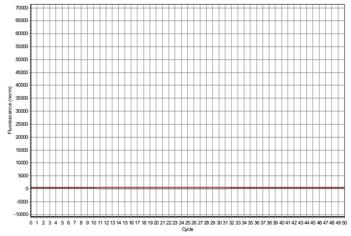
Threshold: 600 (Noiseband)
Baseline settings: automatic, Drift correction OFF

135

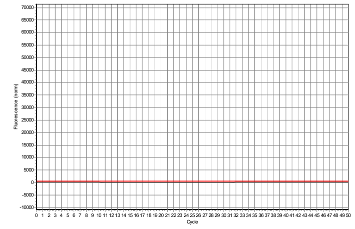
Threshold: 600 (Noiseband)
Baseline settings: automatic, Drift correction OFF

136

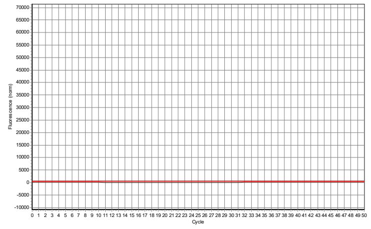
Threshold: 600 (Noiseband)
Baseline settings: automatic, Drift correction OFF

137

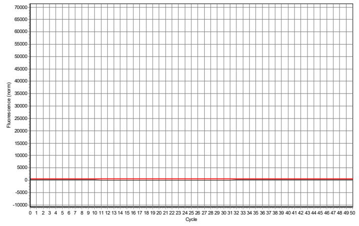
Threshold: 600 (Noiseband)
Baseline settings: automatic, Drift correction OFF

138

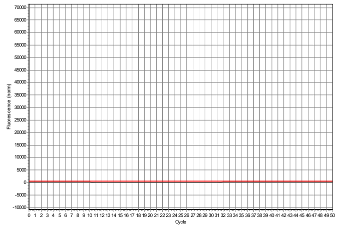
Threshold: 600 (Noiseband)
Baseline settings: automatic, Drift correction OFF

139

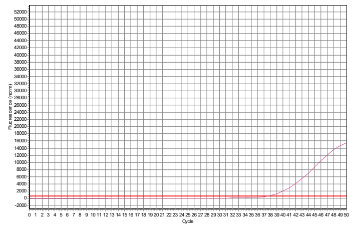
Threshold: 599 (Noiseband)
Baseline settings: automatic, Drift correction OFF

140

SLC45A2-AMACR-PROSTATE-BLOOD SAMPLE   141-150

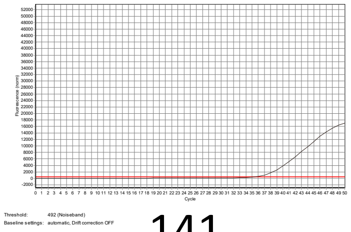

141

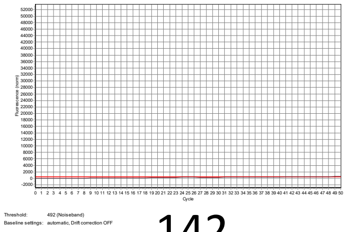

142

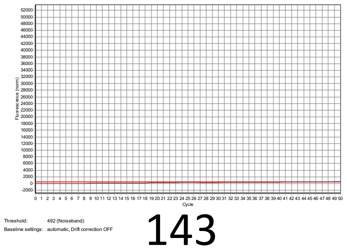

143

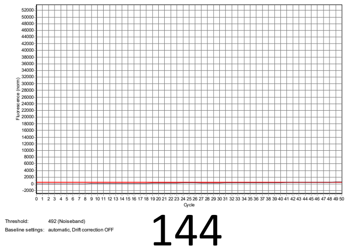

144

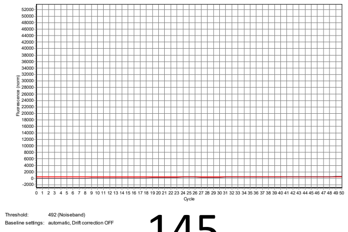

145

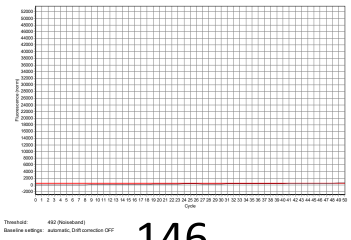

146

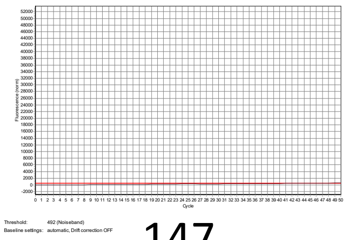

147

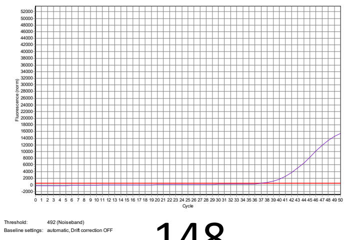

148

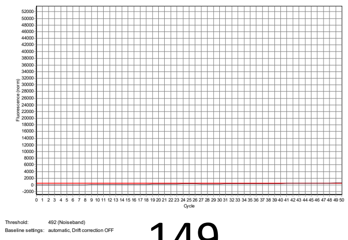

149

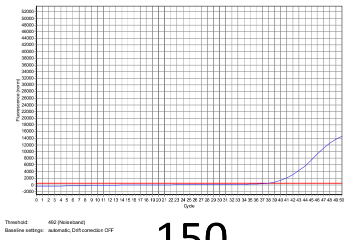

150

Supplemental figure 7

TMEM135-CCDC67 fusion detection
TMEM135-CCDC67-PROSTATE-BLOOD SAMPLE 1-20

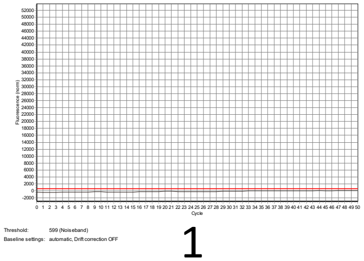

1

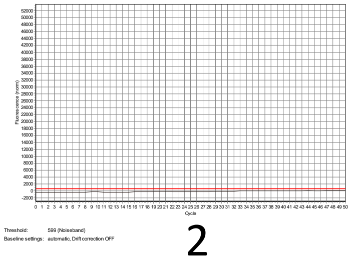

2

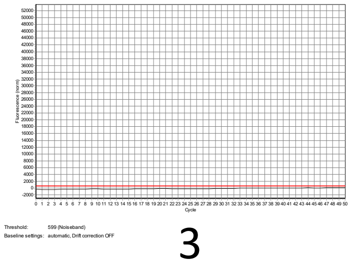

3

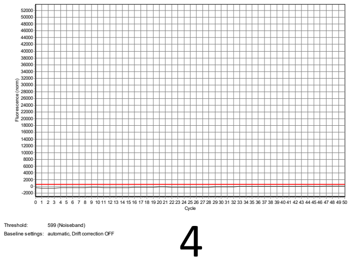

4

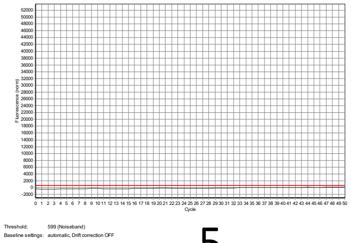

5

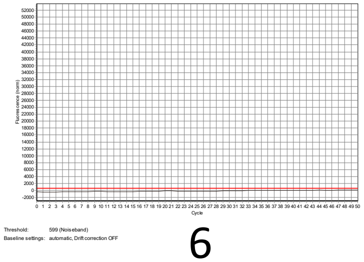

6

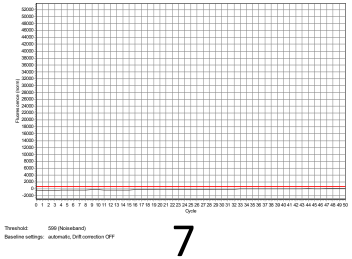

7

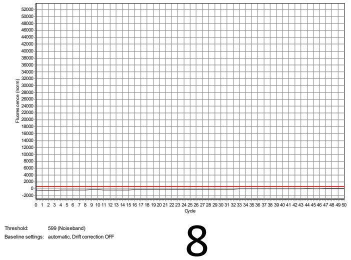

8

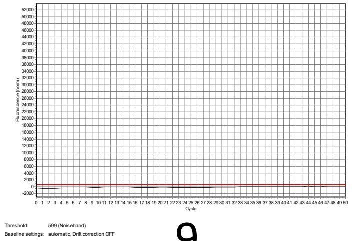

9

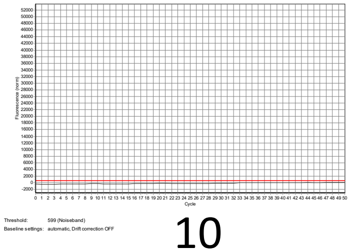

10

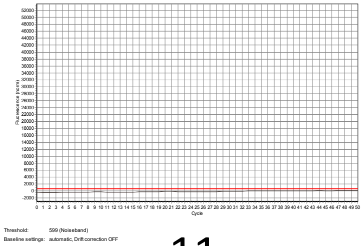

11

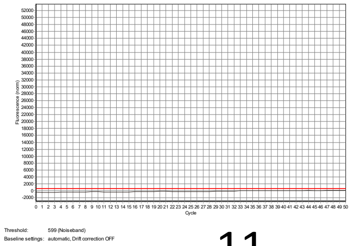

11

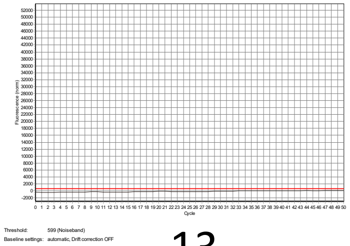

13

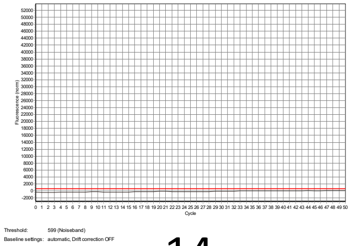

14

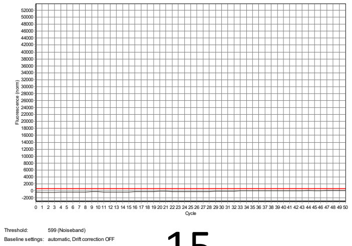

15

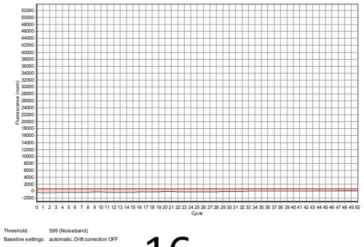

16

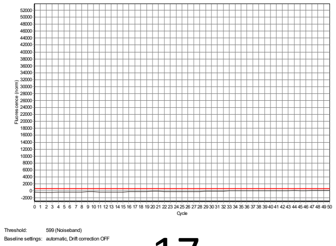

17

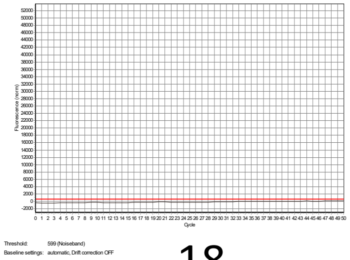

18

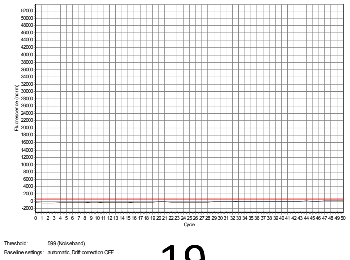

19

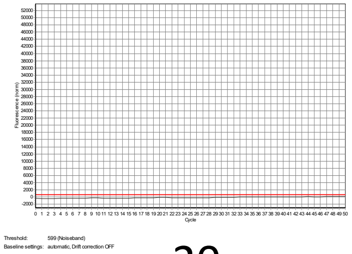

20

TMEM135-CCDC67-PROSTATE-BLOOD SAMPLE 21-40

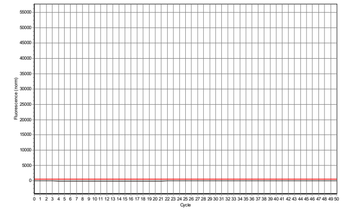
Threshold: 487 (Noiseband)
Baseline settings: automatic, Drift correction OFF

21

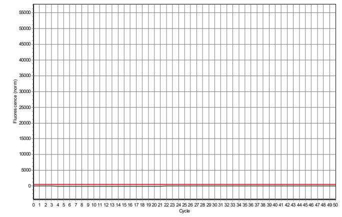
Threshold: 487 (Noiseband)
Baseline settings: automatic, Drift correction OFF

22

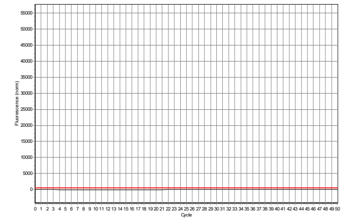
Threshold: 487 (Noiseband)
Baseline settings: automatic, Drift correction OFF

23

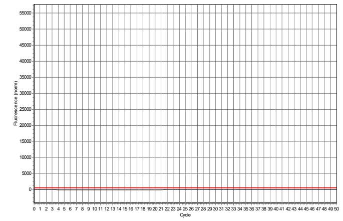
Threshold: 487 (Noiseband)
Baseline settings: automatic, Drift correction OFF

24

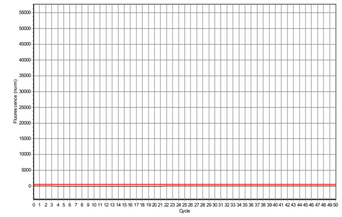
Threshold: 487 (Noiseband)
Baseline settings: automatic, Drift correction OFF

25

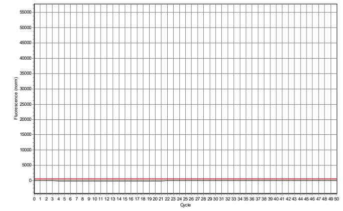
Threshold: 487 (Noiseband)
Baseline settings: automatic, Drift correction OFF

26

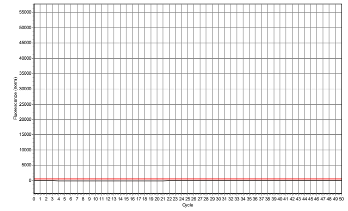
Threshold: 487 (Noiseband)
Baseline settings: automatic, Drift correction OFF

27

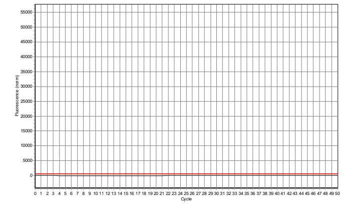
Threshold: 487 (Noiseband)
Baseline settings: automatic, Drift correction OFF

28

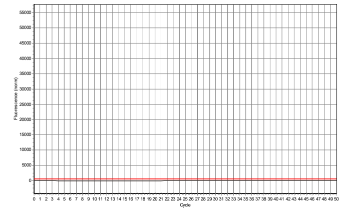
Threshold: 487 (Noiseband)
Baseline settings: automatic, Drift correction OFF

29

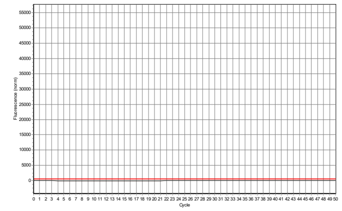
Threshold: 487 (Noiseband)
Baseline settings: automatic, Drift correction OFF

30

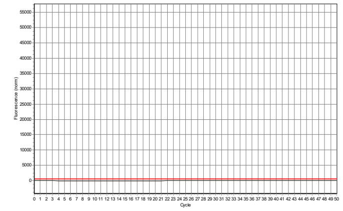
Threshold: 487 (Noiseband)
Baseline settings: automatic, Drift correction OFF

31

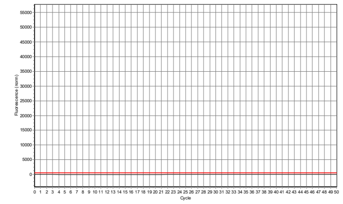
Threshold: 487 (Noiseband)
Baseline settings: automatic, Drift correction OFF

32

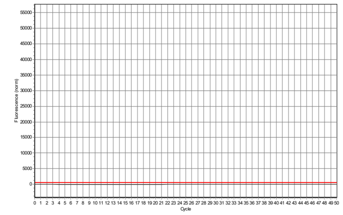
Threshold: 487 (Noiseband)
Baseline settings: automatic, Drift correction OFF

33

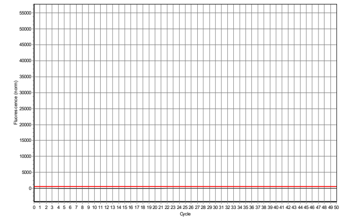
Threshold: 487 (Noiseband)
Baseline settings: automatic, Drift correction OFF

34

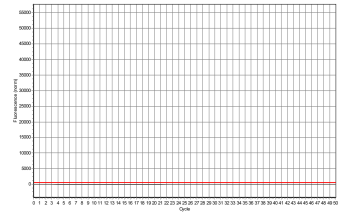
Threshold: 487 (Noiseband)
Baseline settings: automatic, Drift correction OFF

35

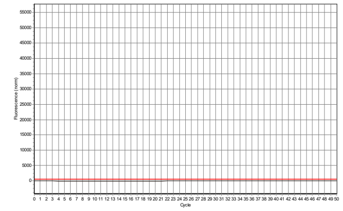
Threshold: 487 (Noiseband)
Baseline settings: automatic, Drift correction OFF

36

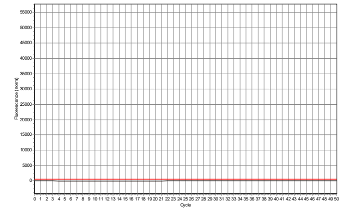
Threshold: 487 (Noiseband)
Baseline settings: automatic, Drift correction OFF

37

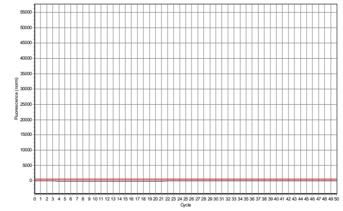
Threshold: 487 (Noiseband)
Baseline settings: automatic, Drift correction OFF

38

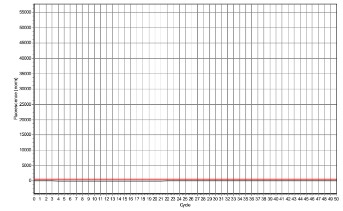
Threshold: 487 (Noiseband)
Baseline settings: automatic, Drift correction OFF

39

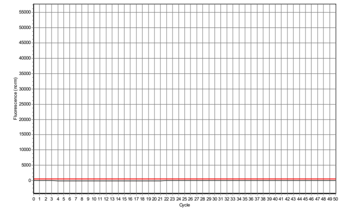
Threshold: 487 (Noiseband)
Baseline settings: automatic, Drift correction OFF

40

TMEM135-CCDC67-PROSTATE-BLOOD SAMPLE 41-60

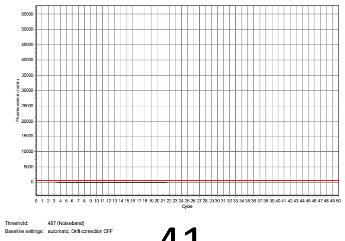

41

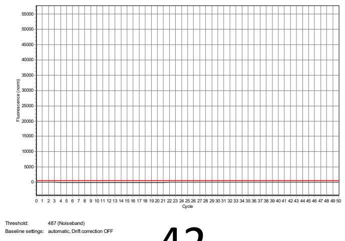

42

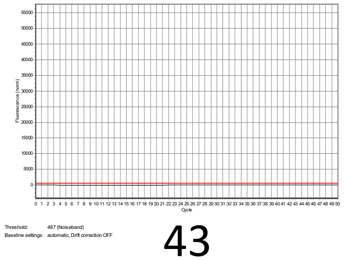

43

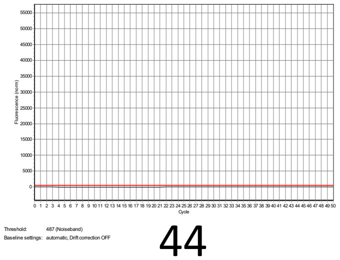

44

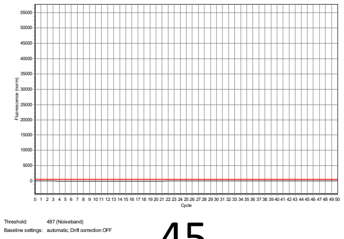

45

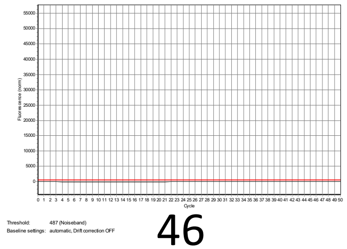

46

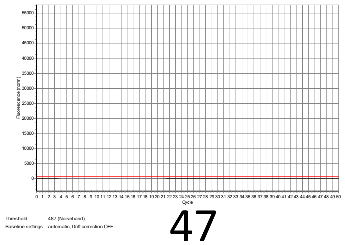

47

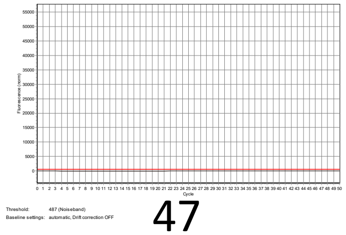

47

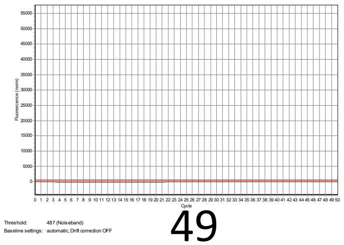

49

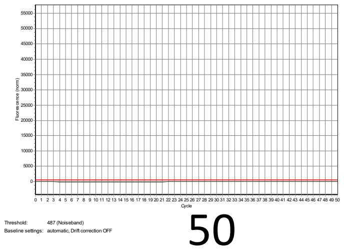

50

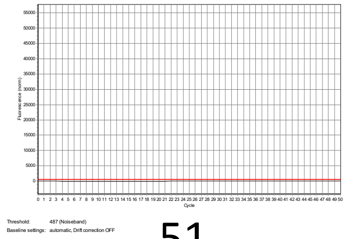

51

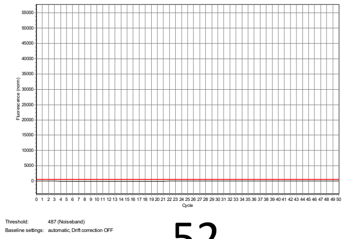

52

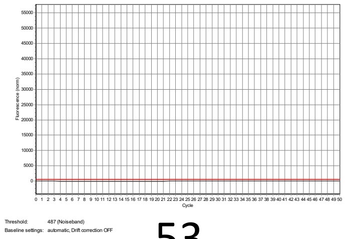

53

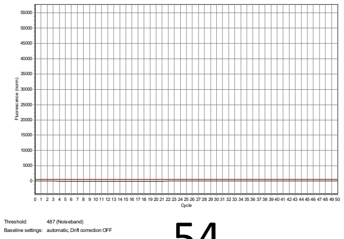

54

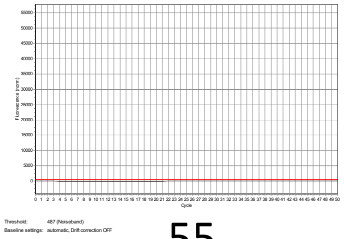

55

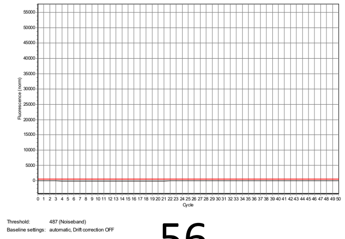

56

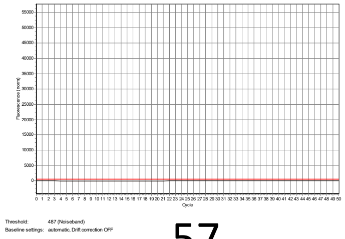

57

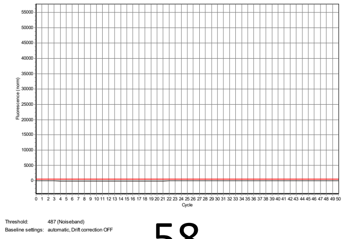

58

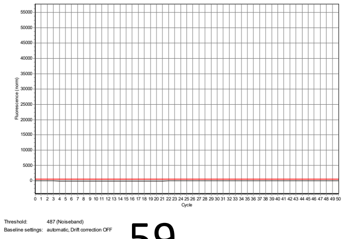

59

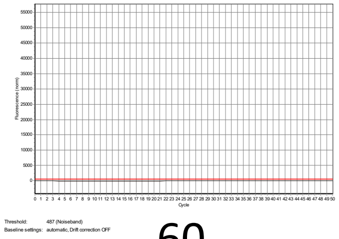

60

TMEM135-CCDC67-PROSTATE-BLOOD SAMPLE 61-80

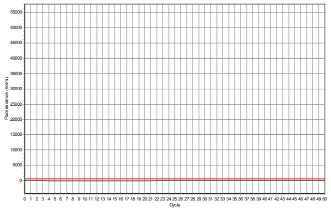
Threshold: 487 (Noiseband)
Baseline settings: automatic, Drift correction OFF

61

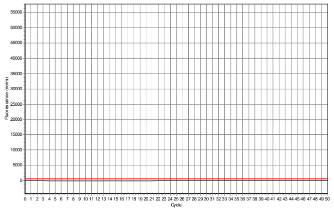
Threshold: 487 (Noiseband)
Baseline settings: automatic, Drift correction OFF

62

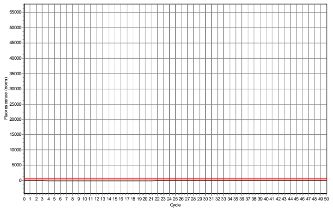
Threshold: 487 (Noiseband)
Baseline settings: automatic, Drift correction OFF

63

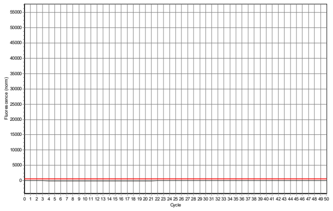
Threshold: 487 (Noiseband)
Baseline settings: automatic, Drift correction OFF

64

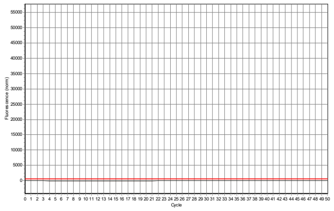
Threshold: 487 (Noiseband)
Baseline settings: automatic, Drift correction OFF

65

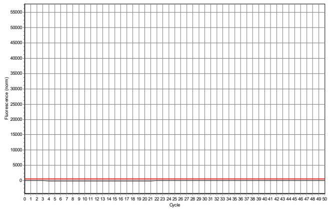
Threshold: 487 (Noiseband)
Baseline settings: automatic, Drift correction OFF

66

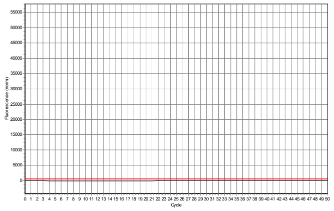
Threshold: 487 (Noiseband)
Baseline settings: automatic, Drift correction OFF

67

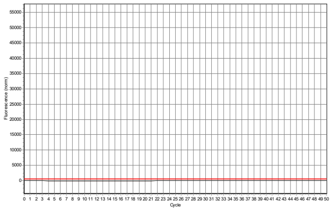
Threshold: 487 (Noiseband)
Baseline settings: automatic, Drift correction OFF

68

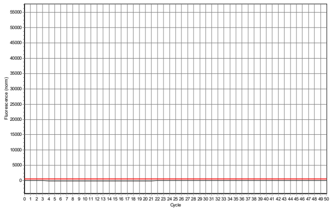
Threshold: 487 (Noiseband)
Baseline settings: automatic, Drift correction OFF

69

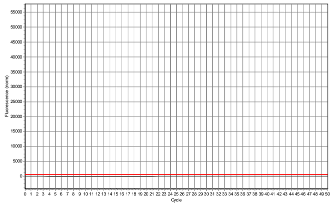
Threshold: 487 (Noiseband)
Baseline settings: automatic, Drift correction OFF

70

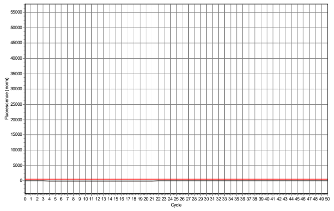
Threshold: 487 (Noiseband)
Baseline settings: automatic, Drift correction OFF

71

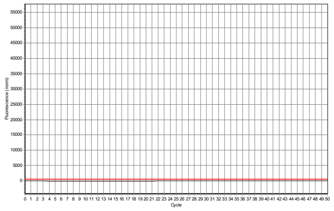
Threshold: 487 (Noiseband)
Baseline settings: automatic, Drift correction OFF

72

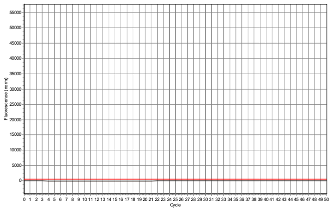
Threshold: 487 (Noiseband)
Baseline settings: automatic, Drift correction OFF

73

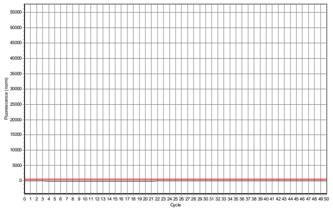
Threshold: 487 (Noiseband)
Baseline settings: automatic, Drift correction OFF

74

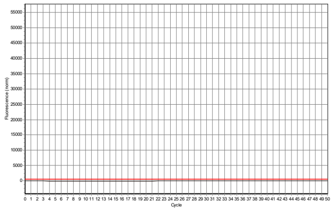
Threshold: 487 (Noiseband)
Baseline settings: automatic, Drift correction OFF

75

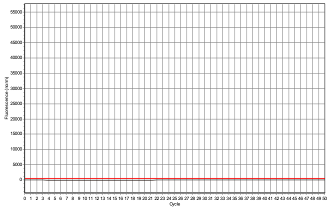
Threshold: 487 (Noiseband)
Baseline settings: automatic, Drift correction OFF

76

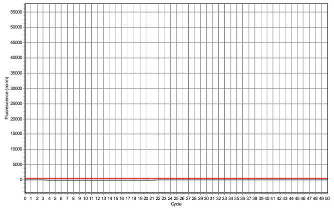
Threshold: 487 (Noiseband)
Baseline settings: automatic, Drift correction OFF

77

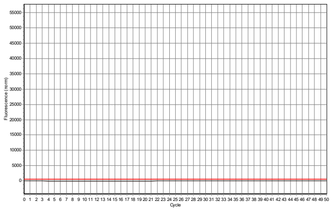
Threshold: 487 (Noiseband)
Baseline settings: automatic, Drift correction OFF

78

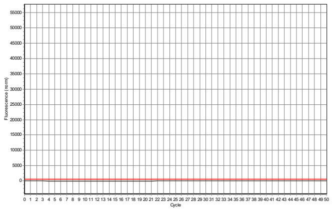
Threshold: 487 (Noiseband)
Baseline settings: automatic, Drift correction OFF

79

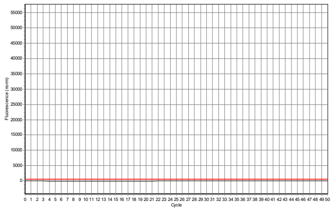
Threshold: 487 (Noiseband)
Baseline settings: automatic, Drift correction OFF

80

TMEM135-CCDC67-PROSTATE-BLOOD SAMPLE 81-100

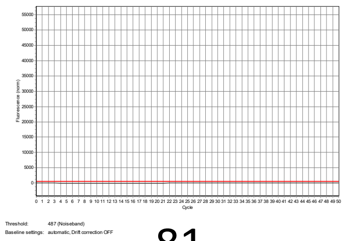

81

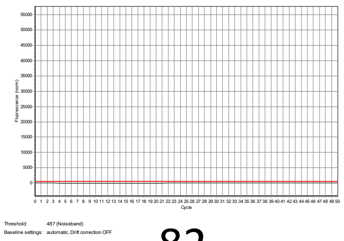

82

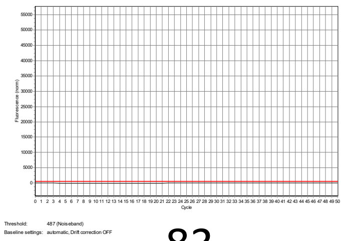

83

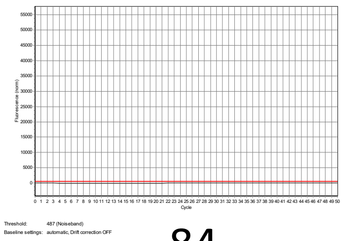

84

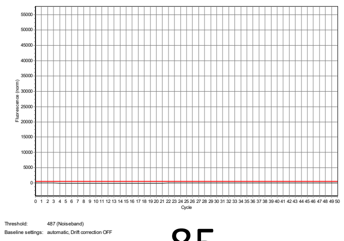

85

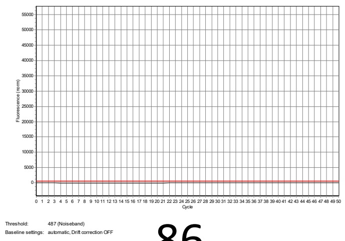

86

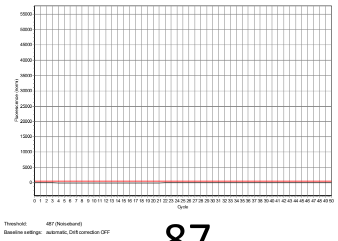

87

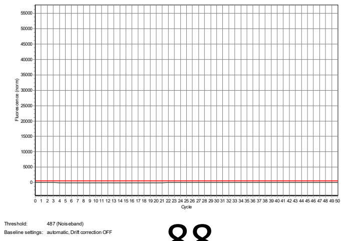

88

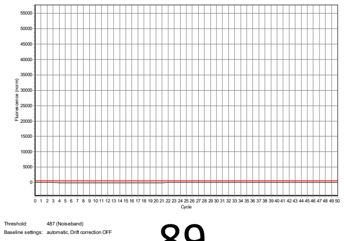

89

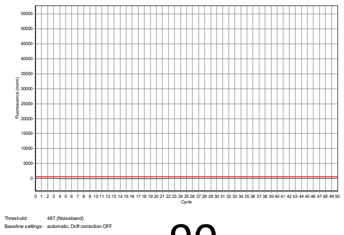

90

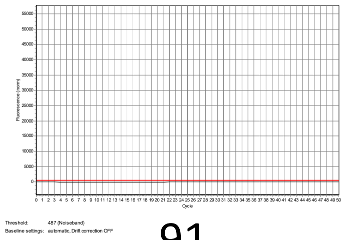

91

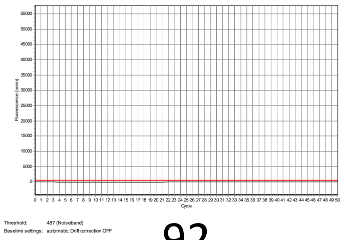

92

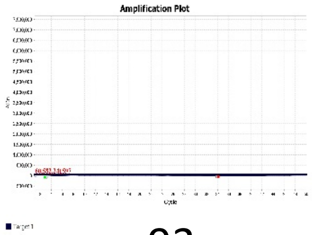

93

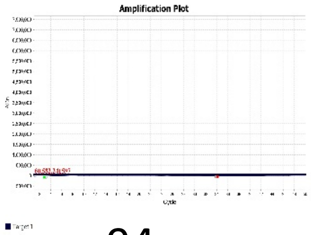

94

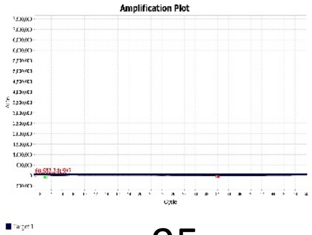

95

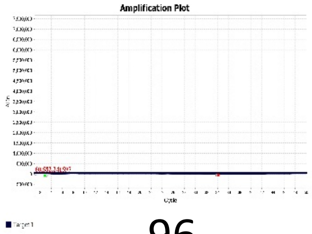

96

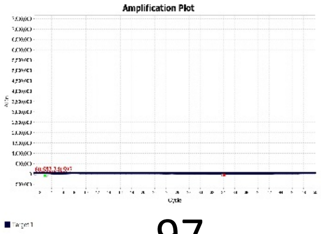

97

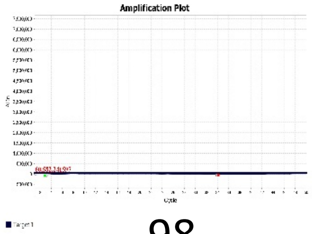

98

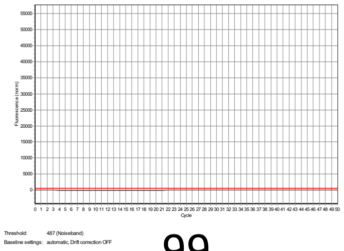

99

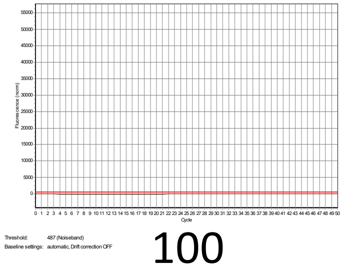

100

TMEM135-CCDC67-PROSTATE-BLOOD SAMPLE 101-120

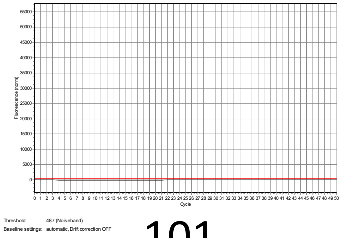
101

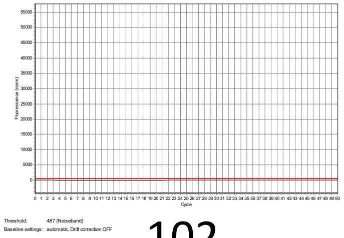
102

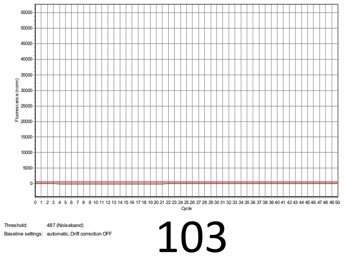
103

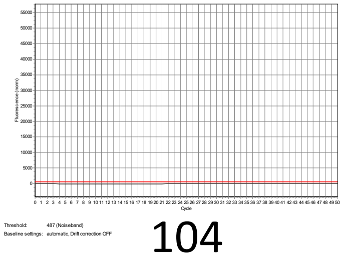
104

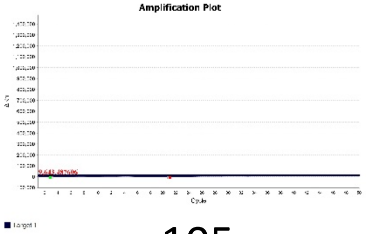

105

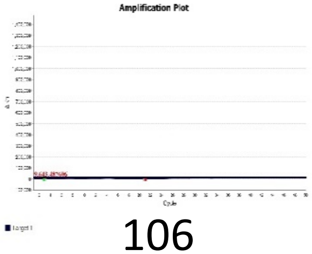

106

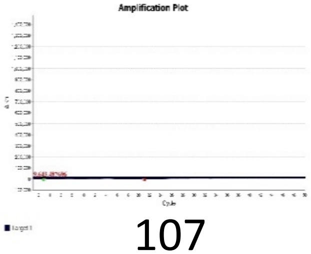

107

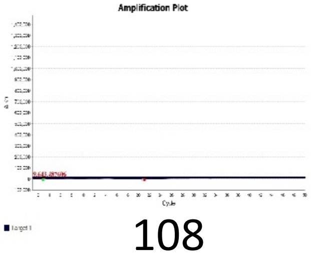

108

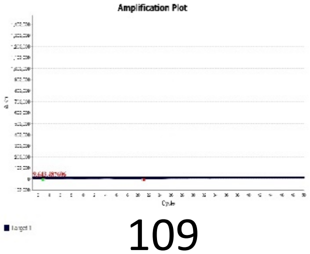

109

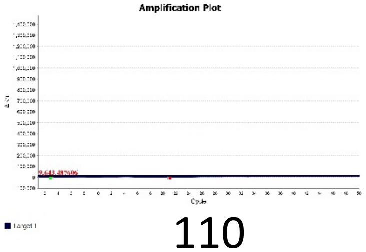

110

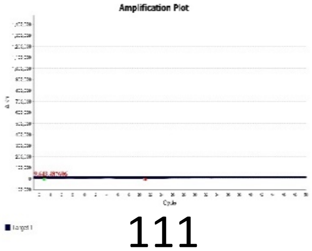

111

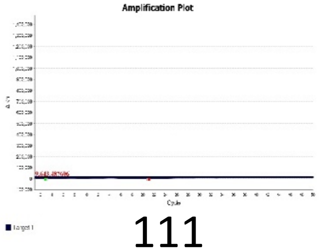

111

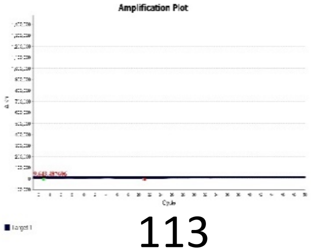

113

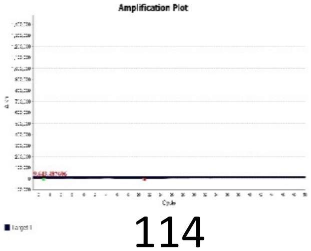

114

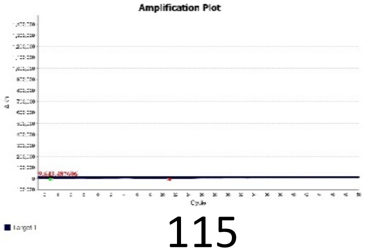

115

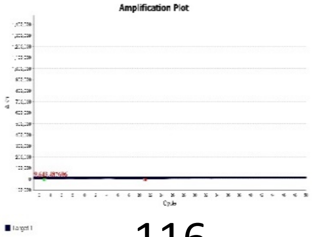

116

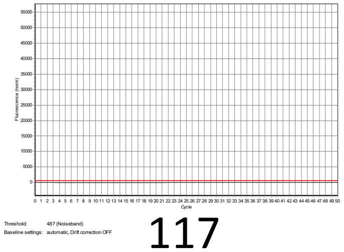
117

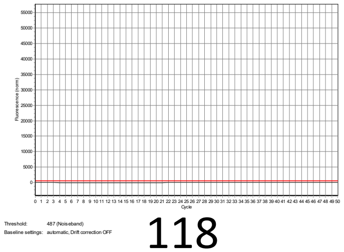
118

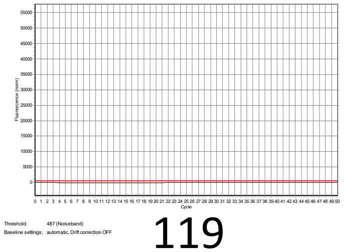
119

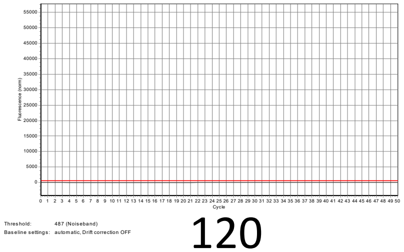
120

TMEM135-CCDC67-PROSTATE-BLOOD SAMPLE 121-140

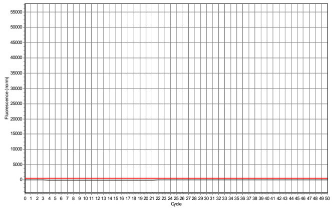
Threshold: 487 (Noiseband)
Baseline settings: automatic, Drift correction OFF

121

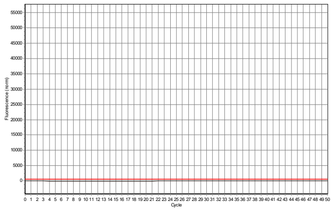
Threshold: 487 (Noiseband)
Baseline settings: automatic, Drift correction OFF

122

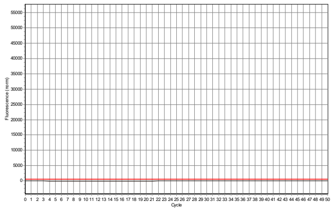
Threshold: 487 (Noiseband)
Baseline settings: automatic, Drift correction OFF

123

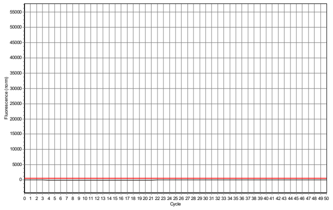
Threshold: 487 (Noiseband)
Baseline settings: automatic, Drift correction OFF

124

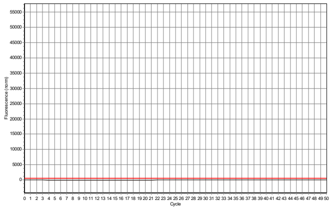
Threshold: 487 (Noiseband)
Baseline settings: automatic, Drift correction OFF

125

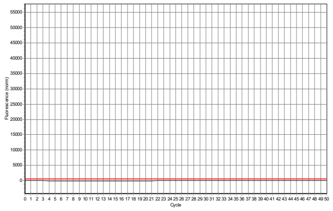
Threshold: 487 (Noiseband)
Baseline settings: automatic, Drift correction OFF

126

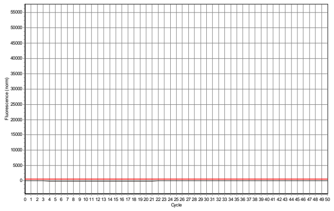
Threshold: 487 (Noiseband)
Baseline settings: automatic, Drift correction OFF

127

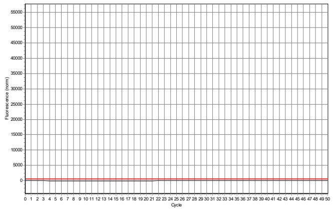
Threshold: 487 (Noiseband)
Baseline settings: automatic, Drift correction OFF

128

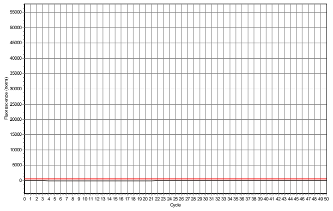
Threshold: 487 (Noiseband)
Baseline settings: automatic, Drift correction OFF

129

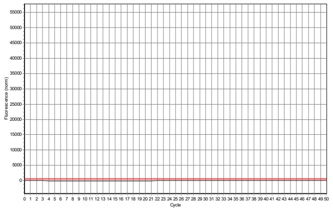
Threshold: 487 (Noiseband)
Baseline settings: automatic, Drift correction OFF

130

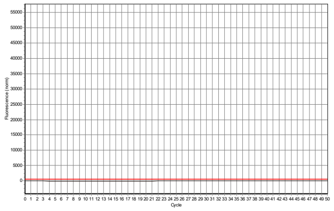
Threshold: 487 (Noiseband)
Baseline settings: automatic, Drift correction OFF

131

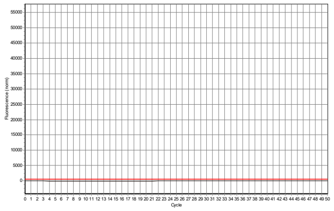
Threshold: 487 (Noiseband)
Baseline settings: automatic, Drift correction OFF

132

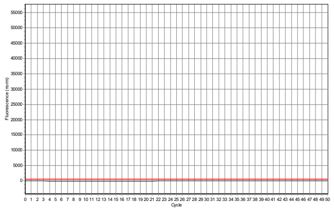
Threshold: 487 (Noiseband)
Baseline settings: automatic, Drift correction OFF

133

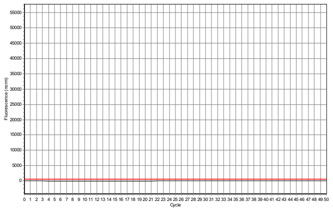
Threshold: 487 (Noiseband)
Baseline settings: automatic, Drift correction OFF

134

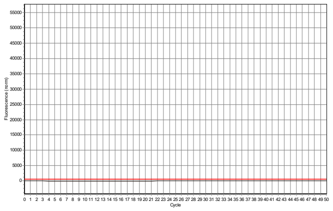
Threshold: 487 (Noiseband)
Baseline settings: automatic, Drift correction OFF

135

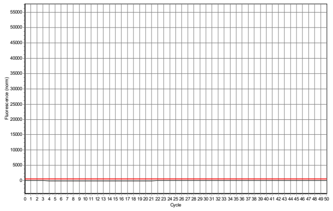
Threshold: 487 (Noiseband)
Baseline settings: automatic, Drift correction OFF

136

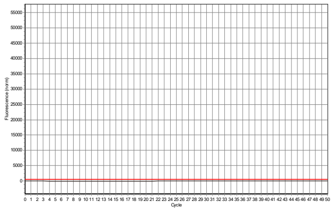
Threshold: 487 (Noiseband)
Baseline settings: automatic, Drift correction OFF

137

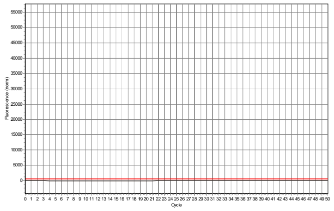
Threshold: 487 (Noiseband)
Baseline settings: automatic, Drift correction OFF

138

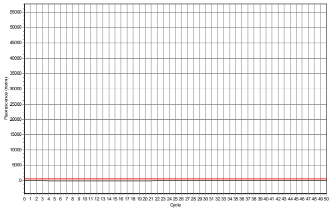
Threshold: 487 (Noiseband)
Baseline settings: automatic, Drift correction OFF

139

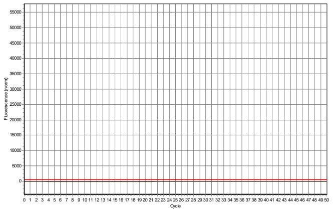
Threshold: 487 (Noiseband)
Baseline settings: automatic, Drift correction OFF

140

TMEM135-CCDC67-PROSTATE-BLOOD SAMPLE 141-150

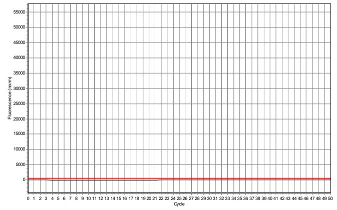

Threshold: 487 (Noiseband)
Baseline settings: automatic, Drift correction OFF

141

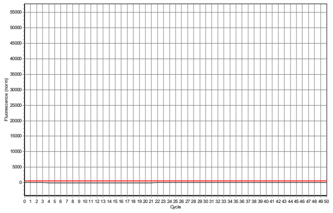

Threshold: 487 (Noiseband)
Baseline settings: automatic, Drift correction OFF

142

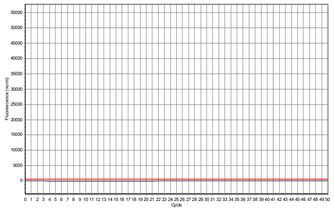

Threshold: 487 (Noiseband)
Baseline settings: automatic, Drift correction OFF

143

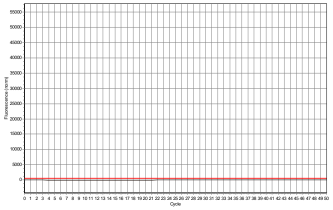

Threshold: 487 (Noiseband)
Baseline settings: automatic, Drift correction OFF

144

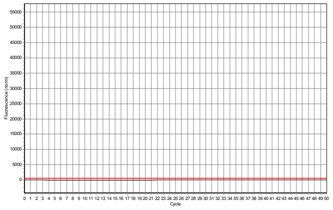

Threshold: 487 (Noiseband)
Baseline settings: automatic, Drift correction OFF

145

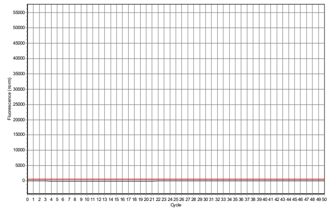

Threshold: 487 (Noiseband)
Baseline settings: automatic, Drift correction OFF

146

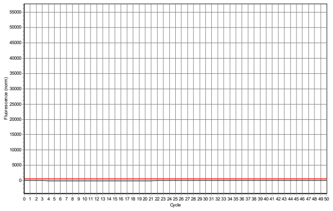

Threshold: 487 (Noiseband)
Baseline settings: automatic, Drift correction OFF

147

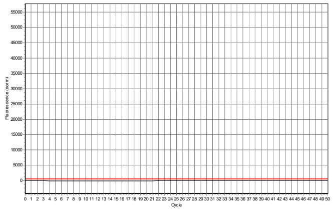

Threshold: 487 (Noiseband)
Baseline settings: automatic, Drift correction OFF

148

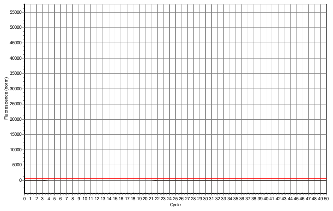

Threshold: 487 (Noiseband)
Baseline settings: automatic, Drift correction OFF

149

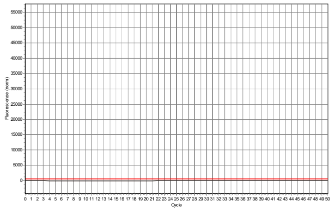

Threshold: 487 (Noiseband)
Baseline settings: automatic, Drift correction OFF

150

Supplemental figure 8

LRRC59-FLJ60017 fusion detection

LRRC59-FLJ60017-PROSTATE-BLOOD SAMPLE 1-20

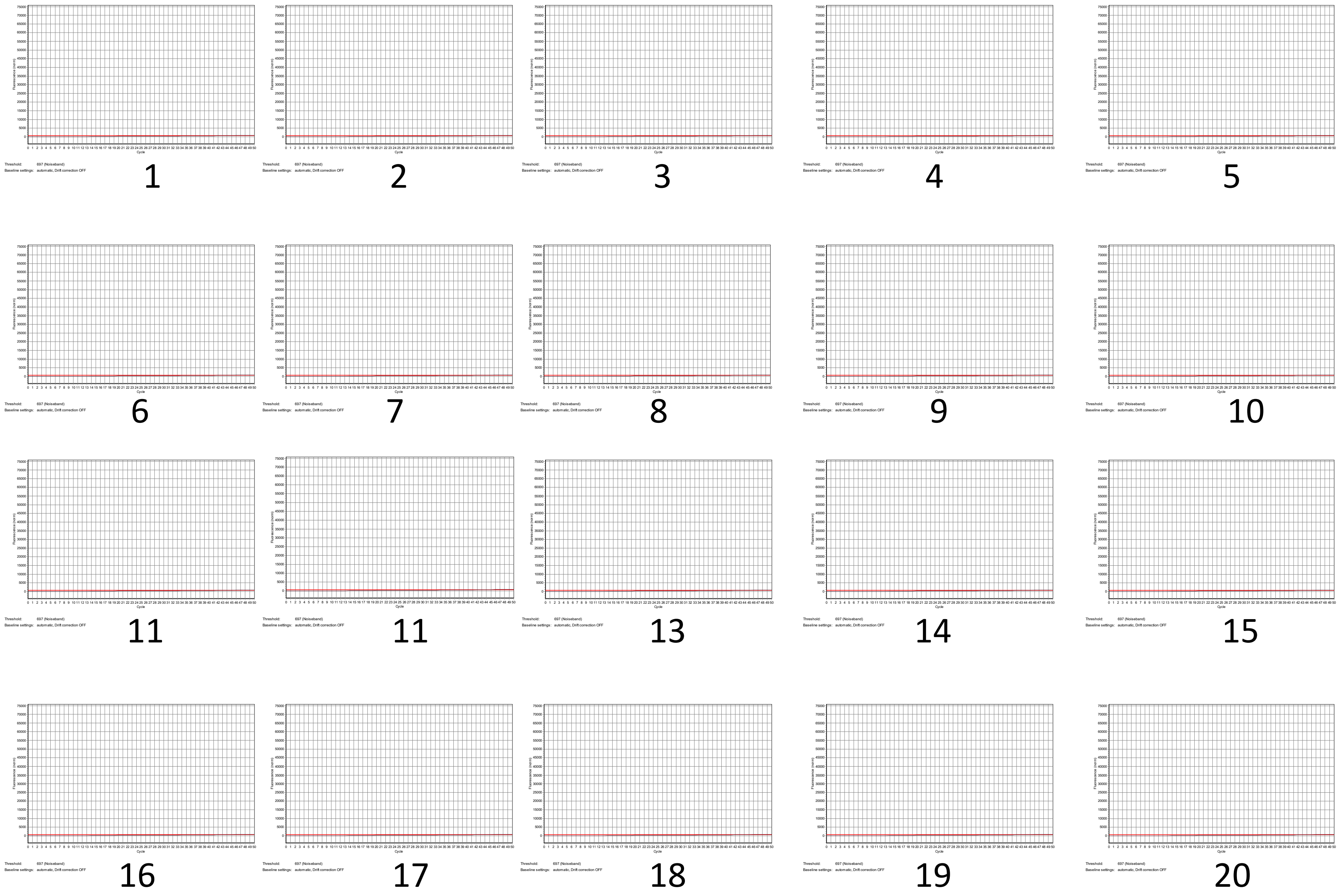

LRRC59-FLJ60017-PROSTATE-BLOOD SAMPLE 21-40

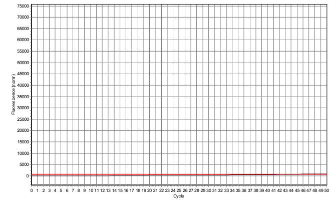

Threshold: 697 (Noiseband)
Baseline settings: automatic, Drift correction OFF

21

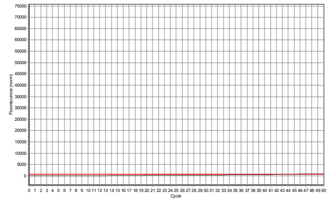

Threshold: 697 (Noiseband)
Baseline settings: automatic, Drift correction OFF

22

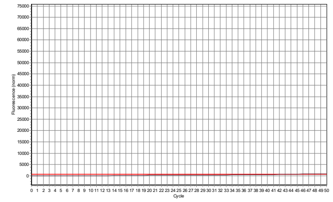

Threshold: 697 (Noiseband)
Baseline settings: automatic, Drift correction OFF

23

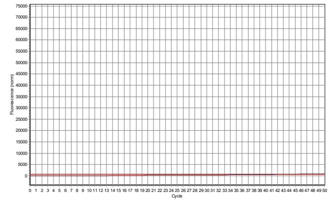

Threshold: 697 (Noiseband)
Baseline settings: automatic, Drift correction OFF

24

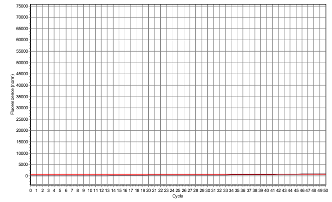

Threshold: 697 (Noiseband)
Baseline settings: automatic, Drift correction OFF

25

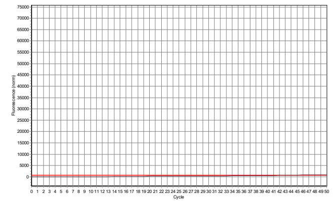

Threshold: 697 (Noiseband)
Baseline settings: automatic, Drift correction OFF

26

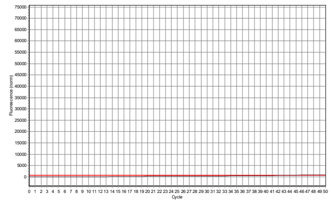

Threshold: 697 (Noiseband)
Baseline settings: automatic, Drift correction OFF

27

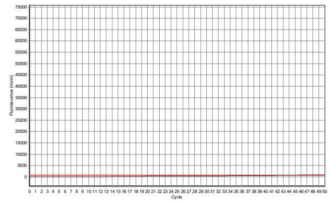

Threshold: 697 (Noiseband)
Baseline settings: automatic, Drift correction OFF

28

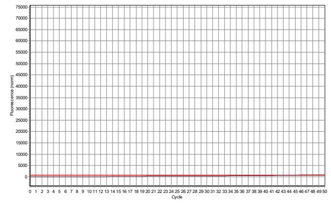

Threshold: 697 (Noiseband)
Baseline settings: automatic, Drift correction OFF

29

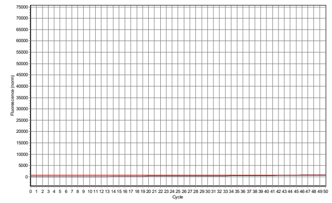

Threshold: 697 (Noiseband)
Baseline settings: automatic, Drift correction OFF

30

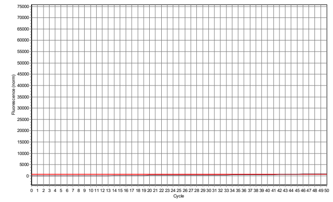

Threshold: 697 (Noiseband)
Baseline settings: automatic, Drift correction OFF

31

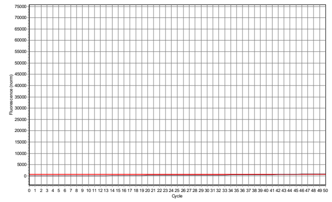

Threshold: 697 (Noiseband)
Baseline settings: automatic, Drift correction OFF

32

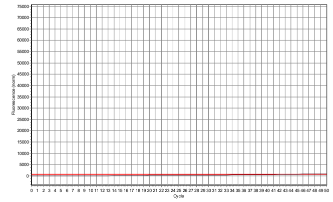

Threshold: 697 (Noiseband)
Baseline settings: automatic, Drift correction OFF

33

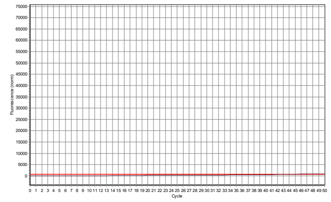

Threshold: 697 (Noiseband)
Baseline settings: automatic, Drift correction OFF

34

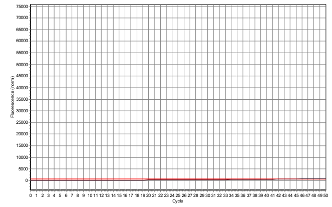

Threshold: 697 (Noiseband)
Baseline settings: automatic, Drift correction OFF

35

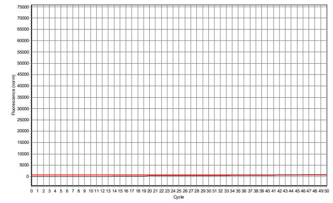

Threshold: 697 (Noiseband)
Baseline settings: automatic, Drift correction OFF

36

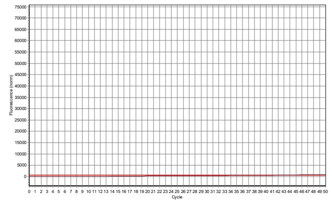

Threshold: 697 (Noiseband)
Baseline settings: automatic, Drift correction OFF

37

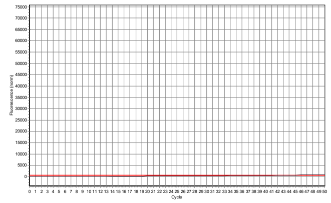

Threshold: 697 (Noiseband)
Baseline settings: automatic, Drift correction OFF

38

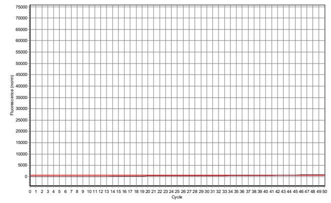

Threshold: 697 (Noiseband)
Baseline settings: automatic, Drift correction OFF

39

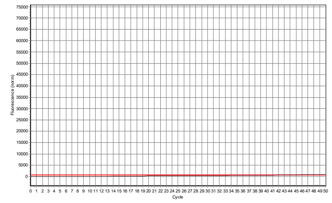

Threshold: 697 (Noiseband)
Baseline settings: automatic, Drift correction OFF

40

LRRC-PROSTATE-BLOOD SAMPLE 41-60

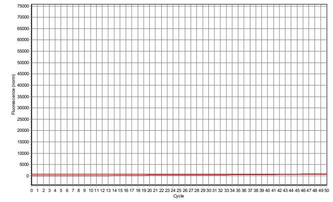

Threshold: 697 (Noiseband)
Baseline settings: automatic, Drift correction OFF

41

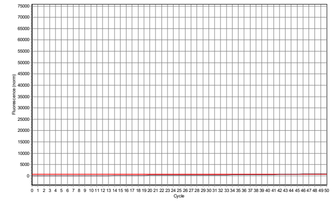

Threshold: 697 (Noiseband)
Baseline settings: automatic, Drift correction OFF

42

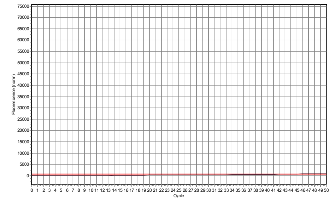

Threshold: 697 (Noiseband)
Baseline settings: automatic, Drift correction OFF

43

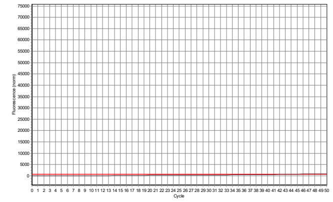

Threshold: 697 (Noiseband)
Baseline settings: automatic, Drift correction OFF

44

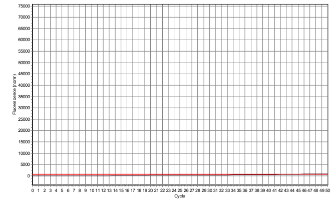

Threshold: 697 (Noiseband)
Baseline settings: automatic, Drift correction OFF

45

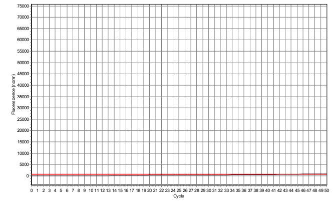

Threshold: 697 (Noiseband)
Baseline settings: automatic, Drift correction OFF

46

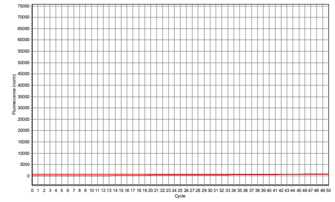

Threshold: 697 (Noiseband)
Baseline settings: automatic, Drift correction OFF

47

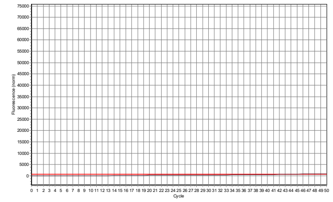

Threshold: 697 (Noiseband)
Baseline settings: automatic, Drift correction OFF

47

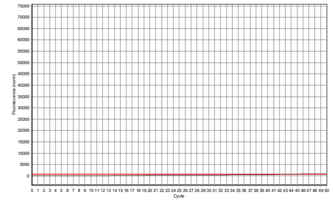

Threshold: 697 (Noiseband)
Baseline settings: automatic, Drift correction OFF

49

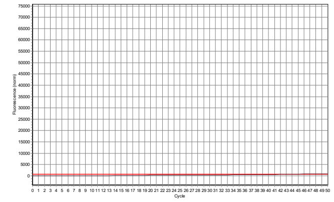

Threshold: 697 (Noiseband)
Baseline settings: automatic, Drift correction OFF

50

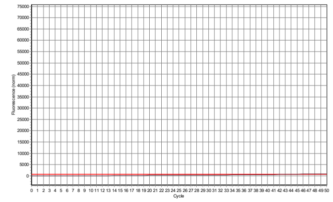

Threshold: 697 (Noiseband)
Baseline settings: automatic, Drift correction OFF

51

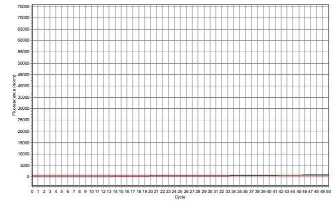

Threshold: 697 (Noiseband)
Baseline settings: automatic, Drift correction OFF

52

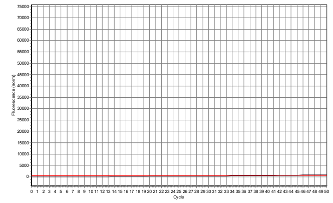

Threshold: 697 (Noiseband)
Baseline settings: automatic, Drift correction OFF

53

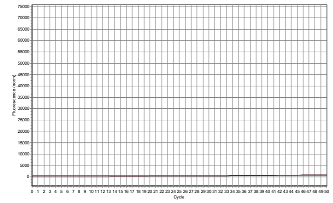

Threshold: 697 (Noiseband)
Baseline settings: automatic, Drift correction OFF

54

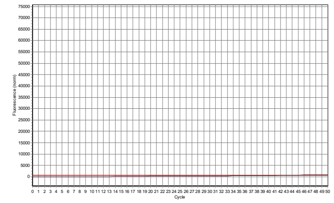

Threshold: 697 (Noiseband)
Baseline settings: automatic, Drift correction OFF

55

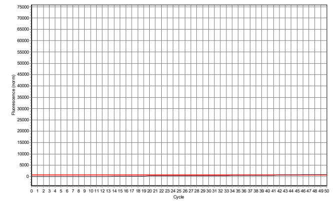

Threshold: 697 (Noiseband)
Baseline settings: automatic, Drift correction OFF

56

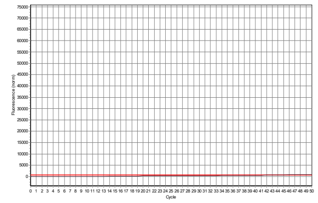

Threshold: 697 (Noiseband)
Baseline settings: automatic, Drift correction OFF

57

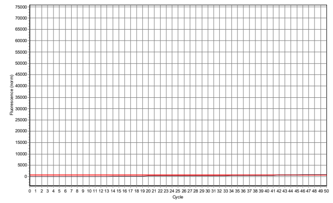

Threshold: 697 (Noiseband)
Baseline settings: automatic, Drift correction OFF

58

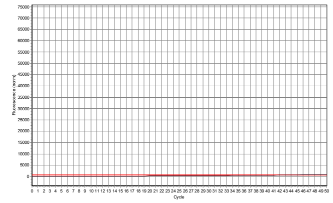

Threshold: 697 (Noiseband)
Baseline settings: automatic, Drift correction OFF

59

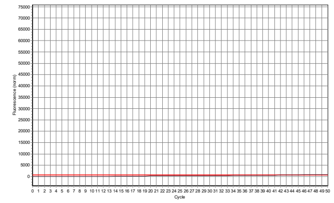

Threshold: 697 (Noiseband)
Baseline settings: automatic, Drift correction OFF

60

LRRC-PROSTATE-BLOOD SAMPLE 61-80

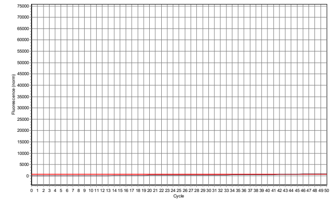

Threshold: 697 (Noiseband)
Baseline settings: automatic, Drift correction OFF

61

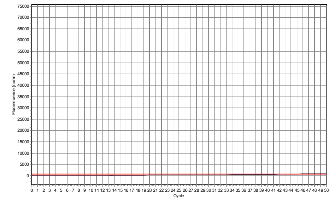

Threshold: 697 (Noiseband)
Baseline settings: automatic, Drift correction OFF

62

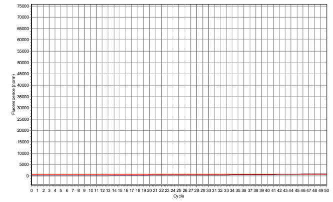

Threshold: 697 (Noiseband)
Baseline settings: automatic, Drift correction OFF

63

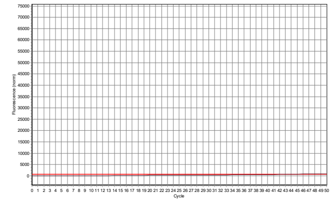

Threshold: 697 (Noiseband)
Baseline settings: automatic, Drift correction OFF

64

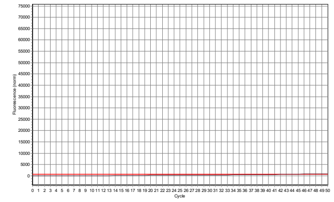

Threshold: 697 (Noiseband)
Baseline settings: automatic, Drift correction OFF

65

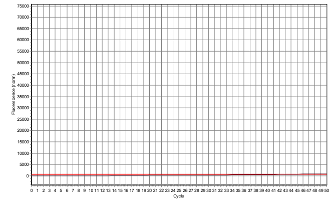

Threshold: 697 (Noiseband)
Baseline settings: automatic, Drift correction OFF

66

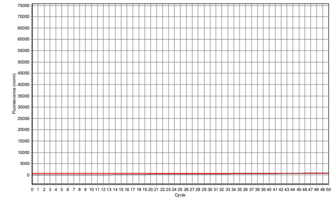

Threshold: 697 (Noiseband)
Baseline settings: automatic, Drift correction OFF

67

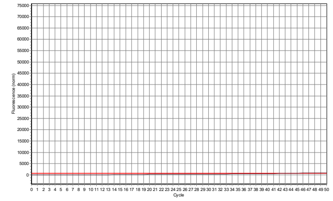

Threshold: 697 (Noiseband)
Baseline settings: automatic, Drift correction OFF

68

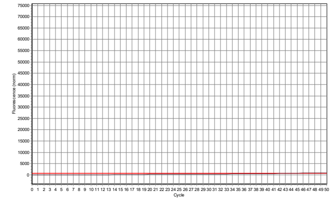

Threshold: 697 (Noiseband)
Baseline settings: automatic, Drift correction OFF

69

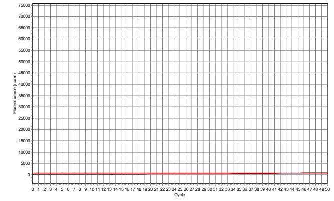

Threshold: 697 (Noiseband)
Baseline settings: automatic, Drift correction OFF

70

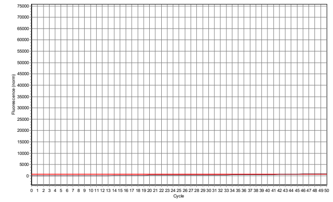

Threshold: 697 (Noiseband)
Baseline settings: automatic, Drift correction OFF

71

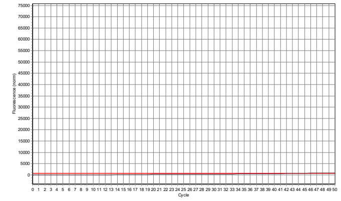

Threshold: 697 (Noiseband)
Baseline settings: automatic, Drift correction OFF

72

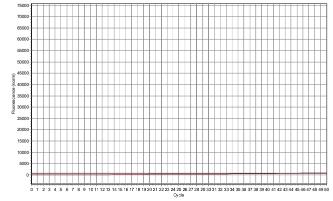

Threshold: 697 (Noiseband)
Baseline settings: automatic, Drift correction OFF

73

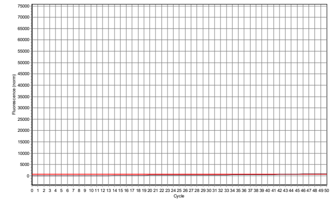

Threshold: 697 (Noiseband)
Baseline settings: automatic, Drift correction OFF

74

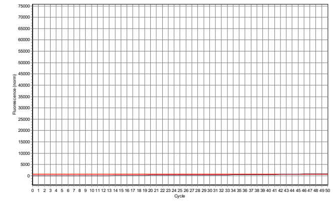

Threshold: 697 (Noiseband)
Baseline settings: automatic, Drift correction OFF

75

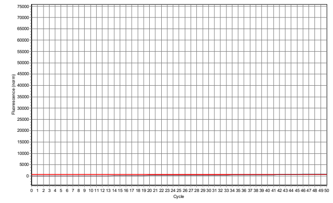

Threshold: 697 (Noiseband)
Baseline settings: automatic, Drift correction OFF

76

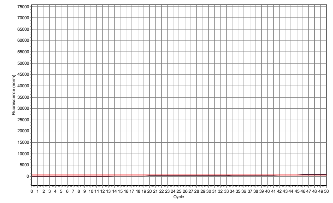

Threshold: 697 (Noiseband)
Baseline settings: automatic, Drift correction OFF

77

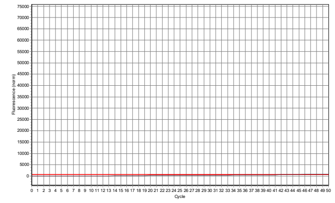

Threshold: 697 (Noiseband)
Baseline settings: automatic, Drift correction OFF

78

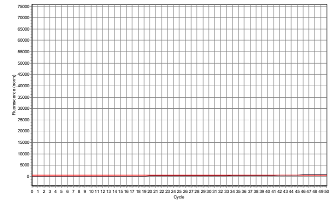

Threshold: 697 (Noiseband)
Baseline settings: automatic, Drift correction OFF

79

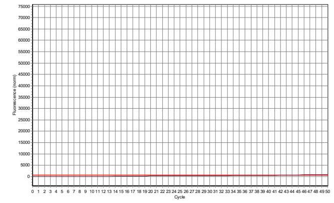

Threshold: 697 (Noiseband)
Baseline settings: automatic, Drift correction OFF

80

LRRC-PROSTATE-BLOOD SAMPLE 81-100

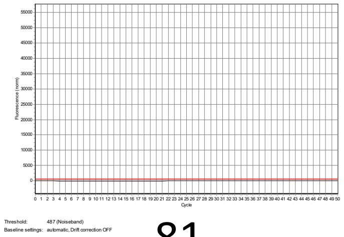

81

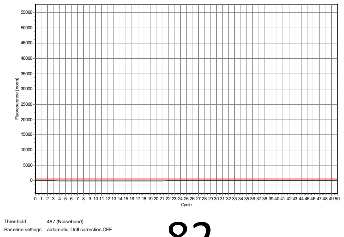

82

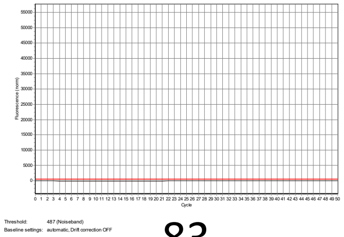

83

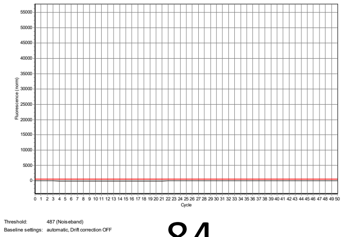

84

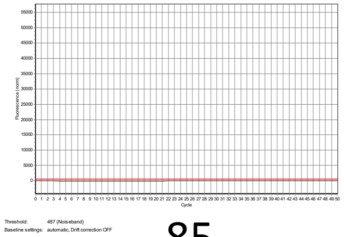

85

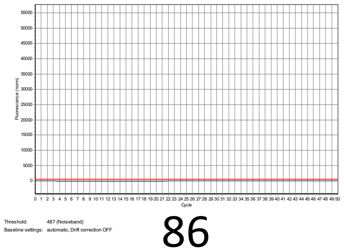

86

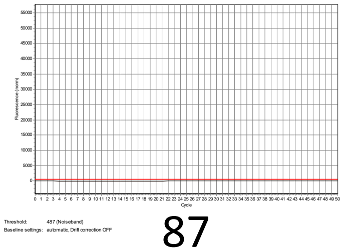

87

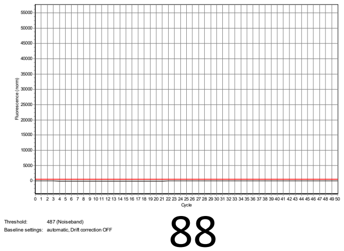

88

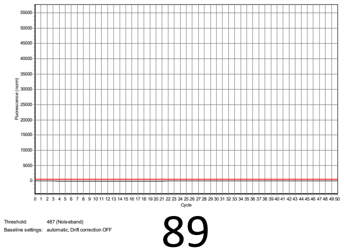

89

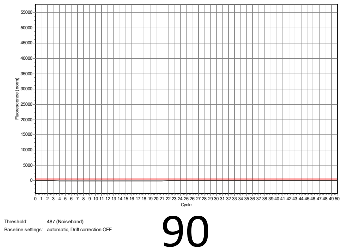

90

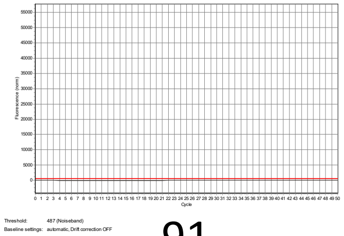

91

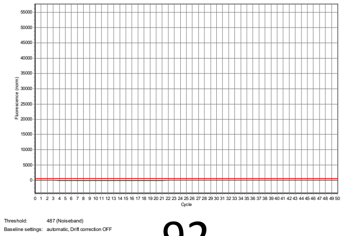

92

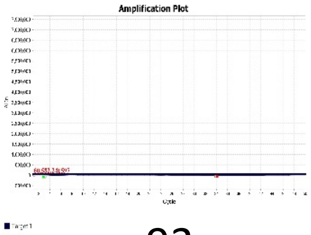

93

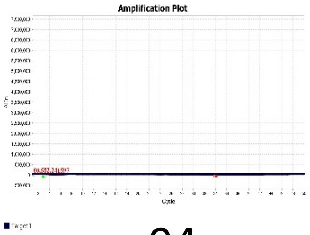

94

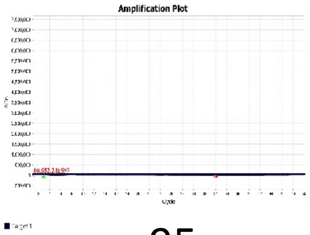

95

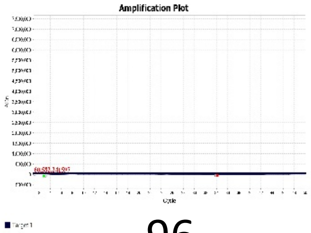

96

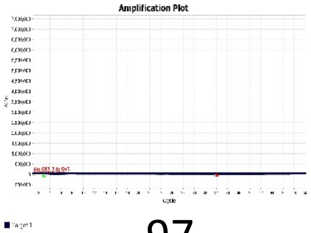

97

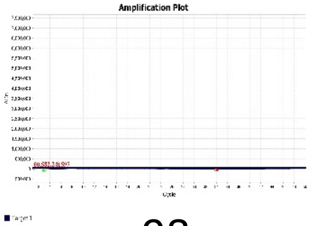

98

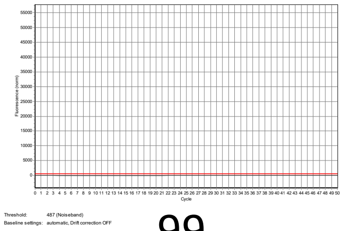

99

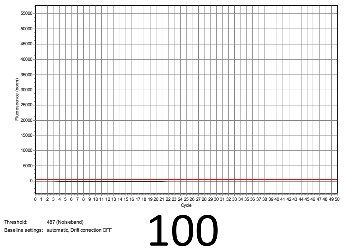

100

LRRC-PROSTATE-BLOOD SAMPLE 101-120

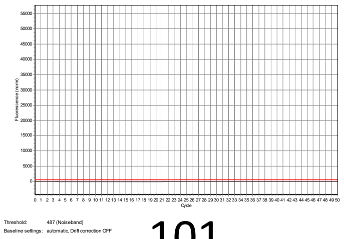

101

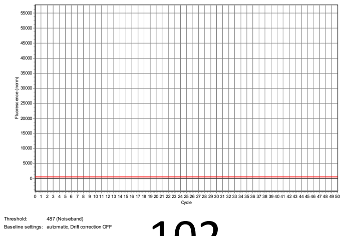

102

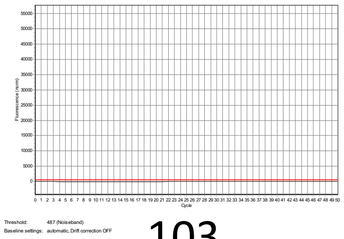

103

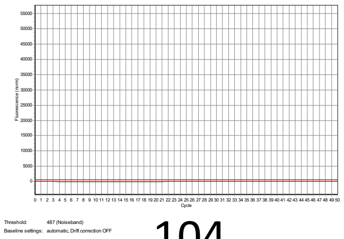

104

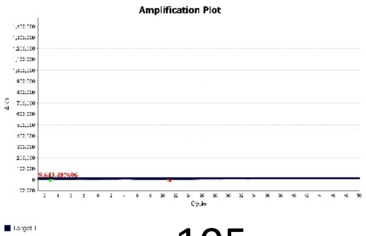

105

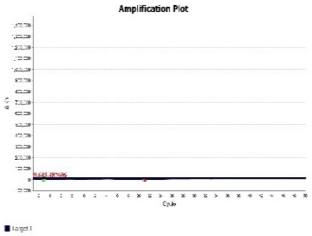

106

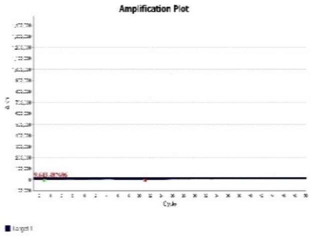

107

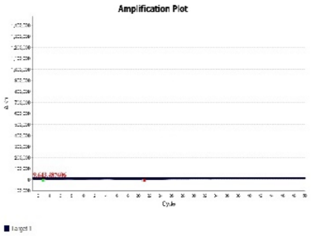

108

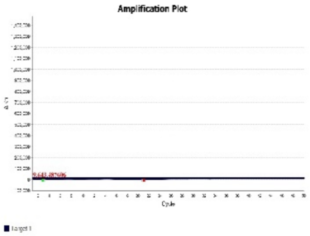

109

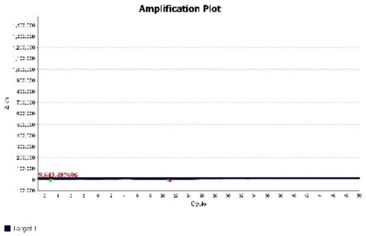

110

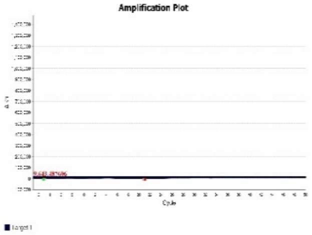

111

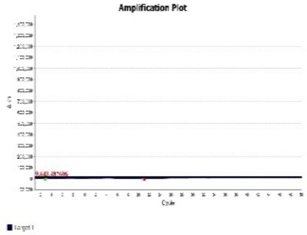

112

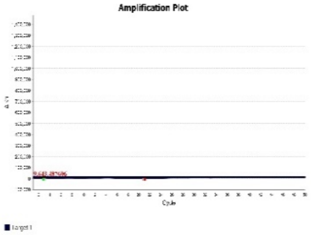

113

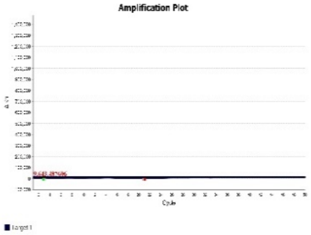

114

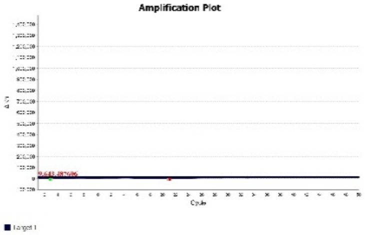

115

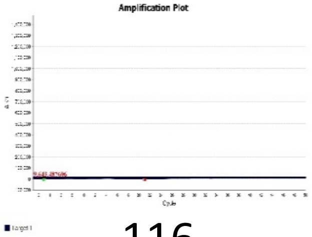

116

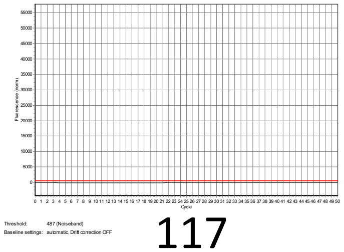

117

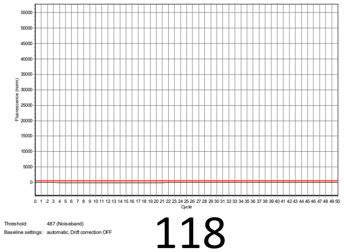

118

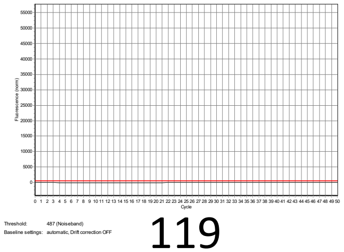

119

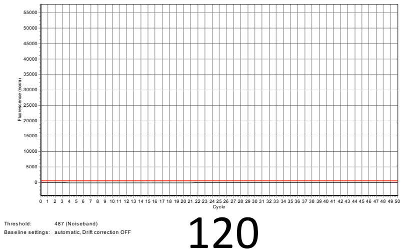

120

LRRC-PROSTATE-BLOOD SAMPLE 121-140

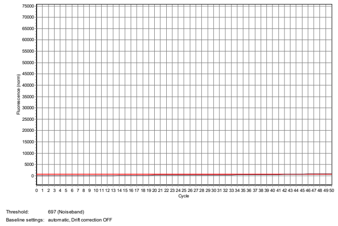

121

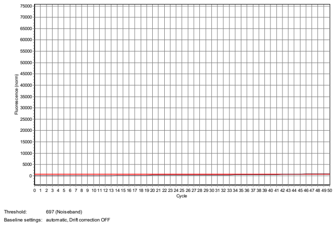

122

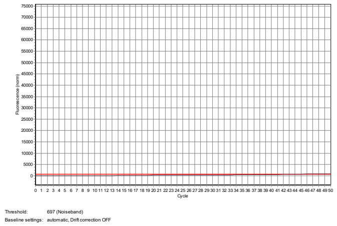

123

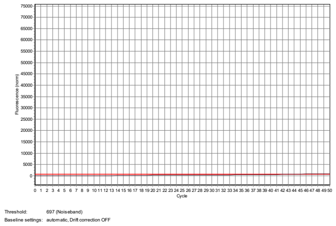

124

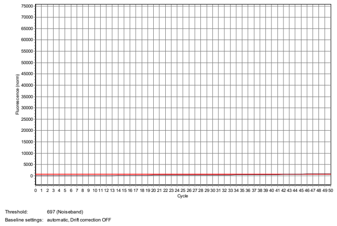

125

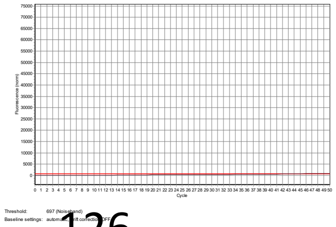

126

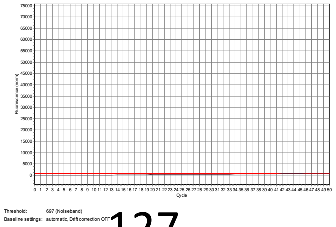

127

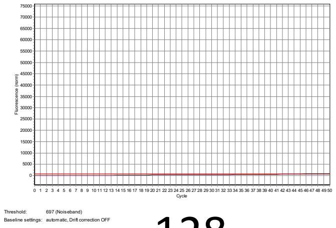

128

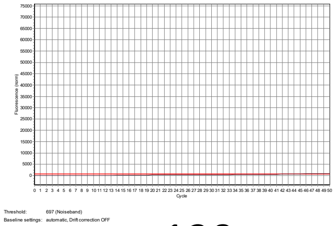

129

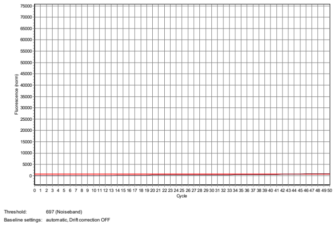

130

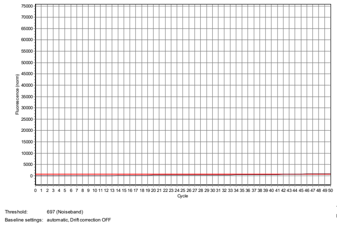

131

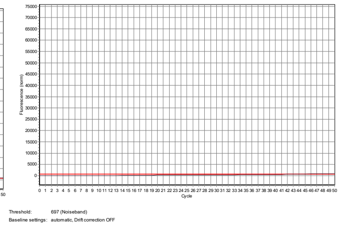

132

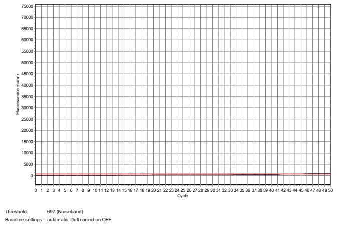

133

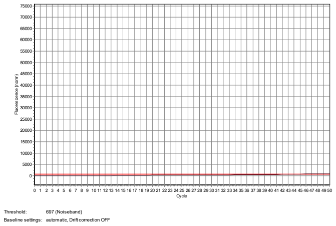

134

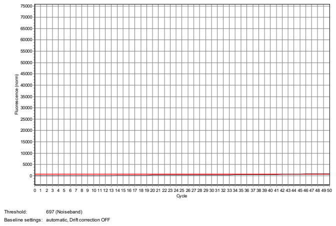

135

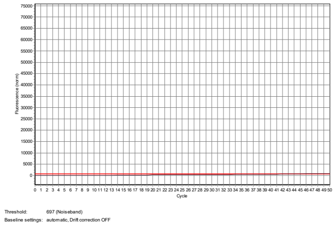

136

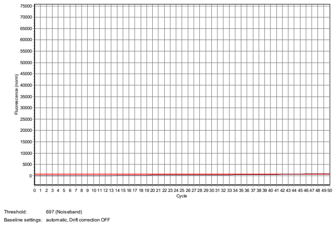

137

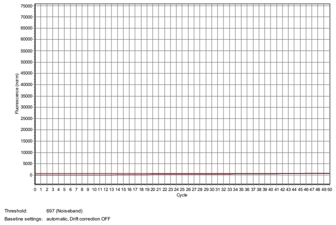

138

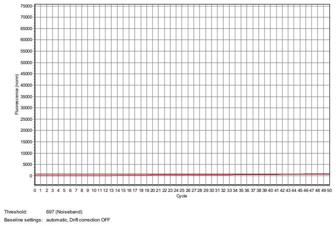

139

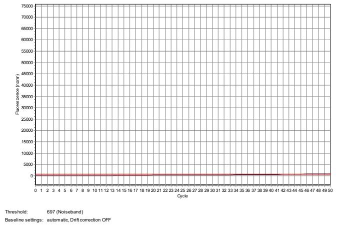

140

LRRC-PROSTATE-BLOOD SAMPLE 141-150

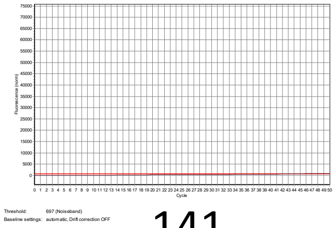

141

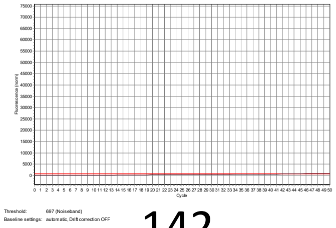

142

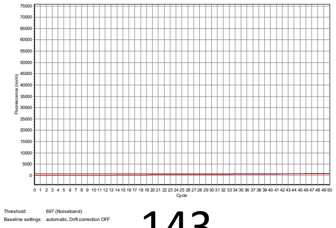

143

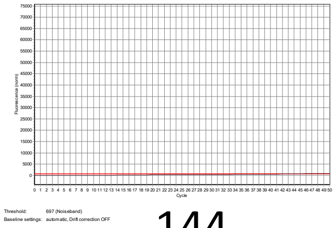

144

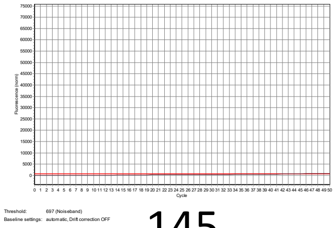

145

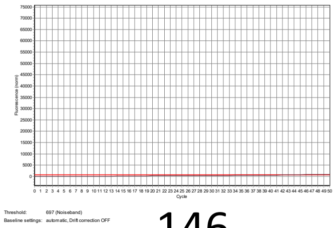

146

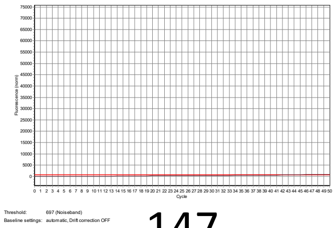

147

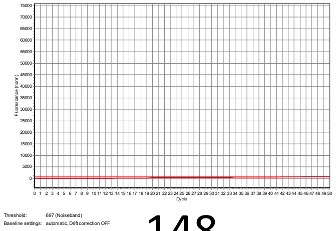

148

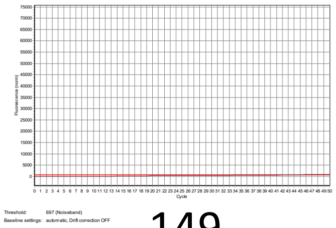

149

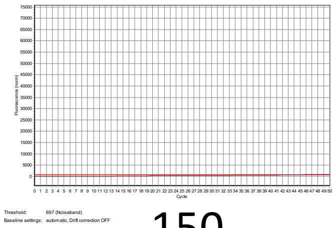

150

Supplemental figure 9

Pten-NOLC1 fusion detection

Pten-NOLC1-PROSTATE-BLOOD SAMPLE 1-20

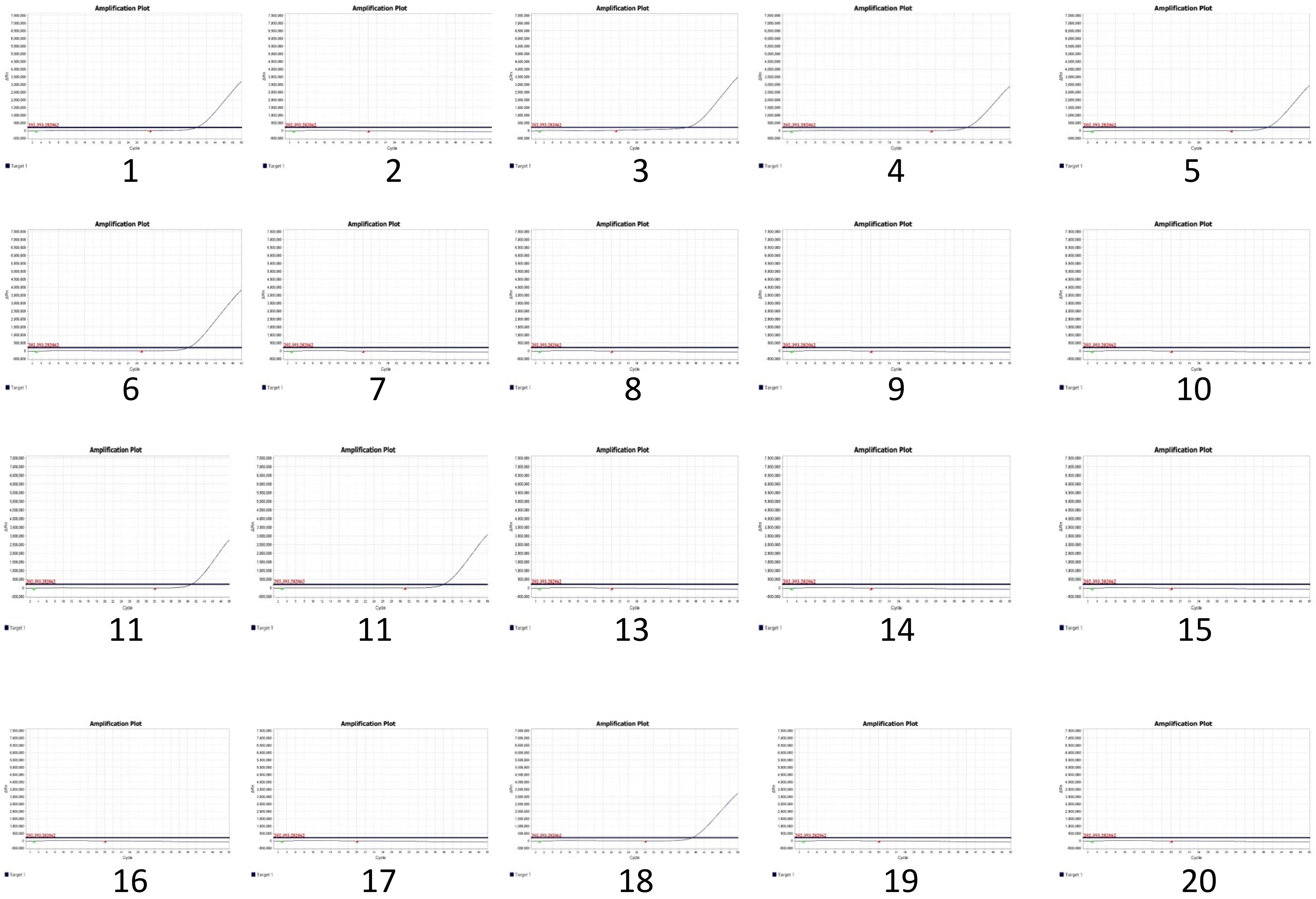

Pten-NOLC1-PROSTATE-BLOOD SAMPLE 21-40

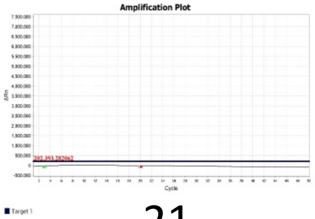

21

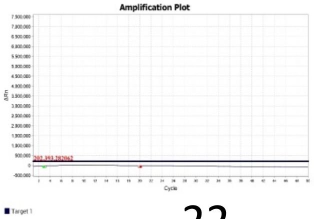

22

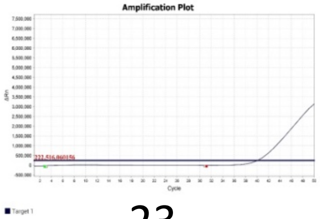

23

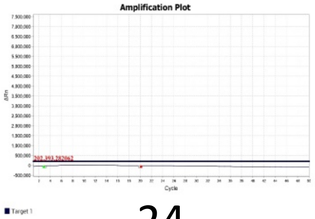

24

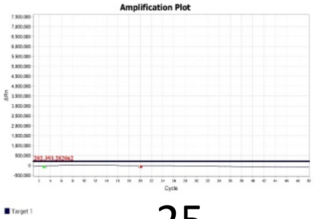

25

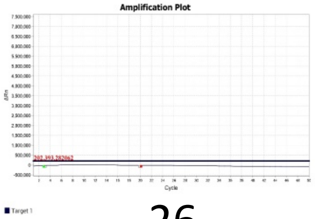

26

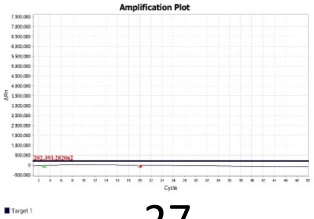

27

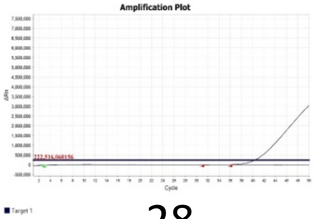

28

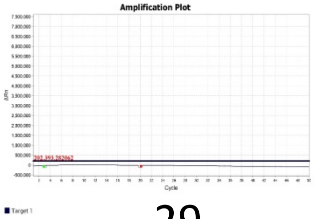

29

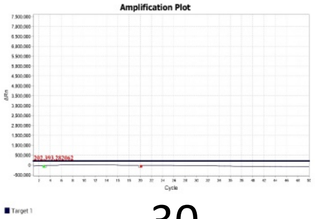

30

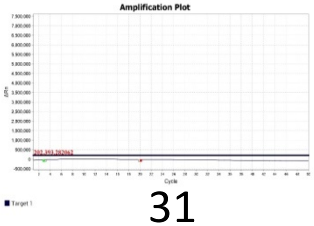

31

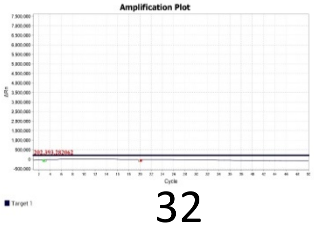

32

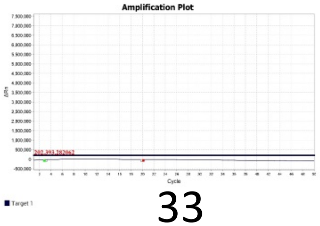

33

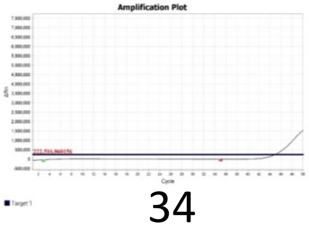

34

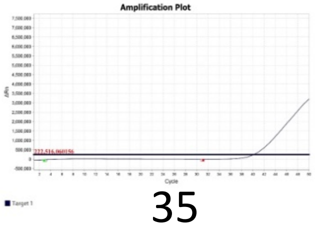

35

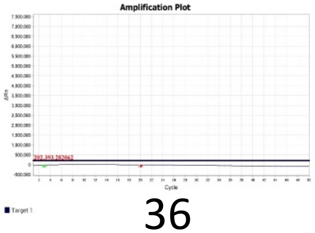

36

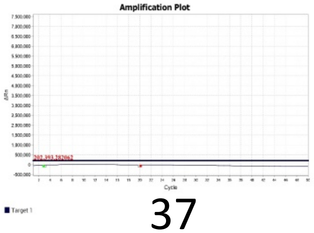

37

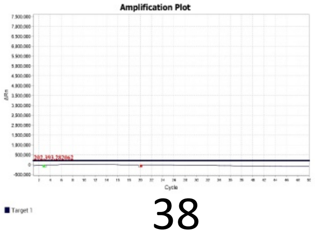

38

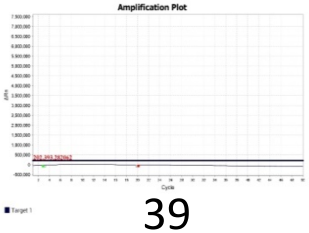

39

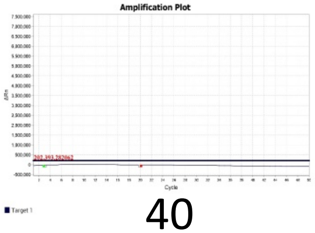

40

Pten-NOLC1-PROSTATE-BLOOD SAMPLE 41-60

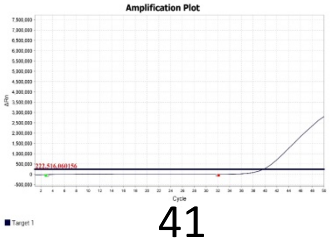

41

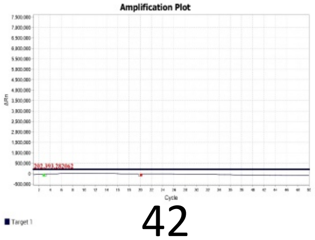

42

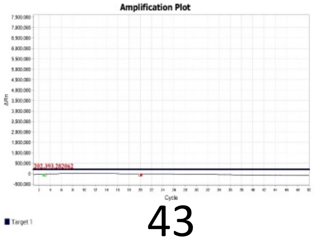

43

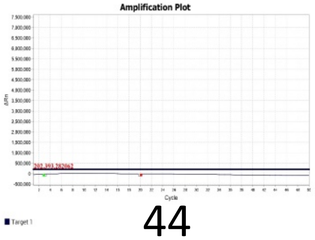

44

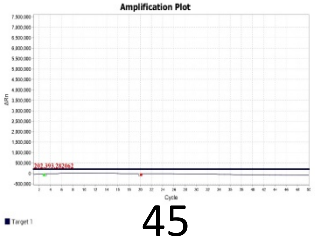

45

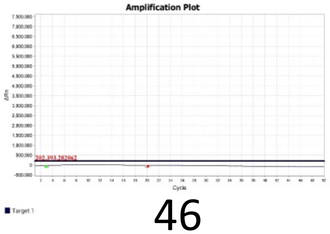

46

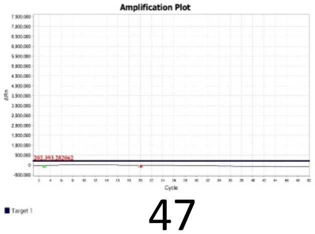

47

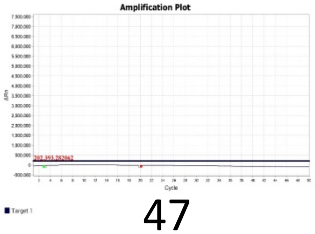

47

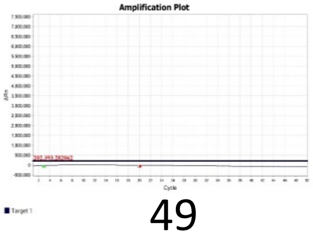

49

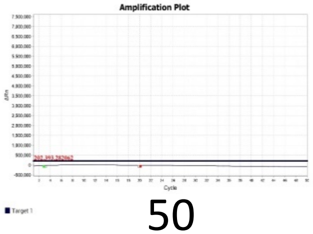

50

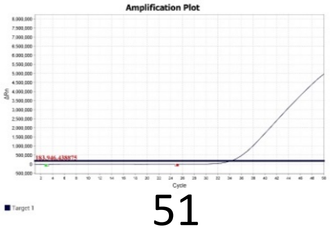

51

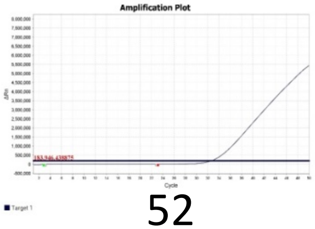

52

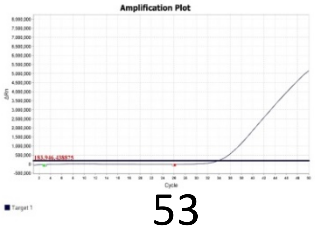

53

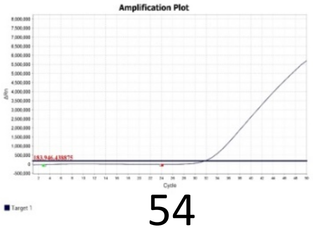

54

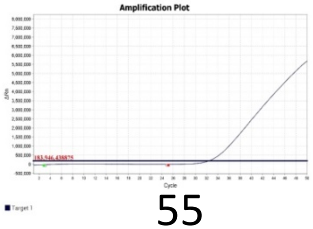

55

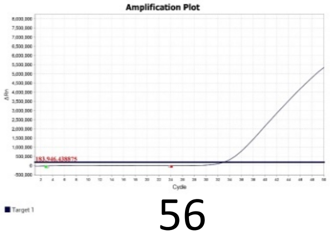

56

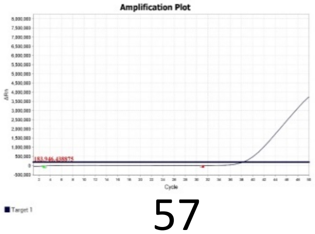

57

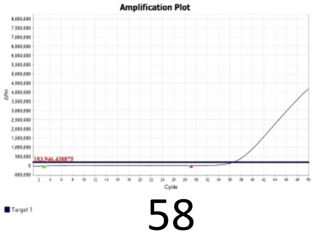

58

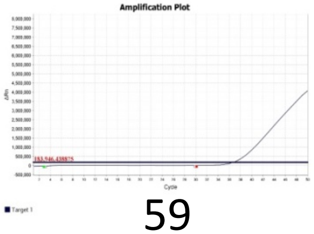

59

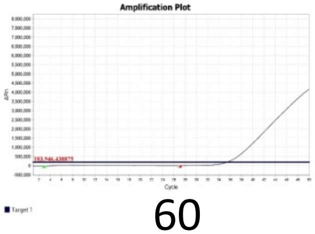

60

Pten-NOLC1-PROSTATE-BLOOD SAMPLE 61-80

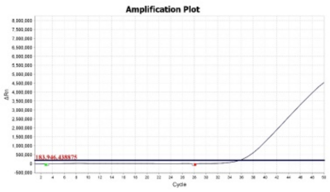

61

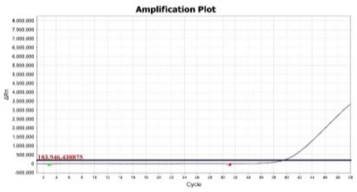

62

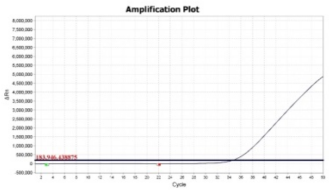

63

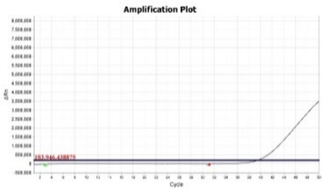

64

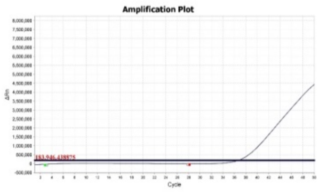

65

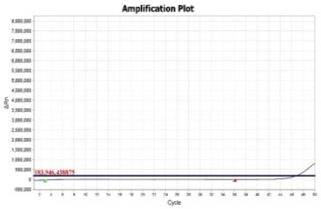

66

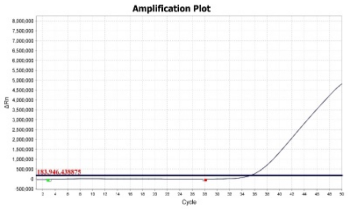

67

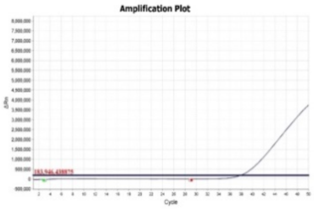

68

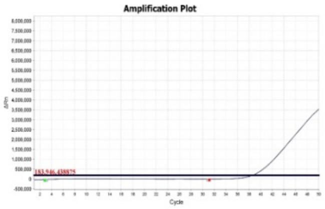

69

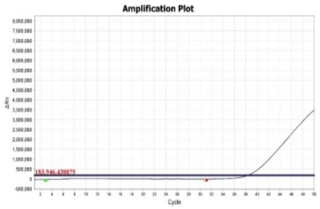

70

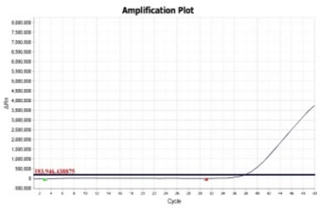

71

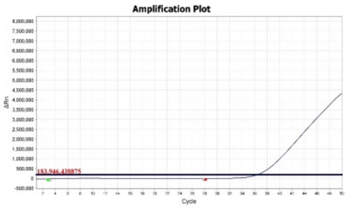

72

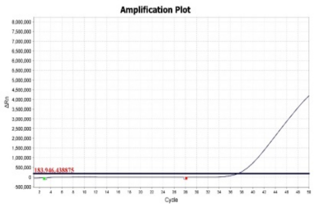

73

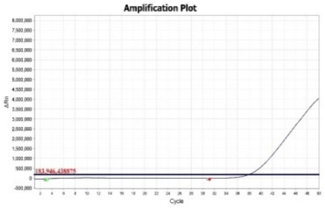

74

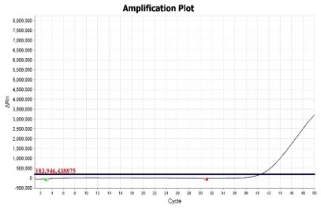

75

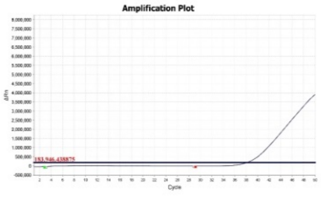

76

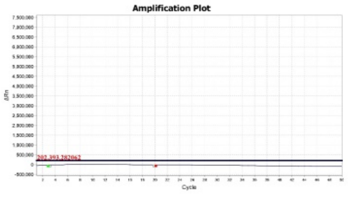

77

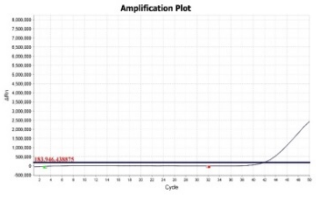

78

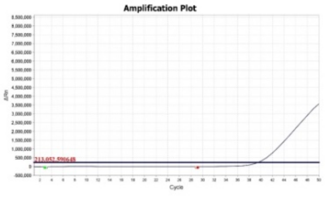

79

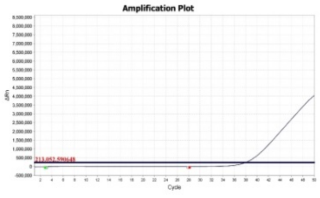

80

Pten-NOLC1-PROSTATE-BLOOD SAMPLE   81-100

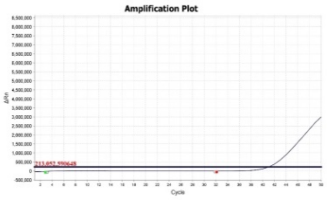

81

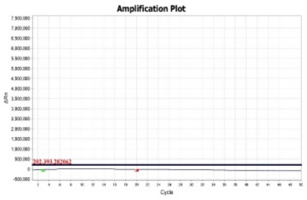

82

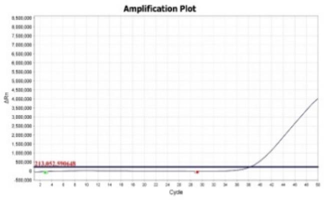

83

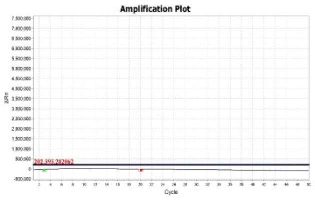

84

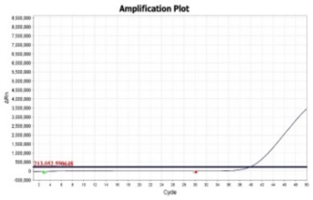

85

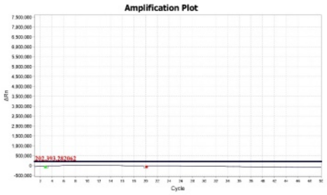

86

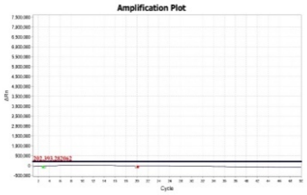

87

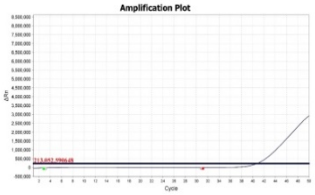

88

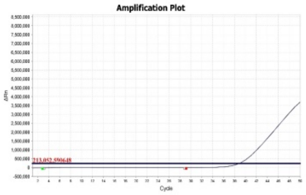

89

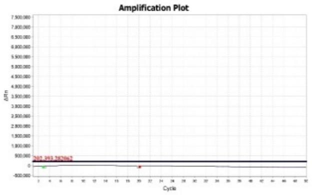

90

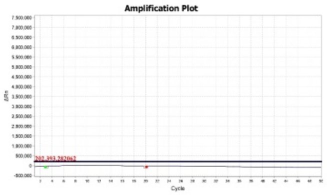

91

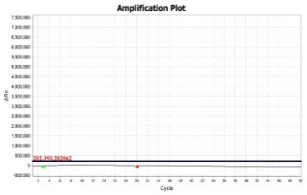

92

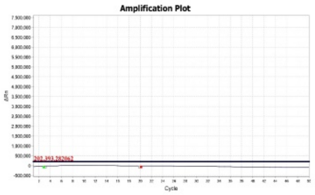

93

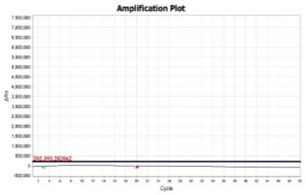

94

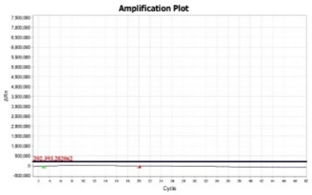

95

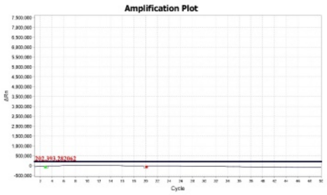

96

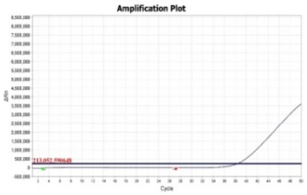

97

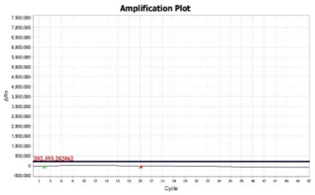

98

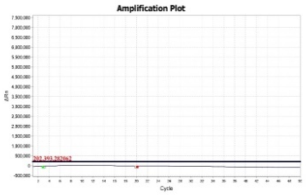

99

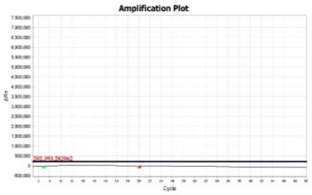

100

Pten-NOLC1-PROSTATE-BLOOD SAMPLE 101-120

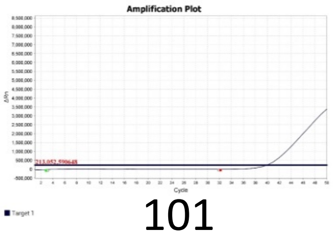

101

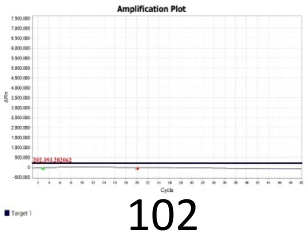

102

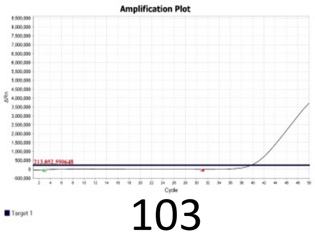

103

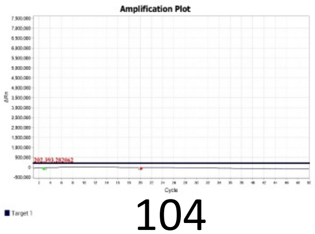

104

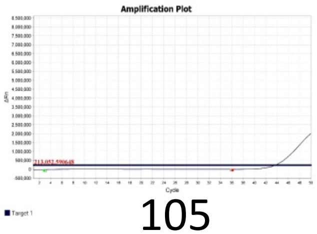

105

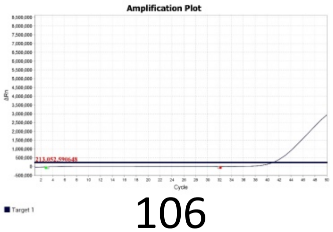

106

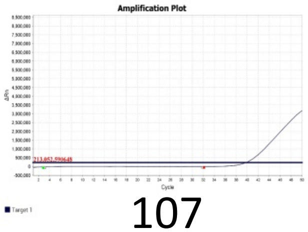

107

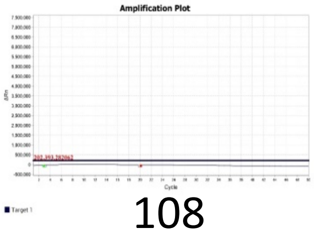

108

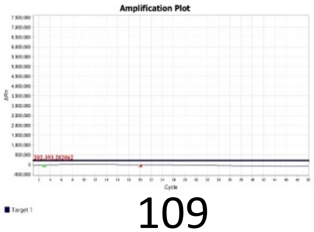

109

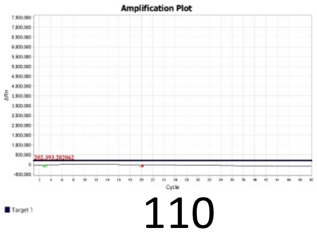

110

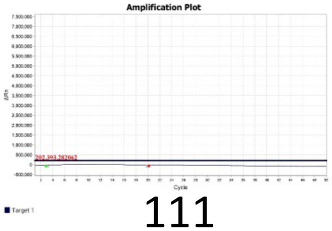

111

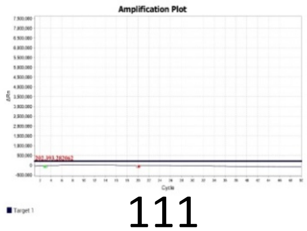

111

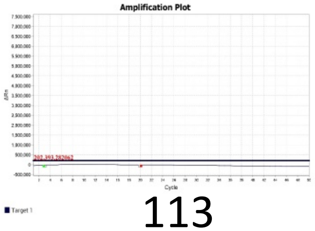

113

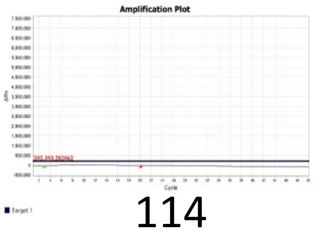

114

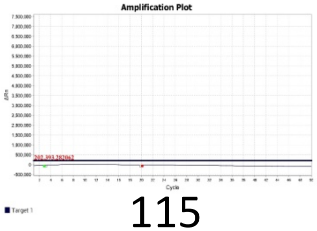

115

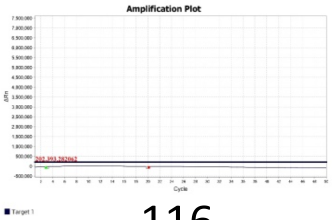

116

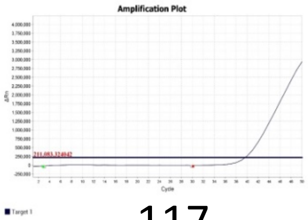

117

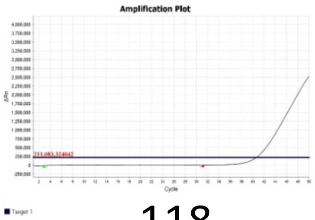

118

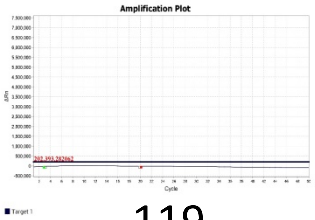

119

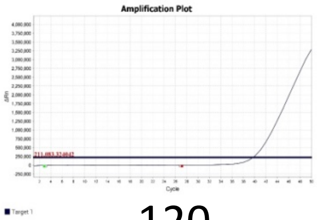

120

Pten-NOLC1-PROSTATE-BLOOD SAMPLE 121-140

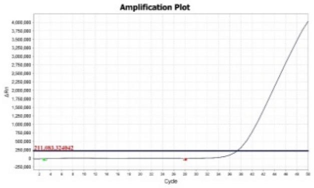

121

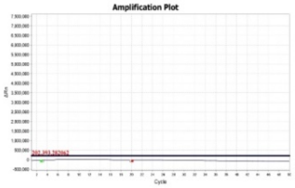

122

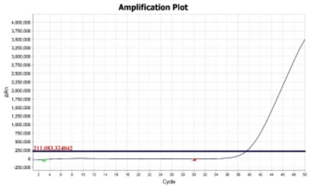

123

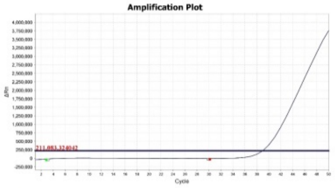

124

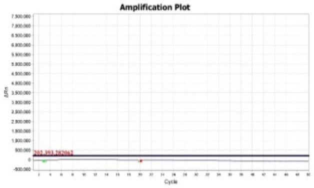

125

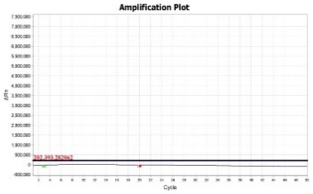

126

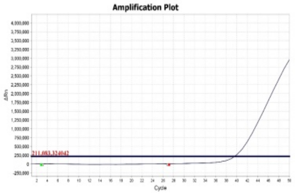

127

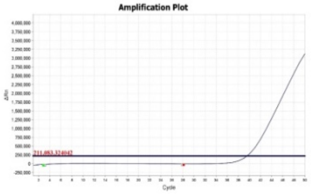

128

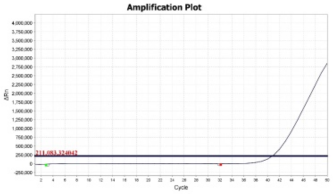

129

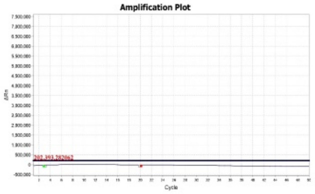

130

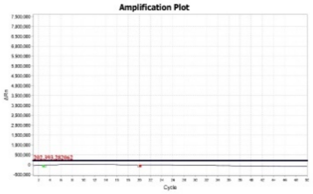

131

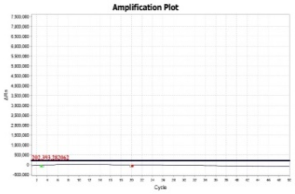

132

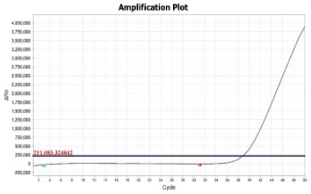

133

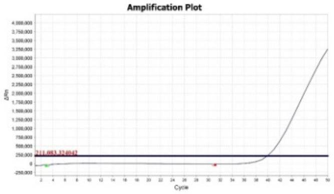

134

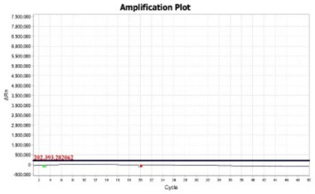

135

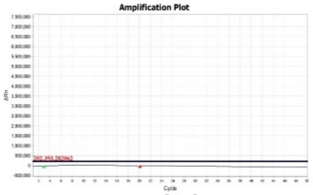

136

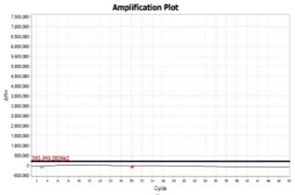

137

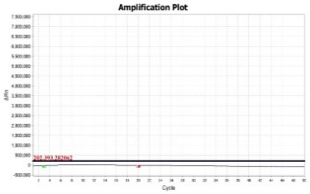

138

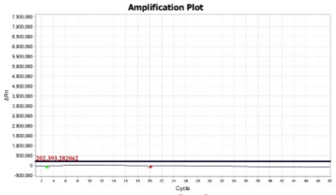

139

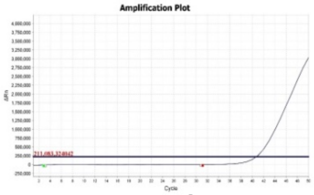

140

Pten-NOLC1-PROSTATE-BLOOD SAMPLE 141-150

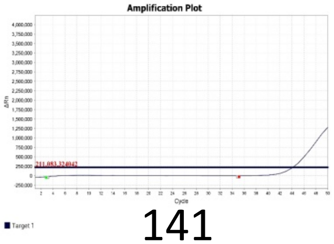

141

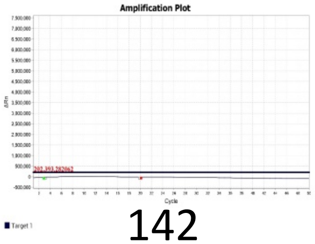

142

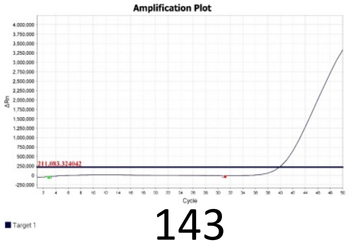

143

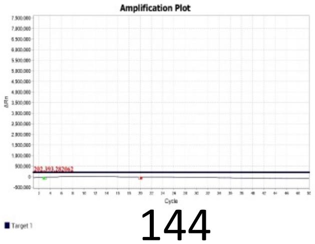

144

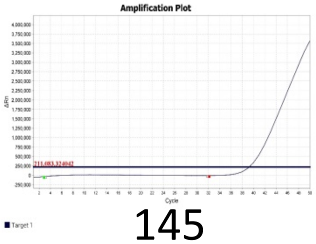

145

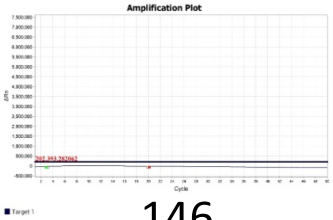

146

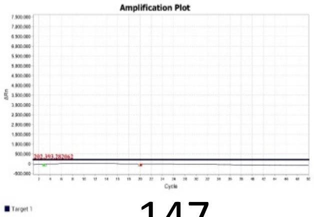

147

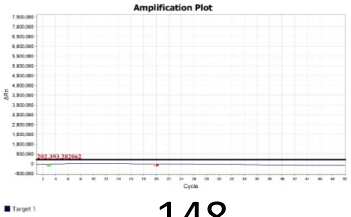

148

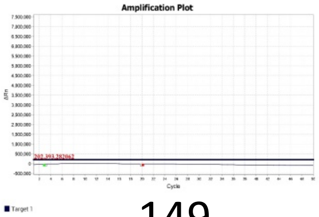

149

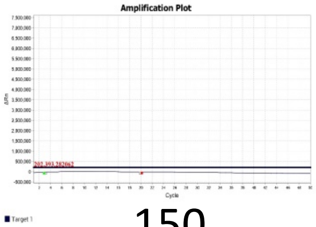

150

Supplemental figure 10

β-ACTIN-PROSTATE-BLOOD SAMPLE   1-20

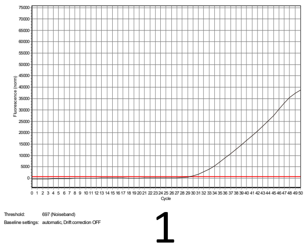
1

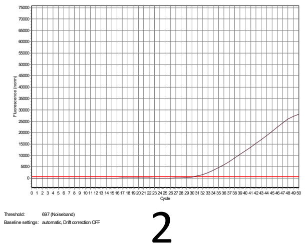
2

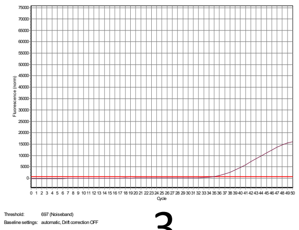
3

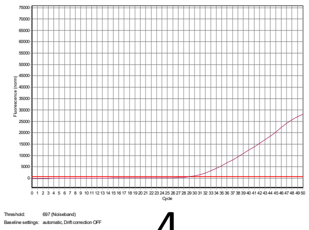
4

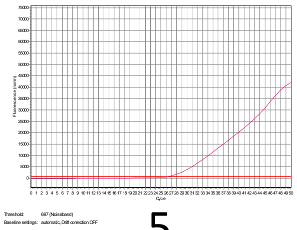
5

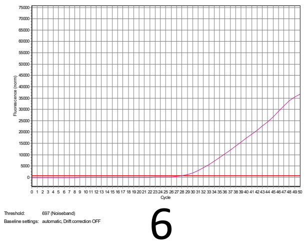
6

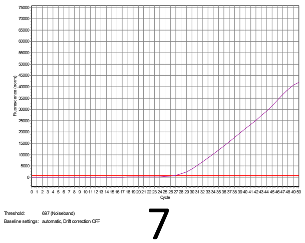
7

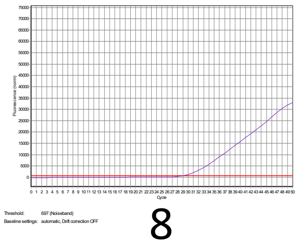
8

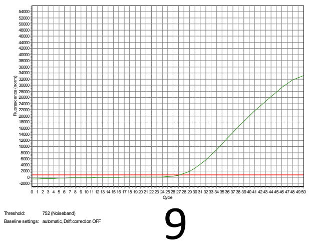
9

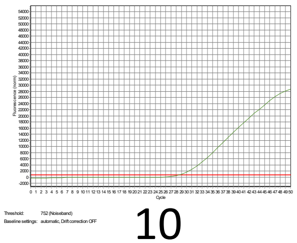
10

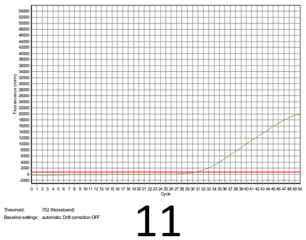
11

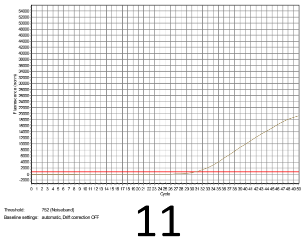
11

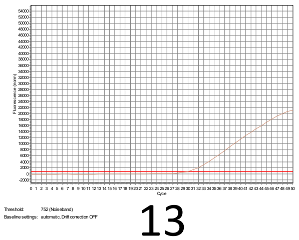
13

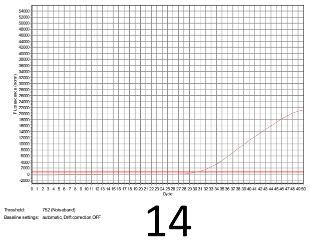
14

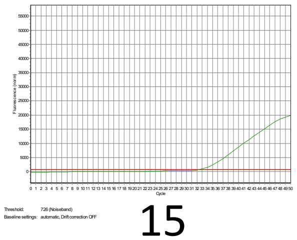
15

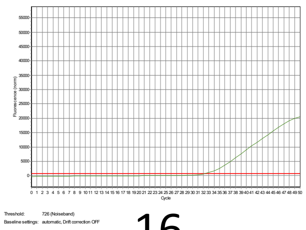
16

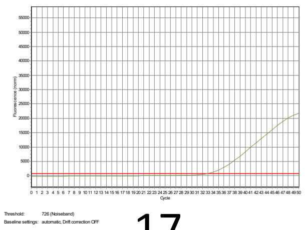
17

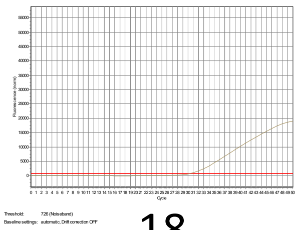
18

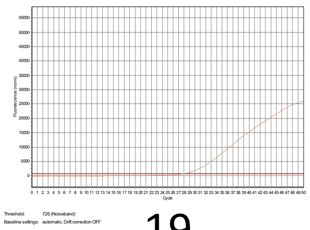
19

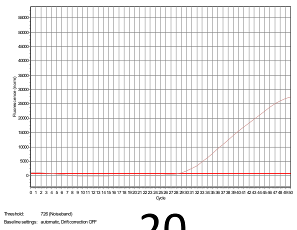
20

β-ACTIN-PROSTATE-BLOOD SAMPLE 21-40

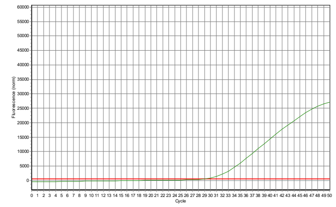

Threshold: 507 (Noiseband)
Baseline settings: automatic, Drift correction OFF

21

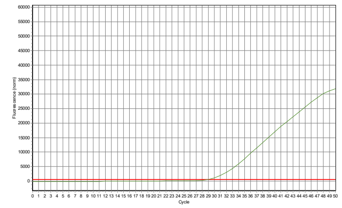

Threshold: 507 (Noiseband)
Baseline settings: automatic, Drift correction OFF

22

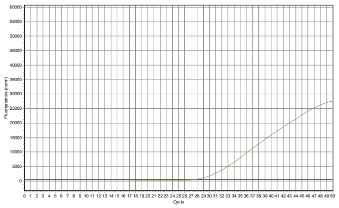

Threshold: 507 (Noiseband)
Baseline settings: automatic, Drift correction OFF

23

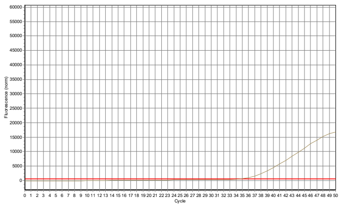

Threshold: 507 (Noiseband)
Baseline settings: automatic, Drift correction OFF

24

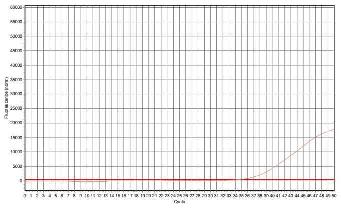

Threshold: 507 (Noiseband)
Baseline settings: automatic, Drift correction OFF

25

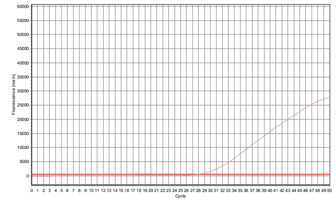

Threshold: 507 (Noiseband)
Baseline settings: automatic, Drift correction OFF

26

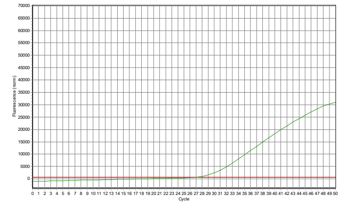

Threshold: 615 (Noiseband)
Baseline settings: automatic, Drift correction OFF

27

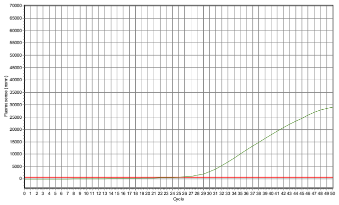

Threshold: 615 (Noiseband)
Baseline settings: automatic, Drift correction OFF

28

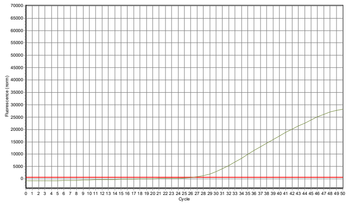

Threshold: 615 (Noiseband)
Baseline settings: automatic, Drift correction OFF

29

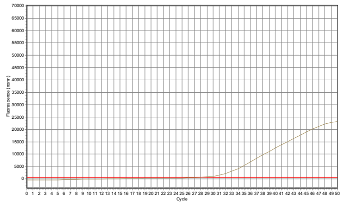

Threshold: 615 (Noiseband)
Baseline settings: automatic, Drift correction OFF

30

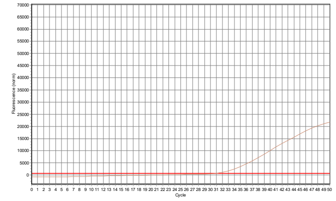

Threshold: 615 (Noiseband)
Baseline settings: automatic, Drift correction OFF

31

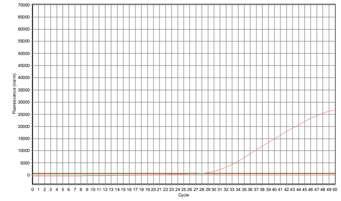

Threshold: 615 (Noiseband)
Baseline settings: automatic, Drift correction OFF

32

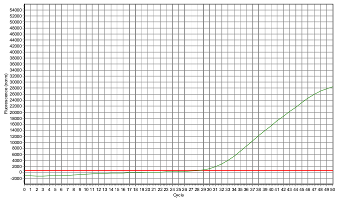

Threshold: 706 (Noiseband)
Baseline settings: automatic, Drift correction OFF

33

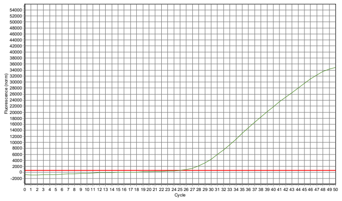

Threshold: 706 (Noiseband)
Baseline settings: automatic, Drift correction OFF

34

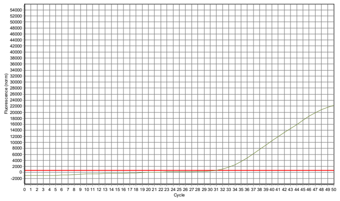

Threshold: 706 (Noiseband)
Baseline settings: automatic, Drift correction OFF

35

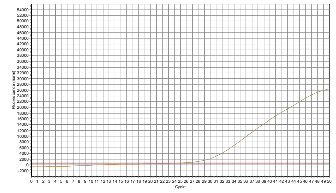

Threshold: 706 (Noiseband)
Baseline settings: automatic, Drift correction OFF

36

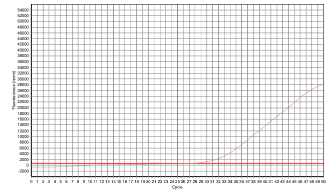

Threshold: 706 (Noiseband)
Baseline settings: automatic, Drift correction OFF

37

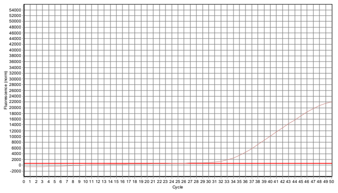

Threshold: 706 (Noiseband)
Baseline settings: automatic, Drift correction OFF

38

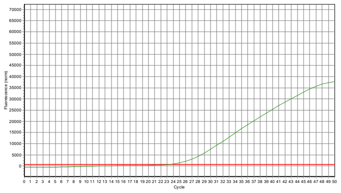

Threshold: 781 (Noiseband)
Baseline settings: automatic, Drift correction OFF

39

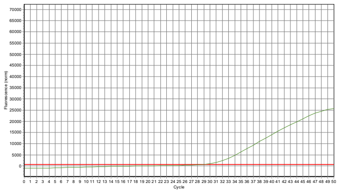

Threshold: 781 (Noiseband)
Baseline settings: automatic, Drift correction OFF

40

β-ACTIN-PROSTATE-BLOOD SAMPLE 41-60

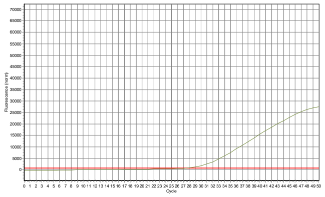

41

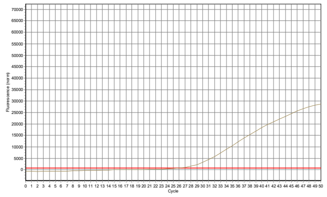

42

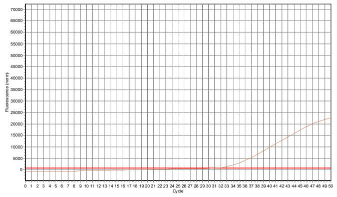

43

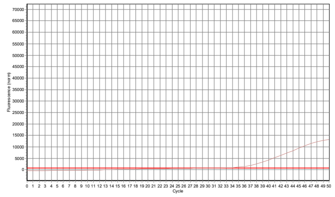

44

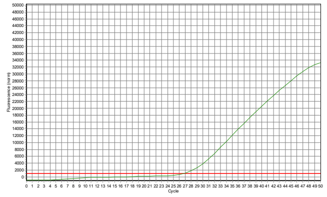

45

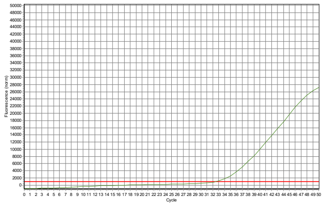

46

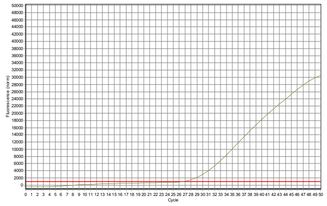

47

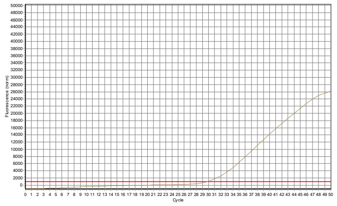

47

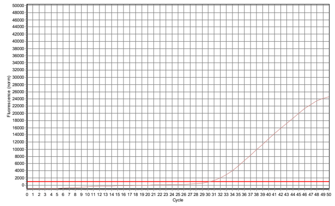

49

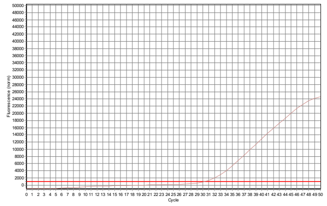

50

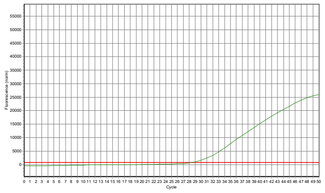

51

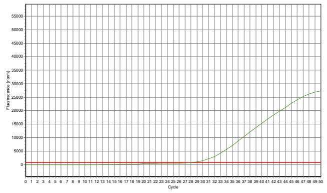

52

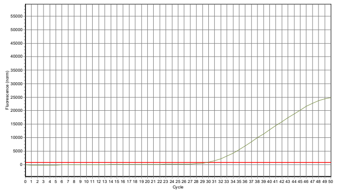

53

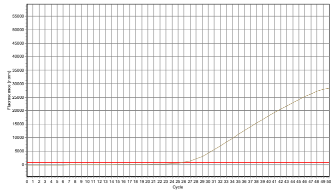

54

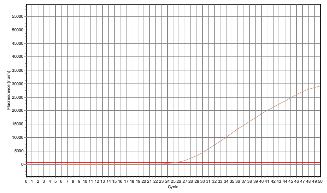

55

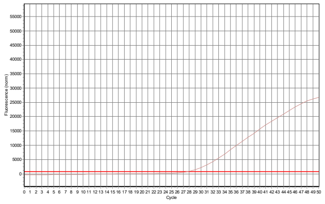

56

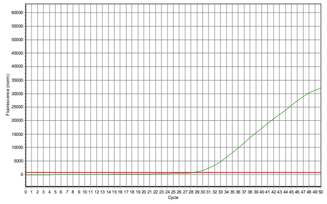

57

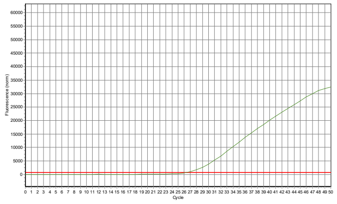

58

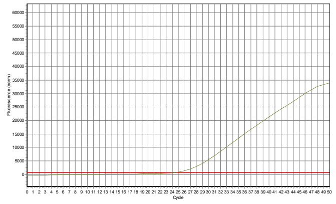

59

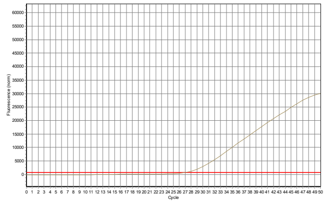

60

β-ACTIN-PROSTATE-BLOOD SAMPLE 61-80

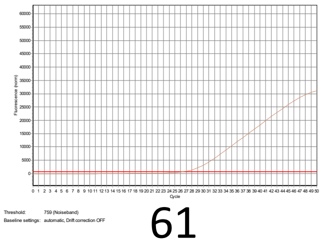
61

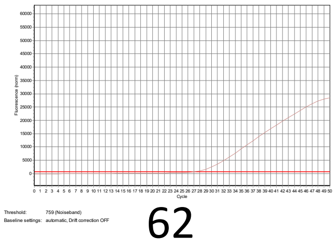
62

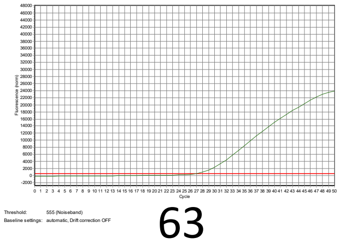
63

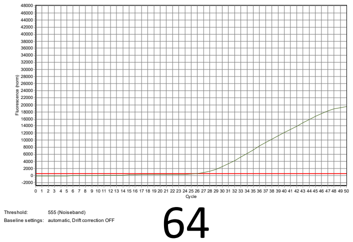
64

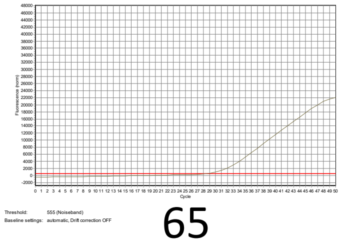
65

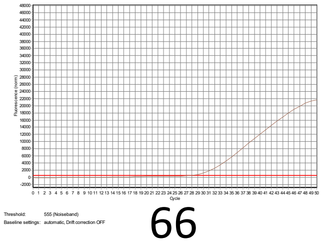
66

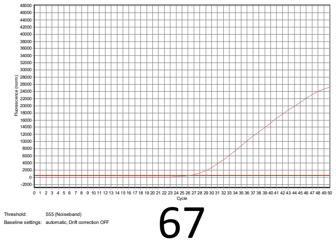
67

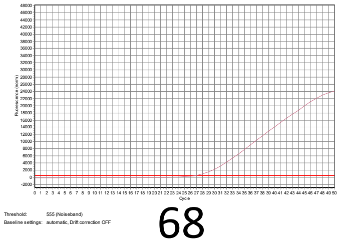
68

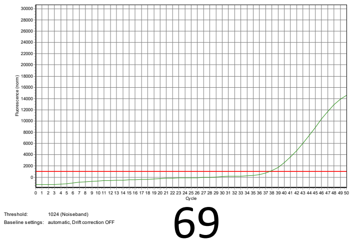
69

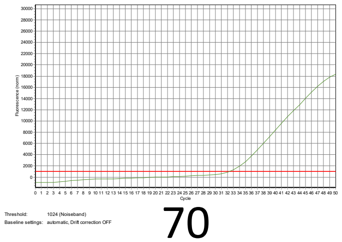
70

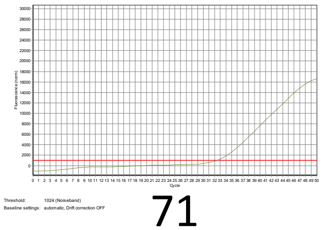
71

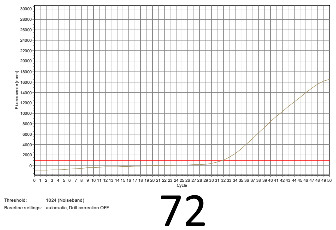
72

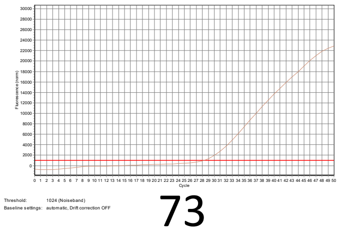
73

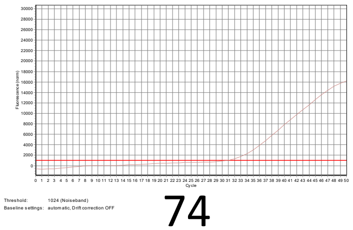
74

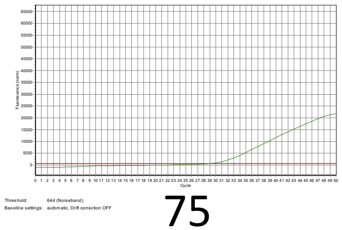
75

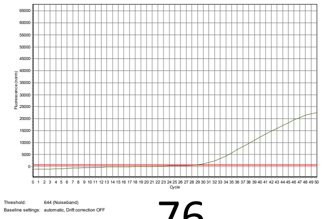
76

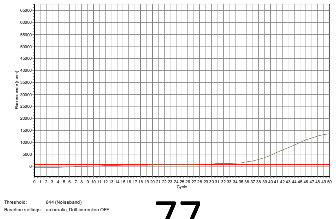
77

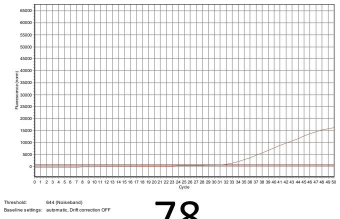
78

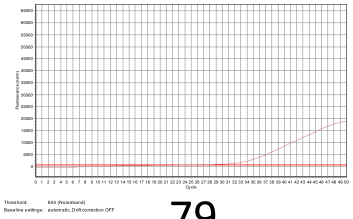
79

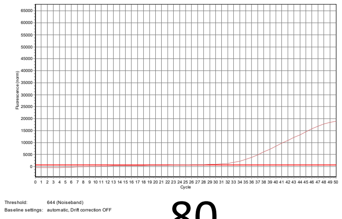
80

β-ACTIN-PROSTATE-BLOOD SAMPLE 81-100

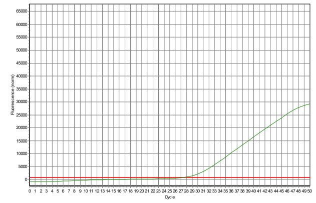

Threshold: 858 (Noiseband)
Baseline settings: automatic, Drift correction OFF

81

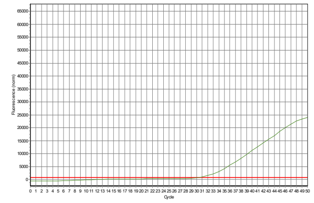

Threshold: 858 (Noiseband)
Baseline settings: automatic, Drift correction OFF

82

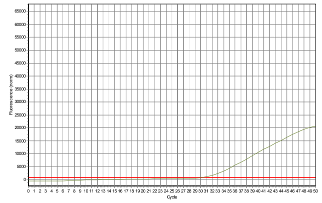

Threshold: 858 (Noiseband)
Baseline settings: automatic, Drift correction OFF

83

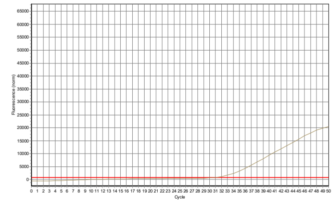

Threshold: 858 (Noiseband)
Baseline settings: automatic, Drift correction OFF

84

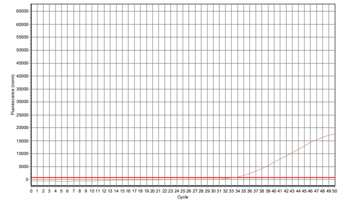

Threshold: 858 (Noiseband)
Baseline settings: automatic, Drift correction OFF

85

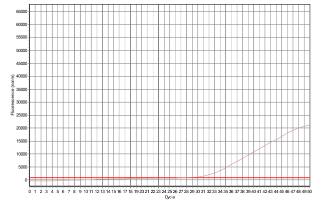

Threshold: 858 (Noiseband)
Baseline settings: automatic, Drift correction OFF

86

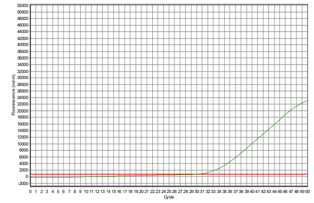

Threshold: 757 (Noiseband)
Baseline settings: automatic, Drift correction OFF

87

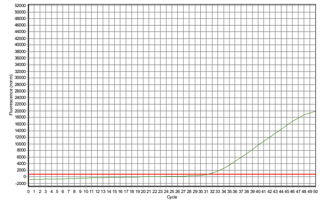

Threshold: 757 (Noiseband)
Baseline settings: automatic, Drift correction OFF

88

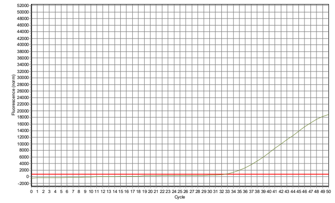

Threshold: 757 (Noiseband)
Baseline settings: automatic, Drift correction OFF

89

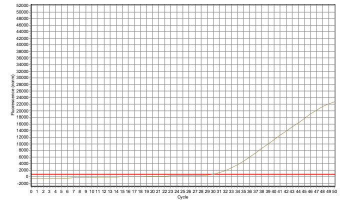

Threshold: 757 (Noiseband)
Baseline settings: automatic, Drift correction OFF

90

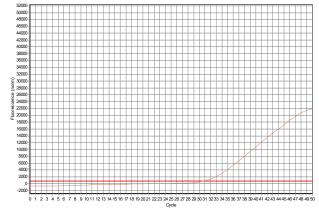

Threshold: 757 (Noiseband)
Baseline settings: automatic, Drift correction OFF

91

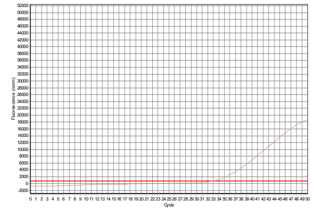

Threshold: 757 (Noiseband)
Baseline settings: automatic, Drift correction OFF

92

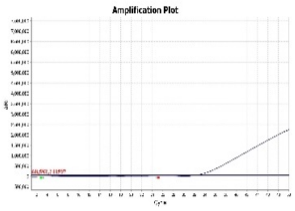

93

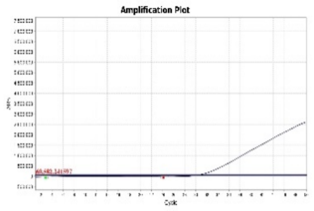

94

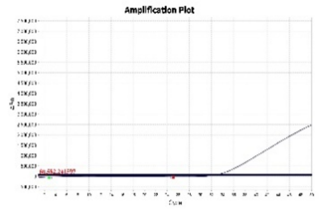

95

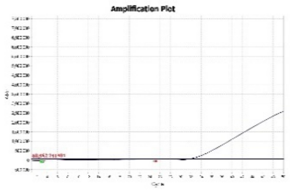

96

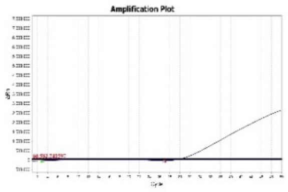

97

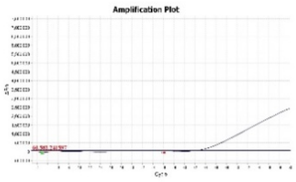

98

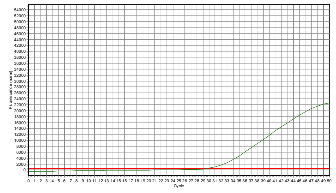

Threshold: 577 (Noiseband)
Baseline settings: automatic, Drift correction OFF

99

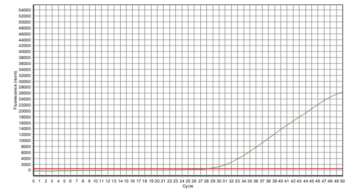

Threshold: 577 (Noiseband)
Baseline settings: automatic, Drift correction OFF

100

β-ACTIN-PROSTATE-BLOOD SAMPLE 101-120

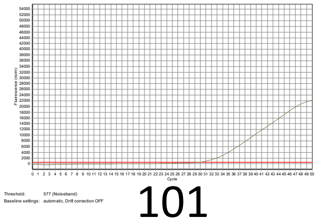

101

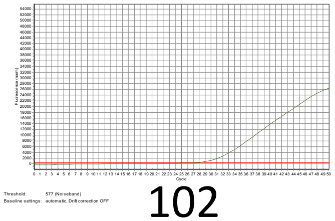

102

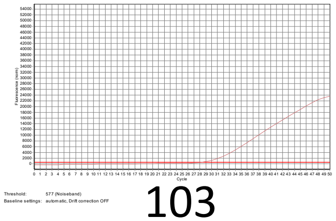

103

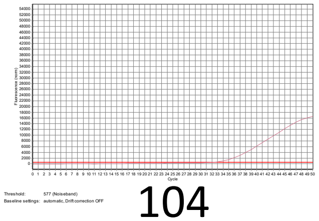

104

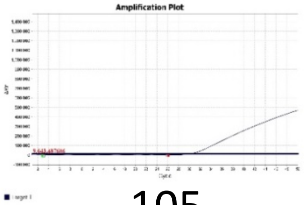

105

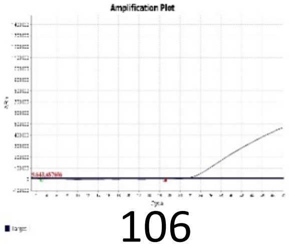

106

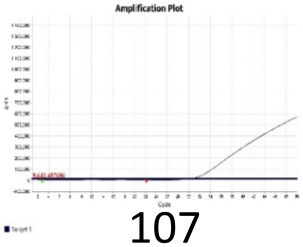

107

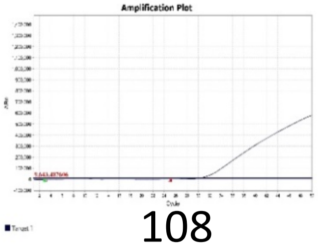

108

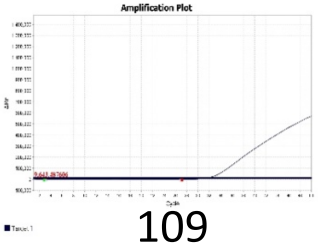

109

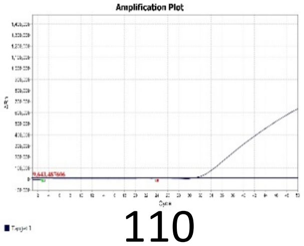

110

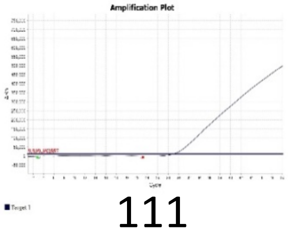

111

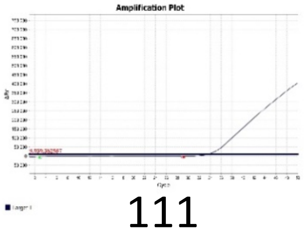

111

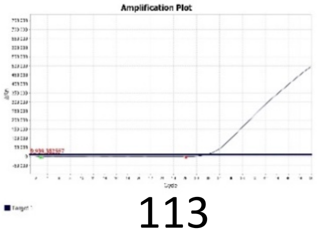

113

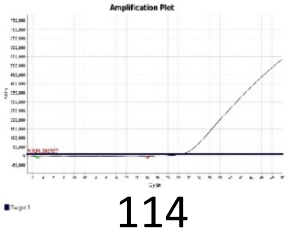

114

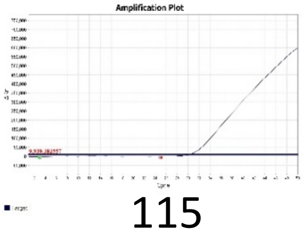

115

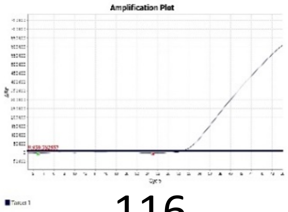

116

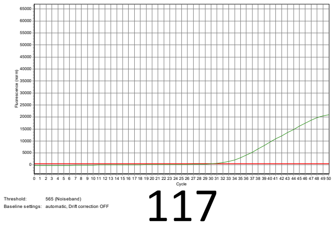

117

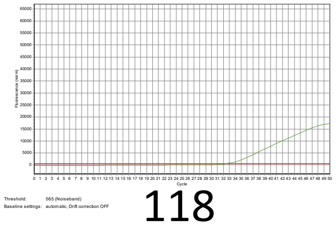

118

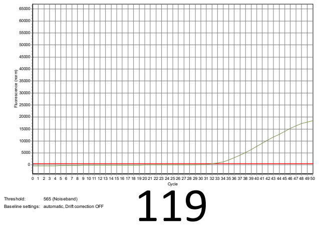

119

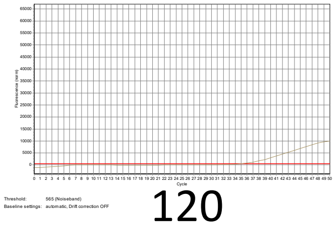

120

β-ACTIN-PROSTATE-BLOOD SAMPLE 121-140

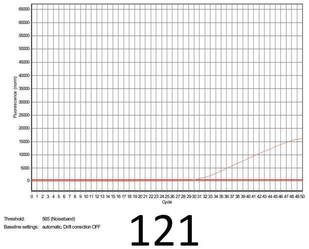
121

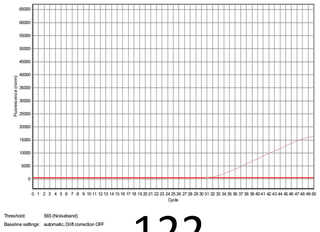
122

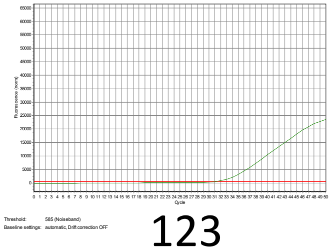
123

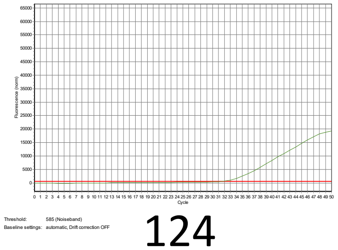
124

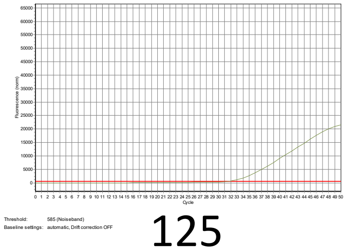
125

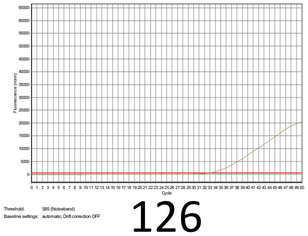
126

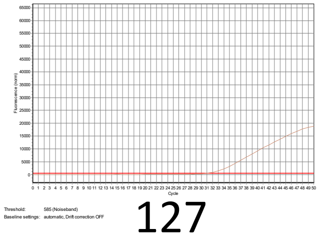
127

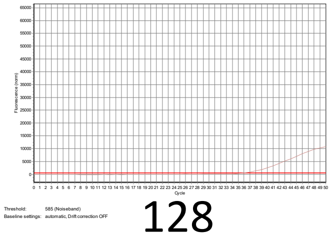
128

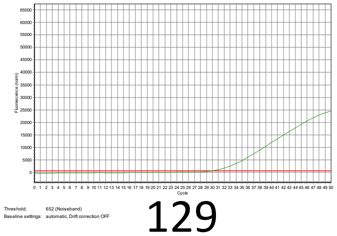
129

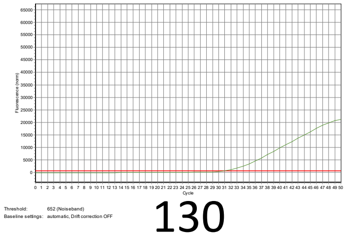
130

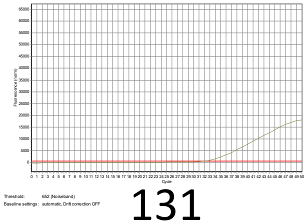
131

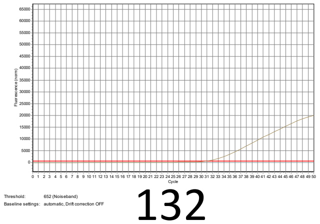
132

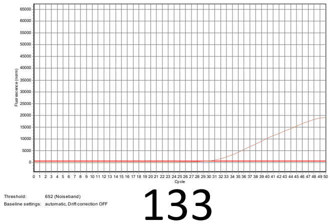
133

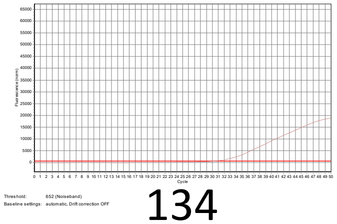
134

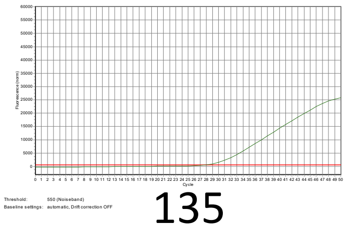
135

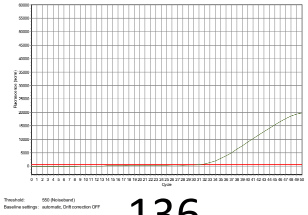
136

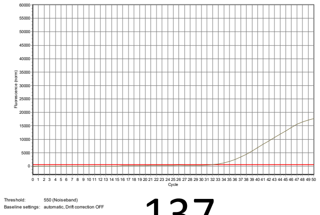
137

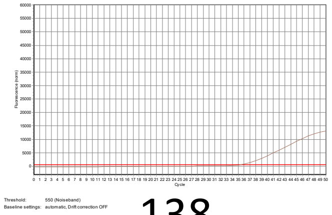
138

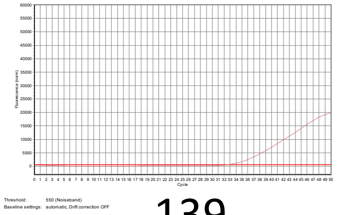
139

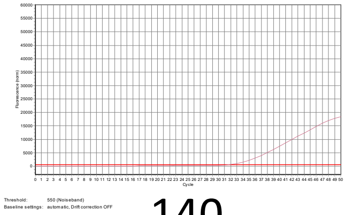
140

β-ACTIN-PROSTATE-BLOOD SAMPLE 141-150

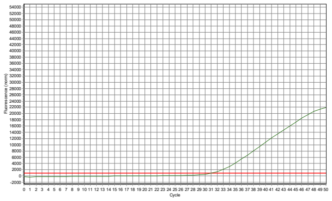

Threshold: 896 (Adjusted manually)
Baseline settings: automatic, Drift correction OFF

141

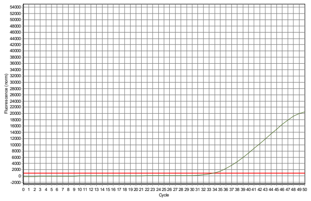

Threshold: 896 (Adjusted manually)
Baseline settings: automatic, Drift correction OFF

142

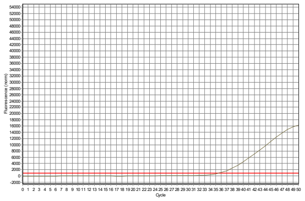

Threshold: 896 (Adjusted manually)
Baseline settings: automatic, Drift correction OFF

143

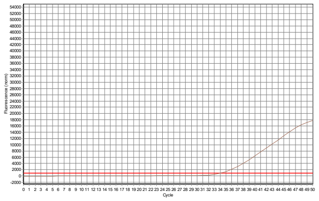

Threshold: 896 (Adjusted manually)
Baseline settings: automatic, Drift correction OFF

144

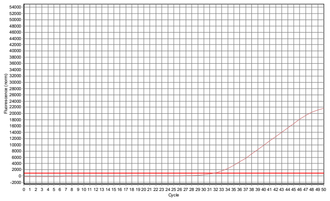

Threshold: 896 (Adjusted manually)
Baseline settings: automatic, Drift correction OFF

145

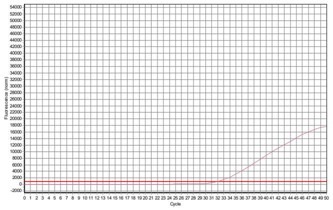

Threshold: 896 (Adjusted manually)
Baseline settings: automatic, Drift correction OFF

146

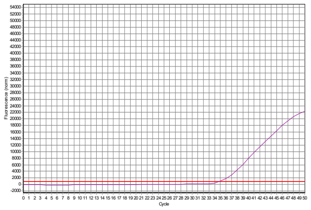

Threshold: 896 (Adjusted manually)
Baseline settings: automatic, Drift correction OFF

147

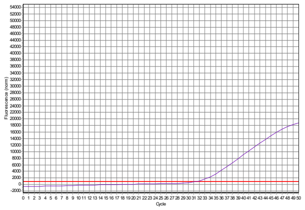

Threshold: 896 (Adjusted manually)
Baseline settings: automatic, Drift correction OFF

148

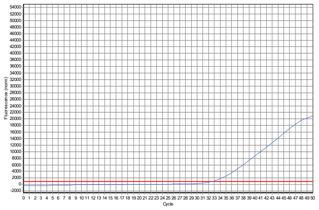

Threshold: 896 (Adjusted manually)
Baseline settings: automatic, Drift correction OFF

149

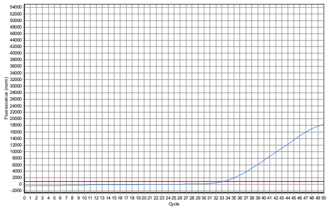

Threshold: 896 (Adjusted manually)
Baseline settings: automatic, Drift correction OFF

150

Supplemental figure 11

KDM4B-AC011523.2

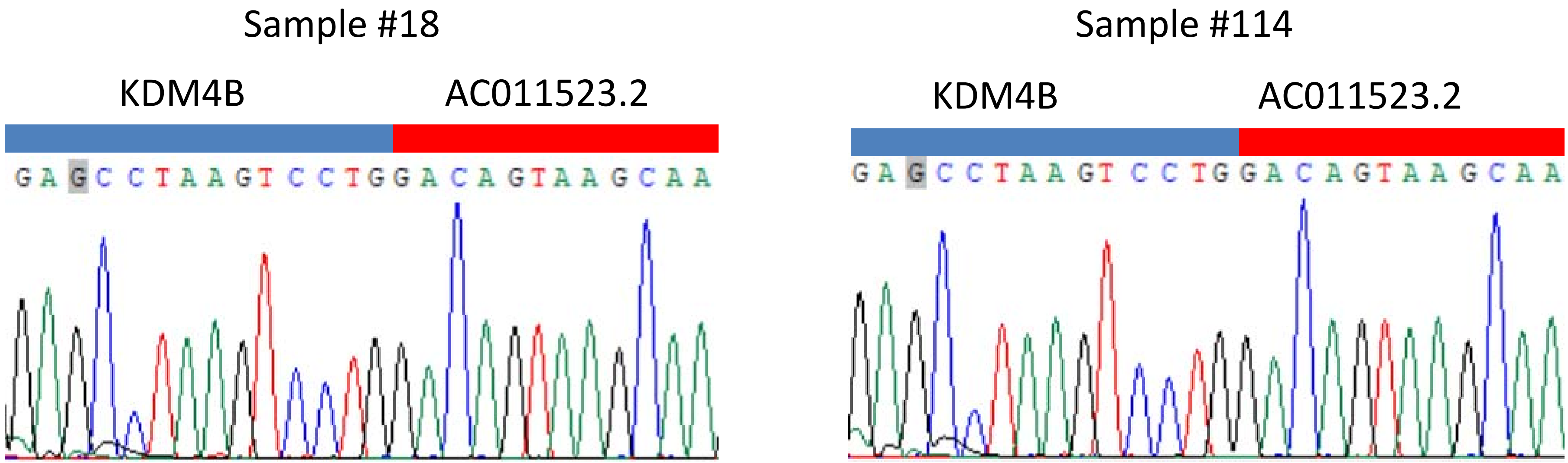

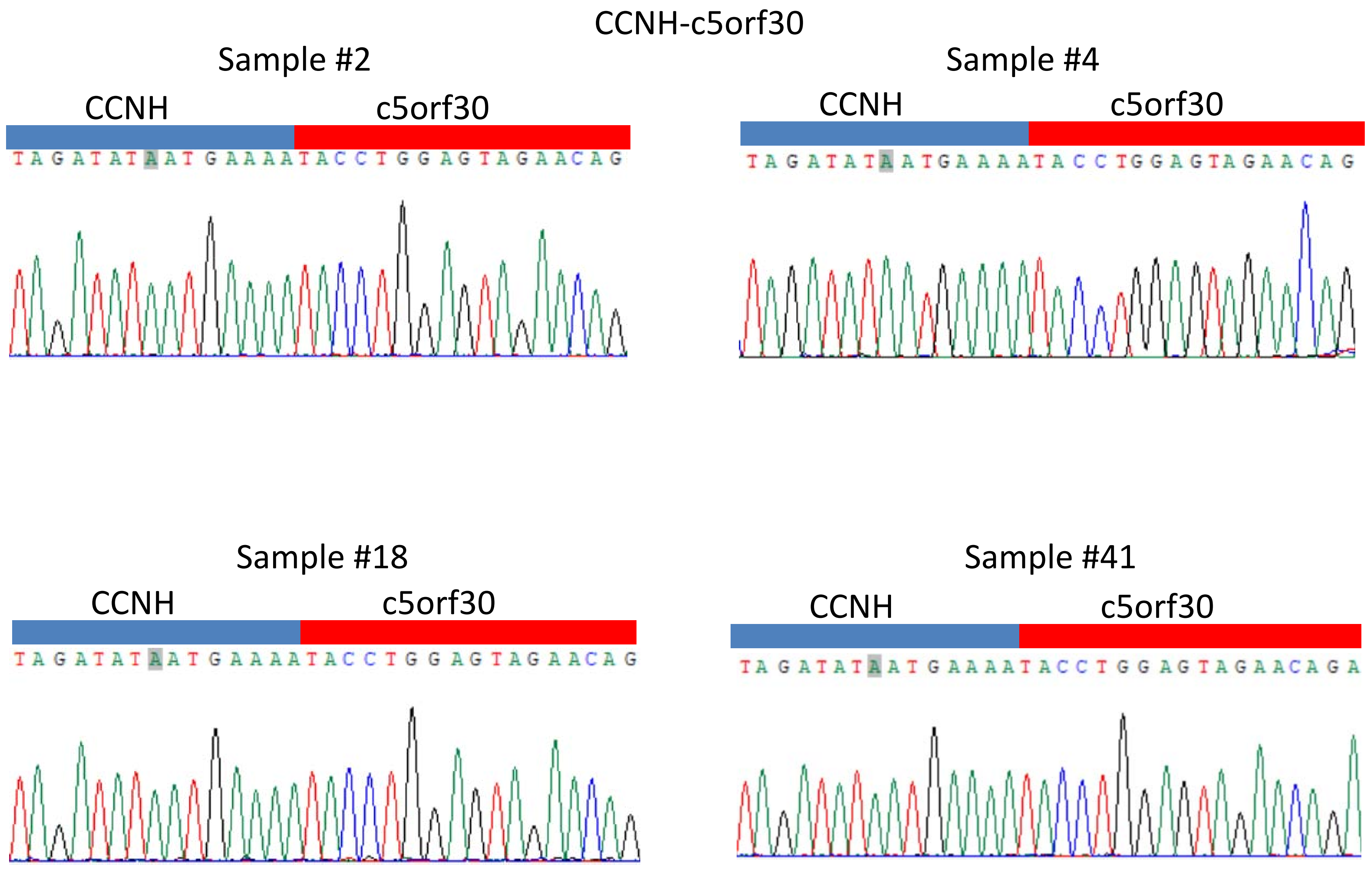
CCNH-c5orf30
Sample #2
CCNH
c5orf30
TAGATATAATGAAAATACCTGGAGTAGAACAG
Sample #4
CCNH
c5orf30
TAGATATAATGAAAATACCTGGAGTAGAACAG
Sample #18
CCNH
c5orf30
TAGATATAATGAAAATACCTGGAGTAGAACAG
Sample #41
CCNH
c5orf30
TAGATATAATGAAAATACCTGGAGTAGAACAGA

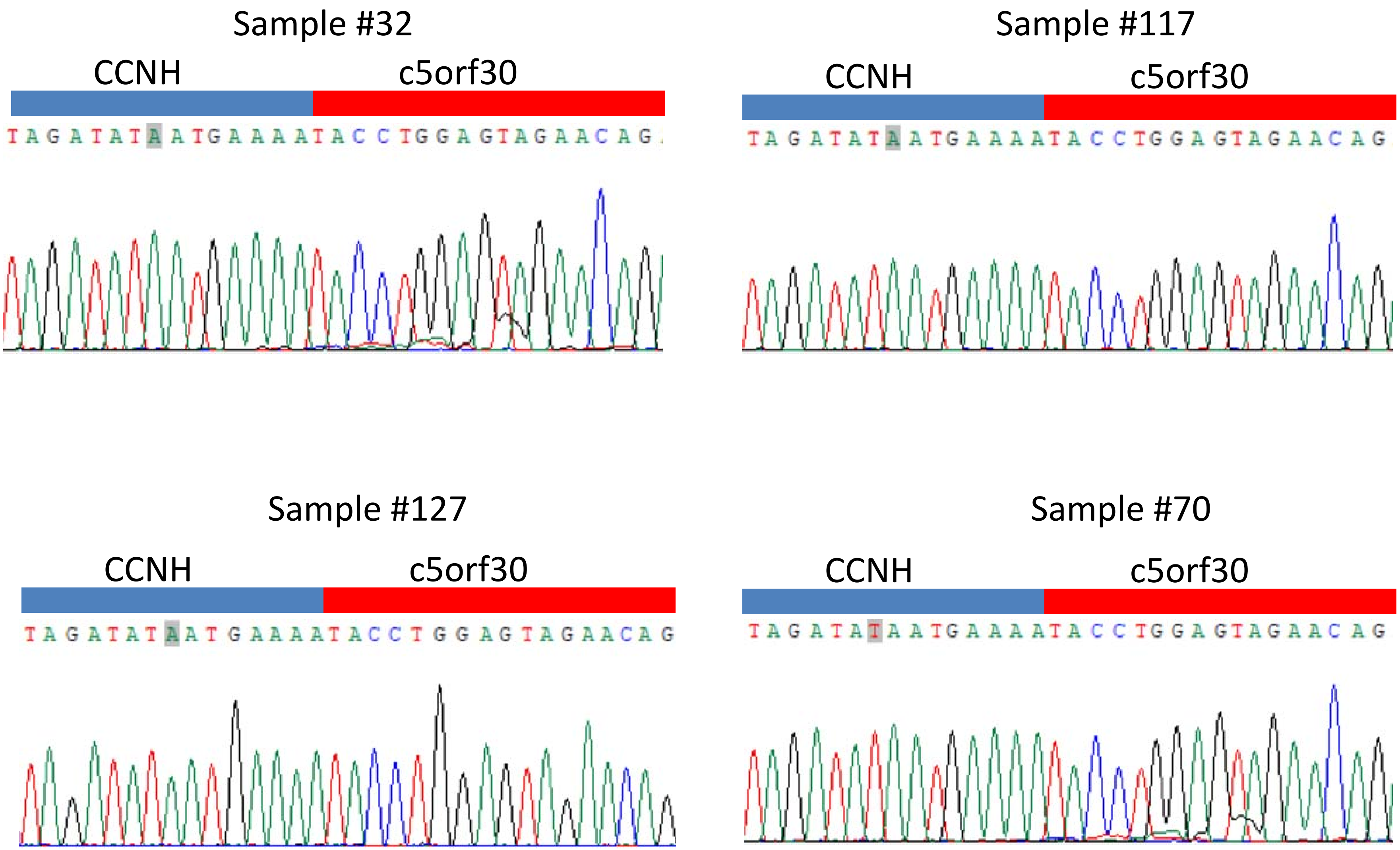
Sample #32
CCNH
c5orf30
TAGATATAATGAAAATACCTGGAGTAGAACAG
Sample #117
CCNH
c5orf30
TAGATATAATGAAAATACCTGGAGTAGAACAG
Sample #127
CCNH
c5orf30
TAGATATAATGAAAATACCTGGAGTAGAACAG
Sample #70
CCNH
c5orf30
TAGATATAATGAAAATACCTGGAGTAGAACAG

mTOR-TP53BP1

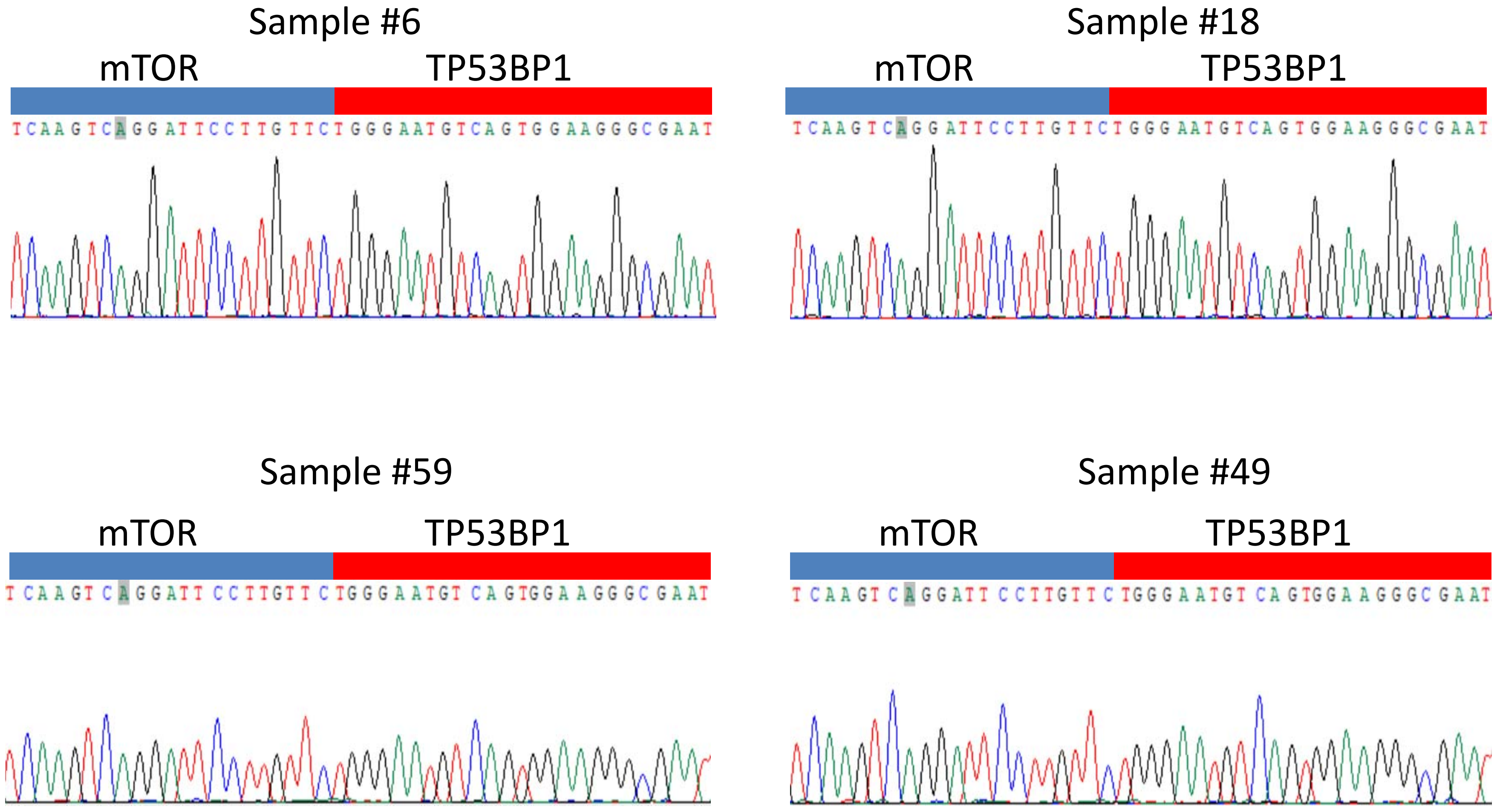

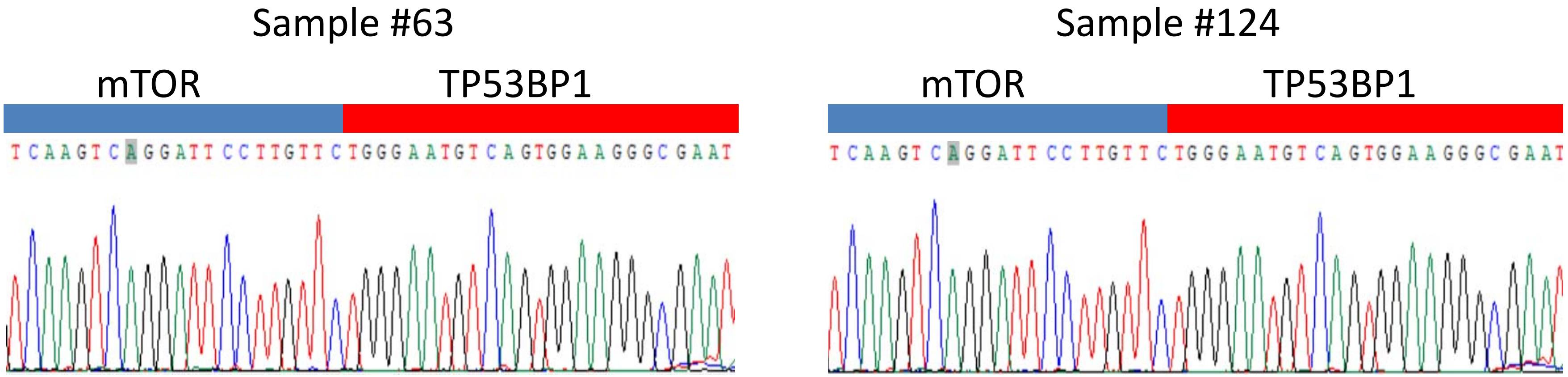
Sample #63
mTOR
TP53BP1
TCAAGTCAGGATTCCTTGTTCTGGGAATGTCAGTGGAAGGGCGAAT
Sample #124
mTOR
TP53BP1
TCAAGTCAGGATTCCTTGTTCTGGGAATGTCAGTGGAAGGGCGAAT

MAN2A1-FER

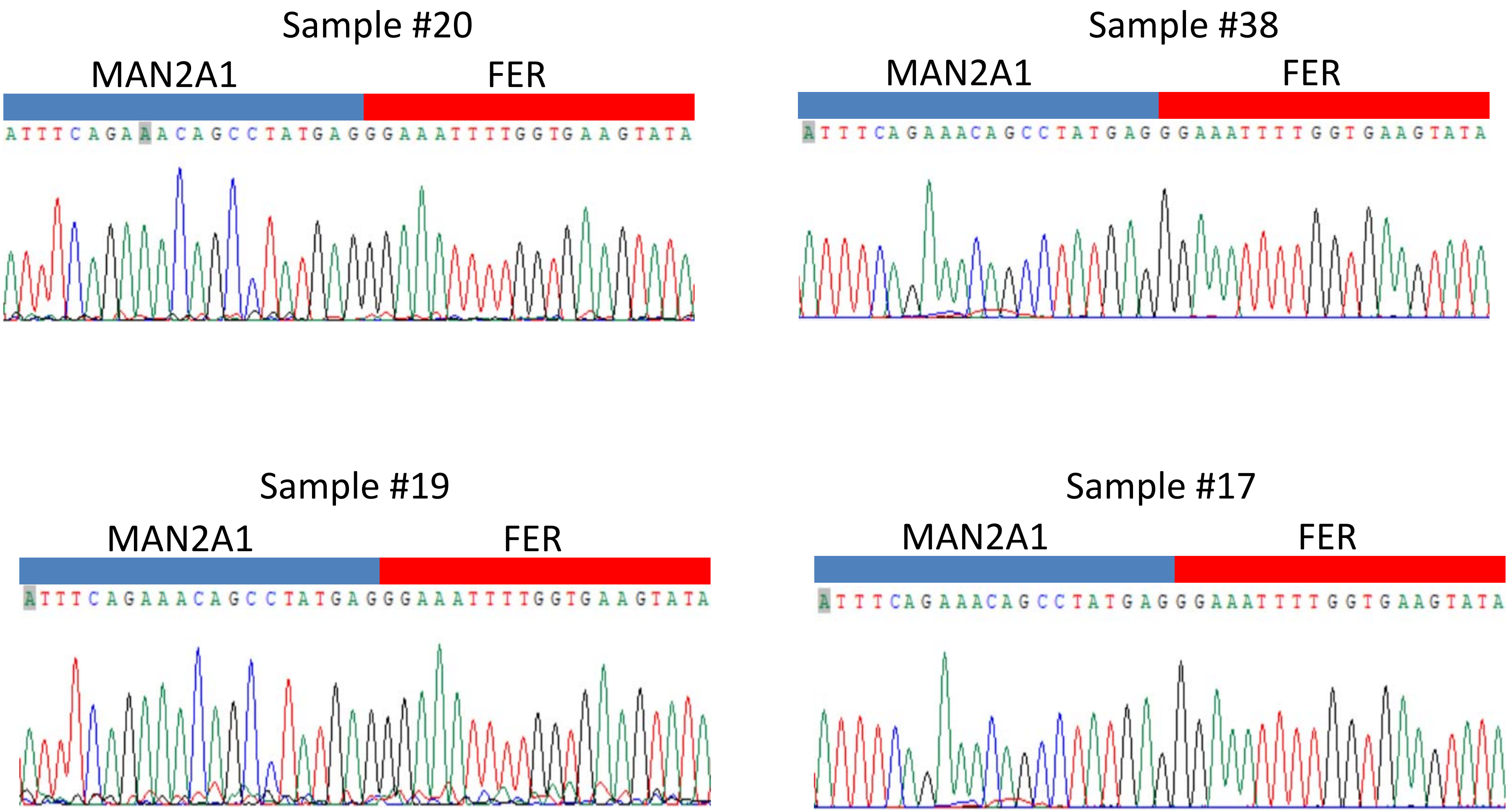
Sample #20
MAN2A1
FER
ATTTCAGAAACAGCCTATGAGGGAAATTTTGGTGAAGTATA
Sample #38
MAN2A1
FER
ATTTCAGAAACAGCCTATGAGGGAAATTTTGGTGAAGTATA
Sample #19
MAN2A1
FER
ATTTCAGAAACAGCCTATGAGGGAAATTTTGGTGAAGTATA
Sample #17
MAN2A1
FER
ATTTCAGAAACAGCCTATGAGGGAAATTTTGGTGAAGTATA

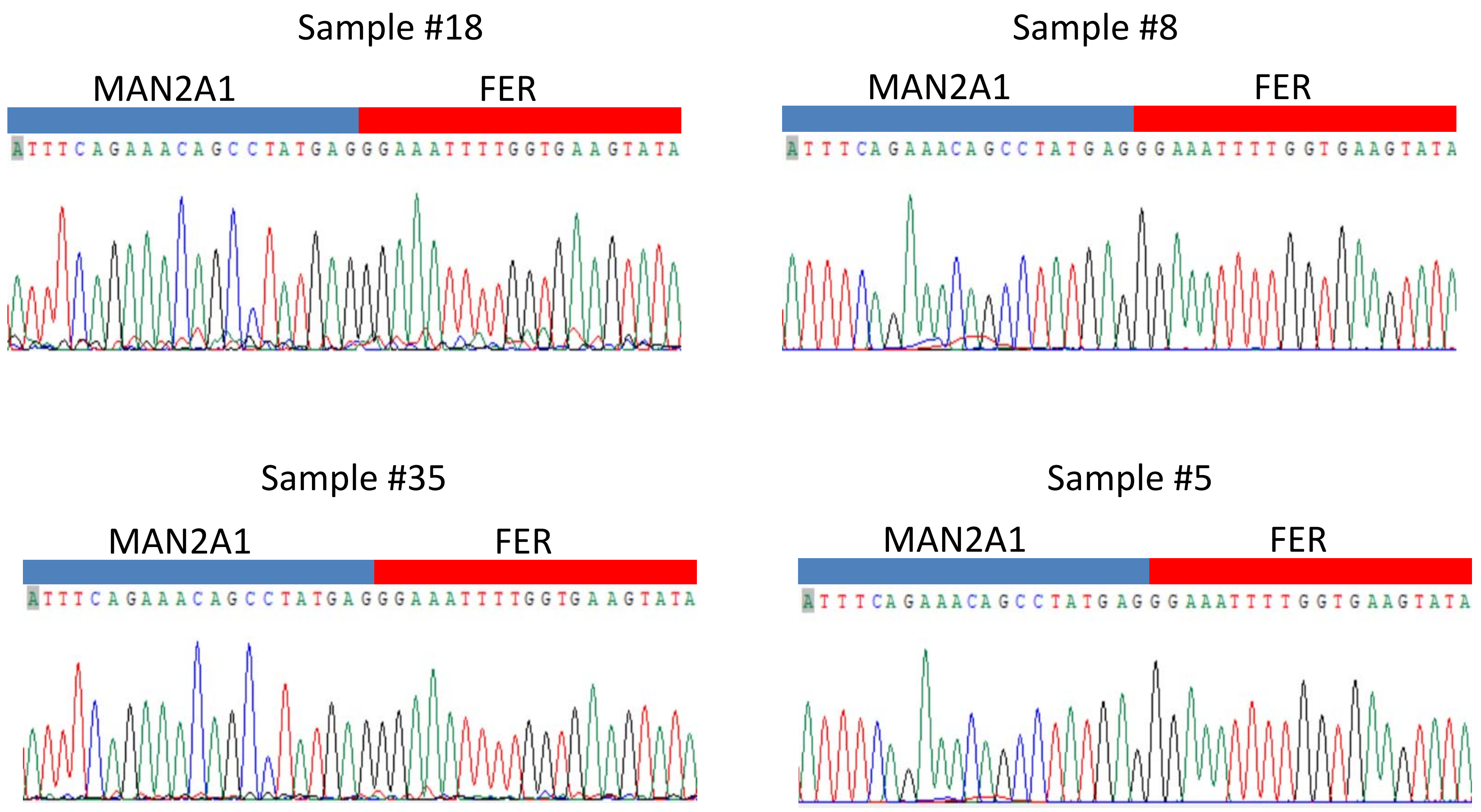

Sample #18
MAN2A1
FER
ATTTCAGAAACAGCCTATGAGGGAAATTTTGGTGAAGTATA
Sample #8
MAN2A1
FER
ATTTCAGAAACAGCCTATGAGGGAAATTTTGGTGAAGTATA
Sample #35
MAN2A1
FER
ATTTCAGAAACAGCCTATGAGGGAAATTTTGGTGAAGTATA
Sample #5
MAN2A1
FER
ATTTCAGAAACAGCCTATGAGGGAAATTTTGGTGAAGTATA

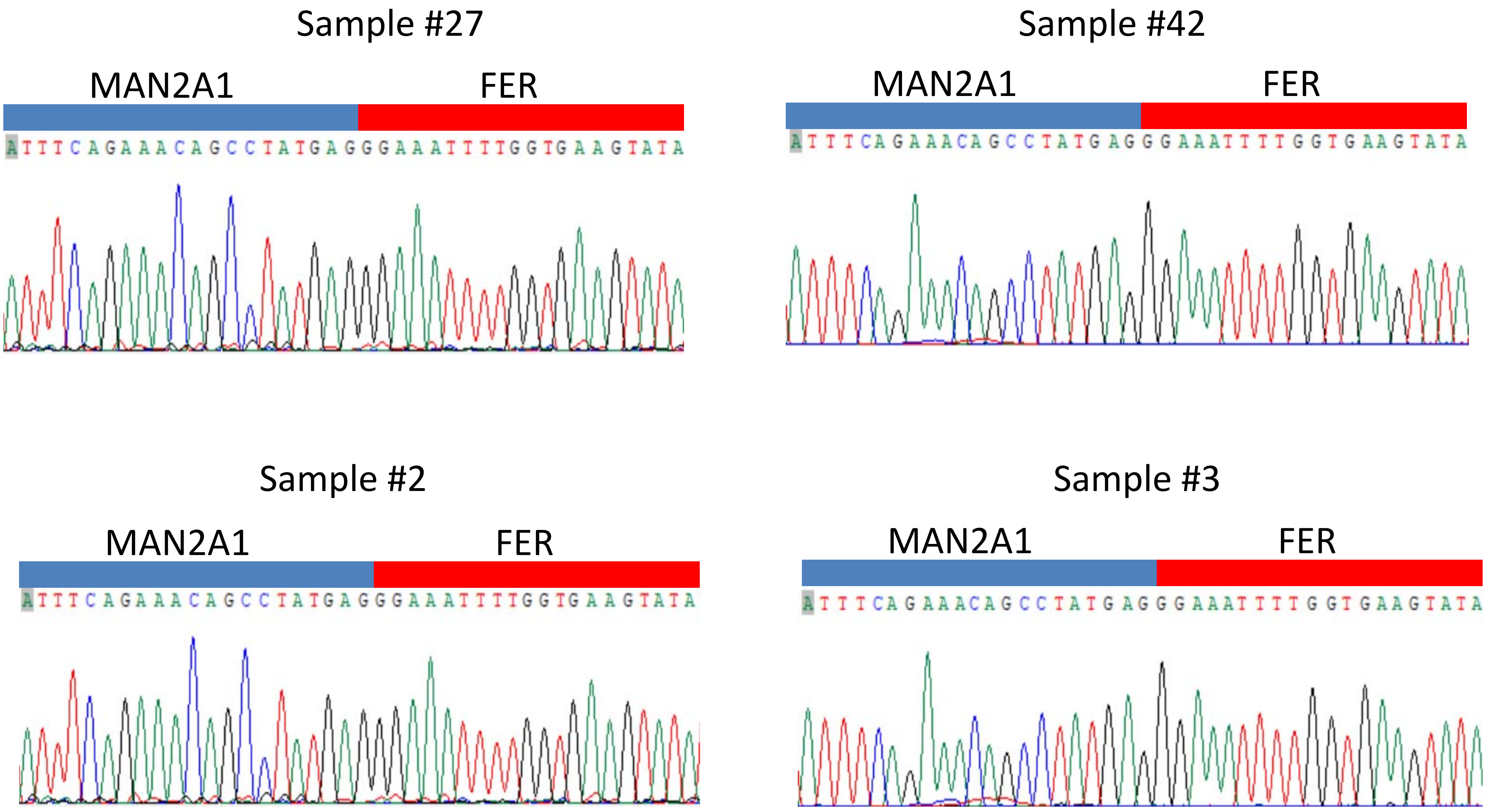
Sample #27
MAN2A1
FER
ATTTCAGAAACAGCCTATGAGGGAAATTTTGGTGAAGTATA
Sample #42
MAN2A1
FER
ATTTCAGAAACAGCCTATGAGGGAAATTTTGGTGAAGTATA
Sample #2
MAN2A1
FER
ATTTCAGAAACAGCCTATGAGGGAAATTTTGGTGAAGTATA
Sample #3
MAN2A1
FER
ATTTCAGAAACAGCCTATGAGGGAAATTTTGGTGAAGTATA

Sample #114

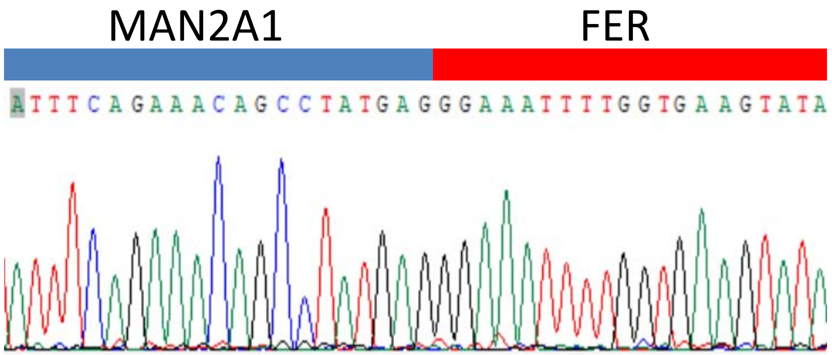

Sample #113

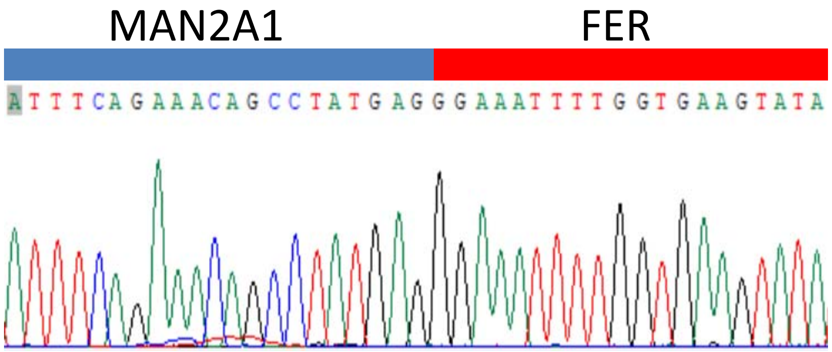

Sample #104

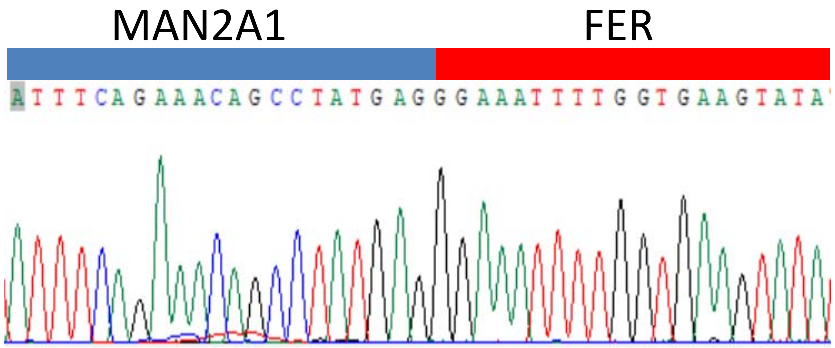

Sample #31

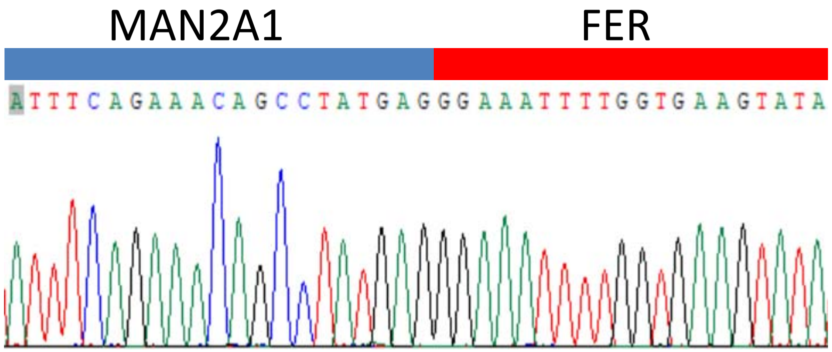

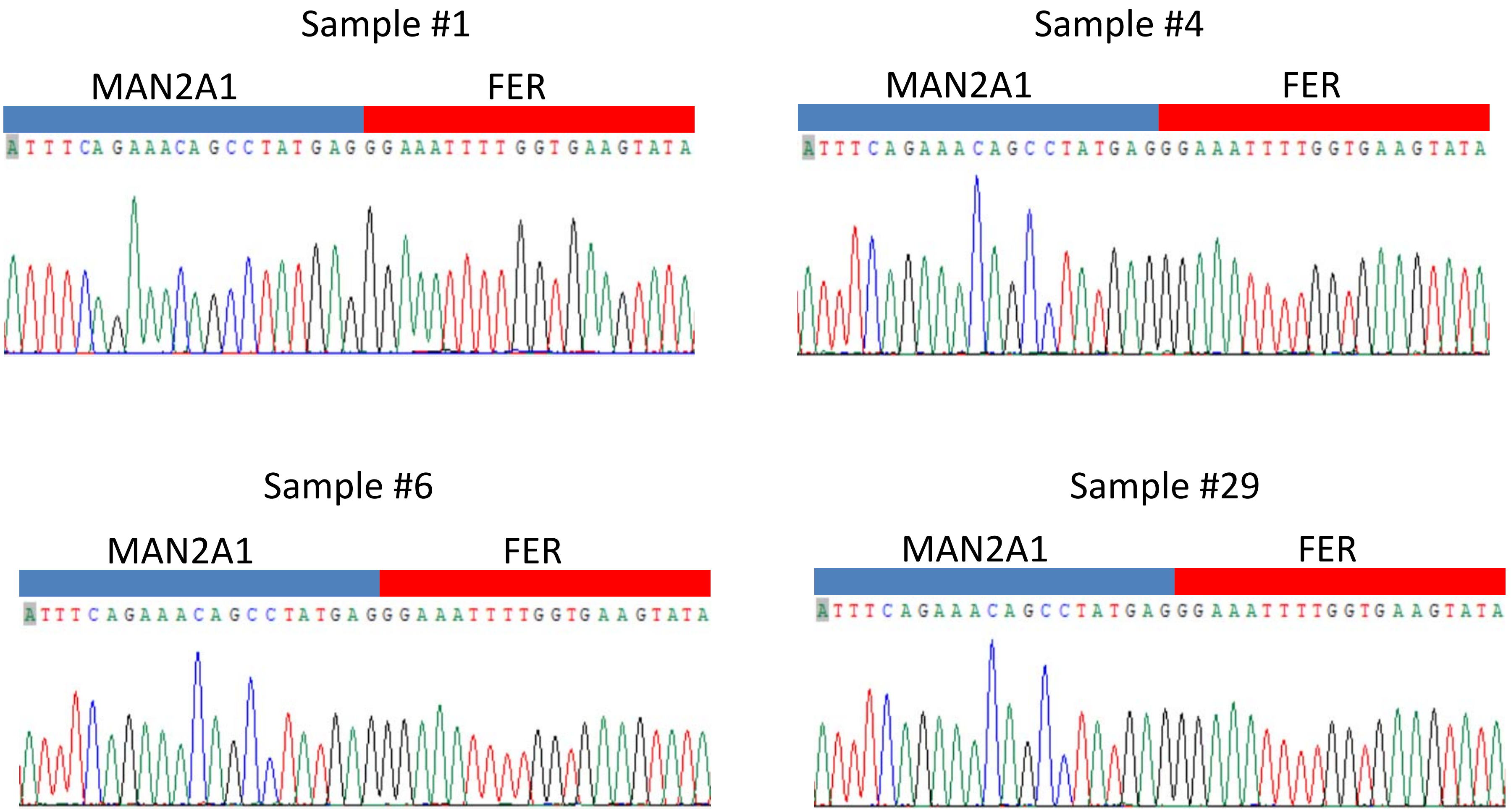

Sample #1
MAN2A1
FER
ATTTCAGAAACAGCCTATGAGGGAAATTTTGGTGAAGTATA
Sample #4
MAN2A1
FER
ATTTCAGAAACAGCCTATGAGGGAAATTTTGGTGAAGTATA
Sample #6
MAN2A1
FER
ATTTCAGAAACAGCCTATGAGGGAAATTTTGGTGAAGTATA
Sample #29
MAN2A1
FER
ATTTCAGAAACAGCCTATGAGGGAAATTTTGGTGAAGTATA

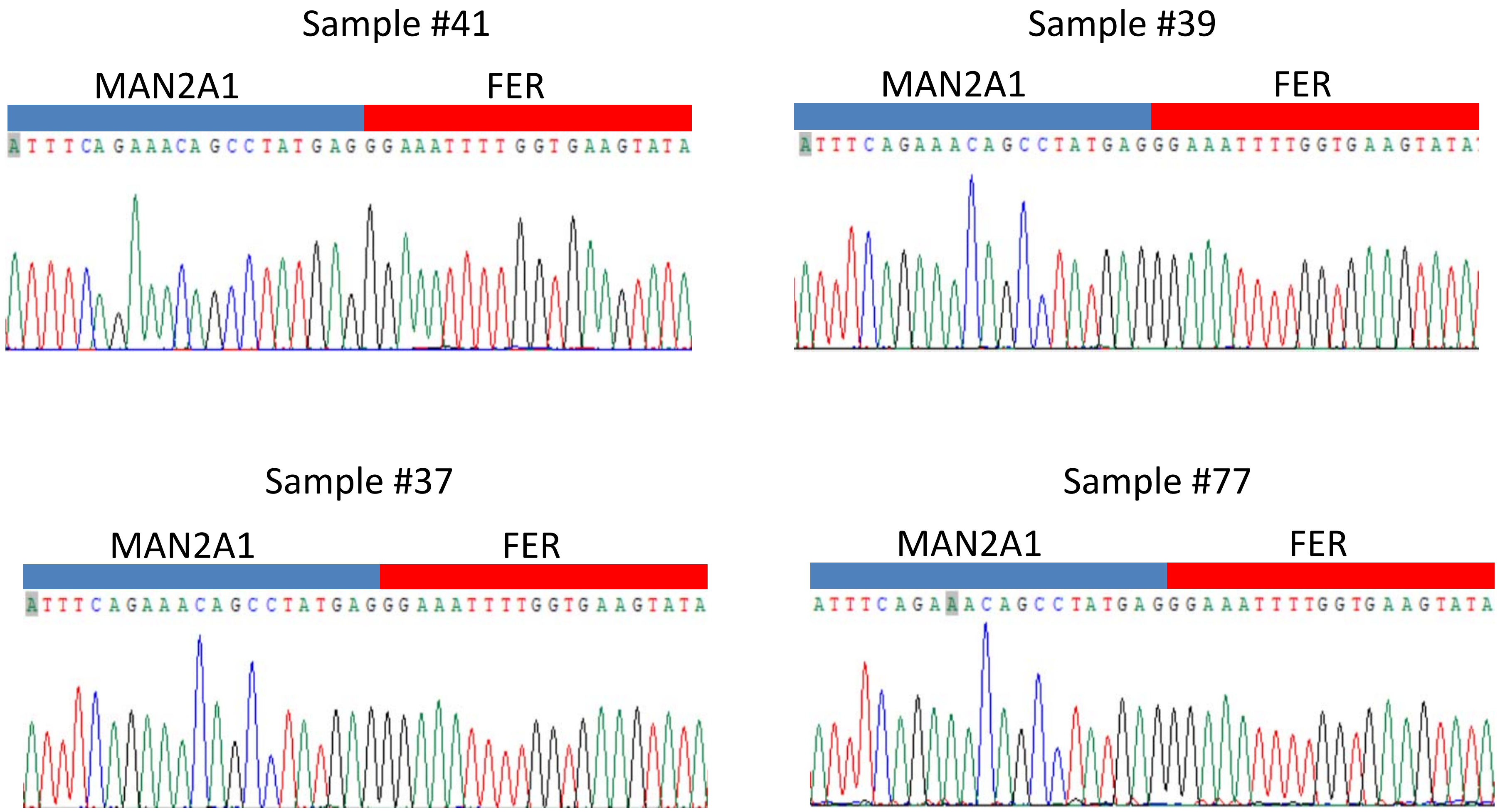
Sample #41
MAN2A1
FER
ATTTCAGAAACAGCCTATGAGGGAAATTTTGGTGAAGTATA
Sample #39
MAN2A1
FER
ATTTCAGAAACAGCCTATGAGGGAAATTTTGGTGAAGTATA
Sample #37
MAN2A1
FER
ATTTCAGAAACAGCCTATGAGGGAAATTTTGGTGAAGTATA
Sample #77
MAN2A1
FER
ATTTCAGAAACAGCCTATGAGGGAAATTTTGGTGAAGTATA

SLC45A2-AMACR

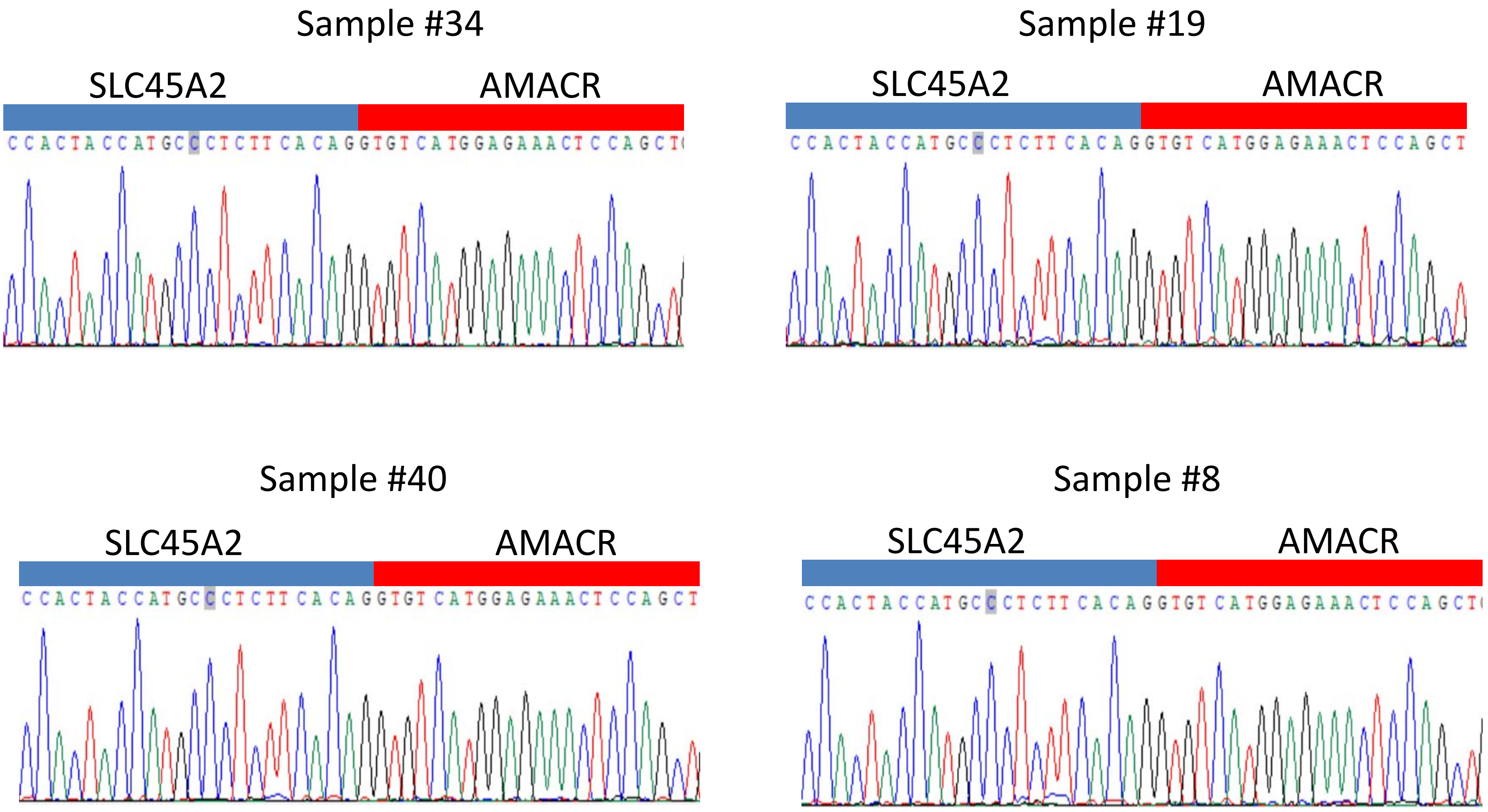

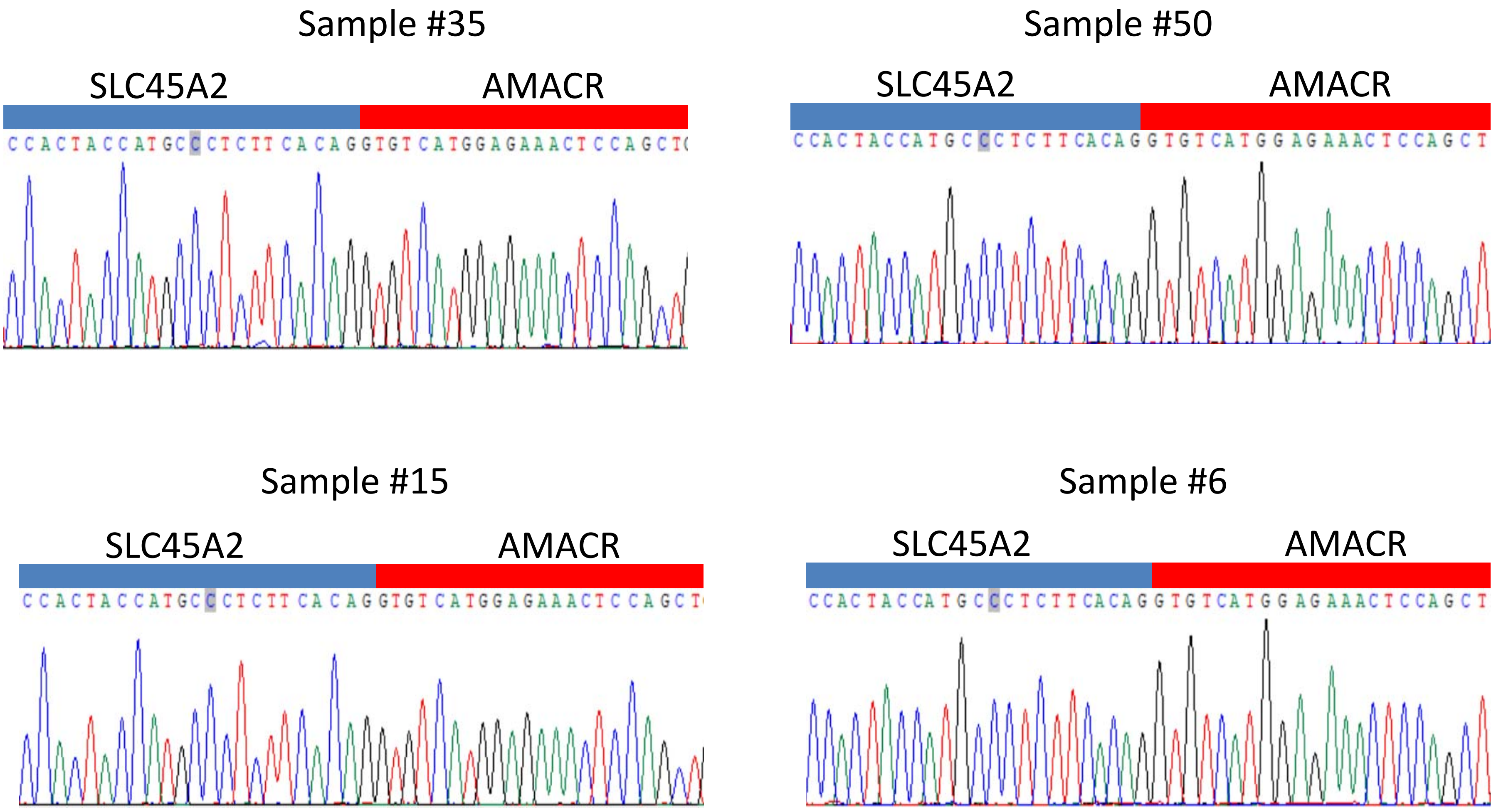

Sample #35
SLC45A2
AMACR
CCACTACCATGCCCTCTTCACAGGTGTCATGGAGAAACTCCAGCT
Sample #50
SLC45A2
AMACR
CCACTACCATGCCCTCTTCACAGGTGTCATGGAGAAACTCCAGCT
Sample #15
SLC45A2
AMACR
CCACTACCATGCCCTCTTCACAGGTGTCATGGAGAAACTCCAGCT
Sample #6
SLC45A2
AMACR
CCACTACCATGCCCTCTTCACAGGTGTCATGGAGAAACTCCAGCT

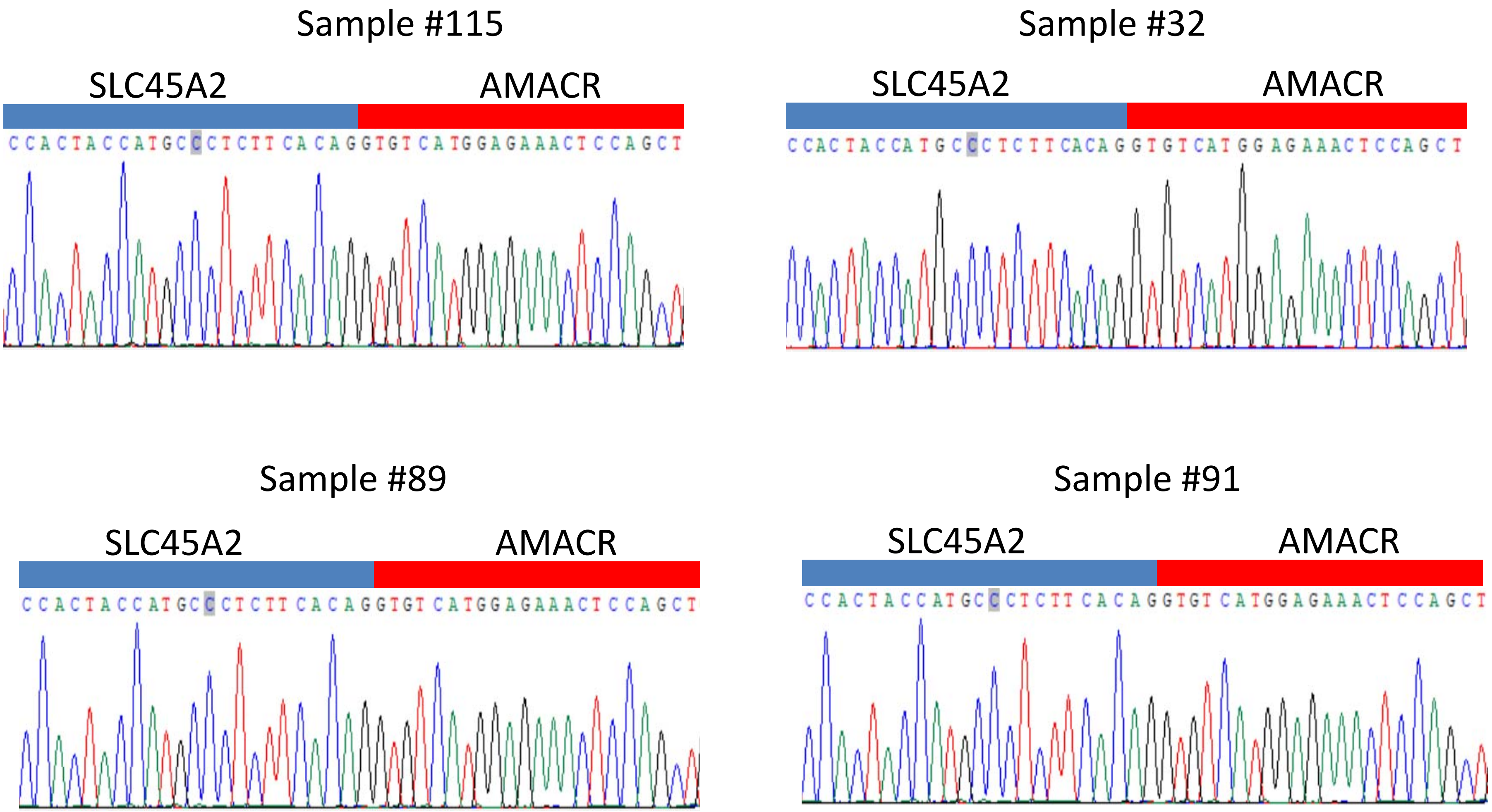

Sample #115
SLC45A2
AMACR
CCACTACCATGCCCTCTTCACAGGTGTCATGGAGAAACTCCAGCT
Sample #32
SLC45A2
AMACR
CCACTACCATGCCCTCTTCACAGGTGTCATGGAGAAACTCCAGCT
Sample #89
SLC45A2
AMACR
CCACTACCATGCCCTCTTCACAGGTGTCATGGAGAAACTCCAGCT
Sample #91
SLC45A2
AMACR
CCACTACCATGCCCTCTTCACAGGTGTCATGGAGAAACTCCAGCT

Pten-NOLC1

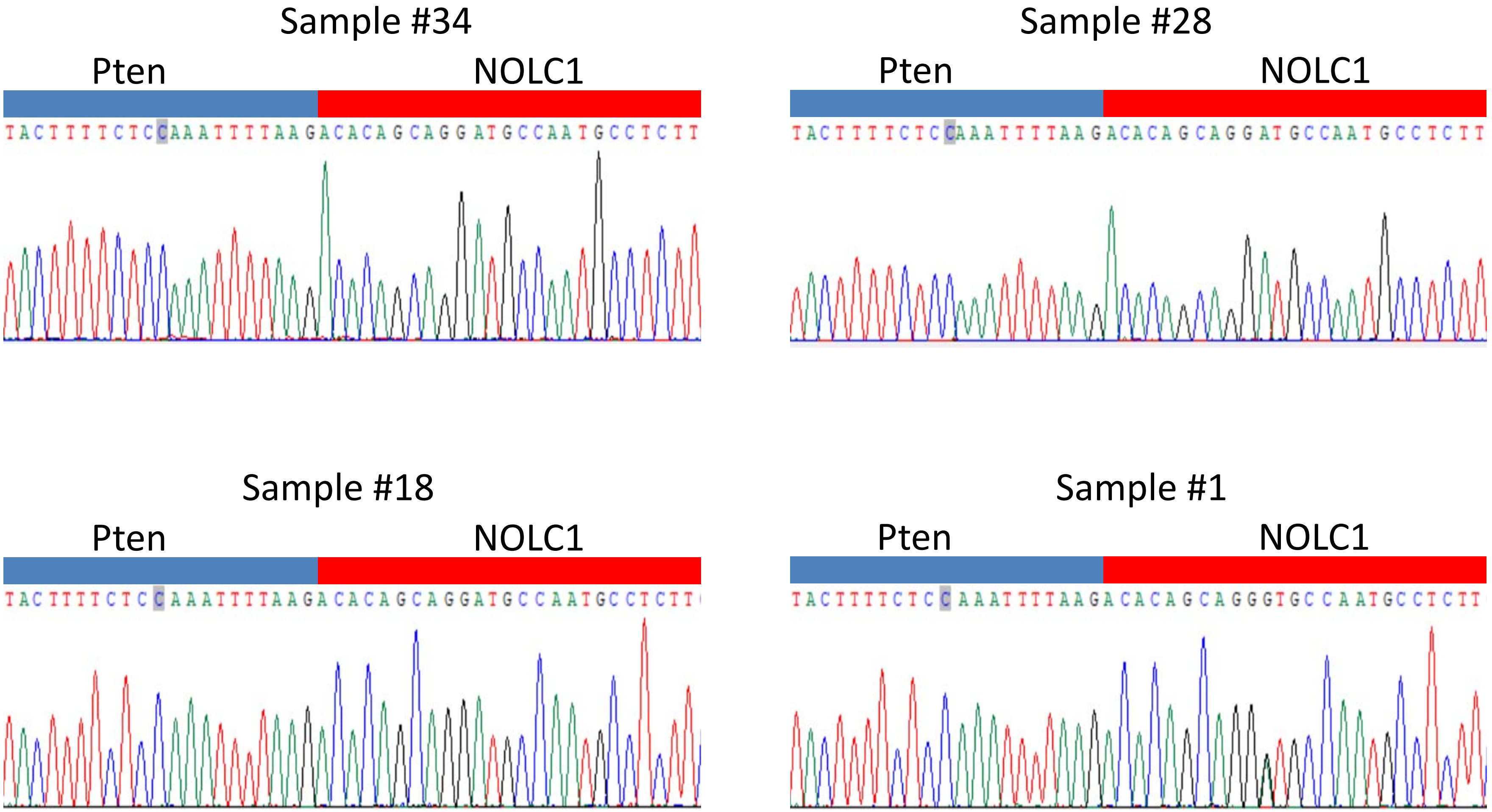
Sample #34
Pten
NOLC1
TACTTTTCTCCAAATTTTAAGACACAGCAGGATGCCAATGCCTCTT
Sample #28
Pten
NOLC1
TACTTTTCTCCAAATTTTAAGACACAGCAGGATGCCAATGCCTCTT
Sample #18
Pten
NOLC1
TACTTTTCTCCAAATTTTAAGACACAGCAGGATGCCAATGCCTCTT
Sample #1
Pten
NOLC1
TACTTTTCTCCAAATTTTAAGACACAGCAGGGTGCCAATGCCTCTT

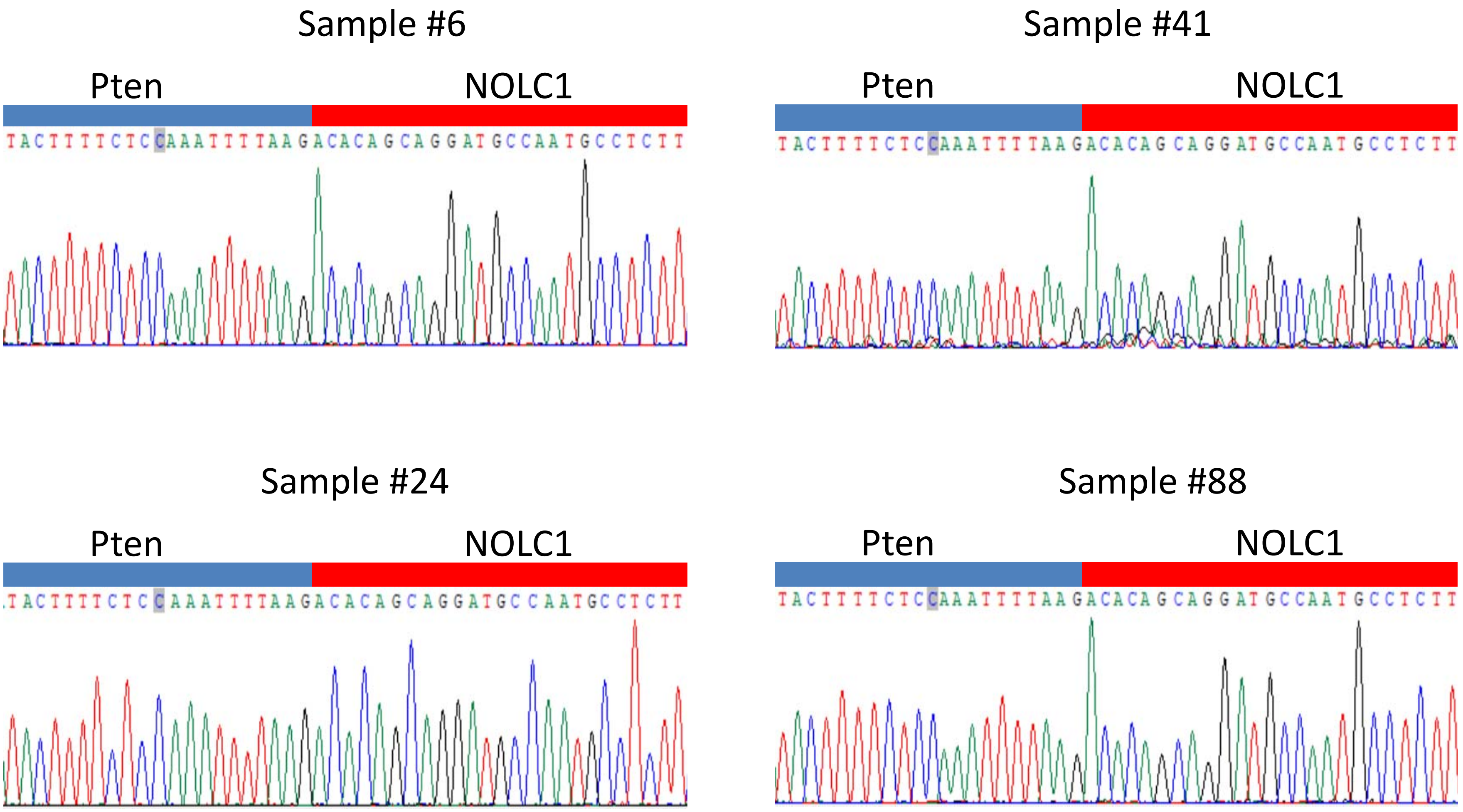

Sample #6
Pten
NOLC1
TACTTTTCTCCAAATTTTAAGACACAGCAGGATGCCAATGCCTCTT
Sample #41
Pten
NOLC1
TACTTTTCTCCAAATTTTAAGACACAGCAGGATGCCAATGCCTCTT
Sample #24
Pten
NOLC1
TACTTTTCTCCAAATTTTAAGACACAGCAGGATGCCAATGCCTCTT
Sample #88
Pten
NOLC1
TACTTTTCTCCAAATTTTAAGACACAGCAGGATGCCAATGCCTCTT

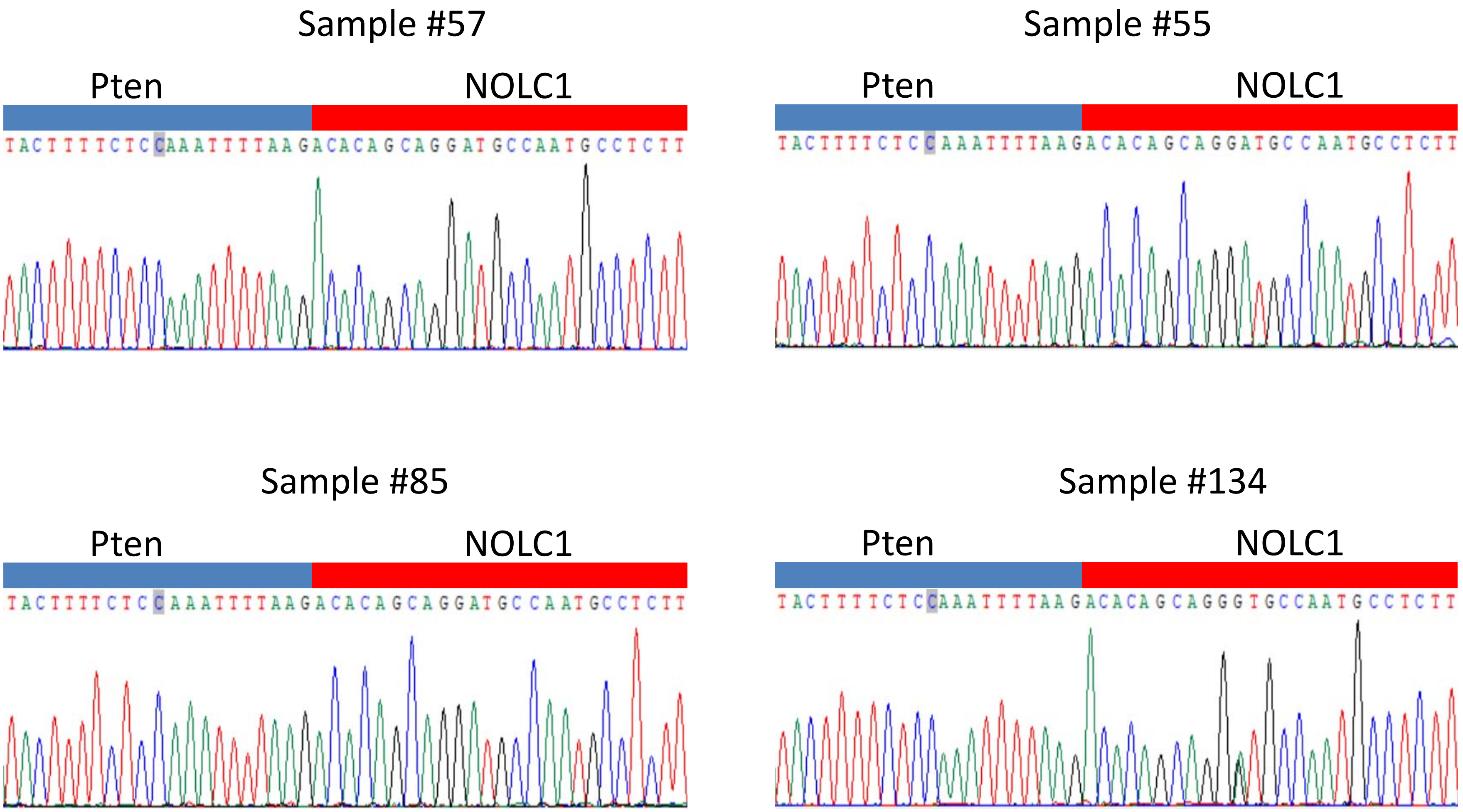
Sample #57
Pten
NOLC1
TACTTTTCTCCAAATTTTAAGACACAGCAGGATGCCAATGCCTCTT
Sample #55
Pten
NOLC1
TACTTTTCTCCAAATTTTAAGACACAGCAGGATGCCAATGCCTCTT
Sample #85
Pten
NOLC1
TACTTTTCTCCAAATTTTAAGACACAGCAGGATGCCAATGCCTCTT
Sample #134
Pten
NOLC1
TACTTTTCTCCAAATTTTAAGACACAGCAGGGTGCCAATGCCTCTT
