# Supplementary material for: Detection of fusion gene transcripts in the blood samples of prostate cancer patients
Source: Sci Rep. 2021 Aug 20;11:16995. doi: 10.1038/s41598-021-96528-9 (PMC8379170; doi:10.1038/s41598-021-96528-9)
Supplement: Supplementary file 3 — Supplementary Information 3. [file 41598_2021_96528_MOESM3_ESM.docx]

**Supplemental table 2. Primers and probes**

Fusion gene Primers Probe

MAN2A1-FER AGCGCAGTTTGGGATACAGCA/ 5’/56-FAM/TCAGAAAC A/ZEN/GCCTATGAGGGAAATT/3IABkFQ/3’

CTTTAATGTGCCCTTATATACTTCACC

SLC45A2-AMACR TTGATGTCTGCTCCCATCAGG/ 5'-/56-FAM/AAGAGGGCA/ZEN/TGGTAGTGGAGGC/3IABkFQ/-3'

CAGCTGGAGTTTCTCCATGAC

CCNH-C5orf30 AAAGTTATTTATCAGAGAGTCTGATGCTG/ 5'-/56-FAM/ACAGGCAAG/ZEN/TTCTGTTCTCTTTCAGCA/3IABkFQ/-3'

CTGTTCTACTCCAGGTATTTTCATTATATC

mTOR-TP53BP1 TGATAGACCAGTCCCGGGATG/ 5'-/56-FAM/ TGTCAGCCT/ZEN/GTCAGAATCCAAGTCAAG/3IABkFQ/-3'

CCACTGACATTCCCAGAACAAG

TRMT11-GRIK2 GCGCTGTCGTGTACCCTTAAC/ 5'-/56-FAM/CGGAACTCC/ZEN/AGATGCTCCTGCG/3IABkFQ/-3'

GAATGCAAGTTCCTCAGCTCC

LRRC59-FLJ60017 GTGACTGCTTGGATGAGAAGC/ 5'-/56-FAM/CAGTGTGCA/ZEN/AACAAGGTGACTGGAAG/3IABkFQ/-3'

CCCTCCTCTGGTTTGTTGTTG

TMEM135-CCDC67 CAGCTGTCATGGAAGTTCAGAC/ 5'-/56-FAM/AGTTCCTTT/ZEN/TAAGACTCACCAAGGGCAA/3IABkFQ/-3'

CCTCATTCTTTCCTGCTCAGAG

KDM4B- AC011523.2 AGACCACCTTCGCCTGGCAC/ 5'-/56-FAM/ACAGCATCA/ZEN/ACTACCTGCACTTTGGG/3IABkFQ/-3'

TCTCTCTCAGATCCAGGCTTG

Pten-NOLC1 CAAAGCAAATAAAGACAAAGCCAACCG/ 5'-/56-FAM/CAGGATGCC/ZEN/AATGCCTCTTCCC/3IABkFQ/-3'

GCCAGAAGCTATAGATGTCTAAGAG

β-actin ACCCCACTTCTCTCTAAGGAG/ 5'-/56-FAM/CCAGTCCTC/ZEN/TCCCAAGTCCACAC/3IABkFQ/-3’

GCAATGCTATCACCTCCCCTG
