# Supplementary material for: Detection of fusion gene transcripts in the blood samples of prostate cancer patients
Source: Sci Rep. 2021 Aug 20;11:16995. doi: 10.1038/s41598-021-96528-9 (PMC8379170; doi:10.1038/s41598-021-96528-9)
Supplement: Supplementary file 4 — Supplementary Information 4. [file 41598_2021_96528_MOESM4_ESM.docx]

**Supplemental table 3. Synthetic oligos corresponding to the fusion transcripts**

Fusion gene Synthetic oligo sequence

MAN2A1-FER TGGAAGTTCAAGTCAGCGCAGTTTGGGATACAGCAAATACTATTTCAGAAACAGCCTATGAGGGAA

ATTTTGGTGAAGTATATAAGGGCACATTAAAGGATAAAACTTCTGTTGCTGTTAAAACATGTAAAG

SLC45A2-AMACR GATGGGCCCATCAAAGCCTACTTATTTGATGTCTGCTCCCATCAGGACAAGGAGAAGGGCCTCCACTACCATGCCCTCT TCACAGGTGTCATGGAGAAACTCCAGCTGGGCCCAGAGATTCTGCAGCGGGAAAAT

CCNH-C5orf30 TTACTATGGAAAGTTATTTATCAGAGAGTCTGATGCTGAAAGAGAACAGAACTTGCCTGTCACAGTTACTAGATATAATG AAAATACCTGGAGTAGAACAGAAAAATTATTATGTCTGTGTTCCCTTGGGACTCATTG

mTOR-TP53BP1 TTGGATCCTTACAAGCACAAAGTGAACATTGGCATGATAGACCAGTCCCGGGATGCCTCTGCTGTCAGCCTGTCAGA

ATCCAAGTCAAGTCAGGATTCCTTGTTCTGGGAATGTCAGTGGAATCTGCTCCTGCTGTGGAGGAAGAGAAGGGA

TRMT11-GRIK2 GCAGCTGCAATGGCGCTGTCGTGTACCCTTAACAGGTATCTGCTCCTCATGGCGCAGGAGCATCTGGAGTTCCGCCTGC CGGTGGTATTTTTGAATATGTGGAATCTGGCCCAATGGGAGCTGAGGAACTTGCATTCAGATTTGCTGT

LRRC59-FLJ60017 TGGCAGGTGACTGCTTGGATGAGAAGCAGTGTAAGCAGTGTGCAAACAAGGTGACTGGAAGCACCTGCTCAATGGCTGC

TCATCAACAACAAACCAGAGGAGGGCTTA

TMEM135-CCDC67 GCAGCTGTCATGGAAGTTCAGACTTTGAGACCATCTTACTGGAAGTTCCTTTTAAGACTCACCAAGGGCAAATAAGAAG CCAACTCCAACAGGTGGAAGAGTACCATAACTCTGAGCAGGAAAGAATGAGGAAT

KDM4B- AC011523.2 GGAAGACCACCTTCGCCTGGCACACCGAGGACATGGACCTGTACAGCATCAACTACCTGCACTTTGGGGAGCCTA AGTCCTGGACAGTAAGCAAGCCTGGATCTGAGAGAGATATCATCTTGCAAGGATGCCTGCTTTACAA

Pten-NOLC1 CTTACTTTAACAAAAAATGATCTTGACAAAGCAAATAAAGACAAAGCCAACCGATACTTTTCTCCAAATTTTAAGAC ACAGCAGGATGCCAATGCCTCTTCCCTCTTAGACATCTATAGCTTCTGGCTCAAG

β-actin TGAGATGCGTTGTTACAGGAAGTCCCTTGCCATCCTAAAAGCCACCCCACTTCTCTCTAAGGAGAATGGC

CCAGTCCTCTCCCAAGTCCACACAGGGGAGGTGATAGCATTGCTTTCGTGTAAATTATGTAATGCAAAAT
